# A flow platform for degradation-free CuAAC bioconjugation

**DOI:** 10.1038/s41467-018-06551-0

**Published:** 2018-10-01

**Authors:** Marine Z. C. Hatit, Linus F. Reichenbach, John M. Tobin, Filipe Vilela, Glenn A. Burley, Allan J. B. Watson

**Affiliations:** 1 Department of Pure and Applied Chemistry, University of Strathclyde, 295 Cathedral Street, Glasgow, G1 1XL UK; 2 Chemical Sciences, Heriot-Watt University, Edinburgh, EH14 4AS UK; 3 School of Chemistry, University of St Andrews, North Haugh, St Andrews, KY16 9ST UK

## Abstract

The Cu-catalyzed azide-alkyne cycloaddition (CuAAC) reaction is a cornerstone method for the ligation of biomolecules. However, undesired Cu-mediated oxidation and Cu-contamination in bioconjugates limits biomedical utility. Here, we report a generic CuAAC flow platform for the rapid, robust, and broad-spectrum formation of discrete triazole bioconjugates. This process leverages an engineering problem to chemical advantage: solvent-mediated Cu pipe erosion generates ppm levels of Cu in situ under laminar flow conditions. This is sufficient to catalyze the CuAAC reaction of small molecule alkynes and azides, fluorophores, marketed drug molecules, peptides, DNA, and therapeutic oligonucleotides. This flow approach, not replicated in batch, operates at ambient temperature and pressure, requires short residence times, avoids oxidation of sensitive functional groups, and produces products with very low ppm Cu contamination.

## Abstract (web summary)

Cu-catalyzed azide-alkyne cycloaddition (CuAAC) reaction is a common bioconjugation technique; however oxidative degradation and residual copper may limit its use. Here, the authors show that ppm levels of Cu, generated in situ by solvent-mediated pipe erosion in flow, catalyze the CuAAC reaction with no product degradation.

## Introduction

The Cu-catalyzed azide-alkyne cycloaddition (CuAAC) reaction (Scheme 1a) is a method of widespread utility throughout medicinal chemistry, chemical biology, and the material sciences^1–6^. The pervasiveness of this methodology can be attributed to the rapid, chemo- and regiospecific generation of 1,2,3-triazole products and bioconjugates.

**Fig. 1:**
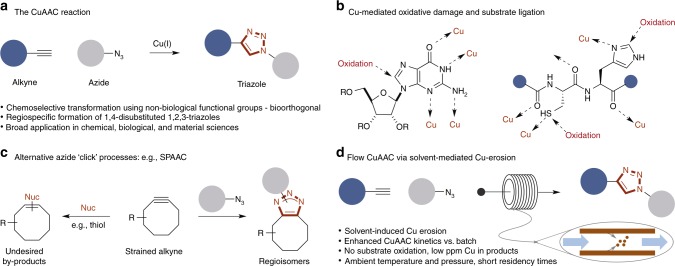
Azide-alkyne cycloaddition strategies. **a** The archetypical CuAAC reaction; **b** Examples of oxidatively labile and ligating functional groups found in biomolecules; **c** The SPAAC reaction and and the formation of regioisomeric triazole products; **d** The flow-assisted CuAAC reaction (this work). CuAAC Cu-catalyzed azide-alkyne cycloaddition, Nuc nucleophile, SPAAC strain-promoted azide-alkyne cycloaddition

A significant limitation of the CuAAC reaction conducted under batch conditions is the need for a Cu catalyst; this can be problematic in a number of applications^5,6^. Cu-mediated oxidative damage of sensitive functional groups can result in product mixtures, which may complicate purification or lead to issues with bioassays due to the need for deconvolution of data or unknown pharmacology (Fig. 1b). In biomolecule tagging CuAAC modification of azide/alkyne biomolecules requires (super)stoichiometric loadings of Cu catalyst due to the presence of a number of Cu-chelating sites (e.g., *N*/*S* sites of peptides^7^, N7 of purines in nucleic acids^8^), which can result in catalyst inhibition and the need for higher concentrations of Cu in the reaction (Fig. 1b)^6^. In addition, oxidative damage of biomolecules is a significant issue associated with current CuAAC-based bioconjugation strategies, severely limiting development^9–11^. These issues have inspired the development of a series of alternative Cu-free click approaches such as strain-promoted azide-alkyne cycloadditions (SPAAC)^12^ and inverse electron demand Diels-Alder (IEDDA) approaches using tetrazines^13^. Despite their moderate to fast kinetics^14^, these processes have their own issues; for example, lacking the chemo- and regiospecificity afforded by the CuAAC reaction due to the reactive (electrophilic) nature of the requisite cyclic alkynes/alkenes^15–21^, which are susceptible to side reactions with nucleophilic residues (e.g., thiol residues in glutathione). Furthermore, the installation of these large lipophilic groups has a significant impact on the overall physicochemical properties of the bioconjugate (Fig. 1c)^22^.

Whilst efforts have been made to overcome the oxidation and Cu contamination issues of the CuAAC reaction by the development of bespoke ligands, conducting these reactions under anaerobic conditions, the addition of oxidation inhibitors, and Cu scavengers, these issues extend from the requirement for high [Cu] to overcome slow catalytic turnover as a result of the numerous Lewis-basic groups typically found in proteins and nucleic acids^23^.

Here we describe the development of a rapid flow-assisted CuAAC reaction that overcomes these problems (Fig. 1d). Our operationally simple strategy couples solvent-induced erosion of a copper tube with the formation of a highly active CuAAC catalyst under laminar flow conditions. This enables the facile formation (*t*_R_ ca. 1–10 min) of discrete ligation products and bioconjugates not possible using conventional batch conditions. Significantly, the level of Cu present in products is well below the reported mammalian cellular toxicity thresholds (e.g., <20 μM for DNA)^5,11,24^ with no associated oxidative damage observed on a series of representative labile biomolecules, including peptides and DNA strands.

## Results

### Reaction design

Flow-based technologies offer distinct advantages over batch, such as enhanced mass transfer, which is particularly advantageous for large molecular weight biomolecules where accessibility of functional groups is significantly compromised in the batch regime^25–29^. Despite these advantages, application of flow-based CuAAC bioconjugation has not been reported due to the need for (i) excess Cu catalyst, which promotes biomolecule degradation, (ii) ionic scavengers, which can result in residual Cu trapped in bioconjugates, (iii) elevated temperatures, which promotes biomolecule degradation, and (iv) organic solvents, which typically limits biocompatibility. This has limited flow CuAAC applications to small molecules and prevented the widespread development of flow-assisted synthesis of discrete bioconjugates^30–41^.

Whilst elemental Cu is an effective catalyst for flow CuAAC, elevated temperature and pressures are required, likely in order to solubilize some Cu in the eluent. However, H_2_O/organic mixtures are extremely effective and biocompatible solvent mixtures for CuAAC-mediated bioconjugation. In addition, the surface of Cu pipes is typically covered in a protective oxide layer, which are generally poorly soluble in organic solvents but more soluble in H_2_O. Indeed, erosion of Cu tubes with H_2_O is a well-studied engineering phenomenon, with Cu leaching a known problem in flow chemistry^31^.

Based on this, we hypothesized that an aqueous/organic mixture (for example, H_2_O/MeCN) would offer a blend of sufficient solubility of the (bio)organic components while promoting controlled erosion of surface Cu salts under laminar flow conditions, with in situ Cu(I)/Cu(II) disproportionation providing the mechanistically essential Cu(I) required for the CuAAC reaction. Whilst the level of solubilized Cu was likely to be very low; the increased circulation established under the flow set up would enhance mass transport to provide reaction efficiency not possible in batch^26^.

**Fig. 2:**
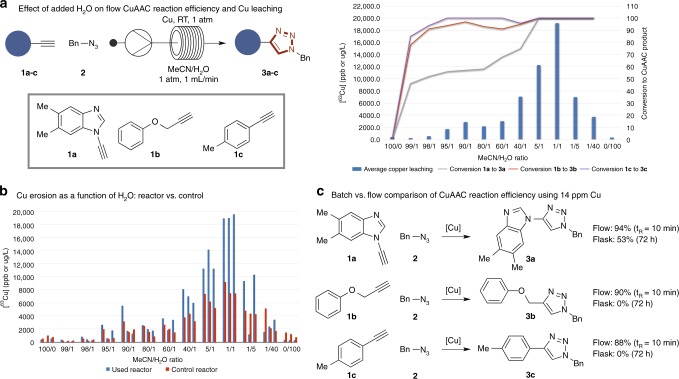
Development of a flow CuAAC process based on H_2_O Cu erosion. **a** The effect of H_2_O on the CuAAC reaction efficiency using three representative alkynes; **b** Correlation of [Cu] vs. solvent composition ((H_2_O]); **c** Demonstration of the flow effect—14 ppm Cu CuAAC reactions in flow and in flask

This hypothesis was found to be valid. Three benchmark CuAAC reactions, using three alkynes (**1a**–**1c**), with known differences in reactivity with benzyl azide (**2**) were evaluated in a laminar flow system comprising a pump and copper reactor at ambient temperature and pressure (Fig. 2a). The reaction does not proceed in pure MeCN or pure H_2_O and [Cu] in the eluent (10 mL collected, 1 mL/min under ambient conditions) was extremely low (<20 ppm). However, the addition of small amounts of H_2_O to the bulk MeCN resulted in the formation of triazole **3a–c**, which peaked at 5:1 solvent mixture. Control experiments with an unused Cu reactor (Fig. 2b, red bars) vs. a reactor used for CuAAC reactions (Fig. 2b, blue bars) demonstrated greater erosion in the used reactor, consistent with a more exposed surface due to repeated chemistry; however the solvent composition/erosion trend was comparable, peaking at 1:1 H_2_O/MeCN. The addition of small percentages of H_2_O to the carrier solvent (MeCN) enabled the CuAAC reaction of equistoichiometric ynamine **1a** and BnN_3_ (**2**) effectively at 5:1 MeCN:H_2_O (*t*_R_ = 10 min; Fig. 2a). Whilst ynamine **1a** exhibits faster batch-reaction kinetics based on a p*K*_*a*_ modifying Cu-ligation^42^, the mixed solvent system was also effective at enabling the CuAAC reaction of more standard alkynes **1b** and **1c** at the same flow rate. Analysis of the eluent by ICP-MS revealed that [Cu] was ~14 ppm, which is well below the limit required for use in in vivo applications^5,11^. Importantly, control experiments identified a flow phenomenon. Attempting the CuAAC reaction of alkynes **1a–1c** in flask experiments at 14 ppm Cu was unsuccessful for **1b** and **1c** and only moderately successful for the more reactive ynamine **1a** (53% yield after 72 h), whereas the flow system results in quantitative conversion in 10 min (Fig. 2c). Residence times were also shortened significantly to ca. 1 min for more reactive substrates.

### Scope of the flow platform

**Fig. 3:**
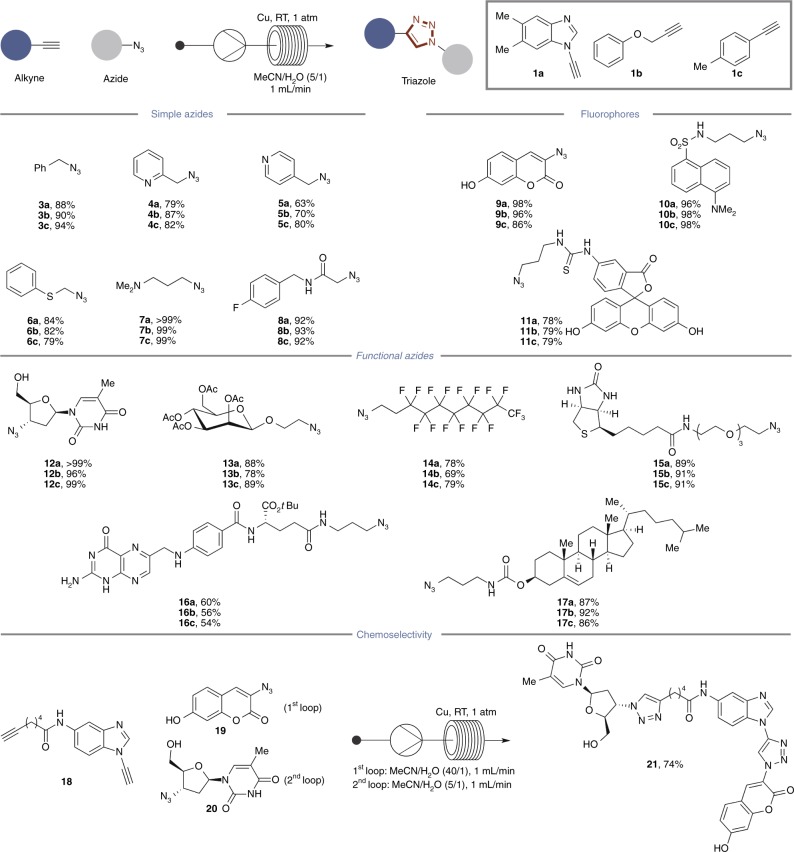
Scope of the flow CuAAC process. For each product number (in bold), data are reported as percent isolated yield. Products with designation **a** are derived from alkyne **1a**, **b** from alkyne **1b**, and **c** from alkyne **1c**

The scope of the flow CuAAC process was both broad and reproducible using three different alkyne classes (**1a**–**1c**) across a series of azide substrates (**3**–**20**; Fig. 3). Triazole products derived from simple azides, azido fluorophores, and azide possessing specific functions for downstream applications, were all isolated in high yield after a single pass. Importantly, ICP-MS analysis of the products again found the residual [Cu] was <20 ppm (see Supporting Information for full details).

We also examined the compatibility of the flow process with regards to established CuAAC chemoselectivity profiles (Fig. 3). Diyne **18**, containing aliphatic alkyne and aromatic ynamine sites, underwent sequential CuAAC ligation, firstly with the coumarin azide **19** at the ynamine site followed by ligation with the nucleobase azide **20** at the aliphatic alkyne site; complete chemoselectivity was observed throughout. This demonstrates that established reactivity profiles^43^ are replicated in the flow format and that our system enhances not only overall reaction kinetics but does so at very low [Cu].

**Fig. 4:**
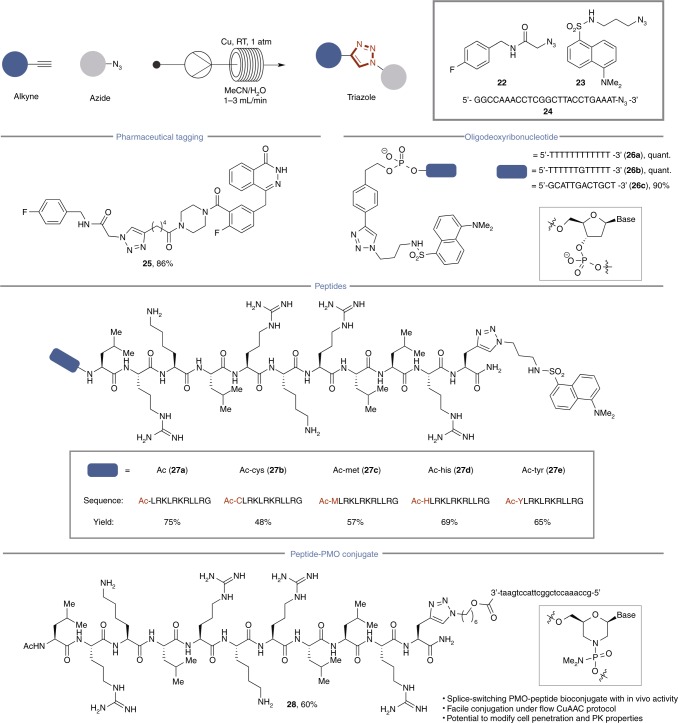
Alkyne scope of the flow CuAAC process. For each product number (in bold), data are reported as percent isolated yield

The biomedical utility of the CuAAC reaction lies primarily in the ligation of bio-relevant molecules. We assessed the flow CuAAC process as a method for the ligation of representative alkyne-derivatives of nucleic acids and peptides, which have known susceptibility to form oxidized byproducts in the presence of a Cu catalyst (Fig. 4)^8,10^. Installation of a fluorinated residue onto a marketed PARP inhibitor^44^, and a common fluorophore onto a series of peptides and DNA strands containing oxidizable functionality produced triazole products with minimal formation of side-products. These include CuAAC ligations with oligodeoxyribonucleotides (ODNs) and the core ApoliproteinE (ApoE) peptide sequence (**27**)^45^, which has demonstrated utility as a delivery vehicle across the blood brain barrier^46^. Residues with known oxidative susceptibility (**27a–e**) under conventional CuAAC batch conditions were installed on the *N*-terminus to report any potential degradation by reactive oxygen species and formed the expected triazole products (1 mL/min; *t*_R_ = 8 min), with trace Cu contamination and no associated degradation.

### Bioconjugation

Finally, we explored applying our flow-based CuAAC ligation approach to prepare therapeutic bioconjugates. Phosphoramidate morpholino oligonucleotides (PMOs) are a class of oligonucleotides with established therapeutic importance^47–49^. An essential requirement for in vivo efficacy of this class of biologics is the need conjugate a cell penetrating peptide sequence onto one of the termini to enable effective delivery to the central nervous system. The bioconjugate triazole **28** was prepared from precursors derived from a PMO azide with known in vivo efficacy as a splice-switching oligonucleotide for the treatment of Spinal Muscular Atrophy (SMA) and a peptide fragment derived from a portion of the ApoE protein^50^. Under flow conditions, the ApoE-PMO bioconjugate (**28**) was formed in 60% yield after 15 passes (1 mL/min; total *t*_R_ = 30 min). No reaction was observed after 24 h under equivalent batch conditions, with only only 26% yield of **28** obtained in batch after 48 h using 100 equiv Cu.

## Discussion

In summary, we have developed a rapid and operationally simple flow-based platform for the CuAAC reaction that operates at ambient temperature and pressure. Solvent-induced erosion of a Cu pipe provides catalytically competent Cu to promote the CuAAC reaction of a range of both small molecules and biomolecules without oxidative damage to labile functional groups and with trace Cu contamination. We have demonstrated the dependency of the system on the composition of the medium and that the observed effect is unique to the flow conditions with comparable isolated experiments of low efficiency. We expect that these findings will significantly increase the utility of flow-assisted CuAAC across a series of academic and industrial applications.

## Methods

### General methods

See Supplementary Methods for further details supporting experiments, Supplementary Tables 1–11 for additional data, and Supplementary Figures 1–125 for spectra.

### General procedure for the flow CuAAC process

Alkyne (0.2 mmol) and azide (0.2 mmol) were dissolved in 10 mL of MeCN/H_2_O (5/1). The CuAAC reactions were carried out in a commercial chemical flow reactor equipped with a 10 mL copper reactor (easy-Scholar from Vapourtec). The reaction mixture was flowed through a copper tube (diameter = 1 mm, volume = 10 mL, surface area = 400 cm^2^) at a flow rate of 1 mL/min at rt (25 °C, *t*_R_ = 10 min). The reaction mixture was then collected and concentrated in vacuo to afford the crude product. Where necessary, purification was achieved by flash silica column chromatography (for small molecule products) or preparative HPLC (for peptide/DNA-based products).

## Electronic supplementary material

Supplementary Information

Peer Review File

## Data Availability

All data generated or analyzed during this study are included in this published article (and its supplementary information files). These data are also available from the author upon request.
