# Supplementary material for: A flow platform for degradation-free CuAAC bioconjugation
Source: Nat Commun. 2018 Oct 1;9:4021. doi: 10.1038/s41467-018-06551-0 (PMC6167327; doi:10.1038/s41467-018-06551-0)
Supplement: Supplementary file 1 — Supplementary Information [file 41467_2018_6551_MOESM1_ESM.pdf]

A flow platform for degradation-free CuAAC bioconjugation

Hatit et al.

Contents

Abbreviations	3
Supplementary Methods	3–10
Supplementary Discussion	10–45
Supplementary Figures 7–125	46–111
Supplementary References	112–113

Abbreviations

ApoE	Apolipoprotein E
CuAAC	Copper-catalysed azide alkyne cycloaddition
DNA	Deoxyribonucleic acid
equiv.	Equivalent
RP-HPLC	Reverse phase - High pressure liquid chromatography
HRMS	High-resolution mass spectrometry
ICP-MS	Inductively coupled plasma mass spectrometry
MALDI-TOF	Matrix-assisted laser desorption/ionisation-time of flight spectrometer
NMR	Nuclear magnetic resonance spectroscopy
ODN	Oligodeoxyribonucleotide
PE	Petroleum ether
PFA	Perfluoroalkoxy alkane
PMO	Phosphorodiamidate morpholino oligomer
rt	Room temperature
R_T	Retention time
TEAA	Triethylammonium acetate
TLC	Thin layer chromatography
t_R	Residence time
SM	Starting material

Supplementary Methods

Starting materials, reagents, and solvents were purchased from commercial sources and used as received unless stated otherwise. Purification of reaction products was carried out according to standard laboratory methods.[1] Thin layer chromatography (TLC) was carried out using Merck silica plates coated with fluorescent indicator UV254. TLC plates were analyzed under 254 nm UV light or developed using potassium permanganate solution. Normal-phase flash chromatography was carried out using ZEOprep 60 HYD 40-63 µm silica gel. ^{1}H spectra were acquired on a Bruker AV400 spectrometer at 400 MHz or on a Bruker AV500 spectrometer at 500 MHz. ^{13}C NMR spectra were acquired on a Bruker AV400 spectrometer at 100 MHz or on a Bruker AV500 spectrometer at 125 MHz. Proton-decoupled ^{19}F spectra were acquired on a Bruker AV400 spectrometer at 376 MHz. Chemical shifts are reported in ppm and coupling constants are reported in Hz with $CDCl_3$ referenced at 7.26 (^{1}H) and 77.16

ppm (^{13}C), DMSO-d$_6$ referenced at 2.50 (^1H) and 39.52 (^{13}C), and MeOD referenced at 3.31 (^1H) and 49.0 (^{13}C) ppm. Despite numerous attempts, varying both relaxation time and number of scans, it was not possible to obtain ^{13}C spectra for compounds **11a-c**, **14a**, **S2**, **21** and **25**.[2] High-resolution mass spectra (HRMS) were obtained through analysis at the EPSRC UK National Mass Spectrometry Facility at Swansea University. Samples were analysed by ICP-MS using an Agilent 7500ce (with octopole reaction system), employing an rf forward power of 1540 W and reflected power of 1 W, with argon gas flows of 0.81 L min^{-1} and 0.22 L min^{-1} for carrier and makeup flows, respectively. Sample solutions were taken up into the Micro mist nebulizer by free aspiration at a rate of approximately 1.0 mL min^{-1}. Skimmer and sample cones were made of nickel. MALDI-TOF mass spectra were recorded using a Shimadzu Biotech Axima CFR spectrometer. The matrices used were sinapinic acid for PMO substrates and 3-hydroxypicolinic acid for DNA substrates.

The preparation of 1-ethynyl-5,6-dimethyl-1*H*-benzo[*d*]imidazole **1a**,[3] 2-(azidomethyl)pyridine **4**,[4] 4-(azidomethyl)pyridine **5**,[4] 3-azido-*N*,*N*-dimethylpropan-1-amine **7**,[5] 2-azido-*N*-(4-fluorobenzyl)acetamide **8**,[6] 3-azido-7-hydroxy-2*H*-chromen-2-one **9**,[7] *N*-(3-azidopropyl)-5-(dimethylamino)naphthalene-1-sulfonamide **10**,[8] 1-(3-azidopropyl)-3-(3,6-dihydroxy-3'-oxo-3'*H*,10*H*-spiro[anthracene-9,1'-isobenzofuran]-5'-yl)thiourea **11**,[9] (2*R*,3*R*,4*S*,5*S*,6*R*)-2-(acetoxymethyl)-6-(2-azidoethoxy)tetrahydro-2*H*-pyran-3,4,5-triyl triacetate **13**,[10] 10-azido-1,1,1,2,2,3,3,4,4,5,5,6,6,7,7,8,8-heptadecaluorodecane **14**,[11] *tert*-butyl N_2-(4-(((2-amino-4-oxo-1,4-dihydropteridin-6-yl)methyl)amino)benzoyl)-N_5-(3-azidopropyl)-*L*-glutaminate **16**[12,13] and *N*-(1-ethynyl-1*H*-benzo[*d*]imidazol-6-yl)hept-6-ynamide **18**[14] was achieved by following literature procedures. The 5' azido functionalized PMO (5'-ggccaaacctcggcttacctgaaat-3') was purchased commercially (Gene Tools).

General Procedure A for the synthesis of triazoles under CuAAC flow conditions

Alkyne (0.2 mmol) and azide (0.2 mmol) were dissolved in 10 mL of MeCN/H_2O (5/1). The CuAAC reactions were carried out in a commercial chemical flow reactor equipped with a 10 mL copper reactor (easy-Scholar from Vapourtec). The reaction mixture was flowed through a copper tube (diameter = 1 mm, volume = 10 mL, surface area = 400 cm^2) at a flow rate of 1 mL/min at rt (25 °C, t_R = 10 min). The reaction mixture was then collected and concentrated *in vacuo*.

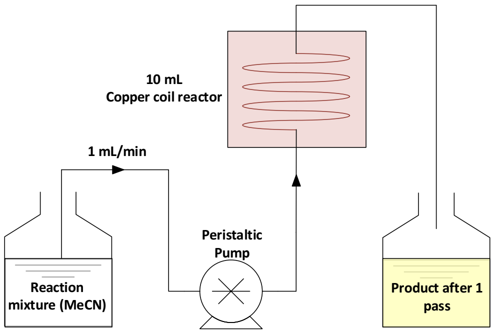

Supplementary Figure 1. Schematic of the experimental setup using the easy-Scholar flow reactor from Vapourtec.

General Procedure B for the solid phase synthesis of oligodeoxyribonucleotides (ODNs)

ODNs were synthesized using phosphoramidite-based solid phase synthesis protocols on an Applied Biosystems 392 DNA/RNA synthesizer. Phosphoramidites and controlled pore glass (CPG) supports were loaded with standard nucleotides were purchased from LINK Technologies Ltd (Bellshill, UK). For the synthesis of ODNs containing the modified alkyne phosphoramidite **S3** (see page 31) installed on the 5' end, longer coupling times of 8 minutes were used. TIPS deprotection was performed using a solution of 50 µL TBAF (1 M in THF) in 1.95 mL of MeCN. The mixture was repeatedly passed over the column containing the CPG support for 3 min. The CPG support was washed with MeCN (3 × 5 mL) and dried with air. Ammonia (DNA grade, 1.5 mL/mmol) was added and the suspension was shaken for 16 hours at room temperature. The supernatant was removed, and the CPG support was then washed with water (2 × 1.5 mL/mmol). The combined aqueous phase were lyophilized and purified by RP-HPLC with a Dionex UltiMate 3000 HPLC using a preparative column (Phenomenex Clarity 5 µM Oligo-RP, 250 × 10 mm) at a flow rate of 3 mL/min, gradient 10–50% 0.1 M triethylamonium acetate (TEAA) in MeCN in 32 min or 10–40% 0.1 M TEAA in MeCN in 22 min. Purity of resultant products was assessed with a Shimadzu Prominence HPLC using an analytical column (Phenomenex Clarity 5 µM Oligo-RP, 250 × 4.6 mm) at a flow rate of 1 mL/min, gradient 10–50% 0.1 M TEAA in MeCN in 35 min. Yields were calculated by measuring the absorbance of the products using extinction coefficient values of 97800 L/(mol × cm) for **ODN1**, 100400 L/(mol × cm) for **ODN2** and 108700 L/(mol × cm) for **ODN3**.

Product	Sequence	Yield (%)[a]
ODN1	5'-**P**$_t$TTT TTT TTT TTT-3'	16
ODN2	5'-**P**$_t$TTT TTT GTT TTT-3'	10
ODN3	5'-**P**$_t$GCA TTG ACT GCT-3'	26

Supplementary Table 1. ODNs prepared in this study. [a] Isolated yields after RP-HPLC purification.

P$_t$ =

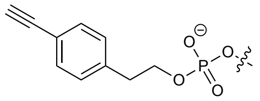

General Procedure C for CuAAC reactions using alkyne-modified ODNs

ODN1-3 (1 equiv) and azide (20 equiv) were dissolved in 100 µL of 0.1 M TEAA in MeCN/H_2O (5/1). The CuAAC reactions were carried out in a commercial chemical flow reactor equipped with a 2 mL copper reactor (easy-Scholar from Vapourtec). As depicted in Figure S1, the reaction mixture was fed through the copper reactor (2 mL total volume) at a flow rate of 9 mL/min at rt (25 °C). The reaction mixture was collected following one cycle through the reactor and lyophilized. The purity of the triazole products **26a-c** was assessed using a Shimadzu Prominence HPLC using an analytical column (Phenomenex Clarity 5 µM Oligo-RP, 250 x 4.6 mm) at a flow rate of 1 mL/min, gradient 10–50% 0.1 M TEAA in MeCN in 35 min. Yields were calculated using the HPLC integrals ratio relative to starting materials.

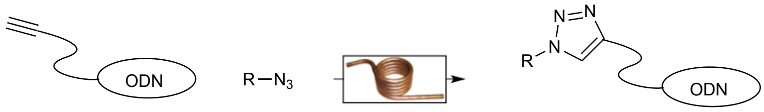

Product	Sequence	Yield (%)[a]
ODN4 (**26a**)	5'-**X**$_t$TTT TTT TTT TTT-3'	Quant
ODN5 (**26b**)	5'-**X**$_t$TTT TTT GTT TTT-3'	Quant
ODN6 (**26c**)	5'-**X**$_t$GCA TTG ACT GCT-3'	91

Supplementary Table 2. Experimental data for the synthesis of **26a-c**. [a] RP-HPLC yields.

X_t =

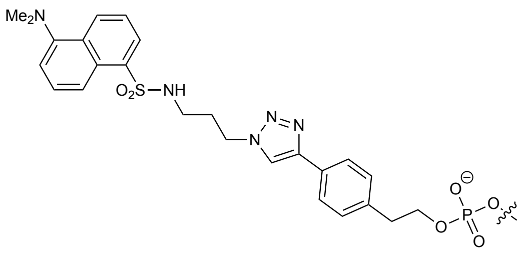

<u>General Procedure D for solid –phase peptide synthesis of **P1-5**</u>

Solid–phase synthesis was performed on Rink amide resin (0.6–0.7 mmol/g, 100–200 mesh) (NovaBiochem) using a Protein Technologies Tribute automated synthesizer. Syntheses were performed from the C-terminus to the N-terminus using Fmoc-protected amino acids. The resin was swelled for 20 min in DCM. Fmoc deprotection was achieved using 20% piperidine in DMF (5 min). Each amino acid (5.0 equiv) was activated with HATU (4.5 equiv) and DIPEA (0.5 M in DMF) for 2 min (10 min for Arginine and Cysteine based amino acids). The solution was then added to the resin and shaken for 20 min (2 h for Arginine and Cysteine based amino acids) then washed with further DMF. Double couplings were used for Arginine and Cysteine based amino acids. Capping of **P1-5** was performed using a solution of 15% acetic anhydride in DMF. Deprotection, coupling, washing, and capping procedure were repeated until the final amino acid had been coupled to the peptide chain. The peptide was then cleaved from the resin using a solution of TFA/phenol/H_2O/TIPS (90/5/2.5/2.5) for 2 h, precipitated by addition of cold Et_2O and centrifuged at 7000 rpm for 25 min (three times). Peptides **P1-5** were then purified by RP-HPLC with a Dionex UltiMate 3000 HPLC using a preparative column (Phenomenex C18, 150 × 21.2 mm) at a flow rate of 9 mL/min, gradient 5–100% MeCN/0.1% TFA in 95 min. Purity of resultant products was assessed by analytical RP-HPLC (Phenomenex C18, 250 × 4.6 mm) at a flow rate of 1 mL/min, gradient 5–60% MeCN/0.1% TFA in 35 min.

Product	Sequence	Yield (%)[a]
P1	Ac-LRKLRKRLLR**X**	36
P2	Ac-CLRKLRKRLLR**X**	27
P3	Ac-MLRKLRKRLLR**X**	22
P4	Ac-HLRKLRKRLLR**X**	39
P5	Ac-YLRKLRKRLLR**X**	33

Supplementary Table 3. List of synthesized peptides **P1-5,** sequence and yields. [a] Isolated yields after RP-HPLC purification.

X = 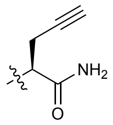

General Procedure E for CuAAC Reactions with Peptides:

Alkyne **P1-5** (1 equiv), azide (2 equiv), and 3-methylbenzo[*d*]thiazol-3-ium **29** (2 equiv) were dissolved in 100 μL of MeCN/H_2O (5/1). The CuAAC reactions were carried out in a commercial chemical flow reactor equipped with a 2 mL copper reactor (easy-Scholar from Vapourtec). The reaction mixture was fed through the copper reactor (2 mL total volume) at a flow rate of 1 mL/min at rt (25 °C). The reaction mixture was collected after four cycles through the reactor, lyophilized, and purified by reverse phase HPLC with a Dionex UltiMate 3000 HPLC using a preparative column (Phenomenex C18, 150 × 21.2 mm) at a flow rate of 9 mL/min, gradient 5–100% MeCN/0.1% TFA in 95 min. Purity of resultant products was assessed by analytical RP-HPLC (Phenomenex C18, 250 × 4.6 mm) at a flow rate of 1 mL/min, gradient 5–60% MeCN/0.1% TFA in 35 min.

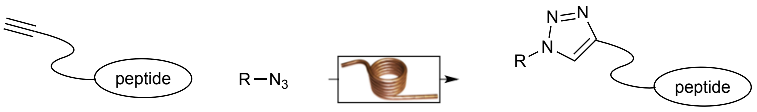

Product	Sequence	Isolated Yield (%)[a]
P6 (**27a**)	Ac-LRKLRKRLLR**Y**	75
P7 (**27b**)	Ac-CLRKLRKRLLR**Y**	48
P8 (**27c**)	Ac-MLRKLRKRLLR**Y**	57
P9 (**27d**)	Ac-HLRKLRKRLLR**Y**	69
P10 (**27e**)	Ac-YLRKLRKRLLR**Y**	65

Supplementary Table 4. Experimental data for the synthesis of **27a-e**. [a] Isolated yields after RP-HPLC purification.

Y = 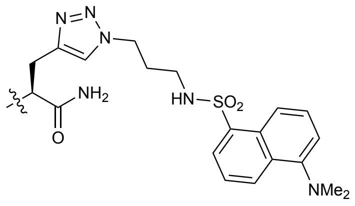

General Procedure F for the synthesis of triazoles in batch using Cu powder

Alkyne **1a-c** (0.2 mmol) and azide **2** (0.2 mmol) were dissolved in 10 mL of MeCN/H_2O (5/1). Cu powder (5 mol %) was then added and the reaction stirred at rt for 24 h. DCM (20 mL) was then added, washed with aq. EDTA solution (10 mg/mL, 1 x 20 mL), brine (2 × 20 mL), dried over Na_2SO_4, and concentrated under reduced pressure. The resulting residue was purified by flash chromatography (silica gel, 3/7 PE/EtOAc). Formation of the expected triazole was only observed with alkyne **1a** (yield 60%), only recovery of starting materials was observed for alkyne **1b** and **1c**.

General Procedure G for the synthesis of triazoles in batch using CuO

Alkyne **1a-c** (0.2 mmol) and azide **2** (0.2 mmol) were dissolved in 10 mL of MeCN/H_2O (5/1). CuO (5 mol %) was then added and the reaction stirred at rt for 24 h. DCM (20 mL) was then added, washed with aq. EDTA solution (10 mg/mL, 1 x 20 mL), brine (2 × 20 mL), dried over Na_2SO_4, and concentrated under reduced pressure. The resulting residue was purified by flash chromatography (silica gel, 3/7 PE/EtOAc). Formation of the expected triazole was only observed with alkyne **1a** (yield 58%), only recovery of starting materials was observed for alkyne **1b** and **1c**.

General Procedure H for the synthesis of triazoles using a PFA flow reactor and Cu powder

Alkyne **1a-c** (0.2 mmol) and azide **2** (0.2 mmol) were dissolved in 10 mL of MeCN/H_2O (5/1) before adding Cu powder (5 mol %). The CuAAC reactions were carried out in a commercial chemical flow reactor equipped with a 10 mL PFA reactor (easy-Scholar from Vapourtec). The reaction mixture was flowed through a PFA tube (diameter = 1 mm, volume = 10 mL, surface area = 400 cm^2) at a flow rate of 1 mL/min at rt (25 °C, t_R = 10 min). The reaction mixture was then collected and concentrated *in vacuo*. Only recovery of SM was observed for all alkynes **1a-c**.

General Procedure I for the synthesis of triazoles using a PFA flow reactor and CuO

Alkyne **1a-c** (0.2 mmol) and azide **2** (0.2 mmol) were dissolved in 10 mL of MeCN/H_2O (5/1) before adding CuO (5 mol %). The CuAAC reactions were carried out in a commercial chemical flow reactor equipped with a 10 mL PFA reactor (easy-Scholar from Vapourtec). The reaction mixture was flowed through a PFA tube (diameter = 1 mm, volume = 10 mL, surface area = 400 cm^2) at a flow rate of 1 mL/min at rt (25 °C, t_R = 10 min). The reaction

mixture was then collected and concentrated *in vacuo*. Only recovery of SM was observed for all alkynes **1a-c**.

Supplementary Discussion

Effect of H_2O on triazole formation

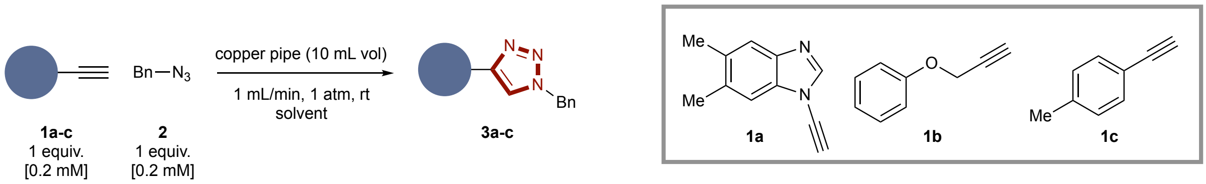

Ratio MeCN/H_2O	Isolated Yield of 3a (%)	Isolated Yield of 3b (%)	Isolated Yield of 3c (%)
100/0	0	0	0
99/1	46	78	85
98/1	52	91	94
95/1	56	94	100
90/1	57	97	100
80/1	58	93	100
60/1	68	91	100
40/1	75	95	96
5/1	100	100	100
1/1	100	100	100

Supplementary Table 5. Solvent ratio *vs.* yields of products **3a-c**. Prepared following General Procedure A.

Investigation of triazole product formation as a function of flow rate

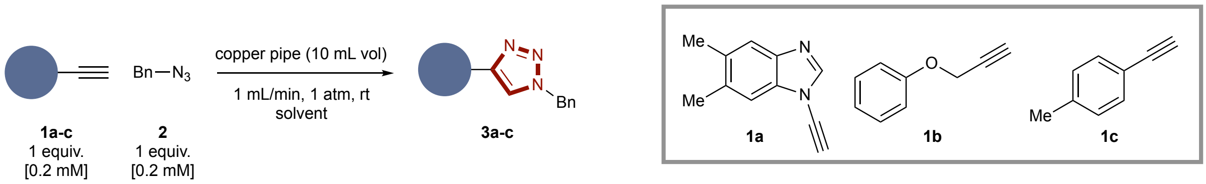

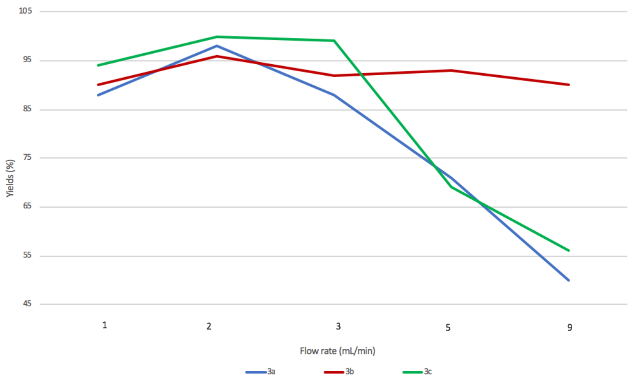

Supplementary Figure 2. Investigation of yield of triazole formation as a function of flow rate using the non-chelating azide **2**. Prepared following General Procedure A. Isolated yields.

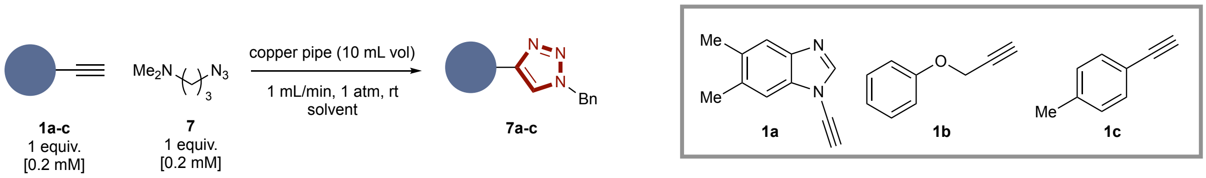

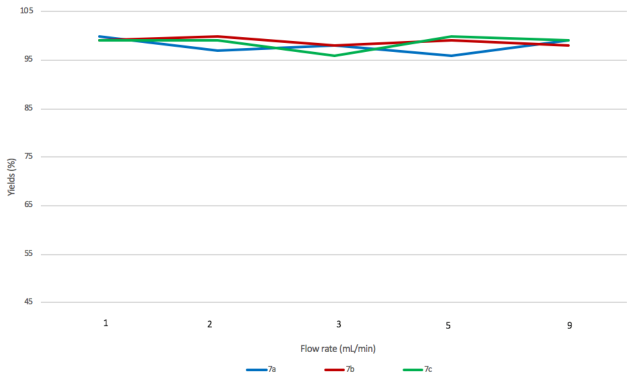

Supplementary Figure 3. Investigation of yield of triazole formation as a function of flow rate using the chelating azide **7**. Prepared following General Procedure A. Isolated yields.

Investigation of triazole product formation as a function of Cu leaching

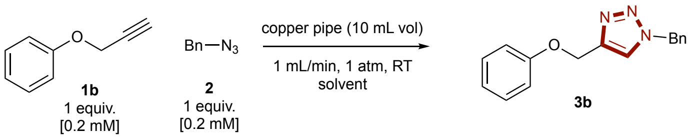

Solvent	Yield (%)	[Cu] (ppm)
MeCN	0	6.92
MeCN/H_2O (5/1)	90	40.9
MeOH	0	7.69
MeOH/H_2O (5/1)	13	1.84
DMF	0	8.17
DMF/H_2O (5/1)	30	9.24
DCM	7	1.2

Supplementary Table 6. Solvent *vs.* yield of **3b** as a function of Cu leaching. Prepared following General Procedure A. Isolated yields.

Optimization of flow CuAAC reaction using alkyne-modified oligodeoxyribonucleotide (ODN)

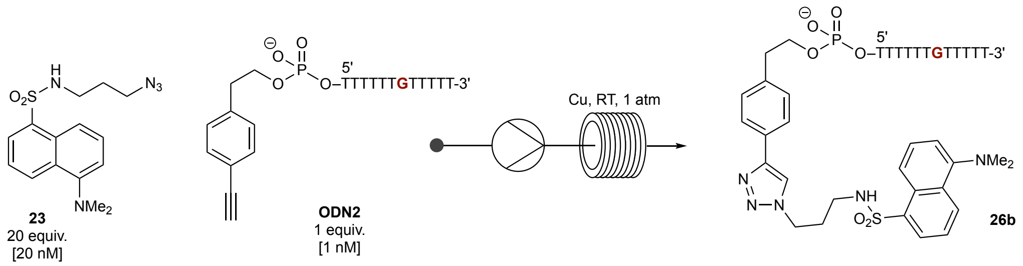

Solvent	Flow Rate (mL/min)	Yields (%)[a]
H_2O	0.2 (t_R = 10 min)	0
H_2O	1 (t_R = 2 min)	7
MeCN/ H_2O (5/1)	0.2 (t_R = 10 min)	4
MeCN/ H_2O (5/1)	1 (t_R = 2 min)	21
MeCN/ H_2O (5/1)	2 (t_R = 1 min)	59
MeCN/ H_2O (5/1)	2 (3 cycles, t_R = 3 min)	36
MeCN/ H_2O (5/1) + 0.1% TEAA	2 (t_R = 1 min)	100

Supplementary Table 7. Optimization of flow CuAAC reaction using ODN_2. Prepared following General Procedure C. [a] Yields determined by RP-HPLC peak integration relative to SM.

Optimization of flow CuAAC reaction using alkyne-modified peptides

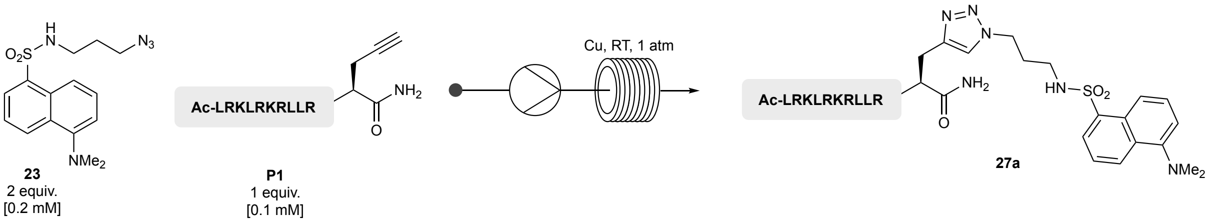

Flow Rate (mL/min)	Conversion (%)
0.2 (t_R = 10 min)	69
0.1 (t_R = 20 min)	79
1 (t_R = 2 min)	68
1 (2 cycles, t_R = 4 min)	96
1 (3 cycles, t_R = 6 min)	97
1 (4 cycles, t_R = 8 min)	100
2 (t_R = 1 min)	47
2 (2 cycles, t_R = 2 min)	87
2 (3 cycles, t_R = 3 min)	84
2 (4 cycles, t_R = 4 min)	92

Supplementary Table 8. Optimization of flow CuAAC reactions using peptide **P1**. Prepared following General Procedure E. Conversion determined by RP-HPLC peak integration relative to SM.

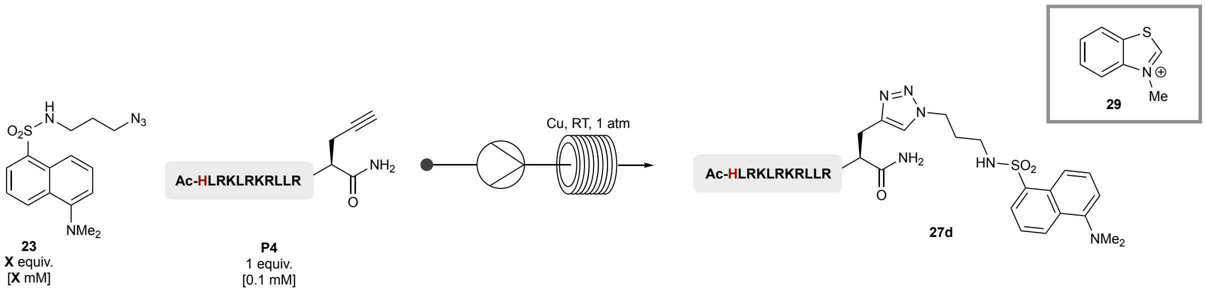

Azide 23	Additive 29	Solvent	Flow Rate (mL/min)	Conversion (%)
1 equiv.	X	MeCN/ H_2O (5/1)	0.2 (t_R = 10 min)	44
2 equiv.	X	MeCN/ H_2O (5/1)	0.2 (t_R = 10 min)	23
1 equiv.	X	MeCN/ H_2O (5/1) + 0.1% TFA	0.2 (t_R = 10 min)	35
2 equiv.	X	MeCN/ H_2O (5/1)	1 (4 cycles, t_R = 8 min)	77
2 equiv.	2 equiv.	MeCN/ H_2O (5/1)	1 (4 cycles, t_R = 8 min)	90

Supplementary Table 9. Optimization of flow CuAAC reactions using peptide **P4**. Prepared following General Procedure E. Conversion determined by RP-HPLC peak integration relative to SM.

ICP-MS results

The instrument was operated in spectrum acquisition mode and three replicate runs per sample were obtained. The masses analysed for Cu were ^{63}Cu and ^{65}Cu. ^{103}Rh was used as an internal standard and added at a concentration of 20 µg/L. Each mass was analysed in full quantitation mode (three points per unit mass) and analysed in standard 'nogas' mode. A series of standards were prepared using single element 1000 mg/L Cu (Fischer Scientific) diluted with distilled water and MeCN to match the samples to give a range from 1-1000 µg/L. An external reference standard CRM SLRS-4 (NRCC, Canada) was diluted 2-fold to check for accuracy of the standard graph.

The samples were taken directly after a single pass through the copper flow reactor (1 mL/min) and diluted to reduce the concentration of MeCN present in the samples to 1% *v/v*. Where samples didn't have any MeCN these were spiked with MeCN to keep all sample/standards in the same matrix to reduce any changes in ionization, *etc*.

Parameters for No gas mode:

Ion Lenses:

Extract1: 0 V

Extract 2: -131 V

Omega Bias-ce: -20 V

Omega Lens-ce: 0 V

Cell Entrance: -30 V

QP focus: 3 V

Cell Exit: -34 V

Quadrupole Parameters:

OctP RF: 180 V

OctP Bias: -6 V

QP Bias: -3 V

Ratio MeCN/H_2O	[Cu] (ppm) of Used Reactor	[Cu] (ppm) of New Reactor
100/0	0.353	0.790
99/1	0.206	0.240
98/1	0.510	0.387
95/1	1.69	1.04
90/1	2.88	2.23
80/1	2.13	1.65
60/1	2.98	2.07
40/1	7.05	3.79
5/1	12.3	6.30
1/1	19.2	8.06
1/5	10.6	4.50
1/40	2.47	3.01
0/100	0.32	1.16

Supplementary Table 10. ICP-MS analysis of different solvent eluents from a used reactor (~ 300 reactions) *vs.* a new one (0 reactions).

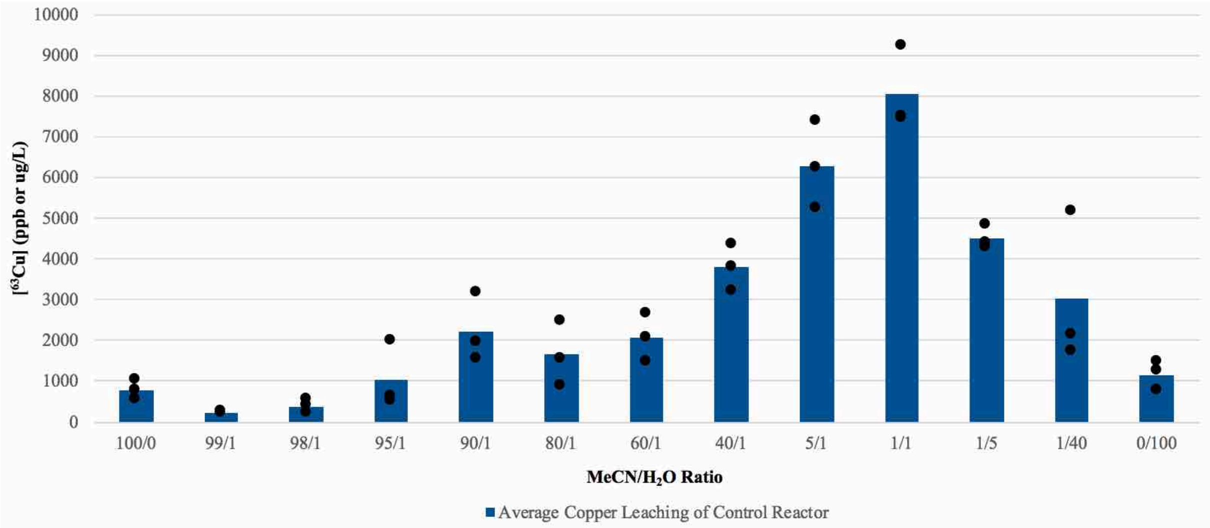

Supplementary Figure 4. Dot plot for ICP-MS analysis from control reactor.

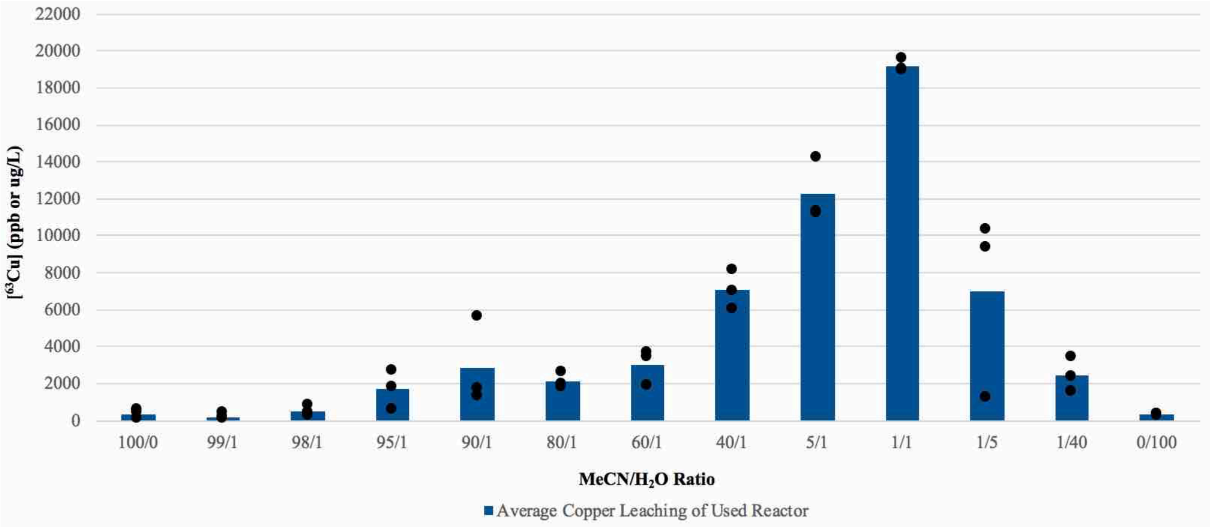

Supplementary Figure 5. Dot plot for ICP-MS analysis from used reactor.

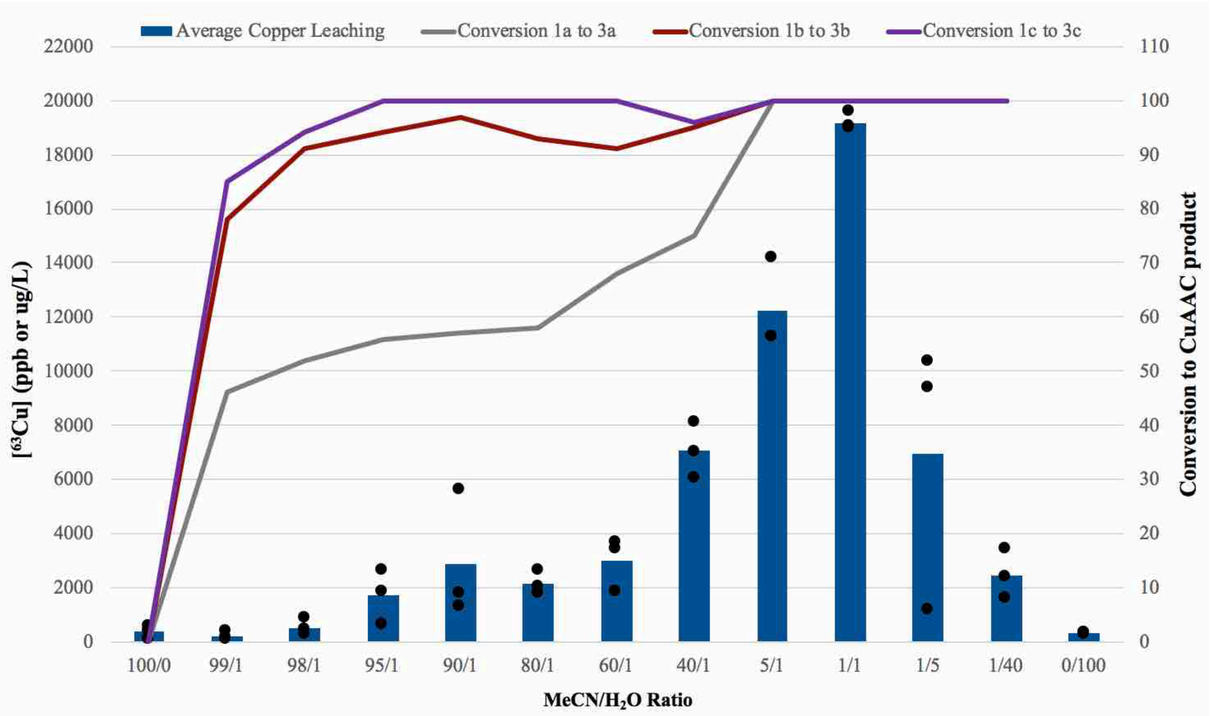

Supplementary Figure 6. Overlaid dot plots for ICP-MS analysis as a function of conversion and solvent composition.

Product	[Cu] (ppm)
**25**	6.21
ODN6 (**26c**)	8.96
P9 (**27d**)	7.13
**28**	7.42

Supplementary Table 11. ICP-MS analysis of biomolecules after CuAAC reactions in flow.

Spectroscopic characterization of small molecules

(1*R*,3*S*,8*R*,9*S*,13*R*,14*S*)-1,13-Dimethyl-17-((*R*)-6-methylheptan-2-yl)-2,3,4,7,8,9,10,11,12,13,14,15,16,17-tetradecahydro-1*H*-cyclopenta[*a*]phenanthren-3-yl (3-azidopropyl) Carbamate (**S1**)

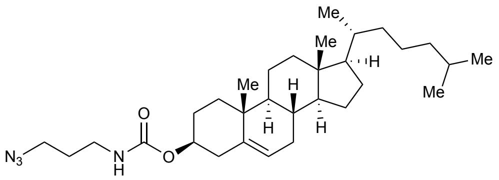

(1*R*,3*S*,8*R*,9*S*,13*R*,14*S*)-1,13-Dimethyl-17-((*R*)-6-methylheptan-2-yl)-2,3,4,7,8,9,10,11,12,13,14,15,16,17-tetradecahydro-1*H*-cyclopenta[*a*]phenanthren-3-yl carbonochloridate (1.0 g, 2.2 mmol, 1 equiv) and NEt_3 (0.9 mL, 6.7 mmol, 3 equiv) were dissolved in DCM (5 mL). 3-

Azidopropan-1-amine (0.27 g, 2.7 mmol, 1.2 equiv) was added dropwise over 15 min and the reaction stirred at rt for 16 h. DCM (20 mL) was then added, washed with brine (3 × 20 mL), dried over Na_2SO_4, and concentrated under reduced pressure. The resulting residue was purified by flash chromatography (silica gel, 9/1 PE/EtOAc) to provide the desired product as a white solid (0.82 g, 72%).

^{1}H NMR (400 MHz, $CDCl_3$): δ 5.35–5.34 (m, 1H), 4.89–4.86 (m, 1H), 4.50–4.42 (m, 1H), 3.34 (t, *J* = 6.7 Hz, 2H), 3.23 (q, *J* = 6.3 Hz, 2H), 2.35–2.21 (m, 2H), 1.99–1.73 (m, 8H), 1.56–1.29 (m, 11H), 1.18–1.02 (m, 8H), 0.96–0.91 (m, 3H), 0.89 (d, *J* = 6.5 Hz, 3H), 0.83 (dd, *J* = 1.7, 6.6 Hz, 6H), 0.65 (s, 3H).

^{13}C NMR (100 MHz, $CDCl_3$): δ 156.3, 139.8, 122.6, 74.5, 56.8, 56.2, 50.1, 49.2, 42.4, 39.8, 39.6, 38.6, 38.4, 37.1, 36.6, 36.3, 35.9, 32.0, 29.3, 28.3, 28.2, 28.1, 24.4, 23.9, 22.9, 22.6, 21.1, 19.4, 18.8, 11.9.

IR ν_{max} (neat): 3305, 2934, 2865, 2095, 1686, 1537, 1268, 1255 cm^{-1}.

HRMS (ESI, $C_{31}H_{53}N_4O_2$, +ve mode): *m/z* $[M+H]^+$calcd. 513.4163, found 513.4156.

1-(1-Benzyl-1*H*-1,2,3-triazol-4-yl)-5,6-dimethyl-1*H*-benzo[*d*]imidazole (**3a**)[3]

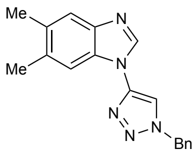

Prepared using General Procedure A. White amorphous solid (53 mg, 88%). Purification on silica gel using 7/3 PE/EtOAc.

^{1}H NMR (400 MHz, $CDCl_3$): δ 8.20 (s, 1H), 7.67 (s, 1H), 7.58 (s, 1H), 7.45–7.39 (m, 4H), 7.37–7.34 (m, 2H), 5.62 (s, 2H), 2.37 (s, 6H).

^{13}C NMR (100 MHz, $CDCl_3$): δ 143.2, 142.4, 140.4, 134.0, 133.6, 132.3, 131.1, 129.5, 129.3, 128.3, 120.7, 113.5, 111.3, 55.2, 20.7, 20.3.

1-Benzyl-4-(phenoxymethyl)-1*H*-1,2,3-triazole (**3b**)[15]

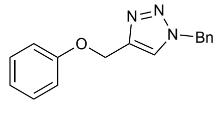

Prepared using General Procedure A. Colorless oil (48 mg, 90%). Purification on silica gel using 9/1 PE/EtOAc.

^{1}H NMR (400 MHz, $CDCl_3$): δ 7.52 (s, 1H), 7.38–7.35 (m, 3H), 7.28–7.26 (m, 4H), 6.98–6.94 (m, 3H), 5.53 (s, 2H), 5.19 (s, 2H).

^{13}C NMR (100 MHz, $CDCl_3$): δ 158.3, 144.9, 134.6, 129.6, 129.3, 128.9, 128.3, 122.7, 121.4, 114.9, 62.2, 54.4.

1-Benzyl-4-(*p*-tolyl)-1*H*-1,2,3-triazole (**3c**)[16]

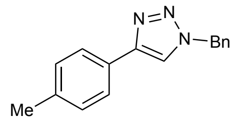

Prepared using General Procedure A. Colorless oil (47 mg, 94%). Purification on silica gel using 9/1 PE/EtOAc.

^{1}H NMR (400 MHz, $CDCl_3$): δ 7.70–7.69 (m, 1H), 7.68–7.67 (s, 1H), 7.63 (s, 1H), 7.38−7.36 (m, 3H), 7.31−7.28 (m, 2H), 7.21−7.19 (m, 2H), 5.54 (s, 2H), 2.36 (s, 3H).

^{13}C NMR (100 MHz, $CDCl_3$): δ 148.4, 138.1, 134.9, 129.6, 129.2, 128.8, 128.1, 127.8, 125.7, 119.3, 54.2, 21.3.

5,6-Dimethyl-1-(1-(pyridin-2-ylmethyl)-1*H*-1,2,3-triazol-4-yl)-1*H*-benzo[*d*]imidazole (**4a**)[3]

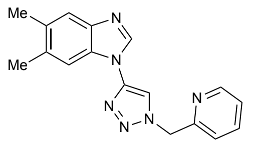

Prepared using General Procedure A. Pale yellow oil (48 mg, 79%). Purification on silica gel using 1/9 PE/EtOAc + 2% NEt_3.

^{1}H NMR (400 MHz, $CDCl_3$): δ 8.60 (d, J = 4.3 Hz, 1H), 8.26 (br. s, 1H), 8.03 (s, 1H), 7.71 (t, J = 7.6 Hz, 1H), 7.57 (s, 1H), 7.45 (s, 1H), 7.34–7.26 (m, 2H), 5.71 (s, 2H), 2.37–2.35 (m, 6H).

^{13}C NMR (100 MHz, $CDCl_3$): δ 153.6, 150.1, 143.0, 142.3, 140.4, 137.6, 133.6, 132.2, 131.0, 123.8, 122.8, 120.6, 114.3, 111.3, 56.4, 20.6, 20.3.

2-((4-(Phenoxymethyl)-1*H*-1,2,3-triazol-1-yl)methyl)pyridine (**4b**)[17]

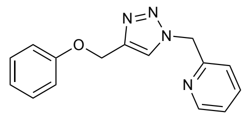

Prepared using General Procedure A. Pale yellow oil (46 mg, 87%). Purification on silica gel using 4/6 PE/EtOAc.

^{1}H NMR (400 MHz, $CDCl_3$): δ 8.59 (br. s, 1H), 7.78 (s, 1H), 7.67 (dt, J = 1.7, 7.7 Hz, 1H), 7.30–7.24 (m, 3H), 7.18 (d, J = 7.7 Hz, 1H), 6.98–6.40 (m, 3H), 5.65 (s, 2H), 5.21 (s, 2H).

^{13}**C NMR** (100 MHz, $CDCl_3$): δ 158.3, 154.4, 149.9, 144.8, 137.5, 129.6, 123.6, 123.4, 122.6, 121.4, 114.9, 62.1, 55.8.

2-((4-(*p*-Tolyl)-1*H*-1,2,3-triazol-1-yl)methyl)pyridine (**4c**)[15]

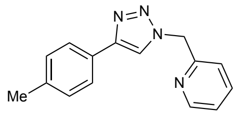

Prepared using General Procedure A. Pale yellow oil (41 mg, 82%). Purification on silica gel using 9/1 PE/EtOAc.

^{1}**H NMR** (400 MHz, $CDCl_3$): δ 8.61 (br. s, 1H), 7.89 (s, 1H), 7.72–7.67 (m, 3H), 7.28–7.25 (m, 1H), 7.23–7.21 (m, 3H), 5.69 (s, 2H), 2.36 (s, 3H).

^{13}**C NMR** (100 MHz, $CDCl_3$): δ 154.7, 149.8, 148.4, 138.1, 137.5, 129.6, 127.8, 125.7, 123.6, 122.6, 120.0, 55.8, 21.4.

5,6-Dimethyl-1-(1-(pyridin-4-ylmethyl)-1*H*-1,2,3-triazol-4-yl)-1*H*-benzo[*d*]imidazole (**5a**)

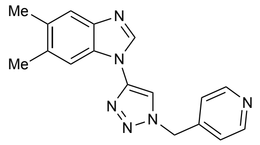

Prepared using General Procedure A. Yellow oil (38 mg, 63%). Purification on silica gel using 1/9 PE/EtOAc + 2% NEt_3.

^{1}**H NMR** (400 MHz, $CDCl_3$): δ 8.63 (br. s, 2H), 8.26 (br. s, 1H), 7.88 (s, 1H), 7.55 (s, 1H), 7.45 (s, 1H), 7.18 (br. s, 2H), 5.62 (s, 2H), 2.34 (s, 6H).

^{13}**C NMR** (100 MHz, $CDCl_3$): δ 150.8, 143.5, 143.0, 142.2, 140.3, 133.9, 132.6, 130.9, 122.3, 120.7, 113.8, 111.4, 53.7, 20.7, 20.3.

IR ν_{max} (neat): 3129, 3073, 3030, 2967, 2935, 1656, 1595, 1498, 1465, 1416, 1288, 1203, 1056 cm^{-1}.

HRMS (ESI, $C_{17}H_{17}N_6$, +ve mode): *m/z* $[M+H]^+$ calcd. 305.1509, found 305.1510.

4-((4-(Phenoxymethyl)-1*H*-1,2,3-triazol-1-yl)methyl)pyridine (**5b**)

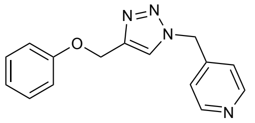

Prepared using General Procedure A. Yellow oil (37 mg, 70%). Purification on silica gel using 4/6 PE/EtOAc.

^{1}**H NMR** (400 MHz, $CDCl_3$): δ 8.62 (br. s, 2H), 7.30–7.26 (m, 3H), 7.10 (d, J = 4.8 Hz, 2H), 6.99–6.96 (m, 3H), 5.55 (s, 2H), 5.23 (s, 2H).

^{13}**C NMR** (100 MHz, $CDCl_3$): δ 158.2, 150.7, 145.4, 143.5, 129.7, 123.0, 122.3, 121.5, 114.9, 62.1, 52.9.

IR ν$_{max}$ (neat): 3138, 3093, 3060, 3032, 2932, 2876, 1686, 1600, 1493, 1416, 1230, 1175, 1050, 1033 cm^{-1}.

HRMS (ESI, $C_{15}H_{15}N_4O$, +ve mode): *m/z* $[M+H]^+$ calcd. 267.1240, found 267.1241.

4-((4-(*p*-Tolyl)-1*H*-1,2,3-triazol-1-yl)methyl)pyridine (**5c**)[18]

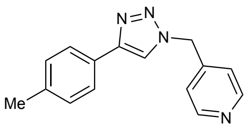

Prepared using General Procedure A. Yellow oil (40 mg, 80%). Purification on silica gel using 9/1 PEt/EtOAc.

^{1}**H NMR** (400 MHz, $CDCl_3$): δ 8.62 (br. s, 2H), 7.71–7.69 (m, 3H), 7.22 (d, J = 7.9 Hz, 2H), 7.14 (d, J = 5.1 Hz, 2H), 5.59 (s, 2H), 2.37 (s, 3H).

^{13}**C NMR** (100 MHz, $CDCl_3$): δ 150.6, 148.9, 143.9, 138.5, 129.7, 127.5, 125.8, 122.2, 119.6, 52.9, 21.4.

5,6-Dimethyl-1-(1-((phenylthio)methyl)-1*H*-1,2,3-triazol-4-yl)-1*H*-benzo[*d*]imidazole (**6a**)

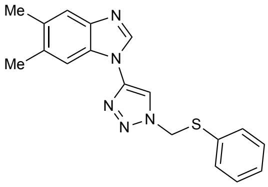

Prepared using General Procedure A. Yellow oil (56 mg, 84%). Purification on silica gel using 3/7 PE/EtOAc.

^{1}**H NMR** (400 MHz, $CDCl_3$): δ 8.24 (br. s, 1H), 7.73 (s, 1H), 7.58 (s, 1H), 7.39–7.38 (m, 2H), 7.34–7.31 (m, 4H), 5.69 (s, 2H), 2.36 (s, 6H).

^{13}**C NMR** (100 MHz, $CDCl_3$): δ 133.8, 132.7, 132.3, 131.5, 129.9, 129.3, 121.0, 113.0, 111.5, 55.1, 20.7, 20.4. Four signals not observed/coincident.

IR ν$_{max}$ (neat): 3114, 2969, 2937, 2921, 1686, 1593, 1496, 1467, 1441, 1390, 1283, 1217, 1046, 1026 cm^{-1}.

HRMS (ESI, $C_{18}H_{18}N_5S$, +ve mode): *m/z* $[M+H]^+$ calcd. 336.1277, found 336.1277.

4-(Phenoxymethyl)-1-((phenylthio)methyl)-1*H*-1,2,3-triazole (**6b**)

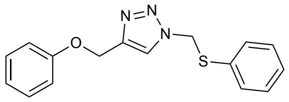

Prepared using General Procedure A. Pale yellow oil (49 mg, 82%). Purification on silica gel using 1/1 PE/EtOAc.

^{1}H NMR (400 MHz, $CDCl_3$): δ 7.60 (s, 1H), 7.31–7.26 (m, 7H), 6.99–6.95 (m, 3H), 5.61 (s, 2H), 5.19 (s, 2H).

^{13}C NMR (100 MHz, $CDCl_3$): δ 158.2, 145.1, 132.5, 131.8, 129.6, 128.9, 122.3, 121.4, 114.9, 114.5, 62.1, 54.1.

IR ν_{max} (neat): 3138, 3156, 2947, 2872, 1599, 1493, 1441, 1229, 1175, 1046 cm^{-1}.

HRMS (ESI, $C_{16}H_{16}N_3OS$, +ve mode): *m/z* $[M+H]^+$ calcd. 298.1009, found 298.1007.

1-((Phenylthio)methyl)-4-(*p*-tolyl)-1*H*-1,2,3-triazole (**6c**)

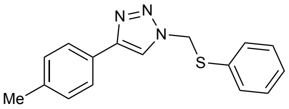

Prepared using General Procedure A. Pale yellow oil (44 mg, 79%). Purification on silica gel using 8/2 PE/EtOAc.

^{1}H NMR (400 MHz, $CDCl_3$): δ 7.73 (br s., 1H), 7.69 (s, 1H), 7.67 (s, 1H), 7.36–7.34 (m, 2H), 7.29–7.28 (m, 3H), 7.22 (s, 1H), 7.20 (s, 1H), 5.62 (s, 2H), 2.37 (s, 3H).

^{13}C NMR (100 MHz, $CDCl_3$): δ 138.1, 132.2, 132.0, 129.5, 129.4, 128.7, 127.6, 125.6, 118.8, 53.8, 21.3. One signal not observed/coincident.

IR ν_{max} (neat): 3093, 3023, 2915, 2859, 1502, 1448, 1396, 1349, 1223, 1193 cm^{-1}.

HRMS (ESI, $C_{16}H_{16}N_3S$, +ve mode): *m/z* $[M+H]^+$ calcd. 282.1059, found 282.1056.

3-(4-(5,6-Dimethyl-1*H*-benzo[*d*]imidazol-1-yl)-1*H*-1,2,3-triazol-1-yl)-*N*,*N*-dimethylprop--an-1-amine (**7a**)

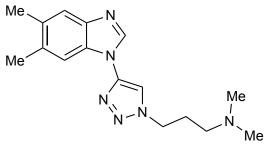

Prepared using General Procedure A. Pale yellow oil (60 mg, Quant). No purification needed.

^{1}H NMR (400 MHz, $CDCl_3$): δ 8.21 (br. s, 1H), 7.84 (s, 1H), 7.61 (s, 1H), 7.46 (s, 1H), 4.53 (t, *J* = 6.8 Hz, 2H), 2.38 (d, *J* = 2.2 Hz, 6H), 2.32 (t, *J* = 6.6 Hz, 2H), 2.24 (s, 6H), 2.17–2.10 (m, 2H).

^{13}C NMR (100 MHz, $CDCl_3$): δ 142.6, 133.6, 132.2, 120.8, 114.3, 111.3, 55.7, 49.0, 45.4, 28.0, 20.6, 20.3. Three signals not observed/coincident.

IR ν_{max} (neat): 3103, 2941, 2816, 2766, 1625, 1587, 1495, 1461, 1377, 1331, 1286, 1219, 1089, 1041 cm^{-1}.

HRMS (ESI, $C_{16}H_{23}N_6$, +ve mode): *m/z* $[M+H]^+$ calcd. 299.1979, found 299.1977.

N,*N*-Dimethyl-3-(4-(phenoxymethyl)-1*H*-1,2,3-triazol-1-yl)propan-1-amine (**7b**)

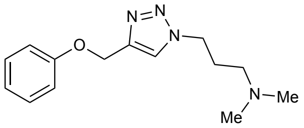

Prepared using General Procedure A. Pale yellow oil (51 mg, 99%). No purification needed.

^{1}H NMR (400 MHz, $CDCl_3$): δ 7.61 (s, 1H), 7.29–7.25 (m, 2H), 6.98–6.93 (m, 3H), 5.21 (s, 2H), 4.41 (t, *J* = 6.9 Hz, 2H), 2.22 (t, *J* = 6.9 Hz 2H), 2.18 (s, 6H), 2.04 (app. quint, *J* = 6.9 Hz, 2H).

^{13}C NMR (100 MHz, $CDCl_3$): δ 158.3, 144.2, 129.6, 123.1, 121.3, 114.9, 62.2, 55.9, 48.2, 45.4, 28.2.

IR ν_{max} (neat): 3138, 2943, 2861, 2816, 2766, 1671, 1587, 1495, 1240, 1175, 1030 cm^{-1}.

HRMS (ESI, $C_{14}H_{21}N_4O$, +ve mode): *m/z* $[M+H]^+$ calcd. 261.1710, found 261.1709.

N,*N*-Dimethyl-3-(4-(*p*-tolyl)-1*H*-1,2,3-triazol-1-yl)propan-1-amine (**7c**)

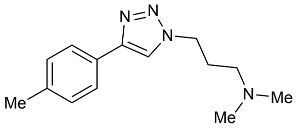

Prepared using General Procedure A. Yellow oil (48 mg, 99%). No purification needed.

^{1}H NMR (400 MHz, $CDCl_3$): δ 7.73 (s, 1H), 7.72–7.73 (m, 1H), 7.71–7.70 (m, 1H), 7.24 (s, 1H), 7.22 (s, 1H), 4.46 (t, *J* = 6.9 Hz, 2H), 2.37 (s, 3H), 2.28 (t, *J* = 6.8 Hz, 2H), 2.22 (s, 6H), 2.08 (app. quint, *J* = 6.9 Hz, 2H).

^{13}C NMR (100 MHz, $CDCl_3$): δ 147.8, 138.0, 129.6, 128.0, 125.7, 119.8, 55.9, 48.1, 45.3, 28.2, 21.4.

IR ν_{max} (neat): 3134, 3108, 2943, 2859, 2816, 2764, 1671, 1500, 1461, 1223, 1043 cm^{-1}.

HRMS (ESI, $C_{14}H_{21}N_4$, +ve mode): *m/z* $[M+H]^+$ calcd. 245.1761, found 245.1760.

2-(4-(5,6-Dimethyl-1*H*-benzo[*d*]imidazol-1-yl)-1*H*-1,2,3-triazol-1-yl)-*N*-(4-fluorobenzyl)

acetamide (**8a**)

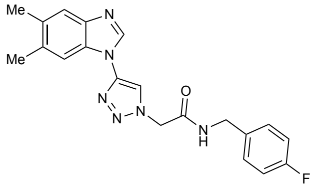

Prepared using General Procedure A. Yellow oil (70 mg, 92%). Purification on silica gel using 9/1 DCM/MeOH + 2% NEt_3.

^{1}H NMR (400 MHz, $CDCl_3$): δ 8.26 (br. s, 1H), 8.02 (s, 1H), 7.54–7.57 (m, 1H), 7.43 (s, 1H), 7.21–7.27 (m, 2H), 6.97–7.02 (m, 2H), 6.83 (br. s, 1H), 5.18 (s, 2H), 4.45 (d, *J* = 5.3 Hz, 2H), 2.38 (s, 6H).

^{19}F NMR (376 MHz, DMSO-d_6): δ 115.8.

^{13}C NMR (100 MHz, $CDCl_3$): δ 165.1, 162.3, 160.3, 141.8, 134.9, 134.8, 132.7, 131.3, 129.5, 129.4, 120.0, 117.2, 115.2 (d, *J* = 20.9 Hz), 111.8, 52.4, 41.7, 20.1, 19.8.

IR ν_{max} (neat): 3426, 2251, 2127, 1666, 1511, 1054, 1026, 1007 cm^{-1}.

HRMS (ESI, $C_{20}H_{20}FN_6O$, +ve mode): *m/z* $[M+H]^+$ calcd. 379.1677, found 379.1677.

2-(4-Fluorophenyl)-*N*-((4-(phenoxymethyl)-1*H*-1,2,3-triazol-1-yl)methyl)acetamide (**8b**)

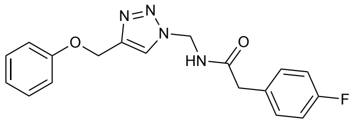

Prepared using General Procedure A. Yellow oil (63 mg, 93%). Purification on silica gel using 9/1 DCM/MeOH + 2% NEt_3.

^{1}H NMR (400 MHz, $CDCl_3$): δ 7.79 (br. s, 1H), 7.33–7.28 (m, 2H), 7.19–7.16 (m, 2H), 6.97–7.01 (m, 5H), 5.22 (s, 2H), 5.08 (s, 2H), 4.69 (d, *J* = 2.4 Hz, 1H), 4.39 (d, *J* = 5.7 Hz, 2H).

^{19}F NMR (376 MHz, $CDCl_3$): δ 114.3.

^{13}C NMR (100 MHz, $CDCl_3$): δ 163.7, 161.3, 158.2, 133.1, 129.8, 129.6, 121.7, 121.6, 116.0 (d, *J* = 21.7 Hz), 115.0, 114.9, 55.9, 43.4, 29.8.

IR ν_{max} (neat): 3445, 2251, 2125, 1656, 1511, 1495, 1223, 1054, 1026, 1007 cm^{-1}.

HRMS (ESI, $C_{18}H_{18}FN_4O_2$, +ve mode): *m/z* $[M+H]^+$ calcd. 341.1408, found 341.1412.

2-(4-Fluorophenyl)-*N*-((4-(*p*-tolyl)-1*H*-1,2,3-triazol-1-yl)methyl)acetamide (**8c**)

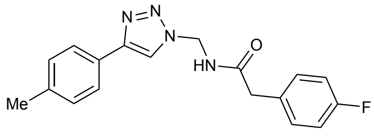

Prepared using General Procedure A. Yellow oil (60 mg, 92%). Purification on silica gel using 1/9 PEt/EtOAc + 2% NEt_3.

^{1}H NMR (400 MHz, DMSO-d$_6$): δ 8.84 (t, *J* = 5.9 Hz, 1H), 8.47 (s, 1H), 7.74–7.76 (m, 2H), 7.32–7.35 (m, 2H), 7.25–7.27 (m, 2H), 7.14–7.19 (m, 2H), 5.19 (s, 2H), 4.32 (d, *J* = 5.7 Hz, 2H), 2.33 (s, 3H).

^{19}F NMR (376 MHz, DMSO-d$_6$): δ 115.9.

^{13}C NMR (100 MHz, $CDCl_3$): δ 165.5, 162.5, 160.0, 146.2, 137.1, 135.0, 129.4, 128.0, 125.0, 122.5, 115.2 (d, *J* = 21.4 Hz), 51.7, 41.7, 20.8.

IR ν_{max} (neat): 3093, 3023, 2915, 2859, 1502, 1448, 1349, 1279, 1193, 1076, 1045 cm^{-1}.

HRMS (ESI, $C_{18}H_{18}FN_4O$, +ve mode): *m/z* $[M+H]^+$ calcd. 325.1459, found 325.1463.

3-(4-(5,6-Dimethyl-1*H*-benzo[*d*]imidazol-1-yl)-1*H*-1,2,3-triazol-1-yl)-7-hydroxy-2*H*-chro-
-men-2-one (**9a**)

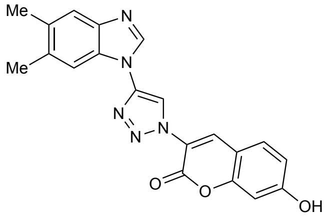

Prepared using General Procedure A. Yellow oil (73 mg, 98%). Purification on silica gel using 9/1 DCM/MeOH + 2% NEt_3.

^{1}H NMR (400 MHz, DMSO-*d*$_6$): δ 11.00 (s, 1H), 9.15 (s, 1H), 8.72 (s, 2H), 7.80–7.60 (m, 3H), 6.96-6.93 (m, 1H), 6.89 (d, *J* = 1.8 Hz, 1H), 2.38 (s, 3H), 2.34 (s, 3H).

^{13}C NMR (100 MHz, DMSO-*d*$_6$): δ 162.8, 156.2, 154.9, 137.6, 132.9, 131.2, 119.1, 116.8, 114.4, 110.2, 102.3, 20.1, 19.8. Seven signals not observed/coincident.

IR ν_{max} (neat): 3126, 2921, 2949, 1722, 1703, 1607, 1590, 1465, 1417, 1380 cm^{-1}.

HRMS (ESI, $C_{20}H_{16}N_5O_3$, +ve mode): *m/z* $[M+H]^+$ calcd. 374.1248, found 374.1249.

7-Hydroxy-3-(4-(phenoxymethyl)-1*H*-1,2,3-triazol-1-yl)-2*H*-chromen-2-one (**9b**)

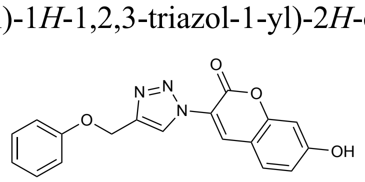

Prepared using General Procedure A. Yellow oil (64 mg, 96%). Purification on silica gel using 9/1 DCM/MeOH + 2% NEt_3.

^{1}H NMR (500 MHz, DMSO-d_6): δ 10.91 (br. s, 1H), 8.67 (s, 1H), 8.62 (s, 1H), 7.75 (d, *J* = 7.9 Hz, 1H), 7.32 (t, *J* = 7.6 Hz, 2H), 7.07 (d, *J* = 8.1 Hz, 2H), 6.95–6.96 (m, 1H), 6.94–6.85 (m, 2H), 5.25 (s, 2H).
^{13}C NMR (125 MHz, DMSO-d_6): δ.158.0, 156.3, 142.9, 136.5, 131.0, 129.5, 125.5, 120.9, 114.7, 60.6. Six signals not observed/coincident.
IR ν_{max} (neat): 3014, 2994, 2981, 1687, 1625, 1614, 1597, 1421, 1389, 1229 cm^{-1}.
HRMS (ESI, $C_{18}H_{14}N_3O_4$, +ve mode): *m/z* $[M+H]^+$ calcd. 336.0979, found 336.0984.

7-Hydroxy-3-(4-(*p*-tolyl)-1*H*-1,2,3-triazol-1-yl)-2*H*-chromen-2-one (**9c**)

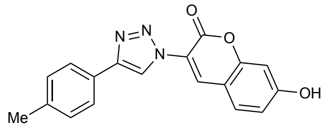

Prepared using General Procedure A. Yellow oil (55 mg, 86%). Purification on silica gel using 9/1 DCM/MeOH + 2% NEt_3.
^{1}H NMR (500 MHz, DMSO-d_6): δ 10.95 (br. s, 1H), 8.93 (s, 1H), 8.64 (s, 1H), 7.84 (d, *J* = 7.7 Hz, 2H), 7.77 (d, *J* = 8.4 Hz, 1H), 7.30 (d, *J* = 7.7 Hz, 2H), 6.94–6.89 (m, 2H), 2.35 (s, 3H).
^{13}C NMR (125 MHz, DMSO-d_6): δ 162.5, 156.3, 154.7, 146.5, 137.6, 136.7, 131.0, 129.5, 127.3, 125.3, 121.7, 119.2, 114.2, 110.3, 102.2, 20.8.
IR ν_{max} (neat): 3167, 3079, 3014, 1731, 1714, 1701, 1603, 1404, 1235 cm^{-1}.
HRMS (ESI, $C_{18}H_{14}N_3O_3$, +ve mode): *m/z* $[M+H]^+$ calcd. 320.1030, found 320.1033.
N-(3-(4-(5,6-Dimethyl-1*H*-benzo[*d*]imidazol-1-yl)-1*H*-1,2,3-triazol-1-yl)propyl)-5-(di--methylamino)naphthalene-1-sulfonamide (**10a**)[3]

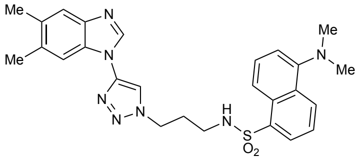

Prepared using General Procedure A (0.1 mmol scale). Yellow oil (52 mg, 96%). Purification on silica gel using 9/1 DCM/MeOH + 2% NEt_3.
^{1}H NMR (500 MHz, $CDCl_3$): δ 8.52 (d, *J* = 8.6 Hz, 1H), 8.27 (d, *J* = 8.9 Hz, 1H), 8.24 (s, 1H), 8.21 (d, *J* = 7.0 Hz, 1H), 7.80 (s, 1H), 7.61 (s, 1H), 7.55 (t, *J* = 7.8 Hz, 1H), 7.50–7.46 (m, 2H), 7.16 (d, *J* = 7.6 Hz, 1H), 5.38 (t, *J* = 6.0 Hz, 1H), 4.54 (t, *J* = 6.0 Hz, 2H), 2.95–2.92 (m, 2H), 2.86 (s, 6H), 2.39 (s, 6H), 2.18–2.13 (m, 2H).

^{13}**C NMR** (500 MHz, $CDCl_3$): δ 152.4, 142.6, 142.4, 140.5, 134.2, 133.8, 132.4, 131.0, 130.1, 129.9, 129.6, 128.9, 123.4, 120.7, 118.3, 115.5, 114.7, 111.4, 47.7, 45.5, 39.9, 30.2, 20.7, 20.4. One signal not observed/coincident.

5-(Dimethylamino)-*N*-(3-(4-(phenoxymethyl)-1*H*-1,2,3-triazol-1-yl)propyl)naphthalene-1-sulfonamide (**10b**)

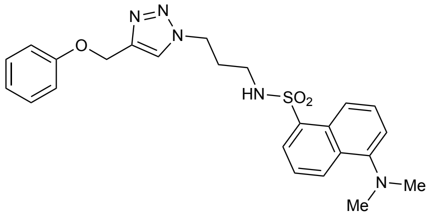

Prepared using General Procedure A (0.1 mmol scale). Yellow oil (46 mg, 98%). Purification on silica gel using 9/1 DCM/MeOH + 2% NEt_3.

^{1}**H NMR** (500 MHz, $CDCl_3$): δ 8.54 (d, *J* = 8.4 Hz, 1H), 8.25 (d, *J* = 8.7 Hz, 1H), 8.19 (d, *J* = 7.1 Hz, 1H), 7.59 (t, *J* = 8.2 Hz, 1H), 7.51 (t, *J* = 7.9 Hz, 1H), 7.47 (s, 1H), 7.29 (t, *J* = 8.0 Hz, 2H), 7.19 (d, *J* = 7.6 Hz, 1H), 6.98 (d, *J* = 7.8 Hz, 3H), 5.17 (s, 2H), 4.96 (t, *J* = 6.2 Hz, 1H), 4.37 (t, *J* = 6.5 Hz, 2H), 2.88 (s, 6H), 2.87–2.85 (m, 2H), 2.04 (app. quint, *J* = 6.3 Hz, 2H).

^{13}**C NMR** (100 MHz, DMSO-d_6): δ 158.3, 152.4, 134.4, 130.9, 130.1, 130.0, 129.7, 128.9, 123.4, 121.9, 121.5, 118.6, 115.5, 115.0, 114.9, 56.3, 45.5, 40.1, 30.2. Two signals not observed/coincident.

IR ν_{max} (neat): 3292, 3142, 3062, 2939, 2867, 2829, 2785, 1587, 1493, 1459, 1312, 1214, 1143 cm^{-1}.

HRMS (ESI, $C_{24}H_{28}N_5O_3S$, +ve mode): *m/z* $[M+H]^+$ calcd. 466.1907, found 466.1906.

5-(Dimethylamino)-*N*-(3-(4-(*p*-tolyl)-1*H*-1,2,3-triazol-1-yl)propyl)naphthalene-1-sulfon--amide (**10c**)

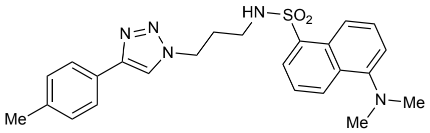

Prepared using General Procedure A (0.1 mmol scale). Yellow oil (44 mg, 98%). Purification on silica gel using 9/1 DCM/MeOH + 2% NEt_3.

^{1}**H NMR** (500 MHz, $CDCl_3$): δ 8.53 (d, *J* = 8.6 Hz, 1H), 8.26 (d, *J* = 8.6 Hz, 1H), 8.21 (d, *J* = 7.5 Hz, 1H), 7.67 (d, *J* = 8.0 Hz, 2H), 7.56–7.60 (m, 2H), 7.49 (t, *J* = 8.0 Hz, 1H), 7.23 (d, *J* = 8.0 Hz, 2H), 7.19 (d, *J* = 7.8 Hz, 1H), 5.01 (t, *J* = 6.2 Hz, 1H), 4.42 (t, *J* = 6.4 Hz, 2H), 2.90 (t, *J* = 6.3 Hz, 2H), 2.88 (s, 6H), 2.38 (s, 3H), 2.07 (app. quint, *J* = 6.3 Hz, 2H).

^{13}C NMR (100 MHz, $CDCl_3$): δ 138.3, 134.3, 130.9, 130.1, 130.0, 129.7, 128.9, 125.8, 123.4, 118.5, 115.5, 47.1, 45.5, 40.1, 30.4, 21.4. Five signals not observed/coincident.

IR ν_{max} (neat): 3285, 3132, 2928, 2865, 1688, 1574, 1455, 1316, 1143 cm^{-1}.

HRMS (ESI, $C_{24}H_{28}N_5O_2S$, +ve mode): *m/z* $[M+H]^+$ calcd. 450.1958, found 450.1966.

1-(3',6'-Dihydroxy-3-oxo-3*H*-spiro[isobenzofuran-1,9'-xanthen]-5-yl)-3-(3-(4-(5,6-dimethyl-

1*H*-benzo[*d*]imidazol-1-yl)-1*H*-1,2,3-triazol-1-yl)propyl)thiourea (**11a**)[3]

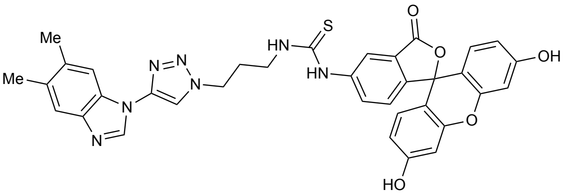

Prepared using General Procedure A (0.1 mmol scale). Orange gum (51 mg, 78%). Purification on silica gel using 9/1 DCM/MeOH + 2% NEt_3.

^{1}H NMR (400 MHz, DMSO-d_6): δ 9.32 (br. s, 1H), 9.00 (s, 1H), 8.45 (s, 1H), 7.86 (d, *J* = 8.0 Hz, 1H), 7.82 (s, 1H), 7.65−7.62 (m, 2H), 7.16 (d, *J* = 8.3 Hz, 1H), 6.72−6.68 (m, 2H), 6.60−6.56 (m, 3H), 4.66 (t, *J* = 6.9 Hz, 2H), 3.51 (s, 2H), 3.16 (s, 2H), 2.40 (s, 3H), 2.38 (s, 3H).

^{13}C NMR could not be obtained due to relaxation issues.

HRMS (ESI, $C_{35}H_{28}N_7O_5S$, -ve mode): *m/z* $[M-OH]^-$ calcd. 642.1923, found 642.1632. Error >5 ppm, due to fluorescein protonation state variation.[19]

1-(3',6'-Dihydroxy-3-oxo-3*H*-spiro[isobenzofuran-1,9'-xanthen]-5-yl)-3-(3-(4-(phenoxy-
-methyl)-1*H*-1,2,3-triazol-1-yl)propyl)thiourea (**11b**)

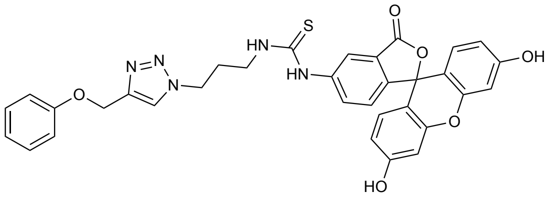

Prepared using General Procedure A (0.1 mmol scale). Orange gum (52 mg, 79%). Purification on silica gel using 9/1 DCM/MeOH + 2% NEt_3.

^{1}H NMR (400 MHz, DMSO-d_6): δ 8.39 (br. s, 1H), 8.31 (br. s, 1H), 7.91 (br. s, 1H), 6.92–7.19 (m, 14H), 5.08 (br. s, 2H), 4.45 (br. s, 2H), 3.54 (br. s, 2H), 2.17 (br. s, 2H).

^{13}C NMR could not be obtained due to relaxation issues.

IR ν_{max} (neat): 3437, 2259, 2190, 2130, 1770, 1693, 1152, 1026 cm^{-1}.

HRMS (ESI, $C_{33}H_{28}N_5O_6S$, +ve mode): *m/z* $[M+H-S]^+$ calcd. 590.2034, found 590.2031, $C_{33}H_{28}N_5O_6S$, +ve mode): *m/z* $[M+H-OH]^+$ calcd. 605.1727, found 605.1678, $C_{33}H_{28}N_5O_6S$, +ve mode): *m/z* $[M+H]^+$ calcd. 606.1806, found 606.1979. Error >5 ppm, due to fluorescein protonation state variation.[19]

1-(3',6'-Dihydroxy-3-oxo-3*H*-spiro[isobenzofuran-1,9'-xanthen]-5-yl)-3-(3-(4-(*p*-tolyl)-1*H*-1,2,3-triazol-1-yl)propyl)thiourea (**11c**)

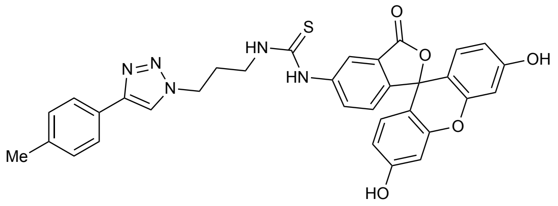

Prepared using General Procedure A (0.1 mmol scale). Orange gum (48 mg, 79%). Purification on silica gel using 9/1 DCM/MeOH + 2% NEt_3.

^{1}H NMR (400 MHz, DMSO-d_6): δ 8.43 (br. s, 2H), 7.96 (br. s, 1H), 7.65 (br. s, 2H), 6.97–7.18 (m, 11H), 4.48 (br. s, 2H), 3.58 (br. s, 2H), 2.22 (br. s, 5H). One drop of pyridine-d_5 added to DMSO-d_6 for solubility resulting in the loss of phenolic protons; basic form of fluorescein.[20]

^{13}C NMR could not be obtained due to relaxation issues.

IR ν_{max} (neat): 3486, 2255, 1766, 1335, 1178, 1154, 1026, 1009 cm^{-1}.

HRMS (ESI, $C_{33}H_{28}N_5O_5S$, +ve mode): *m/z* $[M+H]^+$ calcd. 606.1806, found 606.1977, $C_{33}H_{27}N_5O_5SNa$, +ve mode): *m/z* $[M+Na]^+$ calcd. 628.1625, found 628.1800. Error >5 ppm, due to fluorescein protonation state variation.[19]

1-((2*R*,4*S*,5*S*)-4-(4-(5,6-Dimethyl-1*H*-benzo[*d*]imidazol-1-yl)-1*H*-1,2,3-triazol-1-yl)-5-(hydroxymethyl)tetrahydrofuran-2-yl)-5-methylpyrimidine-2,4(1*H*,3*H*)-dione (**12a**)

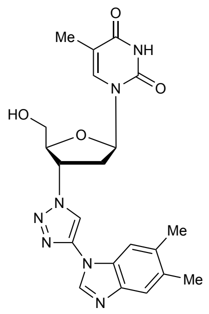

Prepared using General Procedure A (0.1 mmol scale). Yellow oil (44 mg, Quant). Purification on silica gel using 9/1 DCM/MeOH + 2% NEt_3.

^{1}H NMR (500 MHz, DMSO-d_6): δ 11.38 (s, 1H), 8.90 (s, 2H), 7.85–7.61 (m, 3H), 6.49 (s, 1H), 5.50 (s, 1H), 5.38 (s, 1H), 4.38 (s, 1H), 3.78–3.74 (m, 2H), 2.89–2.76 (m, 2H), 2.36 (s, 3H), 2.32 (s, 3H), 1.83 (s, 3H).
^{13}C NMR (125 MHz, DMSO-d_6): δ 165.3, 151.5, 137.5, 134.8, 117.0, 111.0, 85.3, 85.2, 61.7, 61.4, 37.8, 20.8, 20.6, 12.9. Seven signals not observed/coincident.
IR ν_{max} (neat): 3341, 2926, 2255, 2128, 1699, 1680, 1584, 1470, 1405, 1283 cm^{-1}.
HRMS (ESI, $C_{21}H_{24}N_7O_4$, +ve mode): *m/z* $[M+H]^+$ calcd. 438.1884, found 438.1883.

1-((2*R*,4*S*,5*S*)-5-(Hydroxymethyl)-4-(4-(phenoxymethyl)-1*H*-1,2,3-triazol-1-yl)tetrahydro--furan-2-yl)-5-methylpyrimidine-2,4(1*H*,3*H*)-dione (**12b**)[21]

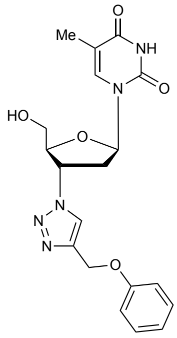

Prepared using General Procedure A (0.1 mmol scale). Yellow oil (38 mg, 96%). Purification on silica gel using 9/1 DCM/MeOH + 2% NEt_3.
^{1}H NMR (500 MHz, DMSO-d_6): δ 11.32 (br. s, 1H), 8.43 (s, 1H), 7.82 (s, 1H), 7.31 (t, *J* = 7.8 Hz, 2H), 7.04 (d, *J* = 8.0 Hz, 2H), 6.96 (t, *J* = 7.3 Hz, 1H), 6.43 (t, *J* = 6.4 Hz, 1H), 5.42–5.38 (m, 1H), 5.28 (t, *J* = 5.1 Hz, 1H), 5.15 (s, 2H), 4.25–4.22 (m, 1H), 3.73–3.69 (m, 1H), 3.65–3.58 (m, 1H), 2.78–2.72 (m, 1H), 2.41–2.24 (m, 1H), 1.81 (s, 3H).
^{13}C NMR (125 MHz, DMSO-d_6): δ 163.7, 158.0, 150.4, 136.2, 129.5, 124.2, 120.8, 114.6, 109.6, 84.4, 83.9, 61.0, 60.1, 59.3, 37.1, 36.1, 12.2.

1-((2*R*,4*S*,5*S*)-5-(Hydroxymethyl)-4-(4-(*p*-tolyl)-1*H*-1,2,3-triazol-1-yl)tetrahydrofuran-2-yl)-5-methylpyrimidine-2,4(1*H*,3*H*)-dione (**12c**)

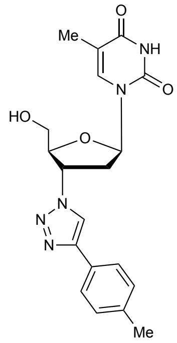

Prepared using General Procedure A (0.1 mmol scale). Yellow oil (39 mg, 99%). Purification on silica gel using 9/1 DCM/MeOH + 2% NEt_3.

^{1}H NMR (500 MHz, DMSO-*d*$_6$): δ 11.35 (s, 1H), 8.72 (s, 1H), 7.83 (d, J = 1.1 Hz, 1H), 7.75 (s, 1H), 7.74 (s, 1H), 7.28 (s, 1H), 7.26 (s, 1H), 6.45 (t, J = 6.5 Hz, 1H), 5.41–5.37 (m, 1H), 4.29–4.27 (m, 1H), 3.74–3.65 (m, 2H), 3.30 (d, J = 11.8 Hz, 1H), 2.82–2.66 (m, 2H), 2.33 (s, 3H), 1.82 (s, 3H).

^{13}C NMR (125 MHz, DMSO-*d*$_6$): δ 163.7, 150.4, 146.6, 137.3, 136.2, 129.5, 127.8, 125.1, 120.5, 109.6, 84.4, 83.9, 60.8, 59.3, 37.1, 20.8, 12.2.

IR ν$_{max}$ (neat): 3473, 2251, 2127, 1656, 1273, 1054, 1026, 1006 cm^{-1}.

HRMS (ESI, $C_{19}H_{22}N_5O_4$, +ve mode): *m/z* [M+H]$^+$ calcd. 384.1666, found 384.1669.

(2*R*,3*R*,4*S*,5*S*,6*R*)-2-(Acetoxymethyl)-6-(2-(4-(5,6-dimethyl-1*H*-benzo[*d*]imidazol-1-yl)-1*H*-1,2,3-triazol-1-yl)ethoxy)tetrahydro-2*H*-pyran-3,4,5-triyl triacetate (**13a**)

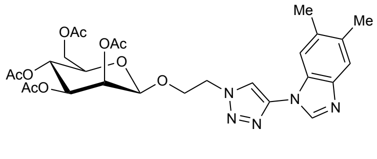

Prepared using General Procedure A (0.1 mmol scale). Yellow oil (52 mg, 88%). Purification on silica gel using 9/1 DCM/MeOH + 2% NEt_3.

^{1}H NMR (500 MHz, DMSO-*d*$_6$): δ 8.79 (s, 1H), 8.50 (s, 1H), 7.61 (s, 1H), 7.54 (s, 1H), 5.10–5.09 (m, 1H), 5.04–5.02 (m, 2H), 4.96 (d, J = 1.4 Hz, 1H), 4.78 (t, J = 5.8 Hz, 2H), 4.12–4.08 (m, 1H), 4.04–3.95 (m, 3H), 3.50–3.47 (m, 1H), 2.36 (s, 3H), 2.34 (s, 3H), 2.08 (s, 3H), 1.97 (s, 3H), 1.90 (s, 3H), 1.79 (s, 3H).

^{13}C NMR (100 MHz, MeOD): 172.1, 171.5, 171.4, 171.2, 135.3, 133.9, 120.5, 118.1, 113.1, 98.3, 70.5, 70.3, 70.1, 66.9, 63.3, 53.7, 51.8, 47.8, 20.7, 20.6, 20.3, 20.2, 9.25. Four signals not observed/coincident.

IR ν$_{max}$ (neat): 2978, 2945, 2623, 2606, 2498, 2047, 1749, 1478, 1400, 1229, 1039 cm^{-1}.

HRMS (ESI, $C_{27}H_{34}N_5O_{10}$, +ve mode): *m/z* [M+H]$^+$ calcd. 588.2300, found 588.2291.

(2*R*,3*R*,4*S*,5*S*,6*R*)-2-(Acetoxymethyl)-6-(2-(4-(phenoxymethyl)-1*H*-1,2,3-triazol-1-yl)-
-ethoxy)tetrahydro-2*H*-pyran-3,4,5-triyl triacetate (**13b**)

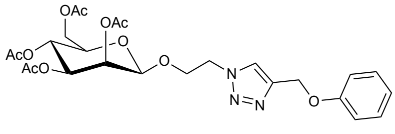

Prepared using General Procedure A (0.1 mmol scale). Yellow oil (43 mg, 78%). Purification on silica gel using 9/1 DCM/MeOH + 2% NEt_3.

^{1}H NMR (400 MHz, MeOD): δ 8.13 (s, 1H), 7.30–7.26 (m, 2H), 7.03–7.01 (m, 2H), 6.95 (t, *J* = 7.4 Hz, 1H), 5.20–5.19 (m, 1H), 5.18 (s, 2H), 5.17–5.16 (m, 2H), 4.84–4.83 (m, 1H), 4.71–4.69 (m, 2H), 4.16–4.09 (m, 2H), 4.01–3.92 (m, 2H), 3.41–3.38 (m, 1H), 2.11 (s, 3H), 2.05 (s, 3H), 1.95 (s, 3H), 1.94 (s, 3H).

^{13}C NMR (100 MHz, MeOD) 172.3, 171.5, 171.5, 171.4, 159.8, 145.3, 130.6, 126.3, 122.3, 115.9, 98.5, 70.6, 70.4, 70.1, 67.2, 66.9, 63.2, 62.4, 51.0, 20.6, 20.5. Two signals not observed/coincident.

IR ν_{max} (neat): 3447, 3409, 2924, 1742, 1600, 1495, 1242, 1138, 1095, 1054, 1035 cm^{-1}.

HRMS (ESI, $C_{25}H_{32}N_3O_{11}$, +ve mode): *m/z* $[M+H]^+$ calcd. 550.2031, found 550.2021.

(2*R*,3*R*,4*S*,5*S*,6*R*)-2-(Acetoxymethyl)-6-(2-(4-(*p*-tolyl)-1*H*-1,2,3-triazol-1-yl)ethoxy)tetra--hydro-2*H*-pyran-3,4,5-triyl triacetate (**13c**)

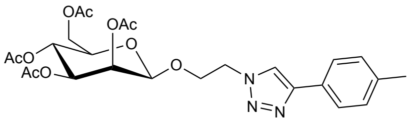

Prepared using General Procedure A (0.1 mmol scale). Yellow oil (47 mg, 89%). Purification on silica gel using 9/1 DCM/MeOH + 2% NEt_3.

^{1}H NMR (400 MHz, MeOD): δ 8.32 (s, 1H), 7.74–7.72 (m, 2H), 7.26–7.24 (m, 2H), 5.23–5.22 (m, 1H), 5.17–5.08 (m, 2H), 4.86 (d, *J* = 1.6 Hz, 1H), 4.74–4.71 (m, 2H), 4.15–3.92 (m, 4H), 3.15–3.11 (m, 1H), 2.35 (s, 3H), 2.10 (s, 3H), 2.01 (s, 3H), 1.95 (s, 3H), 1.72 (s, 3H).

^{13}C NMR (100 MHz, MeOD): δ 172.2, 171.6, 171.5, 171.4, 149.0, 139.5, 130.6, 128.8, 126.8, 123.1, 98.1, 70.6, 70.4, 69.9, 66.9, 66.8, 63.2, 51.1, 21.2, 20.6, 20.5, 20.2. One signal not observed/coincident.

IR ν_{max} (neat): 3136, 3114, 2960, 2926, 1745, 1454, 1370, 1225, 1139, 1091, 1048 cm^{-1}.

HRMS (ESI, $C_{25}H_{32}N_3O_{10}$, +ve mode): *m/z* $[M+H]^+$ calcd. 534.2082, found 534.2071.

1-(1-(3,3,4,4,5,5,6,6,7,7,8,8,9,9,10,10,10-Heptadecafluorodecyl)-1*H*-1,2,3-triazol-4-yl)-5,6-dimethyl-1*H*-benzo[*d*]imidazole (**14a**)

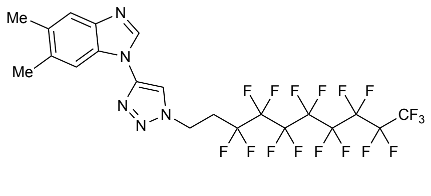

Prepared using General Procedure A (0.1 mmol scale). Yellow oil (51 mg, 78%). Purification on silica gel using 6/4 PE/EtOAc.

^{1}H NMR (400 MHz, $CDCl_3$): δ 8.28 (br, 1H), 7.85 (s, 1H), 7.63 (s, 1H), 7.45 (s, 1H), 4.81 (t, J = 7.2 Hz, 2H), 3.01–2.87 (m, 2H), 2.41 (s, 3H), 2.40 (s, 3H).

^{19}F NMR (376 MHz, $CDCl_3$): δ -80.7 (t, J = 9.9 Hz), -113.9 (app. quint, J = 15.3 Hz), -121.5, -121.8, -122.6, -123.3, -126.0.

^{13}C NMR could not be obtained due to relaxation issues.[2]

IR ν_{max} (neat): 3447, 2251, 2125, 1656, 1054, 1026, 1007 cm^{-1}.

HRMS (ESI, $C_{21}H_{15}F_{17}N_5$, +ve mode): *m/z* $[M+H]^+$ calcd. 660.1051, found 660.1053.

1-(3,3,4,4,5,5,6,6,7,7,8,8,9,9,10,10,10-Heptadecafluorodecyl)-4-(phenoxymethyl)-1*H*-1,2,3-triazole (**14b**)

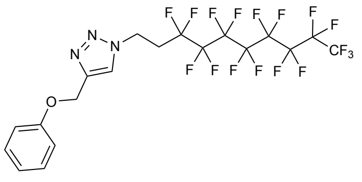

Prepared using General Procedure A (0.1 mmol scale). Yellow oil (43 mg, 69%). Purification on silica gel using 6/4 PE/EtOAc.

^{1}H NMR (400 MHz, $CDCl_3$): δ 7.66 (s, 1H), 7.32–7.28 (m, 2H), 7.00–6.96 (m, 3H), 5.23 (s, 2H), 4.68 (t, J = 7.3 Hz, 2H), 2.90–2.77 (app. sept, J = 7.5 Hz, 2H).

^{19}F NMR (376 MHz, $CDCl_3$): δ -80.7 (t, J = 9.9 Hz), -114.0 (app. quint, J = 15.4 Hz), -121.5 (d, J = 10.2 Hz), -121.8, -122.6, -123.4, -126.0.

^{13}C NMR (100 MHz, MeOD): δ 129.7, 123.2, 121.5, 114.9, 62.1, 29.8.

IR ν_{max} (neat): 2919, 2850, 1602, 1498, 1459, 1199, 1147, 1082, 1045 cm^{-1}.

HRMS (ESI, $C_{19}H_{13}F_{17}N_3O$, +ve mode): *m/z* $[M+H]^+$ calcd. 622.0782, found 622.0777.

1-(3,3,4,4,5,5,6,6,7,7,8,8,9,9,10,10,10-Heptadecafluorodecyl)-4-(*p*-tolyl)-1*H*-1,2,3-triazole (**14c**)

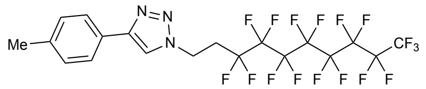

Prepared using General Procedure A (0.1 mmol scale). Yellow oil (48 mg, 79%). Purification on silica gel using 8/2 PE/EtOAc.

^{1}H NMR (400 MHz, $CDCl_3$): δ 7.78 (s, 1H), 7.72 (d, J = 8.1 Hz, 2H), 7.24 (s, 2H), 4.73 (t, J = 7.5 Hz, 2H), 2.87 (app. sept, J = 7.4 Hz, 2H), 2.39 (s, 3H).

^{19}F NMR (376 MHz, $CDCl_3$): δ -80.7 (t, *J* = 9.8 Hz), -114.0 (app. quint, *J* = 15.2 Hz), -121.5, -121.8, -122.6, -123.3, -126.0.

^{13}C NMR (100 MHz, MeOD): δ 138.5, 129.8, 127.5, 125.9, 42.5, 32.1, 21.4.

IR ν_{max} (neat): 3108, 2921, 1502, 1400, 1329, 1197, 1143, 1115, 1048 cm^{-1}.

HRMS (ESI, $C_{19}H_{13}F_{17}N_3$, +ve mode): *m/z* $[M+H]^+$ calcd. 606.0833, found 606.0837.

N-(2-(2-(2-(2-(4-(5,6-Dimethyl-1*H*-benzo[*d*]imidazol-1-yl)-1*H*-1,2,3-triazol-1-yl)ethoxy) ethoxy)ethoxy)ethyl)-5-((3a*R*,4*R*,6a*S*)-2-oxohexahydro-1*H*-thieno[3,4-*d*]imidazol-4-yl) pentanamide (**15a**)[3]

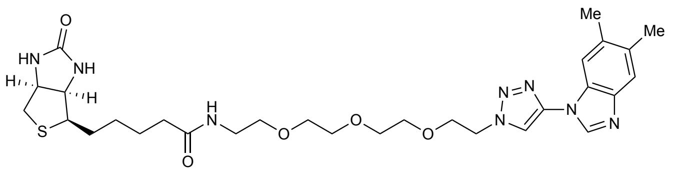

Prepared using General Procedure A (0.1 mmol scale). Yellow oil (55 mg, 89%). Purification on silica gel using 9/1 DCM/MeOH + 2% NEt_3.

^{1}H NMR (400 MHz, MeOD): δ 8.52 (s, 2H), 7.63 (br, 1H), 7.55 (br, 1H), 4.71 (t, *J* = 5.2 Hz, 2H), 4.45 (dd, *J* = 7.8, 4.9 Hz, 1H), 4.25 (dd, *J* = 7.9, 4.5 Hz, 1H), 3.99 (t, *J* = 4.9 Hz, 2H), 3.69−3.66 (m, 2H), 3.64−3.62 (m, 2H), 3.58−3.55 (m, 2H), 3.49−3.47 (m, 2H), 3.38 (t, *J* = 5.4 Hz, 2H), 3.24 (t, *J* = 5.4 Hz, 2H), 3.17−3.12 (m, 1H), 2.88 (dd, *J* = 12.8, 5.0 Hz, 1H), 2.68 (d, *J* = 12.7 Hz, 1H), 2.41 (s, 3H), 2.40 (s, 3H), 2.17−2.13 (m, 2H), 1.73−1.50 (m, 4H), 1.42−1.34 (m, 2H).

^{13}C NMR (100 MHz, MeOD): δ 176.0, 166.1, 135.2, 133.9, 120.6, 118.2, 112.9, 71.5, 71.4, 71.1, 70.5, 63.3, 61.6, 57.0, 52.3, 41.0, 40.2, 36.7, 29.7, 29.4, 26.8, 20.7, 20.3. Six signals not observed/coincident.

5-((3a*R*,4*R*,6a*S*)-2-Oxohexahydro-1*H*-thieno[3,4-*d*]imidazol-4-yl)-*N*-(2-(2-(2-(2-(4-(phen- -oxymethyl)-1*H*-1,2,3-triazol-1-yl)ethoxy)ethoxy)ethoxy)ethyl)pentanamide (**15b**)

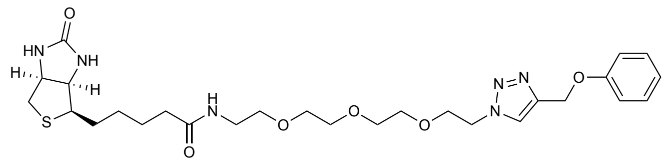

Prepared using General Procedure A (0.1 mmol scale). Yellow oil (52 mg, 91%). Purification on silica gel using 9/1 DCM/MeOH + 2% NEt_3.

^{1}H NMR (500 MHz, MeOD): δ 8.11 (s, 1H), 7.30–7.26 (m, 2H), 7.02–7.00 (m, 2H), 6.97–6.93 (m, 1H), 5.17 (s, 2H), 4.60 (t, *J* = 5.0 Hz, 2H), 4.49–4.46 (m, 1H), 4.28 (dd, *J* = 7.9, 4.5

Hz, 1H), 3.90 (t, J = 5.0 Hz, 2H), 3.61–3.58 (m, 4H), 3.56 (s, 4H), 3.49 (t, J = 5.5 Hz, 2H), 3.35–3.32 (m, 2H), 3.20–3.15 (m, 1H), 2.90 (dd, J = 12.7, 5.0 Hz, 1H), 2.69 (d, J = 12.7 Hz, 1H), 2.19 (t, J = 7.4 Hz, 2H), 1.76–1.54 (m, 4H), 1.46–1.40 (m, 2H).

^{13}C NMR (100 MHz, MeOD): δ 176.1, 166.1, 159.8, 145.0, 130.6, 126.1, 122.3, 71.6, 71.5, 71.4, 71.2, 70.6, 70.3, 63.4, 62.3, 61.6, 57.0, 51.5, 41.0, 40.3, 36.7, 29.7, 29.5, 26.8. One signal not observed/coincident.

IR ν_{max} (neat): 3404, 2508, 2251, 2128, 2063, 1128, 1054, 1026, 1007 cm^{-1}.

HRMS (ESI, $C_{27}H_{41}N_6O_6S$, +ve mode): *m/z* [M+H]$^+$ calcd. 577.2803, found 577.2790.

5-((3a*R*,4*R*,6a*S*)-2-Oxohexahydro-1*H*-thieno[3,4-*d*]imidazol-4-yl)-*N*-(2-(2-(2-(2-(4-(*p*-tolyl)-1*H*-1,2,3-triazol-1-yl)ethoxy)ethoxy)ethoxy)ethyl)pentanamide (**15c**)

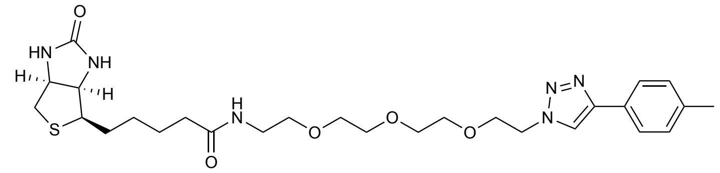

Prepared using General Procedure A (0.1 mmol scale). Yellow oil (51 mg, 91%). Purification on silica gel using 9/1 DCM/MeOH + 2% NEt_3.

^{1}H NMR (400 MHz, $CDCl_3$): δ 8.31–8.12 (m, 1H), 7.71–7.69 (m, 1H), 7.29–7.25 (m, 2H), 7.02–6.95 (m, 1H), 5.17–5.16 (m, 1H), 4.64–4.60 (m, 2H), 4.49–4.46 (m, 1H), 4.30–4.26 (m, 1H), 3.95–3.89 (m, 2H), 3.67–3.45 (m, 12H), 3.20–3.15 (m, 2H), 2.92–2.89 (m, 1H), 2.71–2.68 (m, 1H), 2.37–2.36 (m, 2H), 2.19–2.17 (m, 2H), 1.71–1.69 (m, 4H), 1.44–1.38 (m, 2H).

^{13}C NMR (100 MHz, MeOD): δ 130.6, 126.7, 122.7, 122.3, 115.9, 71.6, 71.5, 71.2, 70.5, 70.3, 63.4, 62.3, 61.6, 57.0, 51.5, 41.0, 40.3, 36.7, 29.7, 29.5, 26.8, 21.3. Three signals not observed/coincident.

IR ν_{max} (neat): 3404, 2474, 2244, 2214, 2138, 2071, 1125, 1093 cm^{-1}.

HRMS (ESI, $C_{27}H_{41}N_6O_5S$, +ve mode): *m/z* [M+H]$^+$ calcd. 561.2854, found 561.2841.

tert-Butyl-*N*2-(4-(((2-amino-4-oxo-1,4-dihydropteridin-6-yl)methyl)amino)benzoyl)-*N*5-(3-(4-(5,6-dimethyl-1*H*-benzo[*d*]imidazol-1-yl)-1*H*-1,2,3-triazol-1-yl)propyl)-*L*-glutami-
-nate (**16a**)

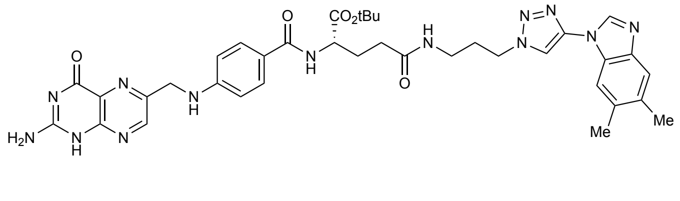

Prepared using General Procedure A (0.1 mmol scale). Yellow oil (45 mg, 60%). Purification on ISOLUTE Flash SCX-2 10g using NH_3 7 M in MeOH. Purity of resultant product was assessed by analytical HPLC (Phenomenex C18, 250 × 4.6 mm) at a flow rate of 1 mL/min,

RP-HPLC R_T = 21.2 min.

IR νmax (neat): 3493, 2251, 2125, 2062, 1126, 1056, 1026, 1007 cm^{-1}.

HRMS (ESI, $C_{37}H_{44}N_{13}O_5$, +ve mode): *m/z* $[M+H]^+$ calcd. 750.3583, found 750.3573.

tert-Butyl N_2-(4-(((2-amino-4-oxo-1,4-dihydropteridin-6-yl)methyl)amino)benzoyl)-N_5-(3-(4-(phenoxymethyl)-1*H*-1,2,3-triazol-1-yl)propyl)-*L*-glutaminate (**16b**)

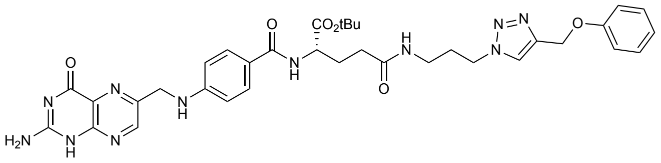

Prepared using General Procedure A (0.1 mmol scale). Yellow oil (40 mg, 56%). Purification on ISOLUTE Flash SCX-2 10g using NH_3 7 M in MeOH. Purity of resultant product was assessed by analytical HPLC (Phenomenex C18, 250 × 4.6 mm) at a flow rate of 1 mL/min, gradient 10–60% MeCN/0.1% TFA in 35 min.

RP-HPLC R_T= 23.0 min.

IR ν_{max} (neat): 3415, 2519, 2253, 2125, 2063, 1126, 1054, 1026, 1007 cm^{-1}.

HRMS (ESI, $C_{35}H_{42}N_{11}O_6$, +ve mode): *m/z* $[M+H]^+$ calcd. 712.3314, found 712.1180.

tert-Butyl N_2-(4-(((2-amino-4-oxo-1,4-dihydropteridin-6-yl)methyl)amino)benzoyl)-N_5-(3-(4-(*p*-tolyl)-1*H*-1,2,3-triazol-1-yl)propyl)-*L*-glutaminate (**16c**)

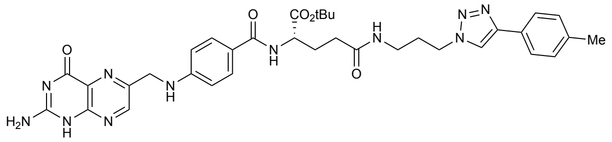

Prepared using General Procedure A (0.1 mmol scale). Yellow oil (38 mg, 54%). Purification on ISOLUTE Flash SCX-2 10g using NH_3 7 M in MeOH. Purity of resultant product was assessed by analytical HPLC (Phenomenex C18, 250 × 4.6 mm) at a flow rate of 1 mL/min, gradient 10–60% MeCN/0.1% TFA in 35 min.

RP-HPLC R_T= 25.5 min.

IR ν_{max} (neat): 3437, 2498, 2257, 2132, 2069, 1461, 1217, 1125, 1050, 1026 cm^{-1}.

HRMS (ESI, $C_{35}H_{42}N_{11}O_5$, +ve mode): *m/z* $[M+H]^+$ calcd. 696.3365, found 696.3374.

(1*R*,3*S*,8*R*,9*S*,13*R*,14*S*)-1,13-Dimethyl-17-((*R*)-6-methylheptan-2-yl)-2,3,4,7,8,9,10,11,12, 13,14,15,16,17-tetradecahydro-1*H*-cyclopenta[*a*]phenanthren-3-yl (3-(4-(5,6-dimethyl-1*H*-benzo[*d*]imidazol-1-yl)-1*H*-1,2,3-triazol-1-yl)propyl)carbamate (**17a**)

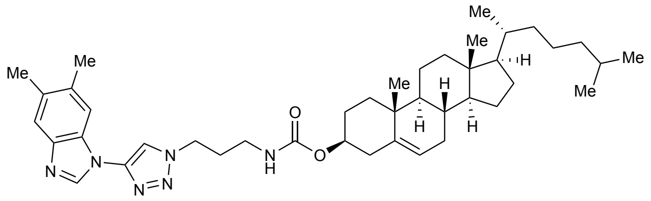

Prepared using General Procedure A (0.1 mmol scale in MeCN/*t*BuOH/H_2O 3/3/1). Yellow oil (59 mg, 87%). Purification on silica gel using 7/3 PE/EtOAc.

^{1}H NMR (500 MHz, $CDCl_3$): δ 8.08–7.65 (m, 4H), 5.32 (s, 1H), 5.10 (br, 1H), 4.53 (br, 2H), 4.47 (br, 1H), 3.27 (br, 2H), 2.38 (d, *J* = 16.7 Hz, 6H), 2.33–2.19 (m, 4H), 2.00–1.96 (m, 2H), 1.84–1.79 (m, 4H), 1.55–1.41 (m, 8H), 1/34–1.32 (m, 3H), 1.26–1.23 (m, 1H), 1.14–1.07 (m, 7H), 0.97 (s, 3H), 0.90 (d, *J* = 6.9 Hz, 3H), 0.85 (dd, *J* = 6.6, 2.2 Hz, 6H), 0.66 (s, 3H).

^{13}C NMR (100 MHz, $CDCl_3$) 156.7, 139.7, 133.8, 131.9, 12.7, 114.6, 74.9, 56.8, 56.2, 50.1, 48.5, 42.4, 39.8, 39.6, 38.6, 37.7, 37.0, 36.6, 36.3, 35.9, 32.0, 31.9, 30.8, 28.3, 28.2, 28.1, 24.4, 23.9, 22.9, 22.7, 21.1, 20.6, 20.4, 19.4, 18.8, 11.9. Six signals not observed/coincident.

IR ν_{max} (neat): 3400, 3127, 2935, 2867, 1690 1595, 1496, 1439, 1271, 1257, 1152, 1017 cm^{-1}.

HRMS (ESI, $C_{42}H_{63}N_6O_2$, [M+H] +ve mode): *m/z* calcd. 683.5007, found 683.5000.

(1*R*,3*S*,8*R*,9*S*,13*R*,14*S*)-1,13-Dimethyl-17-((*R*)-6-methylheptan-2-yl)-2,3,4,7,8,9,10,11,12, 13,14,15,16,17-tetradecahydro-1*H*-cyclopenta[*a*]phenanthren-3-yl (3-(4-(phenoxymethyl) -1*H*-1,2,3-triazol-1-yl)propyl)carbamate (**17b**)

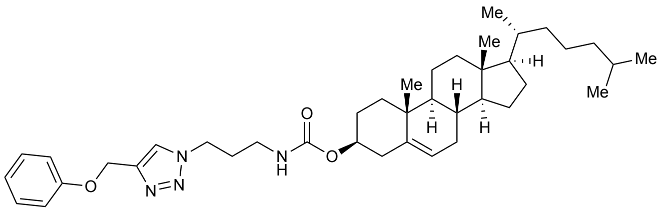

Prepared using General Procedure A (0.1 mmol scale in MeCN/*t*BuOH/H_2O 3/3/1). Yellow oil (59 mg, 92%). Purification on silica gel using 8/2 PE/EtOAc.

^{1}H NMR (400 MHz, $CDCl_3$): δ 7.73 (br, 1H), 7.28–7.26 (m, 2H), 6.98 (br, 2H), 5.37 (br, 1H), 5.22 (br, 1H), 4.92 (br, 1H), 4.45 (br, 2H), 3.20 (br, 2H), 2.34–2.27 (m, 2H), 2.13–1.83 (m, 8H), 1.56–1.43 (m, 7H), 1.33–1.25 (m, 5H), 1.15–1.10 (m, 7H), 1.00 (s, 6H), 0.91 (d, *J* = 6.1 Hz, 3H), 0.86 (d, *J* = 6.2 Hz, 6H), 0.67 (s, 3H).

^{13}C NMR (100 MHz, $CDCl_3$): δ 158.4, 156.5, 139.8, 129.7, 122.7, 121.4, 114.9, 74.7, 62.1, 56.9, 56.3, 50.1, 47.8, 42.4, 39.9, 39.6, 38.7, 37.8, 37.1, 36.7, 36.3, 35.9, 32.0, 31.9, 30.8,

28.3, 28.2, 28.1, 24.4, 24.0, 22.9, 22.7, 21.2, 19.4, 18.8, 12.0. One signal not observed/coincident.

IR ν_{max} (neat): 3369, 3341, 2930, 2863, 2091, 1692, 1599, 1439, 1223, 1046 cm^{-1}.

HRMS (ESI, $C_{40}H_{61}N_4O_3$, +ve mode): *m/z* $[M+H]^+$ calcd. 645.4738, found 645.4731.

(1*R*,3*S*,8*R*,9*S*,13*R*,14*S*)-1,13-Dimethyl-17-((*R*)-6-methylheptan-2-yl)-2,3,4,7,8,9,10,11,12,13,14,15,16,17-tetradecahydro-1*H*-cyclopenta[*a*]phenanthren-3-yl (3-(4-(*p*-tolyl)-1*H*-1,2,3-triazol-1-yl)propyl)carbamate (**17c**)

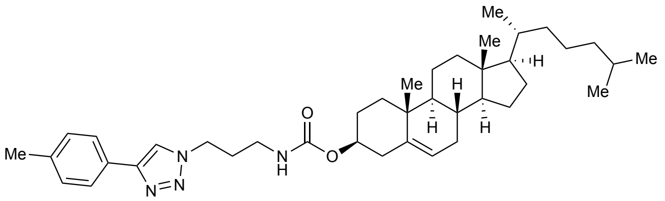

Prepared using General Procedure A (0.1 mmol scale in MeCN/*t*BuOH/H_2O 3/3/1). Yellow oil (54 mg, 86%). Purification on silica gel using 9/1 PE/EtOAc.

^{1}H NMR (400 MHz, $CDCl_3$): δ 7.81 (br. s, 1H), 7.70 (d, *J* = 8.2 Hz, 2H), 7.21 (d, *J* = 8.2 Hz, 2H), 5.35–5.34 (m, 1H), 5.01 (t, *J* = 5.5 Hz, 1H), 4.45 (t, *J* = 6.7 Hz, 3H), 3.21 (q, *J* = 6.0 Hz, 2H), 2.37 (s, 3H), 2.33–2.21 (m, 2H), 2.13 (app. quint, *J* = 6.5 Hz, 2H), 2.02–1.92 (m, 2H), 1.86–1.80 (m, 3H), 1.58–1.44 (m, 7H), 1.42–1.40 (m, 1H), 1.38–1.33 (m, 3H), 1.33–1.24 (m, 2H), 1.16–1.05 (m, 9H), 0.98 (s, 3H), 0.91 (d, *J* = 6.5 Hz, 3H), 0.86 (dd, *J* = 6.6, 1.8 Hz, 6H), 0.67 (s, 3H).

^{13}C NMR (100 MHz, $CDCl_3$): δ 156.5, 148.1, 139.9, 138.1, 129.6, 127.9, 125.7, 122.6, 119.7, 74.7, 56.8, 56.3, 50.1, 47.8, 42.4, 39.8, 39.6, 38.6, 37.9, 37.1, 36.6, 36.3, 35.9, 32.0, 31.9, 30.8, 28.3, 28.2, 28.1, 24.4, 23.9, 22.9, 22.7, 21.4, 21.1, 19.4, 18.8, 12.0.

IR ν_{max} (neat): 3350, 3331, 2935, 2867, 1693, 1532, 1465, 1437, 1366, 1253, 1033 cm^{-1}.

HRMS (ESI, $C_{40}H_{61}N_4O_2$, +ve mode): *m/z* $[M+H]^+$ calcd. 629.4789, found 629.4789.

N-(1-(1-(7-Hydroxy-2-oxo-2*H*-chromen-3-yl)-1*H*-1,2,3-triazol-4-yl)-1*H*-benzo[*d*]imidazol-6-yl)hept-6-ynamide (**S2**)

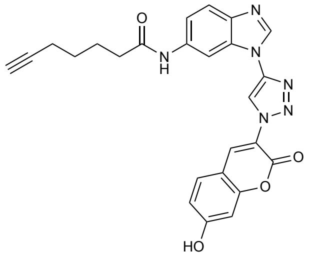

Prepared using General Procedure A (0.2 mmol scale). Yellow oil (91 mg, 75%). Purification on silica gel using 9/1 DCM/MeOH + 2% NEt_3.

^{1}H NMR (500 MHz, DMSO-d_6): δ 10.08 (br, 1H), 9.10 (br, 1H), 8.72 (br, 1H), 8.46 (br, 1H), 7.78 (br, 2H), 7.45 (br, 1H), 6.88 (br, 2H), 2.35 (br, 1H), 2.19 (br, 2H), 2.07 (br, 1H), 1.69 (br, 2H), 1.50 (br, 2H), 1.31 (br, 2H). One proton under D_2O peak, see COSY in Supplementary Figure S114.

^{13}C NMR could not be obtained due to relaxation issues.

IR ν_{max} (neat): 3447, 2251, 2125, 1656, 1054, 1026, 1007 cm^{-1}.

HRMS (ESI, $C_{25}H_{21}N_6O_4$, +ve mode): *m/z* $[M+H]^+$ calcd.469.1619, found 469.1620.

N-(1-(1-(7-Hydroxy-2-oxo-2*H*-chromen-3-yl)-1*H*-1,2,3-triazol-4-yl)-1*H*-benzo[*d*]imidazol-6-yl)-5-(1-((2*S*,3*S*,5*R*)-2-(hydroxymethyl)-5-(5-methyl-2,4-dioxo-3,4-dihydropyrimidin-1(2*H*)-yl)tetrahydrofuran-3-yl)-1*H*-1,2,3-triazol-4-yl)pentanamide (**21**)

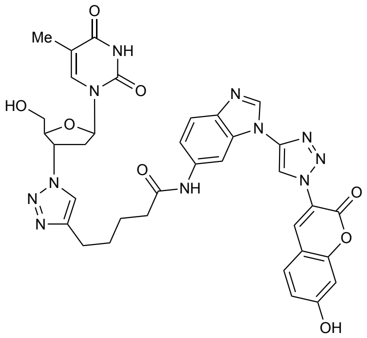

Prepared using General Procedure A (0.1 mmol scale). Brown oil (55 mg, 75%). Purification on silica gel using 9/1 DCM/MeOH + 2% NEt_3.

^{1}H NMR (500 MHz, DMSO-d_6): δ 11.35 (br, 1H), 10.08 (br, 1H), 9.11–8.73 (m, 2H), 8.53–8.31 (m, 1H), 8.05–7.67 (m, 3H), 7.55–7.45 (m, 1H), 7.88–7.83 (m, 2H), 6.49–6.40 (m, 1H), 5.52–5.30 (m, 2H), 4.38–4.19 (m, 1H), 3.84–3.68 (m, 2H), 3.61–3.60 (m, 1H), 2.86–2.62 (m, 4H), 2.39–2.38 (m, 2H), 1.83–1.80 (m, 3H), 1.78–1.66 (m, 4H). Two protons under D_2O peak, see COSY in Supplementary Figure S114.

^{13}C NMR could not be obtained due to relaxation issues.

IR ν_{max} (neat): 3443, 2251, 2125, 1682, 1612, 1054, 1026, 1007 cm^{-1}.

HRMS (ESI, $C_{35}H_{34}N_{11}O_8$, +ve mode): *m/z* $[M+H]^+$ calcd.736.2586, found 736.2595.

2-(4-(5-(4-(2-Fluoro-5-((4-oxo-3,4-dihydrophthalazin-1-yl)methyl)benzoyl)piperazin-1-yl)-5-oxopentyl)-1*H*-1,2,3-triazol-1-yl)-*N*-(4-fluorobenzyl)acetamide (**25**)

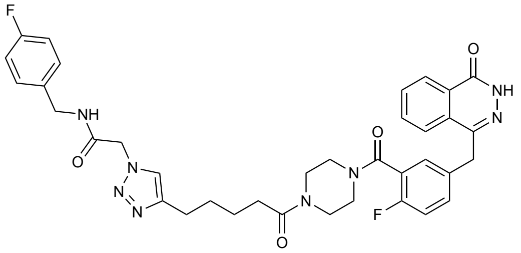

Prepared using General Procedure A (0.1 mmol scale at 3 mL/min). Yellow oil (59 mg, 86%). No purification needed.

^{1}H NMR (400 MHz, $CDCl_3$): δ 8.42–8.36 (m, 1H), 7.78–7.68 (m, 3H), 7.51 (d, *J* = 9.8 Hz, 1H), 7.31–7.30 (m, 2H), 7.25–7.23 (m, 1H), 7.17–7.13 (m, 2H), 7.06–6.99 (m, 2H), 6.95–6.87 (m, 2H), 5.06 (d, *J* = 16.8 Hz, 2H), 4.42 (d, *J* = 6.1 Hz, 1H), 4.36–4.34 (m, 2H), 4.28 (s, 2H), 4.03 (s, 1H), 3.72–3.67 (m, 2H), 3.52–3.49 (m, 1H), 3.46–3.43 (m, 1H), 3.33–3.23 (m, 2H), 2.75–2.69 (m, 2H), 2.36–2.32 (m, 2H), 1.73–1.64 (m, 4H);

^{19}F NMR (376 MHz, $CDCl_3$) δ -114.5, -114.7, -117.8, -118.0.

^{13}C NMR could not be obtained due to relaxation issues.[2]

IR ν_{max} (neat): 3296, 3071, 2926, 2865, 1638, 1511, 1437, 1225 cm^{-1}.

HRMS (ESI, $C_{36}H_{37}F_2N_8O_4$, +ve mode): *m/z* $[M+H]^+$ calcd.683.2900, found 683.2891.

2-Cyanoethyl (4-((triisopropylsilyl)ethynyl)phenethyl) diisopropylphosphoramidite (**S3**)

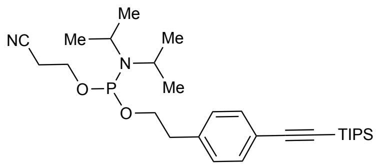

N,*N*-Diisopropylethylamine (1.6 mL, 9.4 mmol, 9 equiv.) and 2-cyanoethyl diisopropylchlorophosphoramidite (0.3 mL, 1.3 mmol, 1.2 equiv.) were added to a solution of 2-(4-((triisopropylsilyl)ethynyl)phenyl)ethan-1-ol (0.3 g, 1.0 mmol, 1 equiv.) in dry THF (10 mL) under argon atmosphere. The reaction was stirred at room temperature for 1.5 h, and then partitioned between $CHCl_3$ (50 mL) and saturated $NaHCO_3$ (50 mL). The organic layer was wash with brine (3 × 20 mL), dried over Na_2SO_4, and concentrated under reduced pressure. The resulting residue was purified by flash chromatography (silica gel, *n*-hexane/EtOAc, 3/1) to provide the desired product as a colorless oil (0.4 g, 76%).

^{1}H NMR (500 MHz, $CDCl_3$): δ 7.39 (d, *J* = 7.7 Hz, 2H), 7.16 (d, *J* = 7.7 Hz, 2H), 3.87–3.73 (m, 4H), 3.61–3.55 (m, 2H), 2.91 (d, *J* = 7.2 Hz, 2H), 2.58 (d, *J* = 6.4 Hz, 2H), 1.20–1.17 (m, 12H), 1.15–1.12 (m, 21H).

^{13}C NMR (125 MHz, $CDCl_3$): δ 139.3, 132.2, 129.1, 121.7, 117.8, 107.3, 90.2, 64.2, 58.5, 58.4, 43.3, 43.2, 37.9, 37.8, 24.8, 24.7, 24.6, 24.5, 18.8, 11.5.

^{31}P NMR (202 MHz, $CDCl_3$): δ 147.5.

IR ν_{max} (neat): 2961, 2939, 2863, 2249, 2155, 1463, 1364, 1184, 1026 cm^{-1}.

HRMS (ESI, $C_{28}H_{47}N_2O_2PSi$, +ve mode): *m/z* $[M+H]^+$ calcd. 503.3217, found 503.3209.

1. Characterization of ODNs

Alkyne modified ODN_{1-3} were prepared using General Procedure B, purified by preparative RP-HPLC and characterized by MALDI-MS and analytical RP-HPLC.

ODN1: $C_{130}H_{166}N_{24}O_{85}P_{12}$ MW 3796.53 g/mol. **MALDI-TOF** (+ve mode; matrix 3-hydroxy--picolinic acid) *m/z* $[M+H]^+$ found 3797.66. **RP-HPLC** R_T= 20.5 min.

ODN2: $C_{130}H_{165}N_{27}O_{84}P_{12}$ MW 3821.54 g/mol. **MALDI-TOF** (+ve mode; matrix 3-hydroxy--picolinic acid) *m/z* $[M+H]^+$ found 3821.57. **RP-HPLC** R_T= 19.1 min.

ODN3: $C_{127}H_{158}N_{42}O_{75}P_{12}$ MW 3844.57 g/mol. **MALDI-TOF** (+ve mode; matrix 3-hydroxy--picolinic acid) *m/z* $[M+H]^+$ found 3844.72. **RP-HPLC** R_T= 16.3 min.

Triazole ODN_{4-6} were prepared using General Procedure C and characterized by MALDI-MS and analytical RP-HPLC.

ODN4 (**26a**): $C_{145}H_{185}N_{29}O_{87}P_{12}S$ MW 4129.94 g/mol. **MALDI-TOF** (+ve mode; matrix 3-hydroxypicolinic acid) *m/z* $[M+H]^+$ found 4130.83. **RP-HPLC** R_T= 25.7 min.

ODN5 (**26b**): $C_{145}H_{184}N_{32}O_{86}P_{12}S$ MW 4154.95 g/mol. **MALDI-TOF** (+ve mode; matrix 3-hydroxypicolinic acid) *m/z* $[M+H]^+$ found 4155.26. **RP-HPLC** R_T= 25.6 min.

ODN6 (**26c**): $C_{142}H_{177}N_{47}O_{77}P_{12}S$ MW 4177.96 g/mol. **MALDI-TOF** (+ve mode; matrix 3-hydroxypicolinic acid) *m/z* $[M+H]^+$ found 4178.88. **RP-HPLC** R_T= 23.4 min.

2. Characterization of Peptides

Alkyne modified peptides **P1-5** were prepared using General Procedure D, purified by preparative RP-HPLC and characterized by MALDI-MS and analytical RP-HPLC.

P1: $C_{67}H_{126}N_{26}O_{12}$ MW 1487.92 g/mol. **MALDI-TOF** (+ve mode; matrix α-cyano-4-hydroxycinnamic acid) *m/z* $[M+H]^+$ found 1487.96. **HRMS** (+ve mode) *m/z* $[M/2+H]^+$ calcd. 744.5097, found 744.5083. **RP-HPLC** R_T= 19.7 min.

P2: $C_{70}H_{131}N_{27}O_{13}S$ MW 1591.05 g/mol. **MALDI-TOF** (+ve mode; matrix α-cyano-4-hydroxycinnamic acid) *m/z* $[M+H]^+$ found 1590.97. **HRMS** (+ve mode) *m/z* $[M/2+H]^+$ calcd. 796.0143, found 796.0129. **RP-HPLC** R_T= 21.0 min.

P3: $C_{72}H_{135}N_{27}O_{13}S$ MW 1619.11 g/mol. **MALDI-TOF** (+ve mode; matrix α-cyano-4-hydroxycinnamic acid) *m/z* $[M+H]^+$ found 1619.01. **HRMS** (+ve mode) *m/z* $[M/2+H]^+$ calcd. 810.0299, found 810.0282. **RP-HPLC** R_T= 22.5 min.

P4: $C_{73}H_{133}N_{29}O_{13}$ MW 1625.06 g/mol. **MALDI-TOF** (+ve mode; matrix α-cyano-4-hydroxycinnamic acid) *m/z* $[M+H]^+$ found 1625.04. **HRMS** (+ve mode) *m/z* $[M/2+H]^+$ calcd. 813.0392, found 813.0376. **RP-HPLC** R_T= 18.1 min.

P5: $C_{76}H_{135}N_{27}O_{14}$ MW 1651.09 g/mol. **MALDI-TOF** (+ve mode; matrix α-cyano-4-hydroxycinnamic acid) *m/z* $[M+H]^+$ found 1651.02. **HRMS** (+ve mode) *m/z* $[M/2+H]^+$ calcd. 826.0414, found 826.0395. **RP-HPLC** R_T= 21.7 min.

Triazole peptides **27a-e** were prepared using General Procedure C and characterized by MALDI-MS and analytical RP-HPLC.

27a: $C_{82}H_{145}N_{31}O_{14}S$ MW 1821.33 g/mol. **MALDI-TOF** (+ve mode; matrix α-cyano-4-hydroxycinnamic acid) *m/z* $[M+H]^+$ found 1821.09. **HRMS** (+ve mode) *m/z* $[M/2+H]^+$ calcd. 911.0727, found 911.5724. **RP-HPLC** R_T= 22.5 min.

27b: $C_{85}H_{150}N_{32}O_{15}S_2$ MW 1924.46 g/mol. **MALDI-TOF** (+ve mode; matrix α-cyano-4-hydroxycinnamic acid) *m/z* $[M+H]^+$ found 1924.19. **HRMS** (+ve mode) *m/z* $[M/2+H]^+$ calcd. 962.5772, found 962.5773. **RP-HPLC** R_T= 23.6 min.

27c: $C_{87}H_{154}N_{32}O_{15}S_2$ MW 1952.52 g/mol. **MALDI-TOF** (+ve mode; matrix α-cyano-4-hydroxycinnamic acid) *m/z* $[M+H]^+$ found 1952.21. **HRMS** (+ve mode) *m/z* $[M/2+H]^+$ calcd. 976.5929, found 977.0945. **RP-HPLC** R_T= 24.9 min.

27d: $C_{88}H_{152}N_{34}O_{15}S$ MW 1958.47 g/mol. **MALDI-TOF** (+ve mode; matrix α-cyano-4-hydroxycinnamic acid) *m/z* $[M+H]^+$ found 1958.17. **HRMS** (+ve mode) *m/z* $[M/2+H]^+$ calcd. 979.6021, found 980.1034. **RP-HPLC** R_T= 21.0 min.

27c: $C_{91}H_{154}N_{32}O_{16}S$ MW 1984.50 g/mol. **MALDI-TOF** (+ve mode; matrix α-cyano-4-hydroxycinnamic acid) *m/z* $[M+H]^+$ found 1984.17. **HRMS** (+ve mode) *m/z* $[M/2+H]^+$ calcd. 992.6043, found 993.1051. **RP-HPLC** R_T= 24.1 min.

Peptide-PMO Conjugate Synthesis and Characterization Data

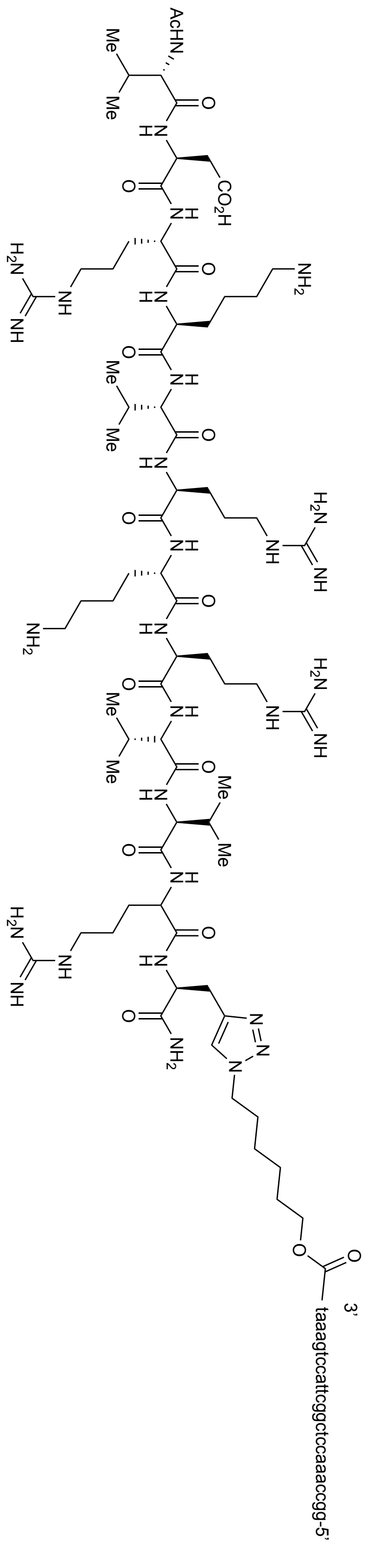

ApoE-PMO (**28**)[22]

PMO azide (10 μL, 10 nmol, 1 equiv), peptide **P1** (20 μL, 1 00 nmol, 10 equiv) and 3-methylbenzo[*d*]thiazol-3-ium **29** (1.0 mg, 1 000 nmol, 100 equiv.) were dissolved in 50 μL of MeCN/H_2O (5/1). CuAAC reaction was carried out in a commercial chemical flow reactor equipped with a 2 mL copper reactor (easy-Scholar from Vapourtec) at a flow rate of 1 mL/min at 37 °C. The reaction mixture was collected after 15 cycles through the reactor and lyophilised. The residue was suspended in H_2O and subjected to a round of spin concentration using spin concentration Vivaspin 500 (Sartorius) for 30 min at 13.5 rpm, affording the expected conjugate **28** with 60% yield. Yield was calculated by measuring absorbance of the product, purity was assessed by analytical HPLC (Phenomenex C18, 250 × 4.6 mm) at a flow rate of 1 mL/min, gradient 5–20% MeCN/0.1% TFA in 35 min.

28: MS: $C_{369}H_{600}N_{175}O_{113}P_{25}$ MW 10070.32 g/mol. **MALDI-TOF** (+ve mode; matrix sinapinic acid) *m/z* $[M+H]^+$ found 10070.49. **RP-HPLC** R_T= 7.81 min.

Supplementary Figures 7-125

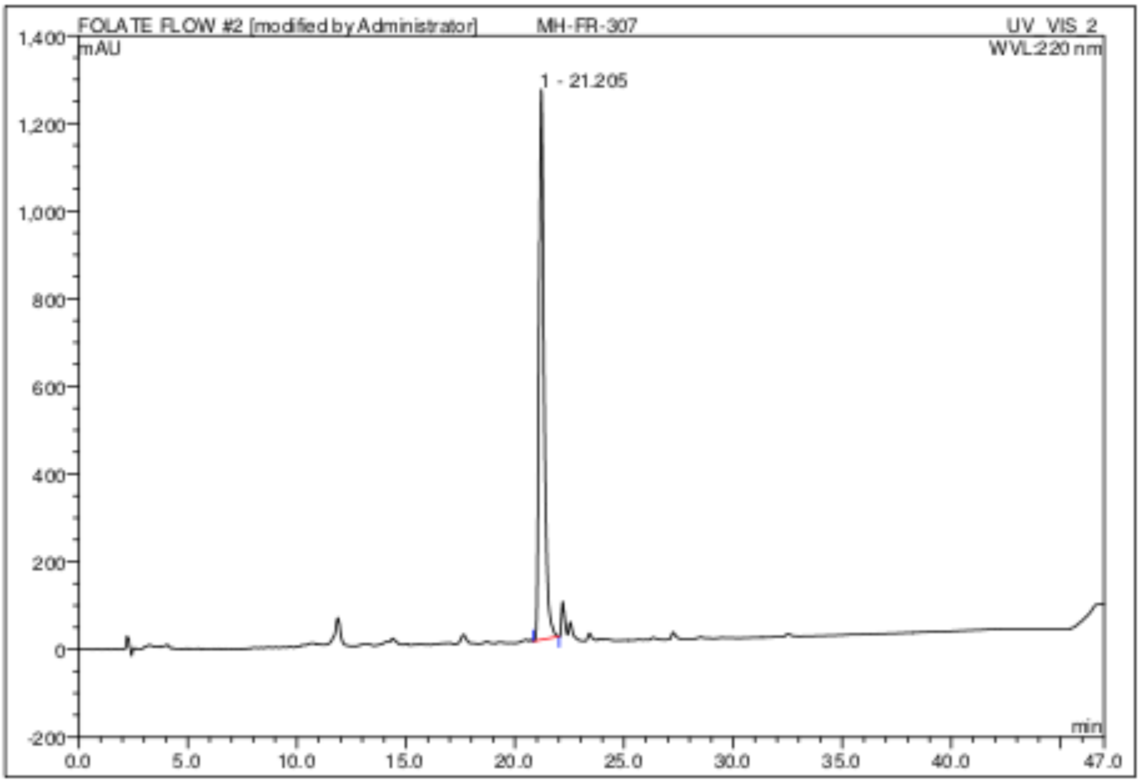

No.	Ret.Time min	Peak Name	Height mAU	Area mAU*min	Rel.Area %	Amount	Type
1	21.21	n.a.	1254.774	334.457	100.00	n.a.	BMB*
Total:			1254.774	334.457	100.00	0.000	

Supplementary Figure 7. RP-HPLC trace of **16a**.

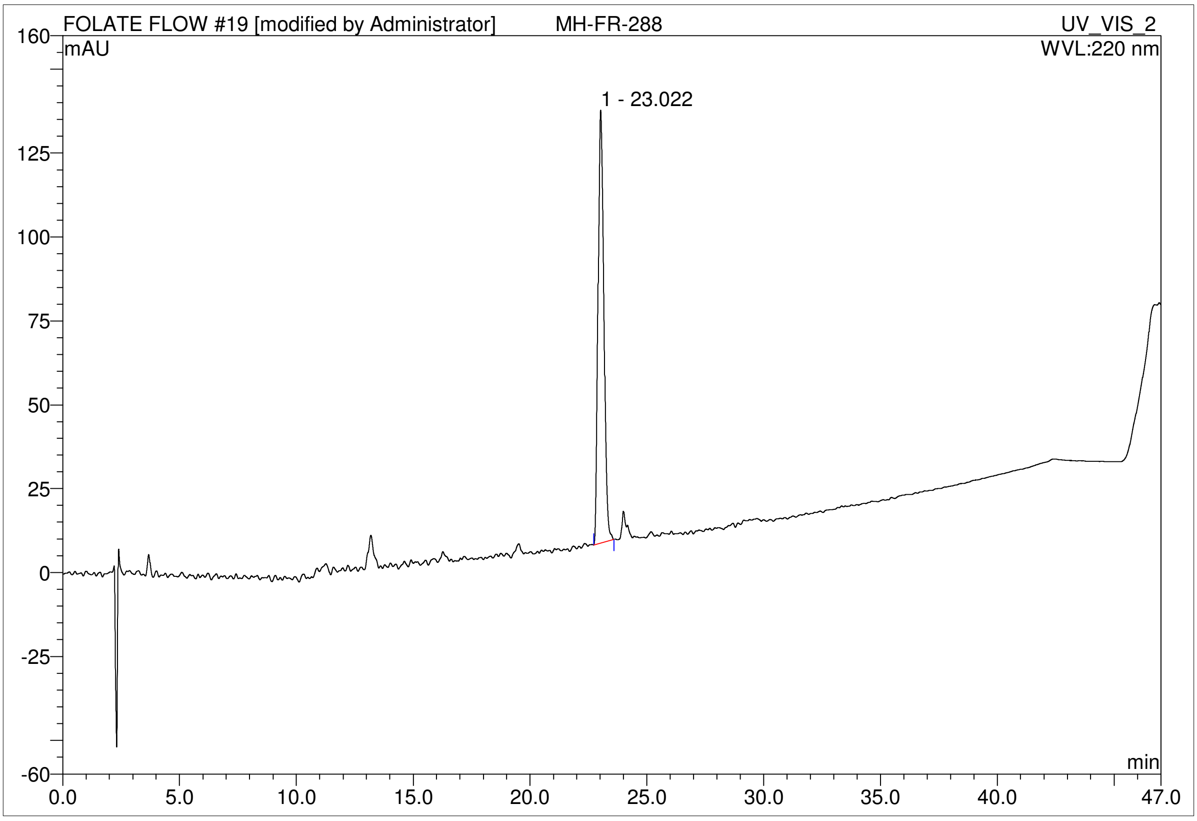

No.	Ret.Time min	Peak Name	Height mAU	Area mAU*min	Rel.Area %	Amount	Type
1	23.02	n.a.	129.018	37.328	100.00	n.a.	BMB
Total:			129.018	37.328	100.00	0.000	

Supplementary Figure 8. RP-HPLC trace of **16b**.

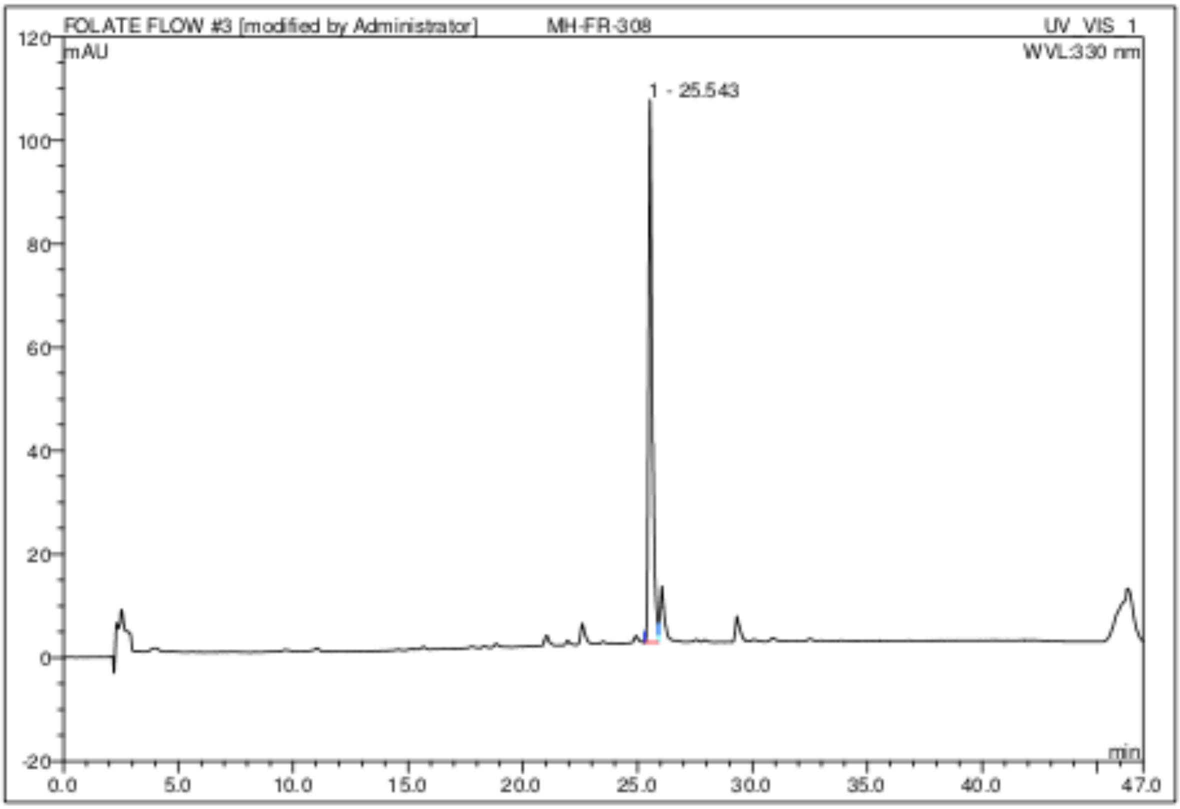

No.	Ret.Time min	Peak Name	Height mAU	Area mAU*min	Rel.Area %	Amount	Type
1	25.54	n.a.	104.943	19.818	100.00	n.a.	BM *
Total:			104.943	19.818	100.00	0.000	

Supplementary Figure 9. RP-HPLC trace of **16c**.

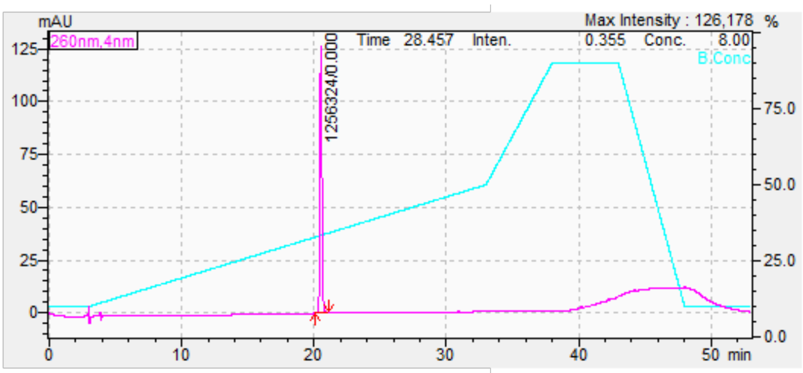

Peak#	Ret. Time	Area	Area%
1	20.513	1256324	100.000
Total		1256324	100.000

Supplementary Figure 10. RP-HPLC trace of ODN1.

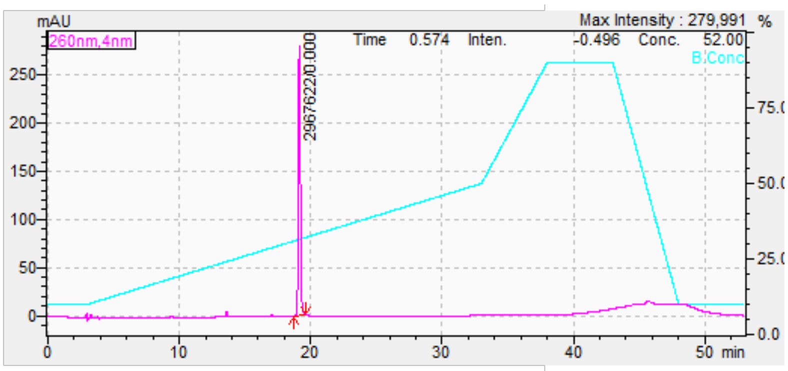

Peak#	Ret. Time	Area	Area%
1	19.130	2967622	100.000
Total		2967622	100.000

Supplementary Figure 11. RP-HPLC trace of ODN2.

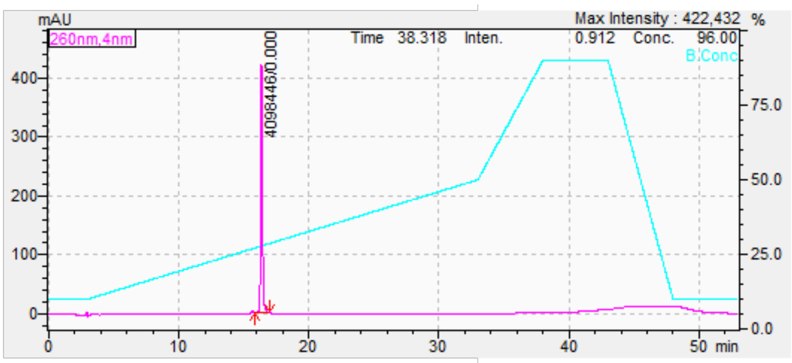

Peak#	Ret. Time	Area	Area%
1	16.346	4098446	100.000
Total		4098446	100.000

Supplementary Figure 12. RP-HPLC trace of ODN3.

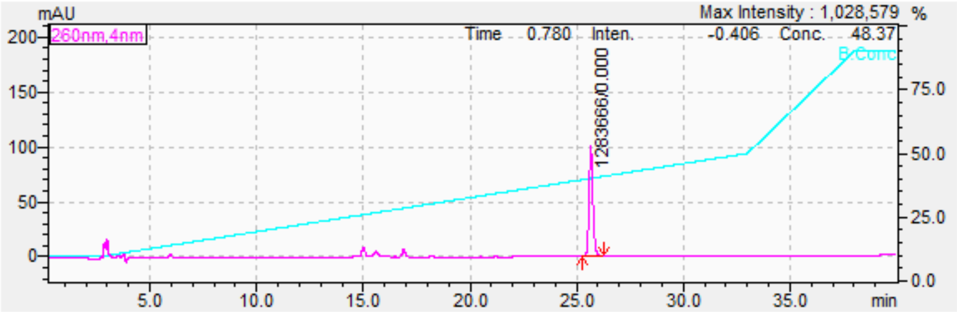

Peak#	Ret. Time	Area	Area%
1	25.661	1283666	100.000
Total		1283666	100.000

Supplementary Figure 13. RP-HPLC trace of ODN4 (**26a**).

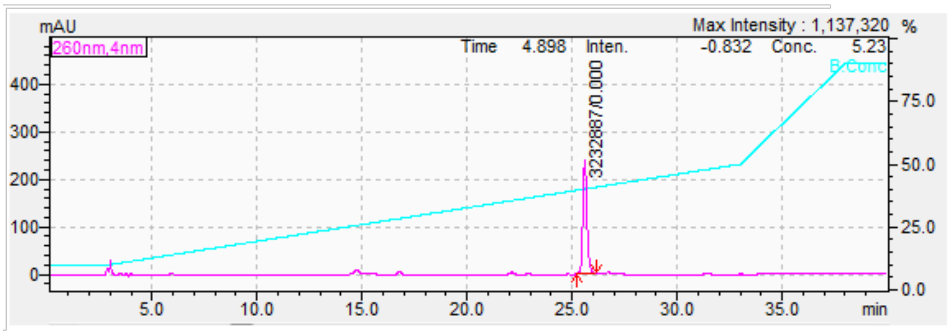

Peak#	Ret. Time	Area	Area%
1	25.604	3232887	100.000
Total		3232887	100.000

Supplementary Figure 14. RP-HPLC trace of ODN5 (**26b**).

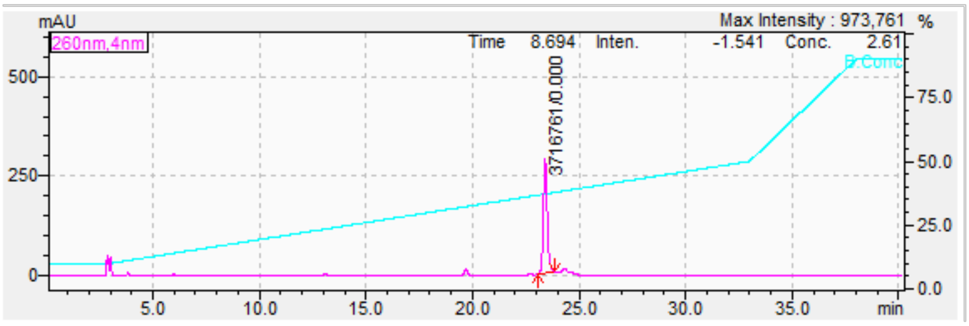

Peak#	Ret. Time	Area	Area%
1	23.408	3716761	100.000
Total		3716761	100.000

Supplementary Figure 15. RP-HPLC trace of ODN6 (**26c**).

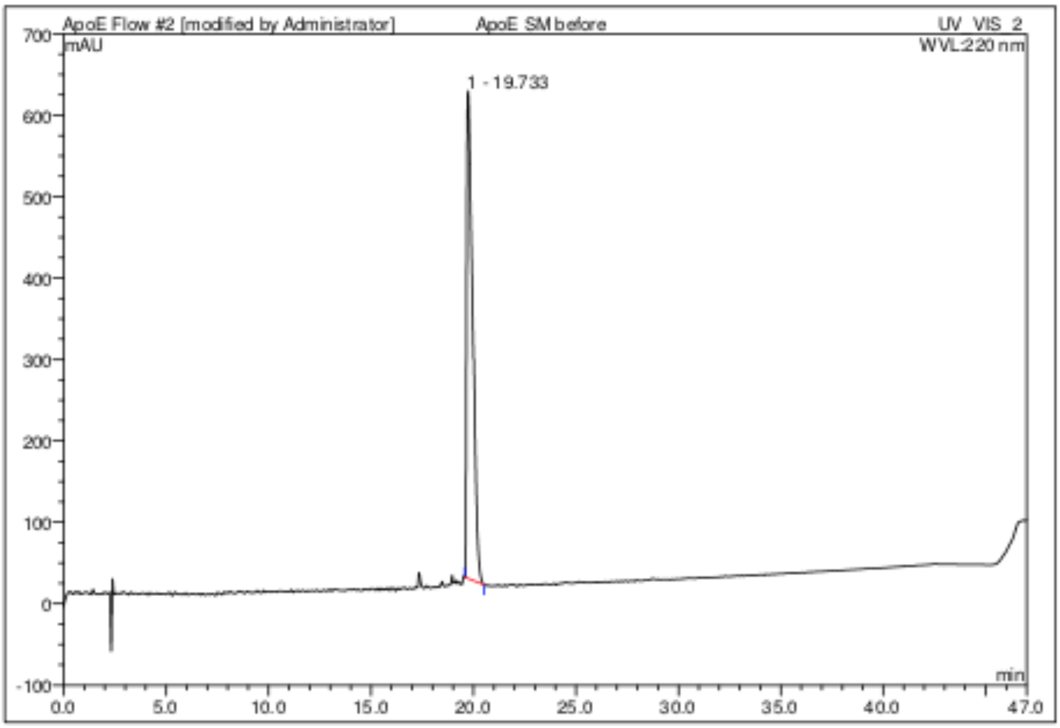

No.	Ret.Time min	Peak Name	Height mAU	Area mAU*min	Rel.Area %	Amount	Type
1	19.73	n.a.	598.801	200.853	100.00	n.a.	BMB
Total:			598.801	200.853	100.00	0.000	

Supplementary Figure 16. RP-HPLC trace of **P1**.

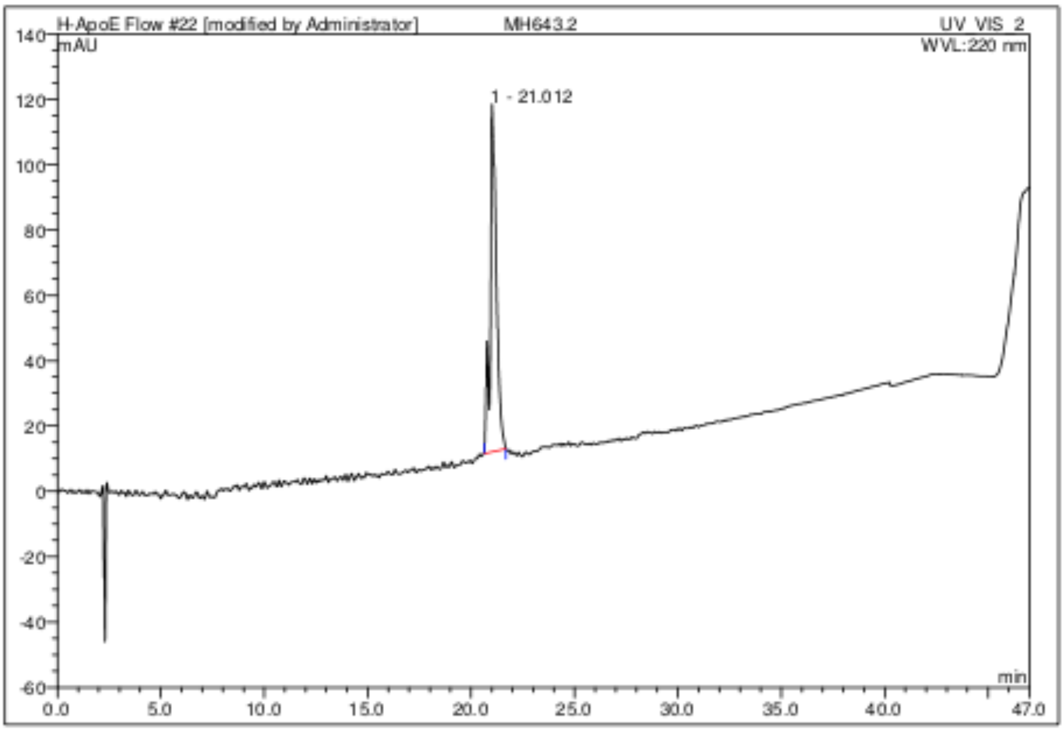

No.	Ret.Time min	Peak Name	Height mAU	Area mAU*min	Rel.Area %	Amount	Type
1	21.01	n.a.	106.371	36.292	100.00	n.a.	BMB*
Total:			106.371	36.292	100.00	0.000	

Supplementary Figure 17. RP-HPLC trace of **P2**.

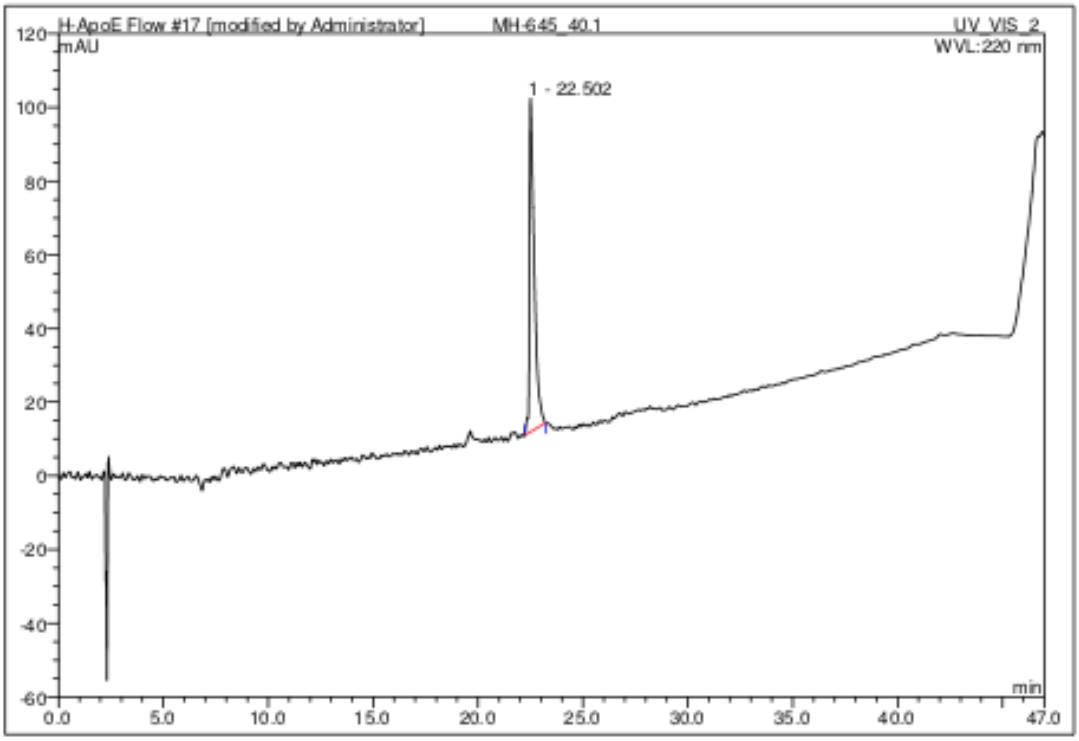

No.	Ret.Time min	Peak Name	Height mAU	Area mAU*min	Rel.Area %	Amount	Type
1	22.50	n.a.	90.477	24.233	100.00	n.a.	BMB*
Total:			90.477	24.233	100.00	0.000	

Supplementary Figure 18. RP-HPLC trace of **P3**.

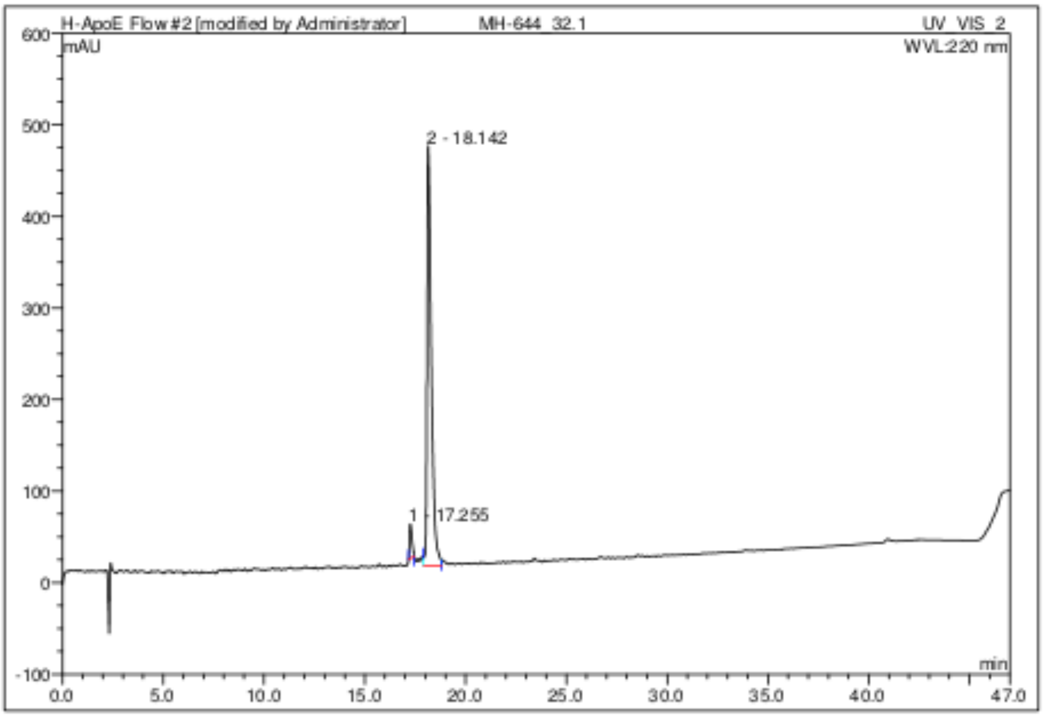

No.	Ret.Time min	Peak Name	Height mAU	Area mAU*min	Rel.Area %	Amount	Type
1	17.26	n.a.	37.648	5.155	4.32	n.a.	BMB*
2	18.14	n.a.	458.553	114.169	95.68	n.a.	M *
Total:			496.201	119.325	100.00	0.000	

Supplementary Figure 19. RP-HPLC trace of **P4**.

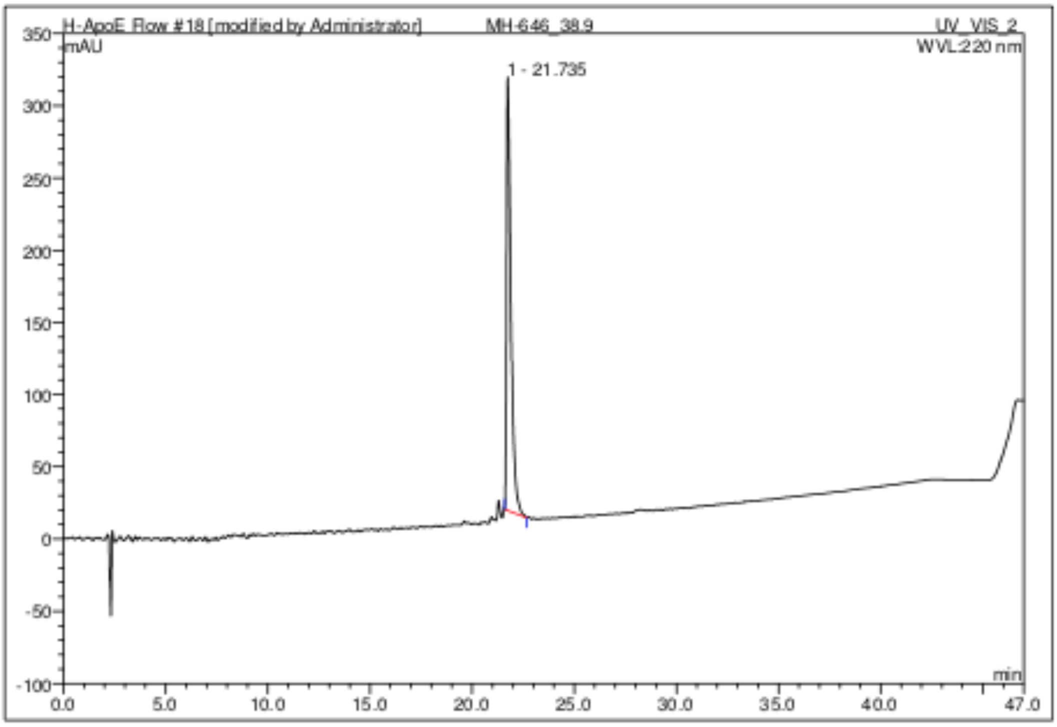

No.	Ret.Time min	Peak Name	Height mAU	Area mAU*min	Rel.Area %	Amount	Type
1	21.74	n.a.	300.133	75.007	100.00	n.a.	BMB
Total:			300.133	75.007	100.00	0.000	

Supplementary Figure 20. RP-HPLC trace of **P5**.

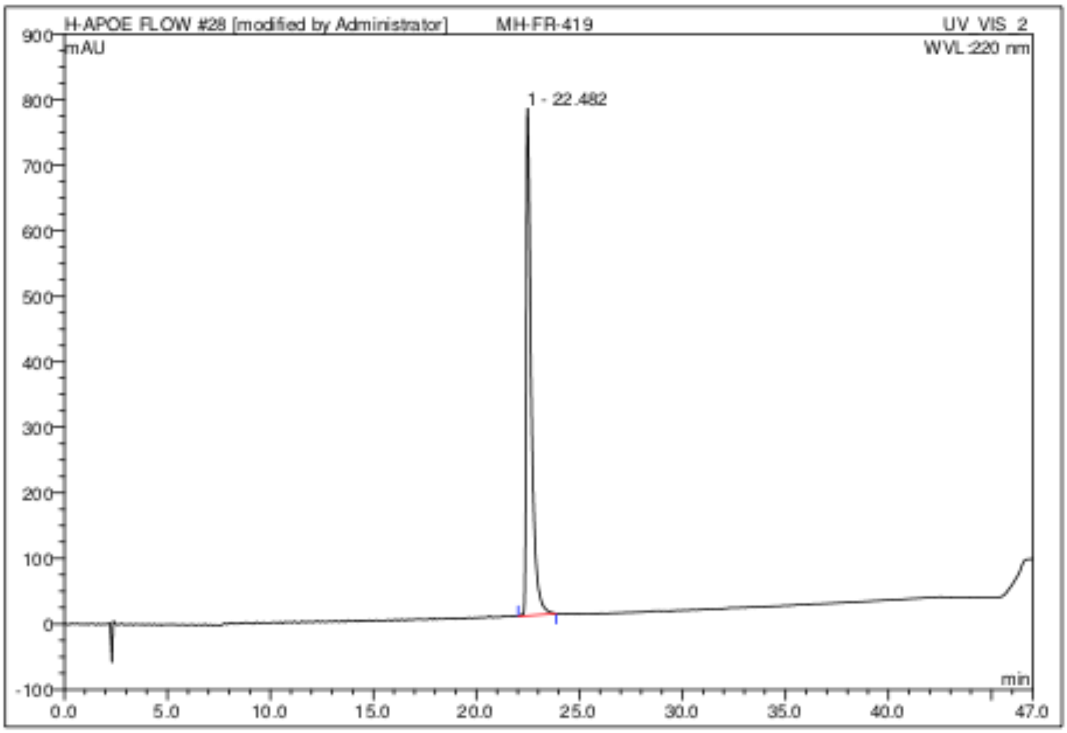

No.	Ret.Time min	Peak Name	Height mAU	Area mAU*min	Rel.Area %	Amount	Type
1	22.48	n.a.	773.735	216.391	100.00	n.a.	BMB
Total:			773.735	216.391	100.00	0.000	

Supplementary Figure 21. RP-HPLC trace of **27a**.

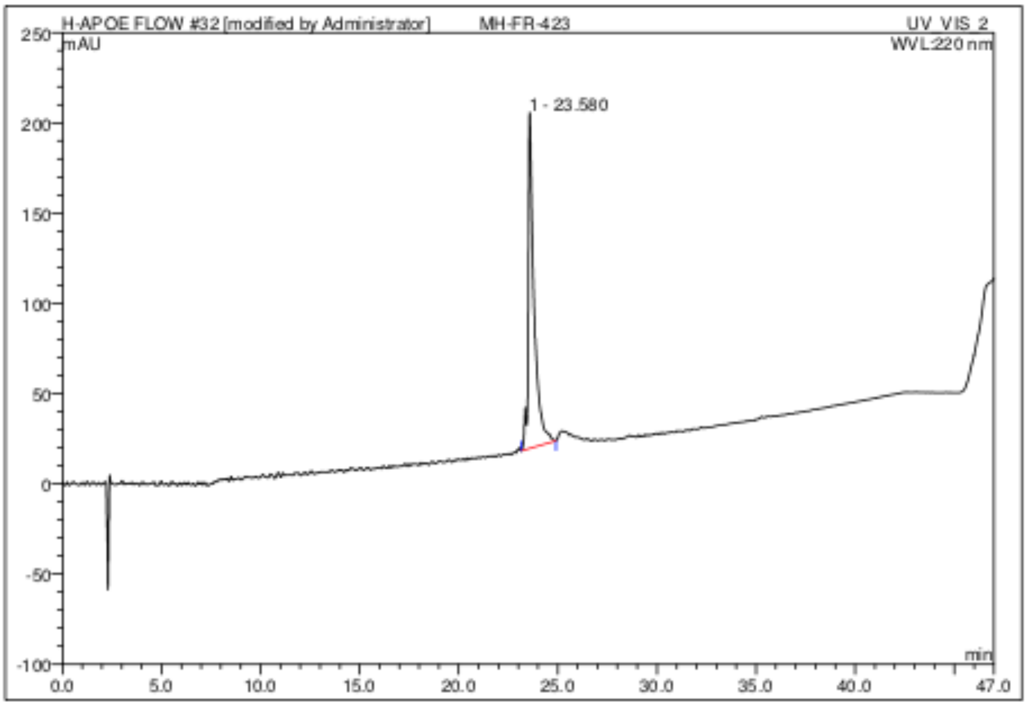

No.	Ret.Time min	Peak Name	Height mAU	Area mAU*min	Rel.Area %	Amount	Type
1	23.58	n.a.	186.322	61.340	100.00	n.a.	BMB*
Total:			186.322	61.340	100.00	0.000	

Supplementary Figure 22. RP-HPLC trace of **27b**.

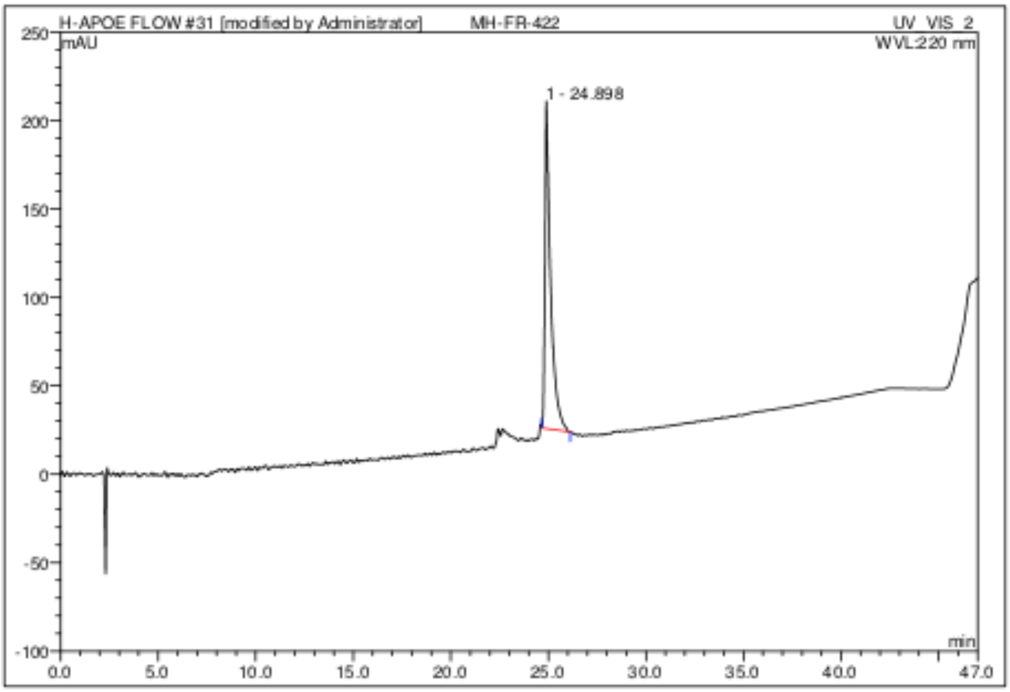

No.	Ret.Time min	Peak Name	Height mAU	Area mAU*min	Rel.Area %	Amount	Type
1	24.90	n.a.	185.083	61.579	100.00	n.a.	BMB
Total:			185.083	61.579	100.00	0.000	

Supplementary Figure 23. RP-HPLC trace of **27c**.

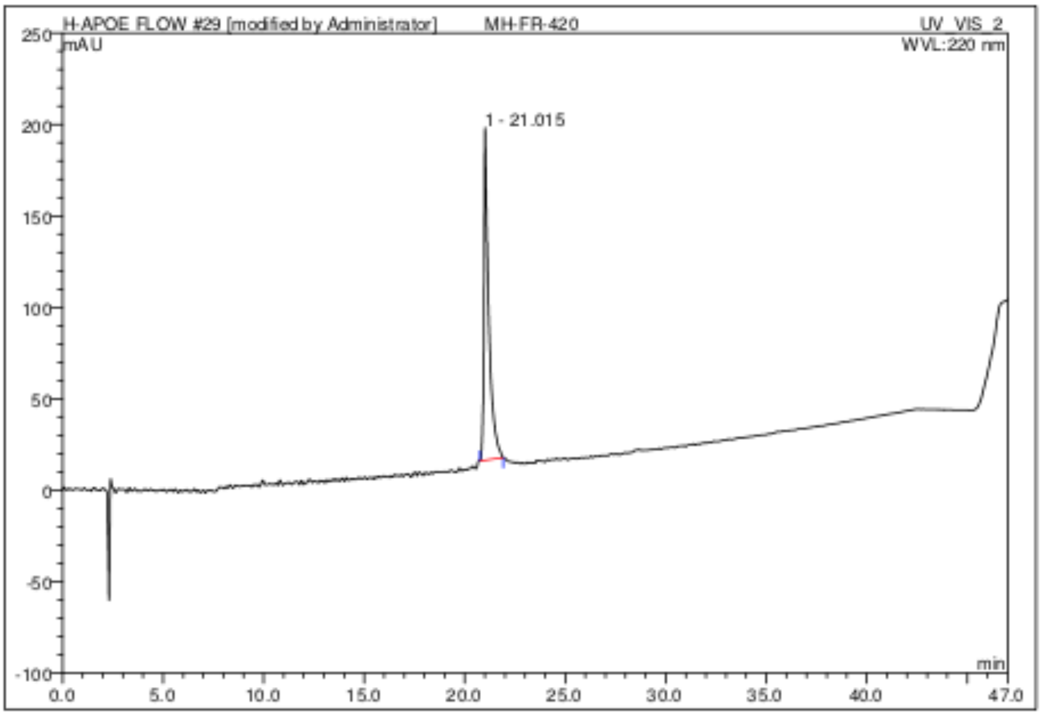

No.	Ret.Time min	Peak Name	Height mAU	Area mAU*min	Rel.Area %	Amount	Type
1	21.02	n.a.	181.727	49.083	100.00	n.a.	BMB
Total:			181.727	49.083	100.00	0.000	

Supplementary Figure 24. RP-HPLC trace of **27d**.

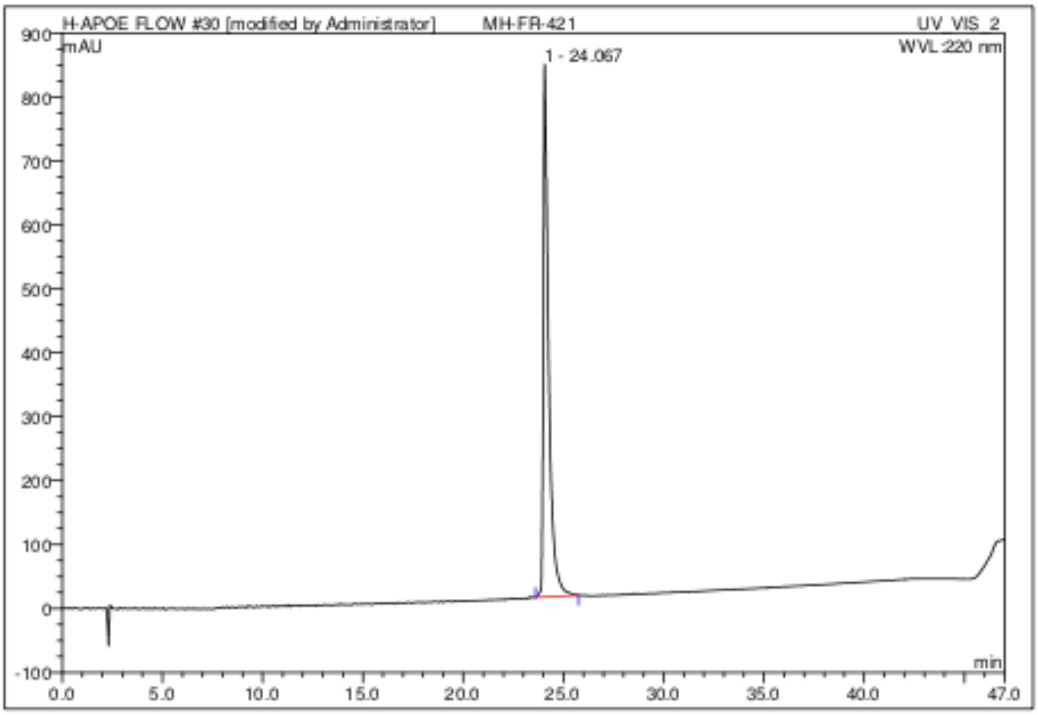

No.	Ret.Time min	Peak Name	Height mAU	Area mAU*min	Rel.Area %	Amount	Type
1	24.07	n.a.	833.434	253.707	100.00	n.a.	BMB
Total:			833.434	253.707	100.00	0.000	

Supplementary Figure 25. RP-HPLC trace of **27e**.

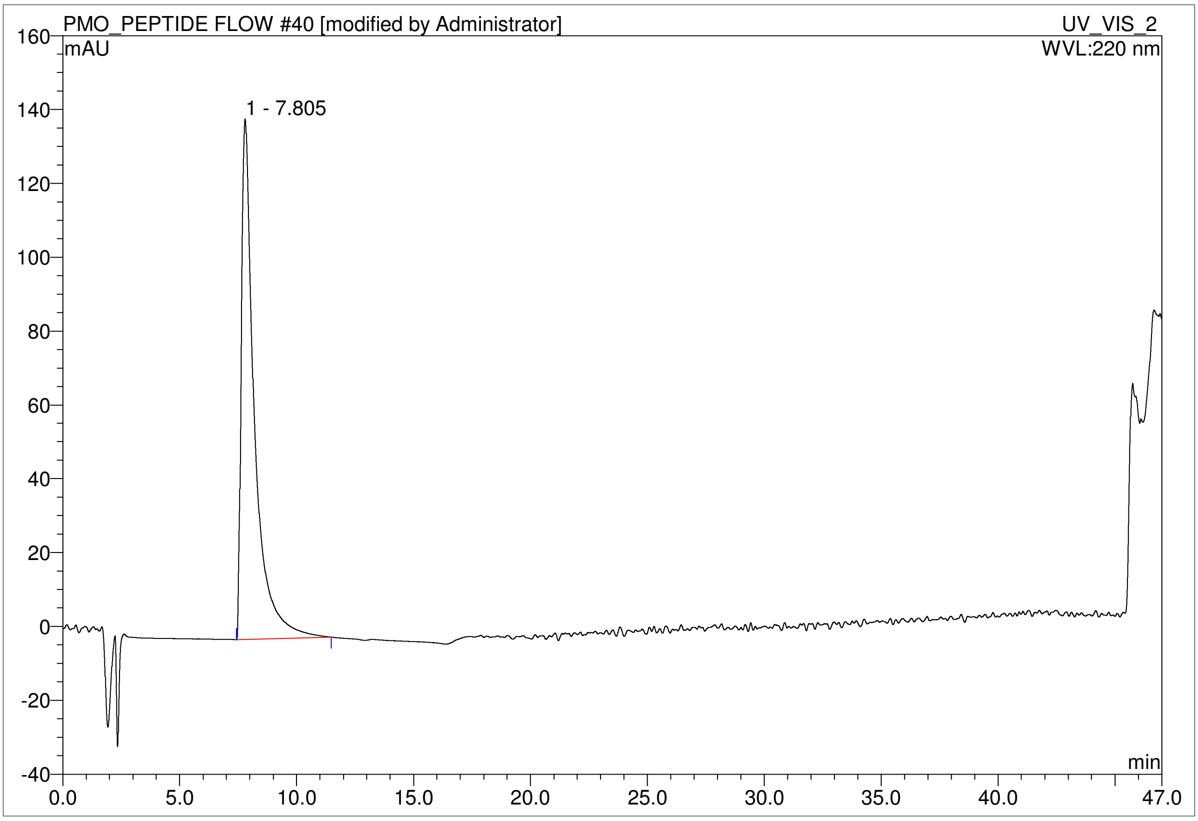

No.	Ret.Time min	Peak Name	Height mAU	Area mAU*min	Rel.Area %	Amount	Type
1	7.81	n.a.	140.949	92.197	100.00	n.a.	BMB*
Total:			140.949	92.197	100.00	0.000	

Supplementary Figure 26. RP-HPLC trace of **28**.

Supplementary Figure 27

MALDI-TOF Analysis of Peptide-PMO Conjugate **28**

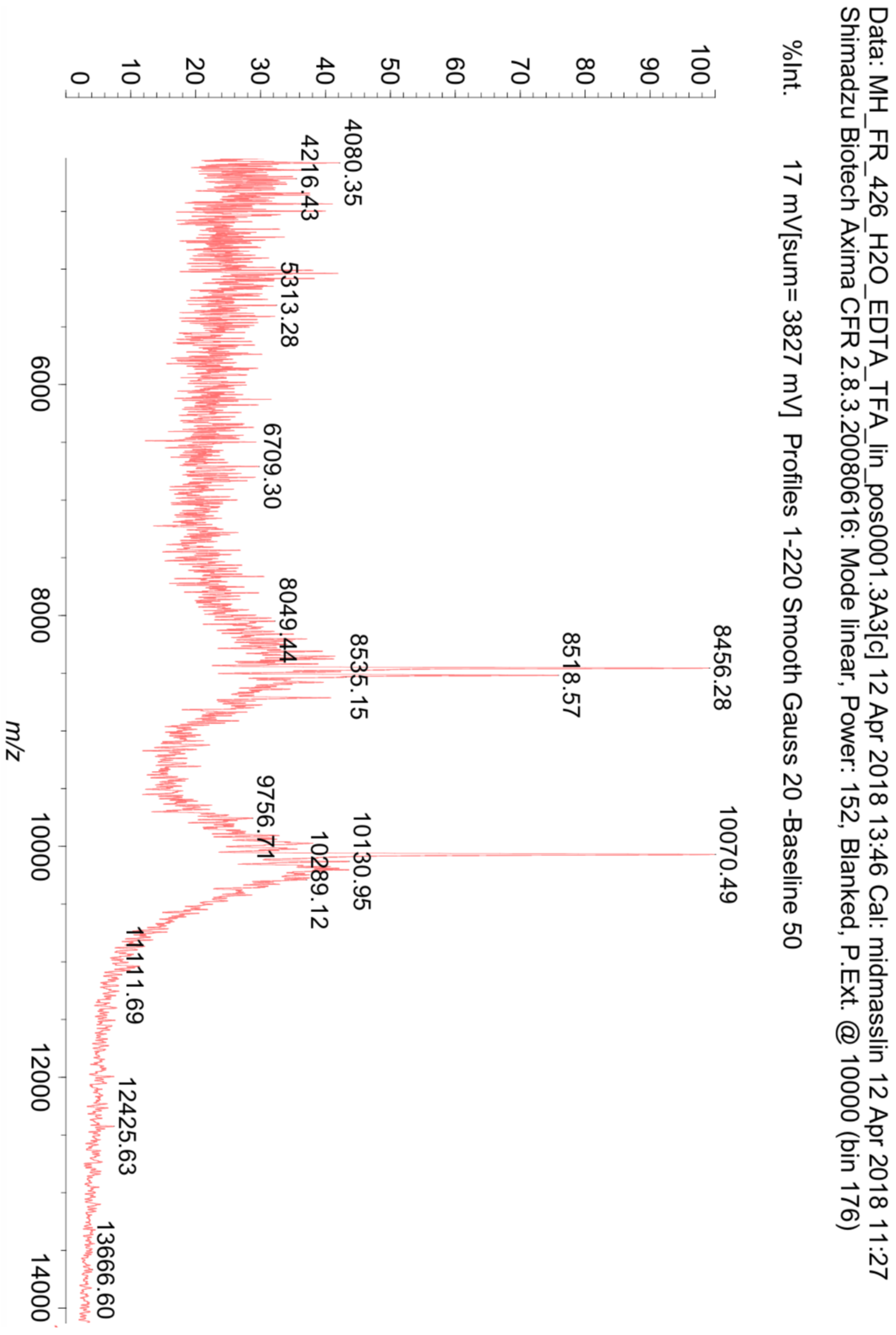

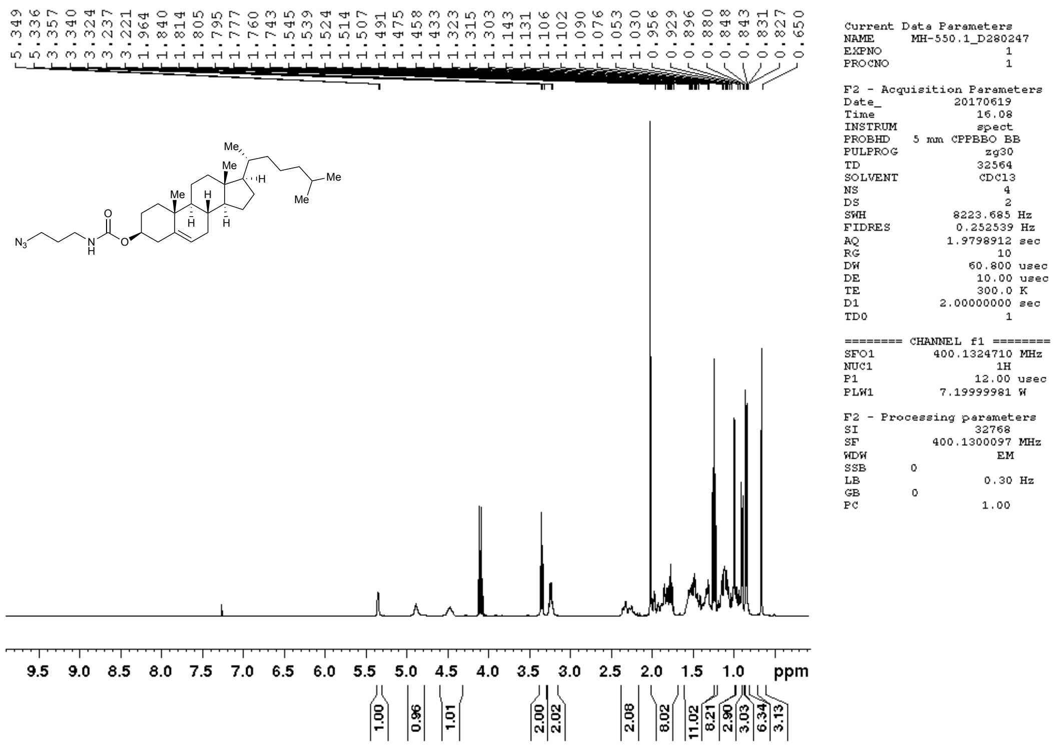

Supplementary Figure 28. ^{1}H NMR spectrum of **1**.

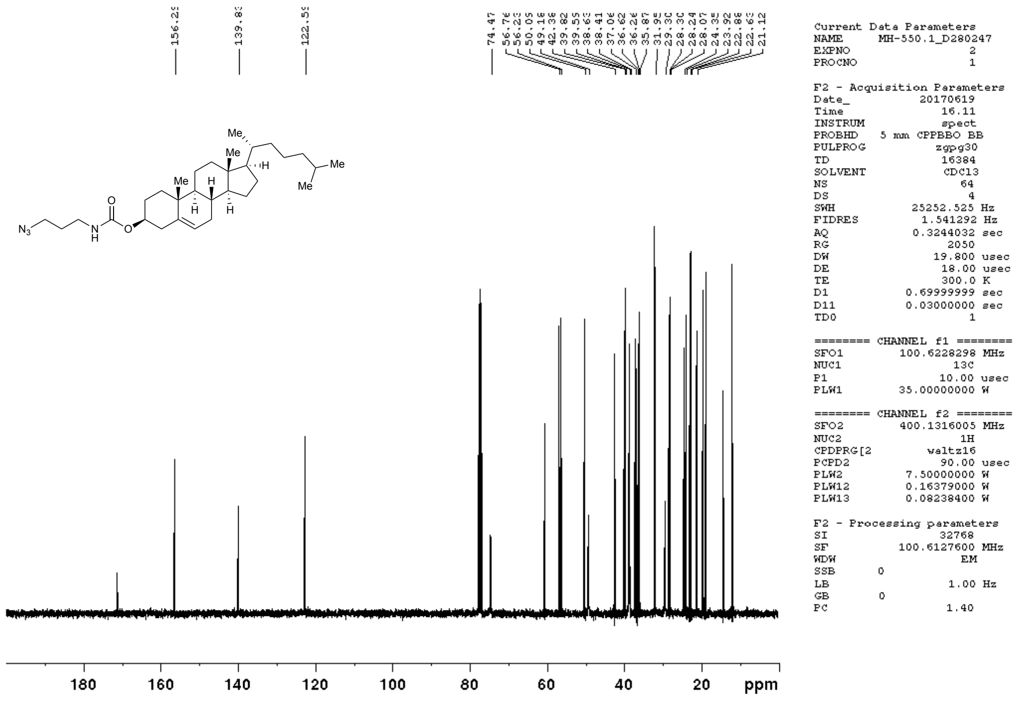

Supplementary Figure 29. ^{13}C NMR spectrum of **1**.

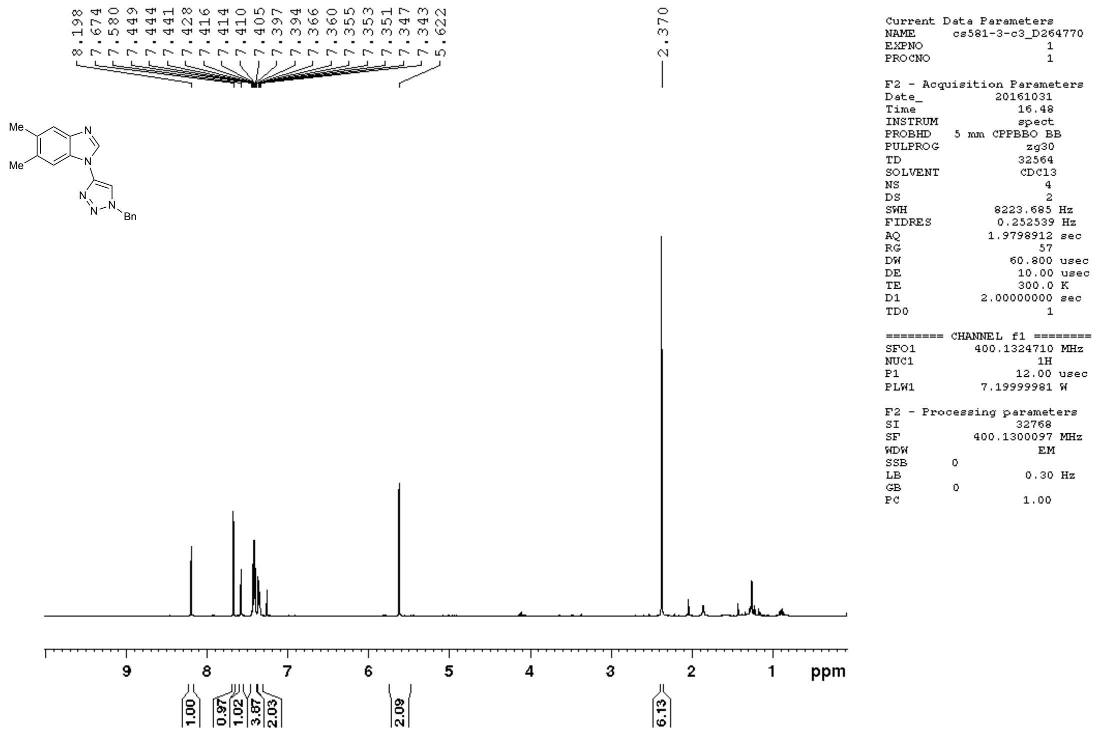

Supplementary Figure 30. ^{1}H NMR spectrum of **a**.

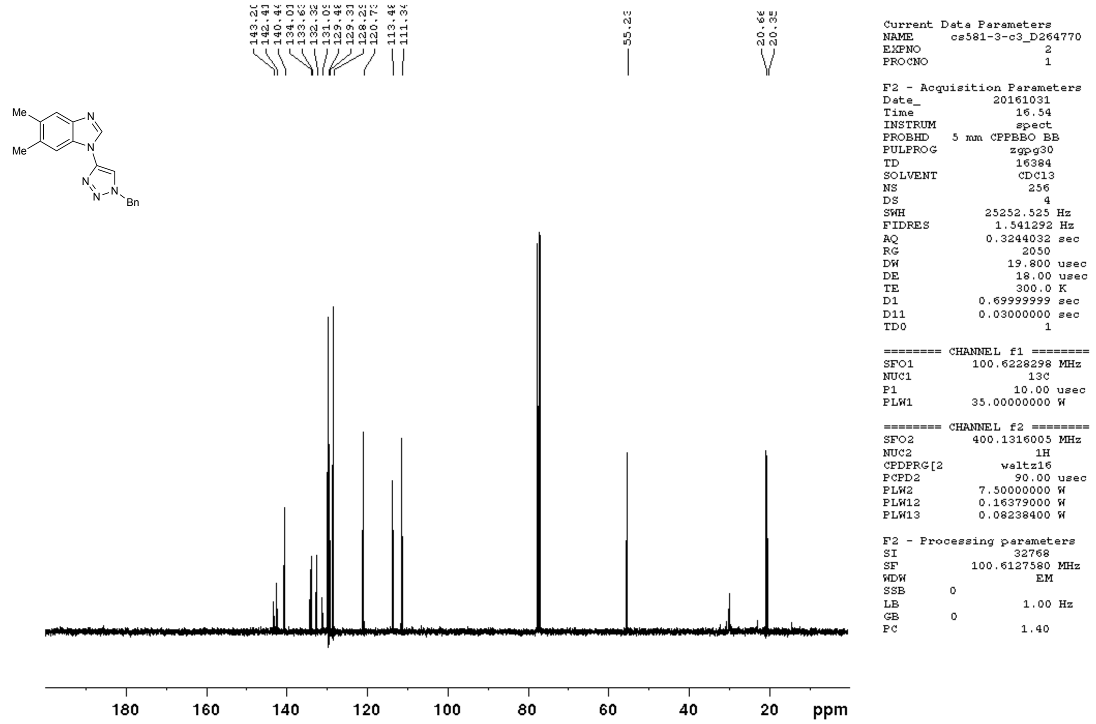

Supplementary Figure 31. ^{13}C NMR spectrum of **3a**.

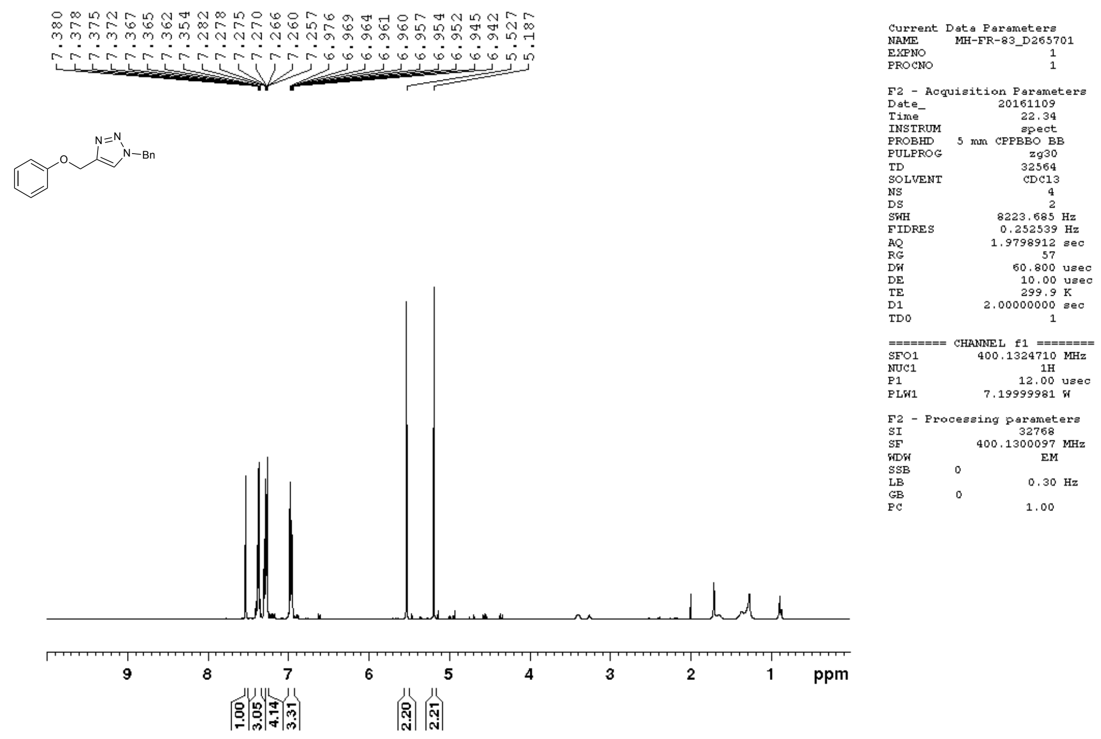

Supplementary Figure 32. ^{1}H NMR spectrum of **3b**.

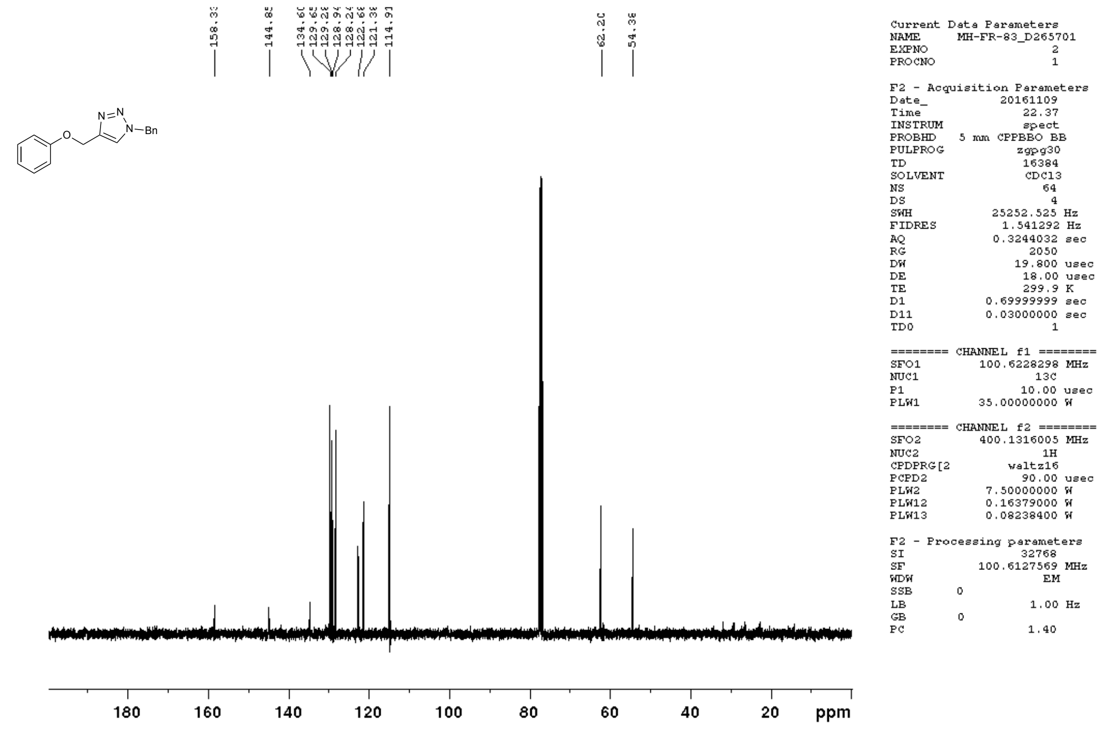

Supplementary Figure 33. ^{13}C NMR spectrum of **3b.**

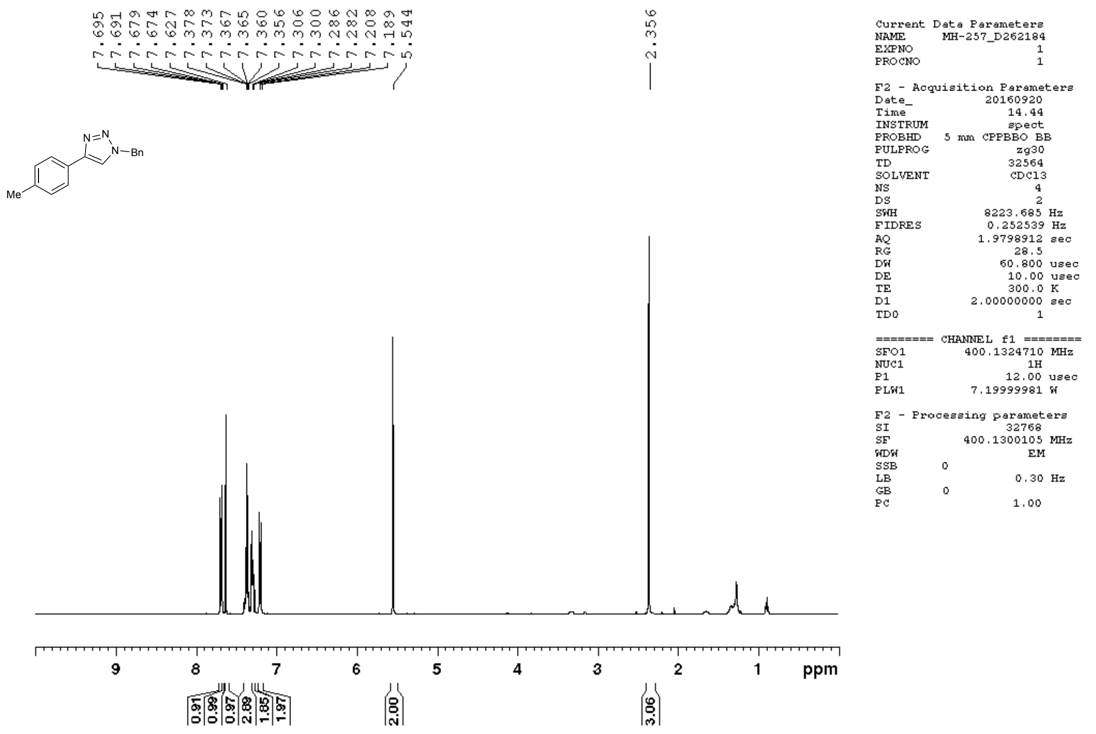

Supplementary Figure 34. ^{1}H NMR spectrum of **3c**.

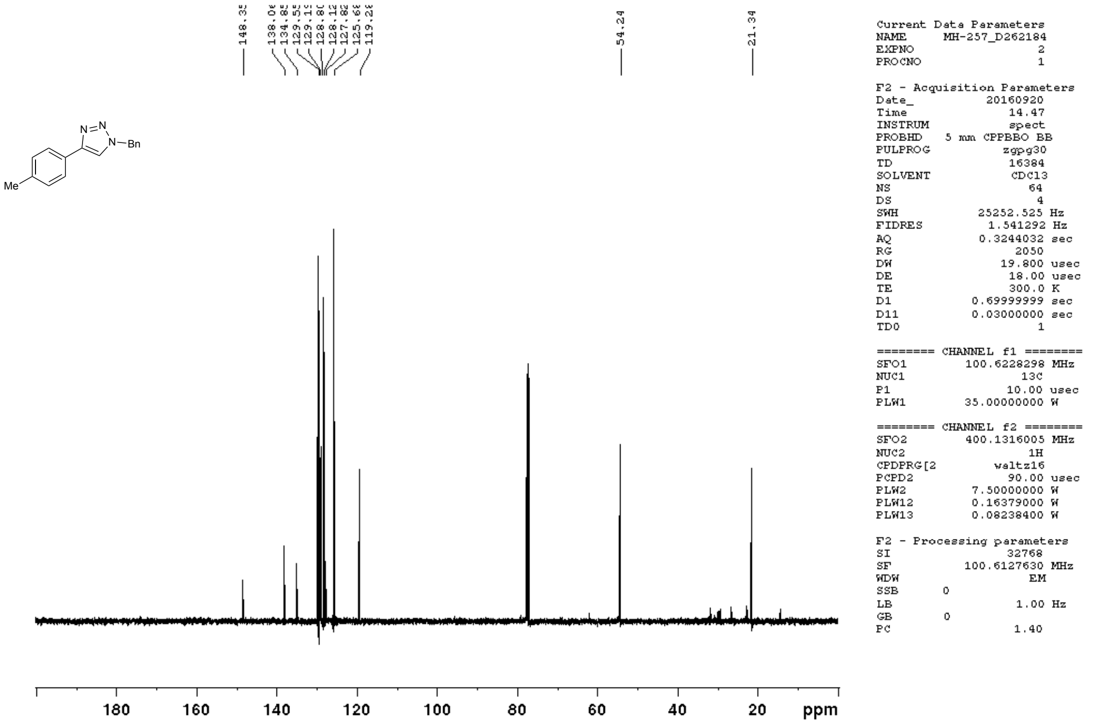

Supplementary Figure 35. ^{13}C NMR spectrum of **3c**.

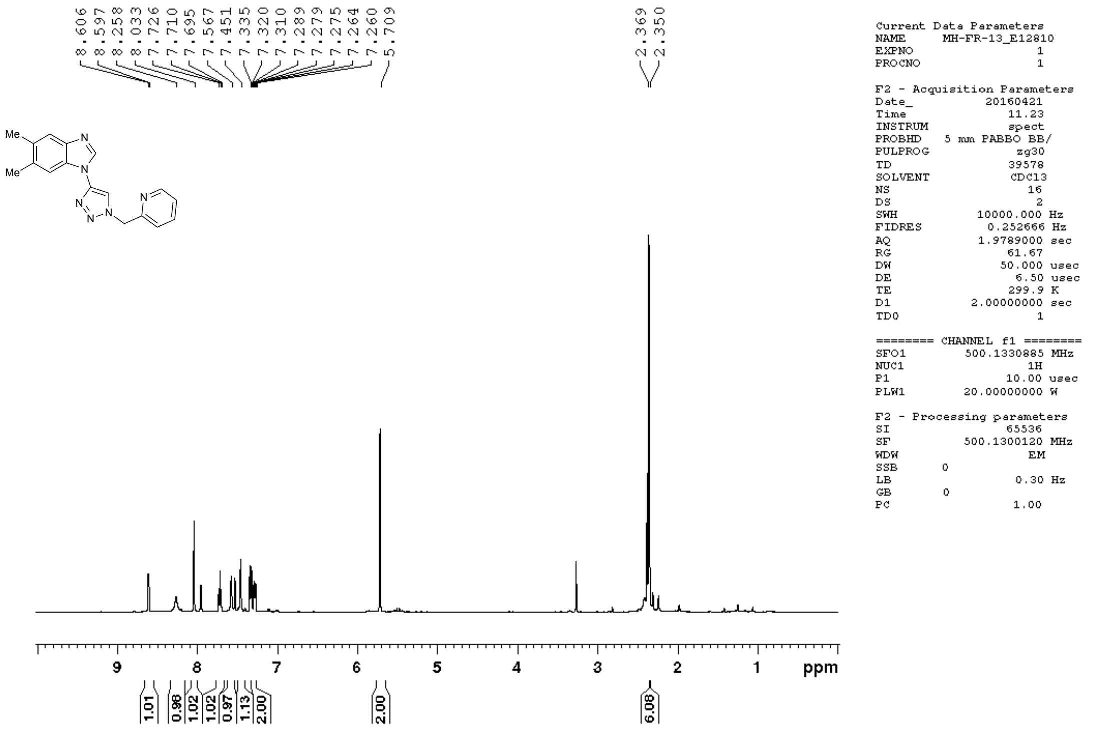

Supplementary Figure 36. ^{1}H NMR spectrum of **4a**.

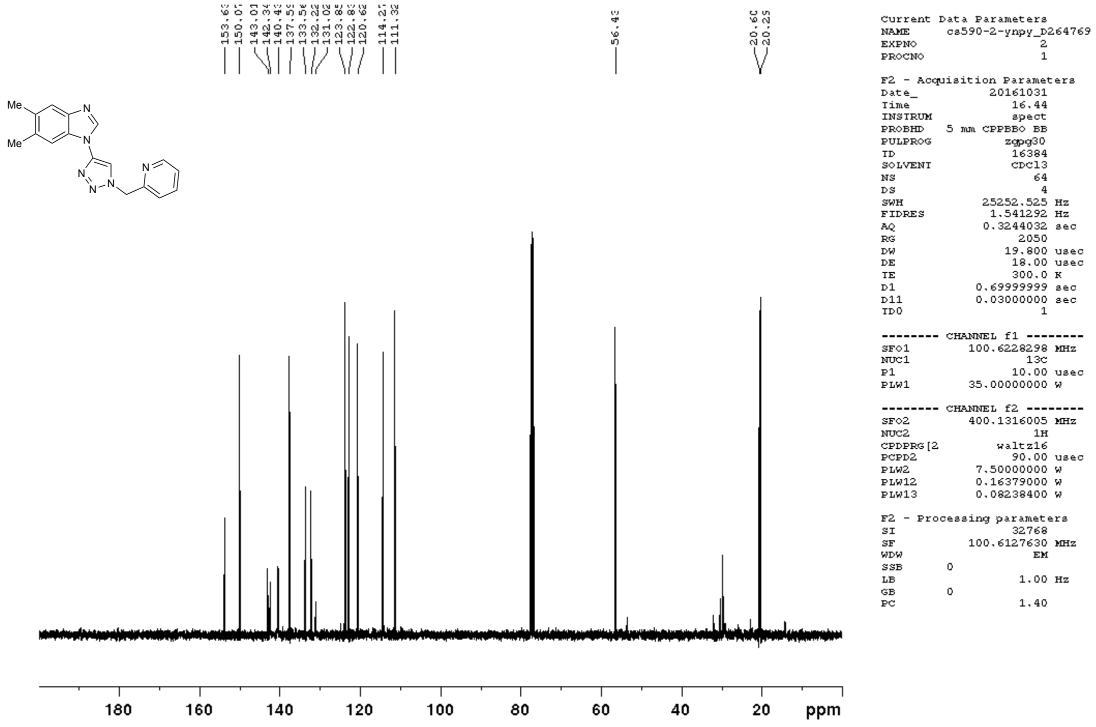

Supplementary Figure 37. ^{13}C NMR spectrum of **4a**.

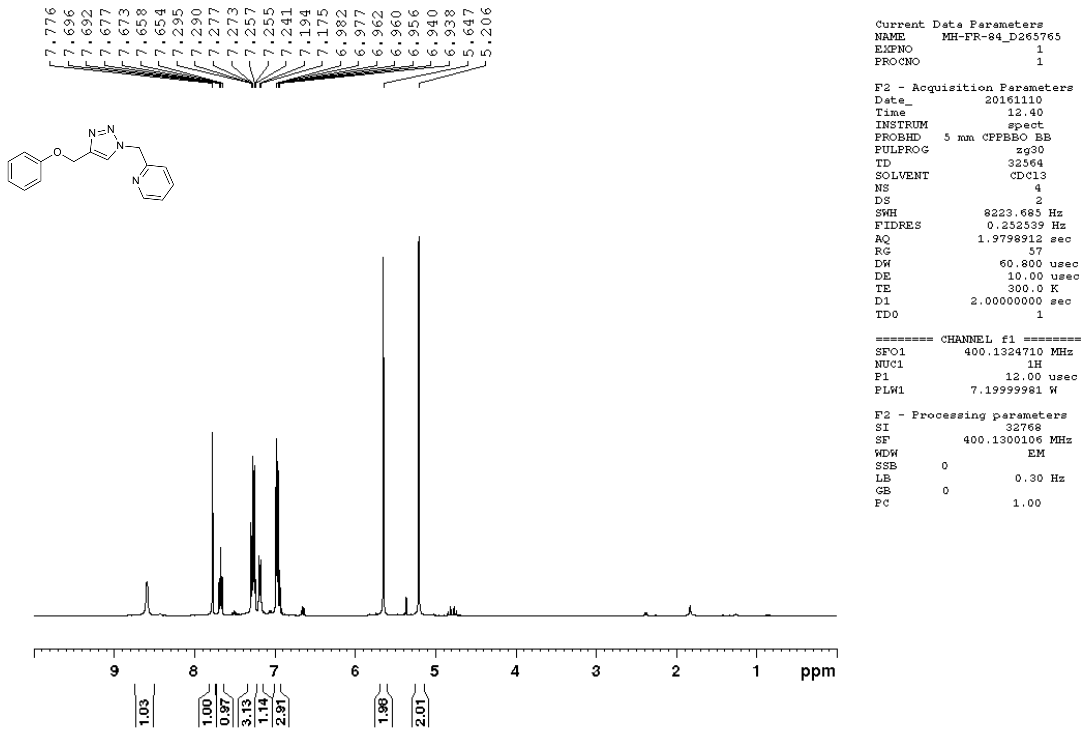

Supplementary Figure 38. ^{1}H NMR spectrum of **4b**.

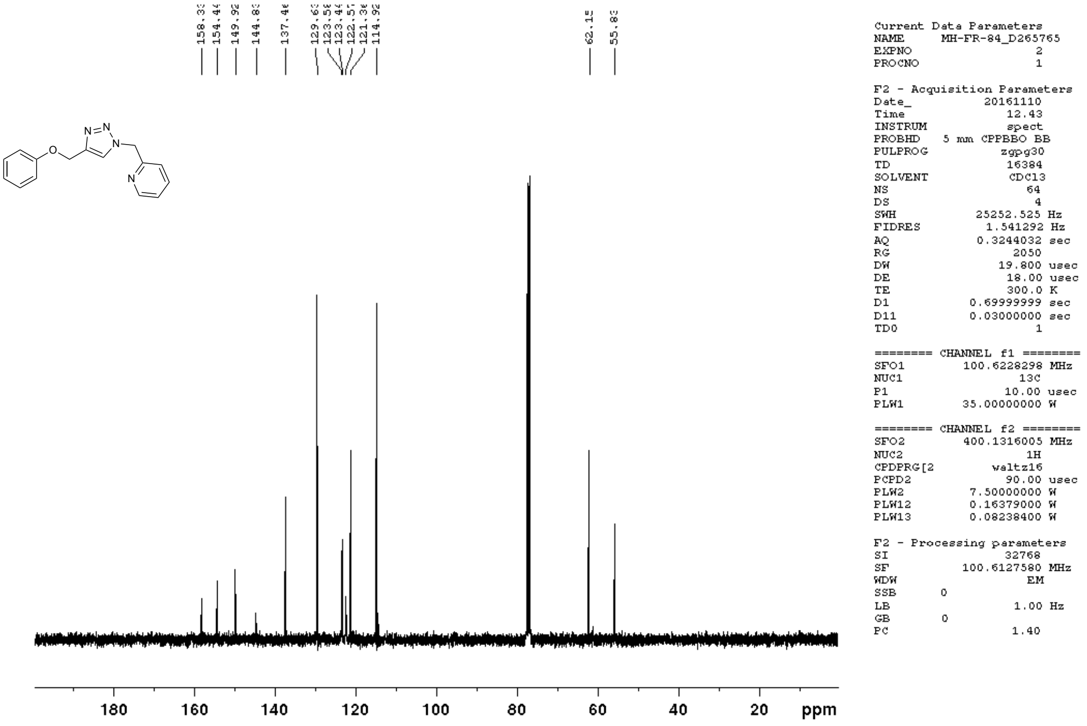

Supplementary Figure 39. ^{13}C NMR spectrum of **4b**.

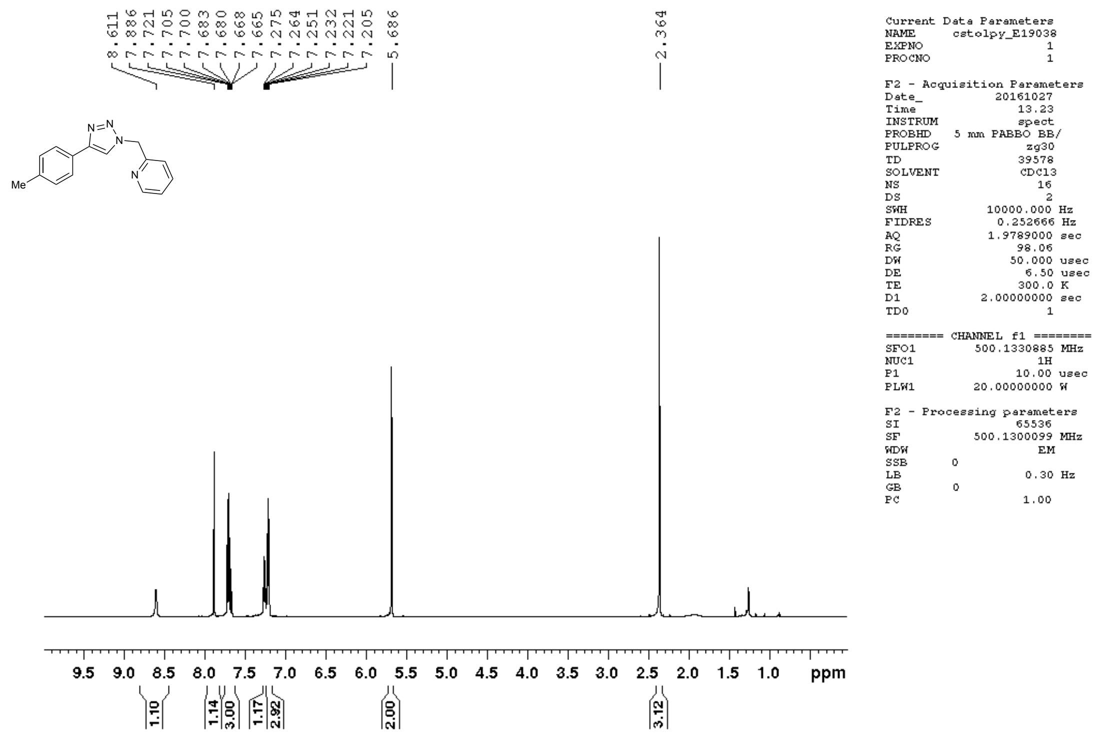

Supplementary Figure 40. ^{1}H NMR spectrum of **4c**.

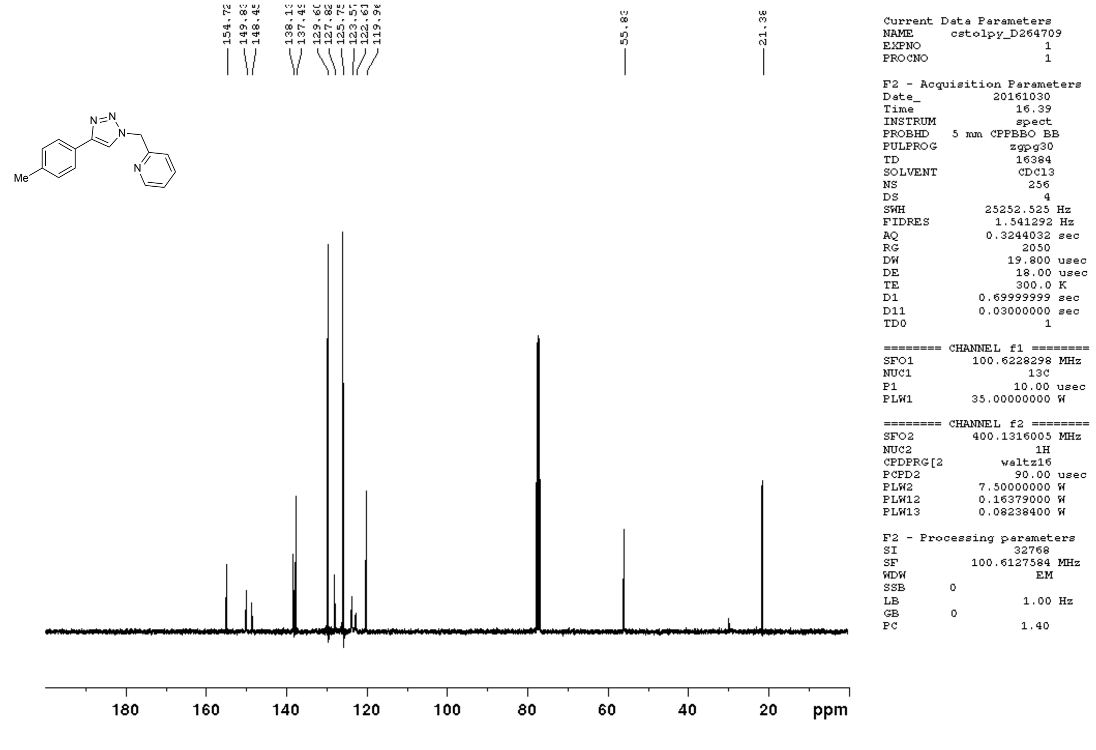

Supplementary Figure 41. ^{13}C NMR spectrum of **4c**.

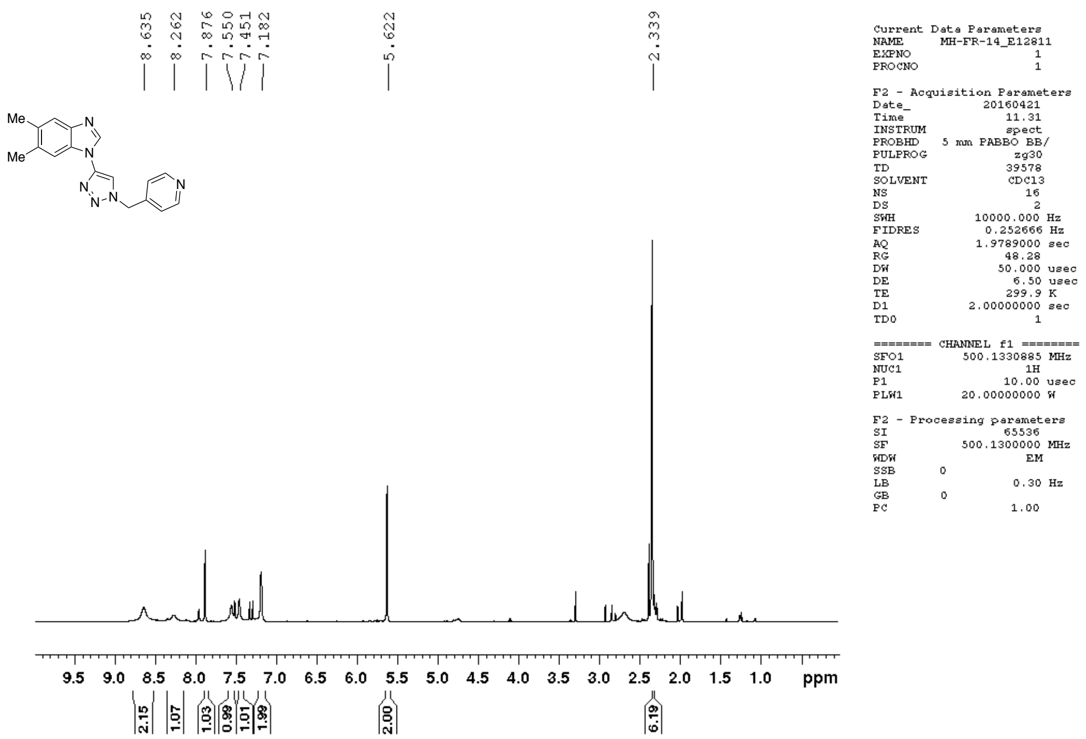

Supplementary Figure 42. ^{1}H NMR spectrum of **5a**.

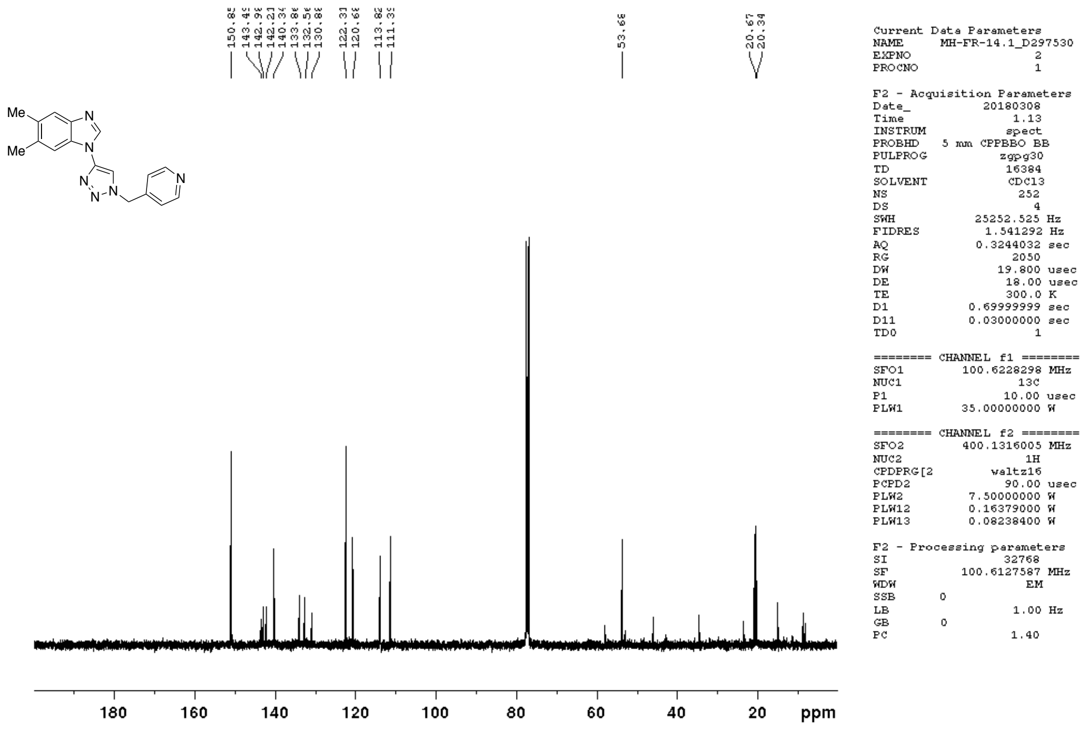

Supplementary Figure 43. ^{13}C NMR spectrum of **5a**.

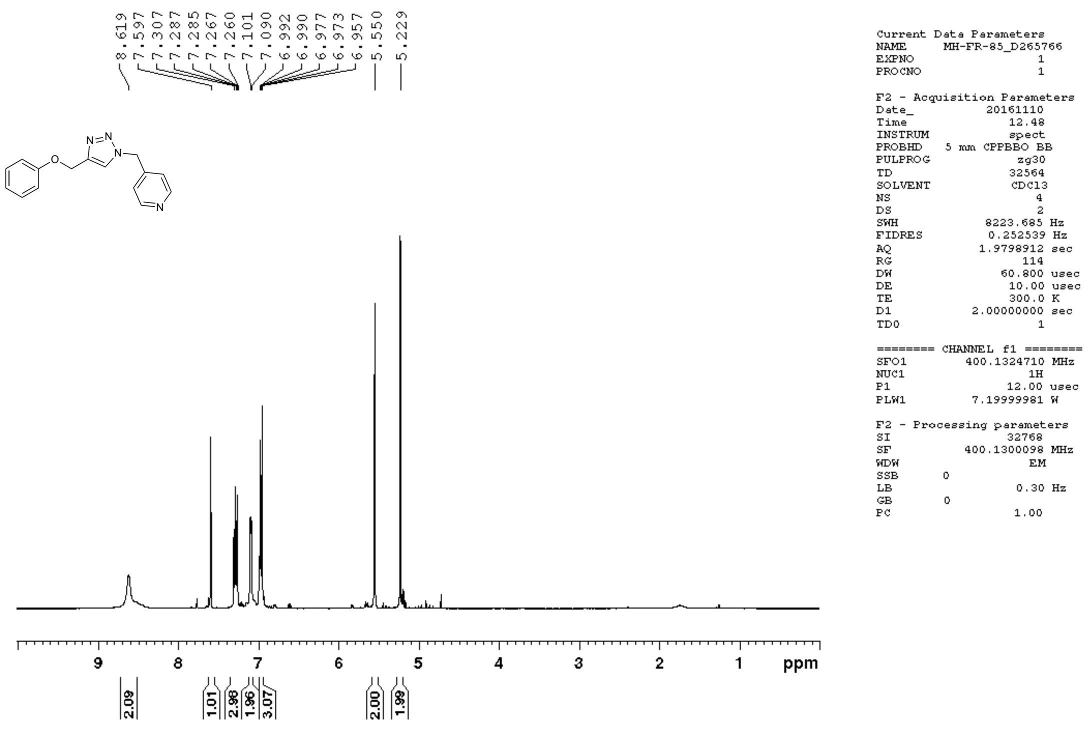

Supplementary Figure 44. ^{1}H NMR spectrum of **5b**.

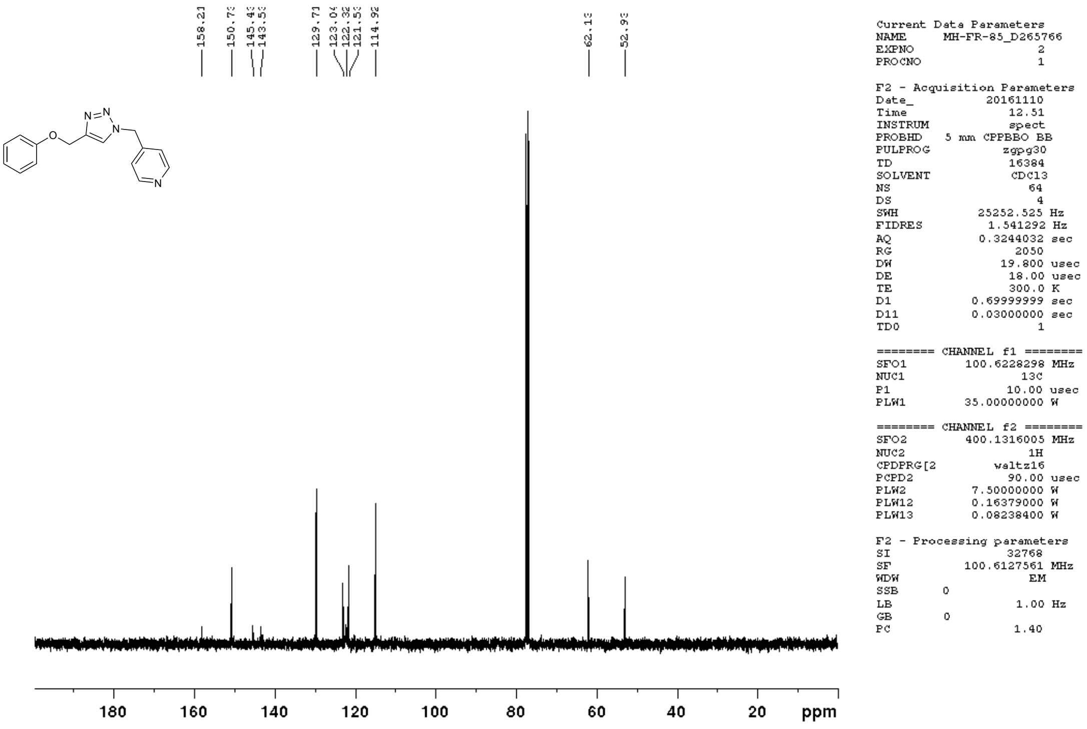

Supplementary Figure 45. ^{13}C NMR spectrum of **5b**.

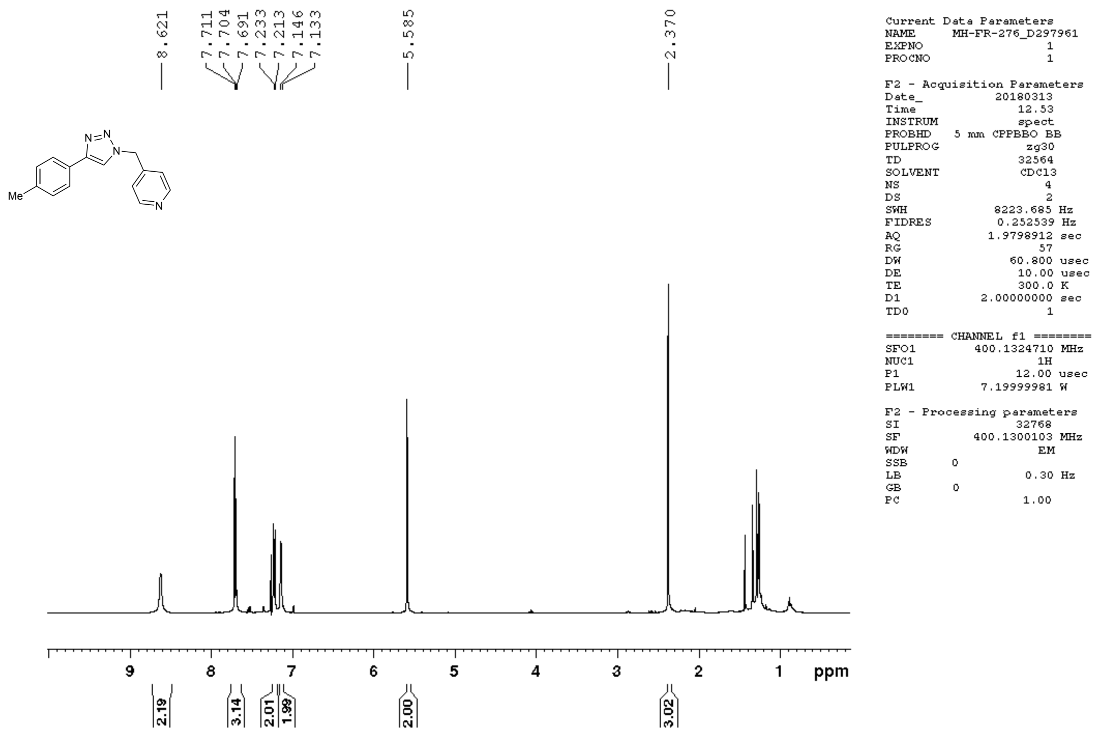

Supplementary Figure 46. ^{1}H NMR spectrum of **5c**.

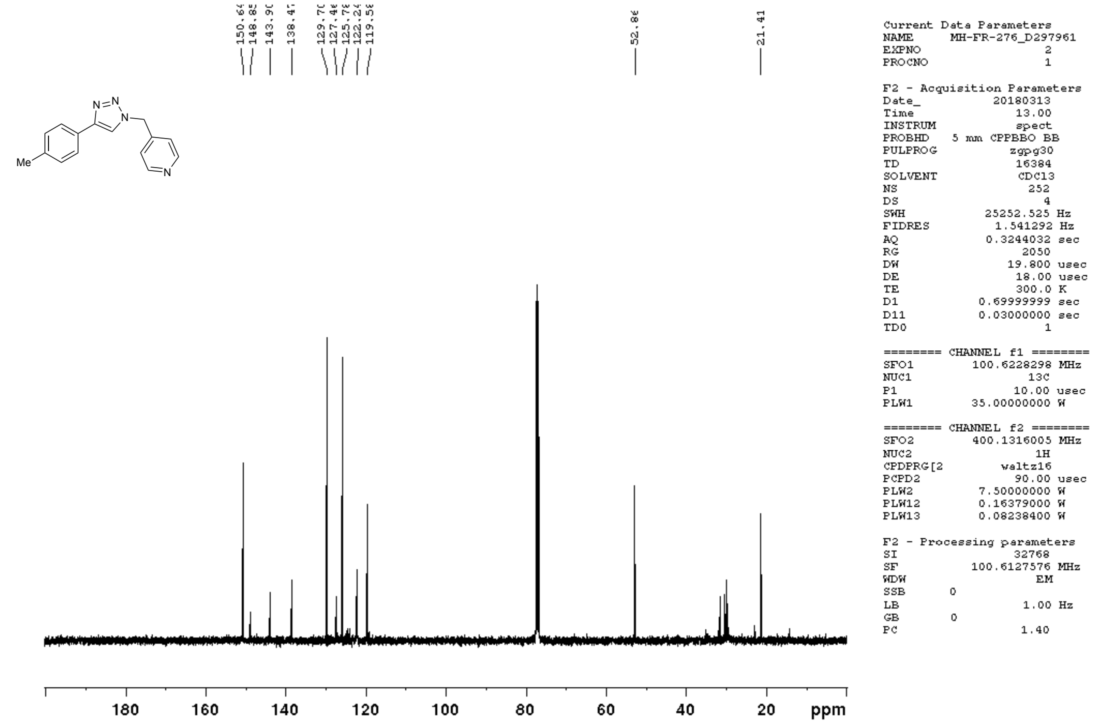

Supplementary Figure 47. ^{13}C NMR spectrum of **5c**.

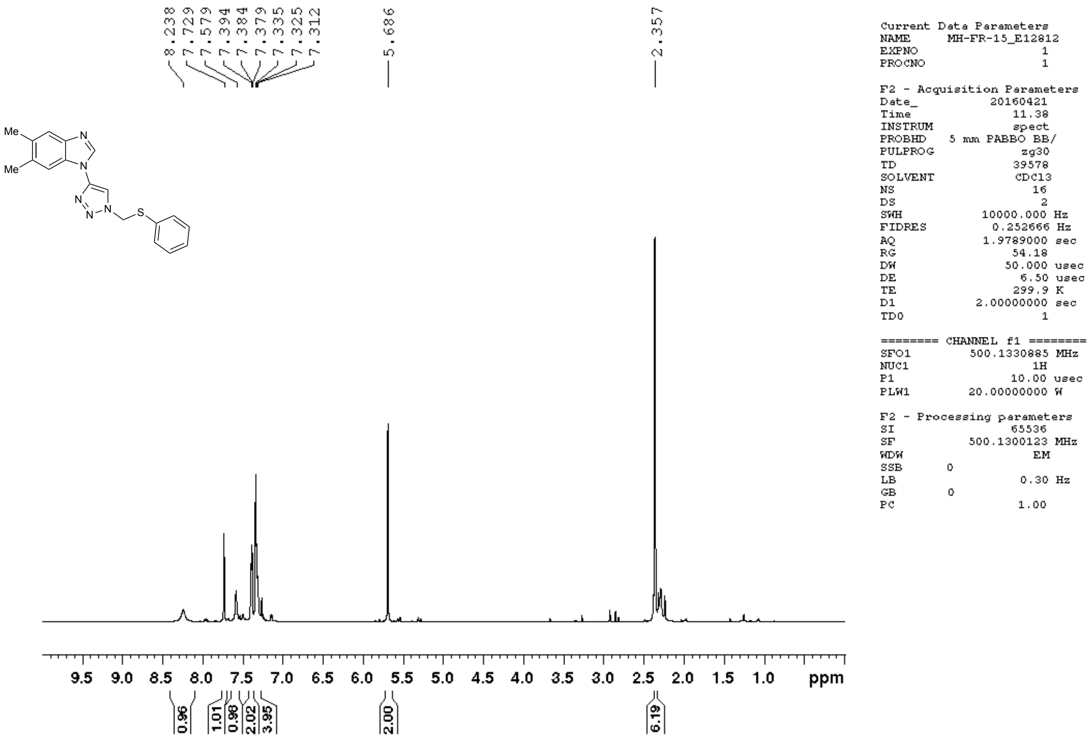

Supplementary Figure 48. ^{1}H NMR spectrum of **6a**.

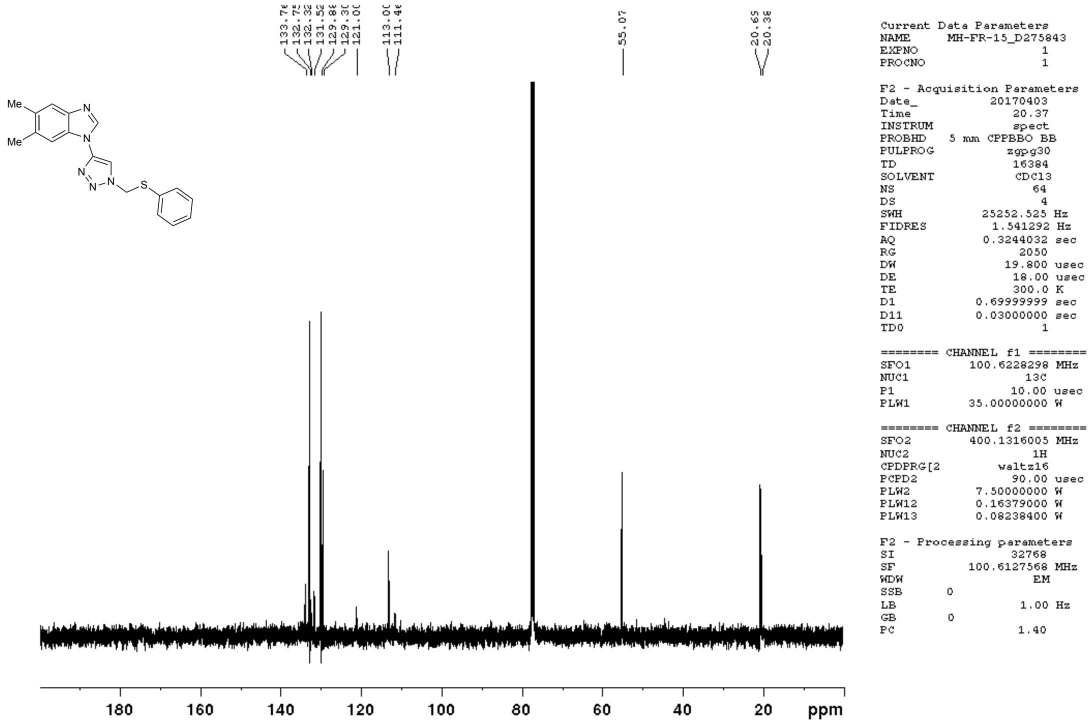

Supplementary Figure 49. ^{13}C NMR spectrum of **6a**.

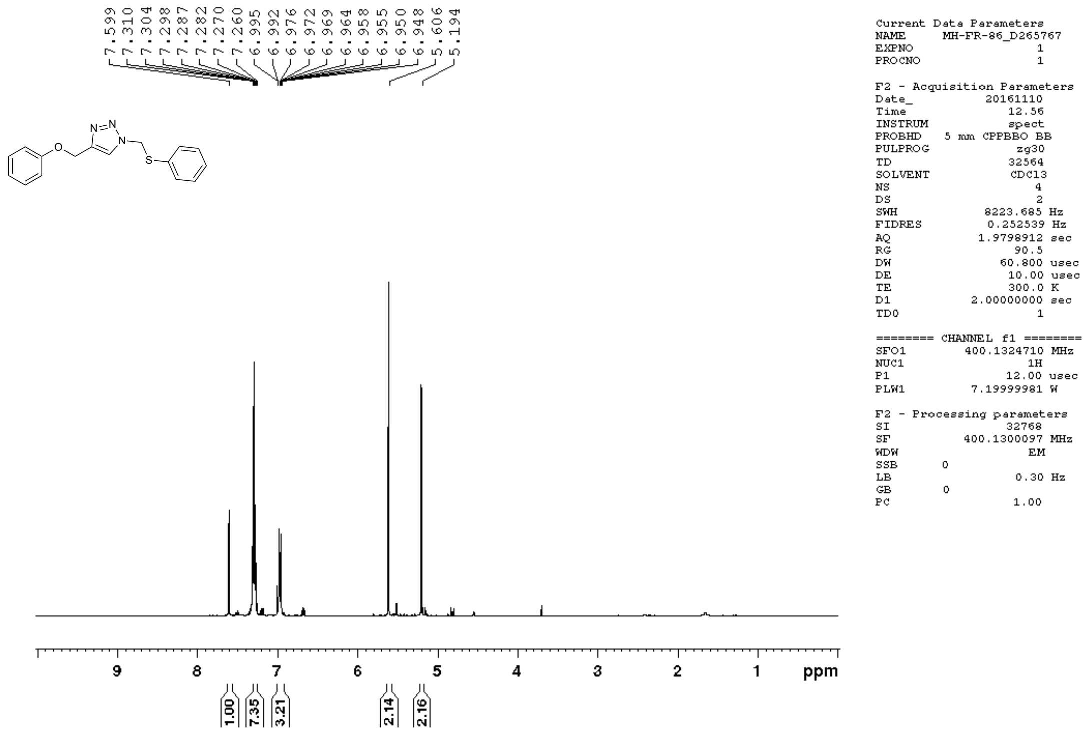

Supplementary Figure 50. ^{1}H NMR spectrum of **6b**.

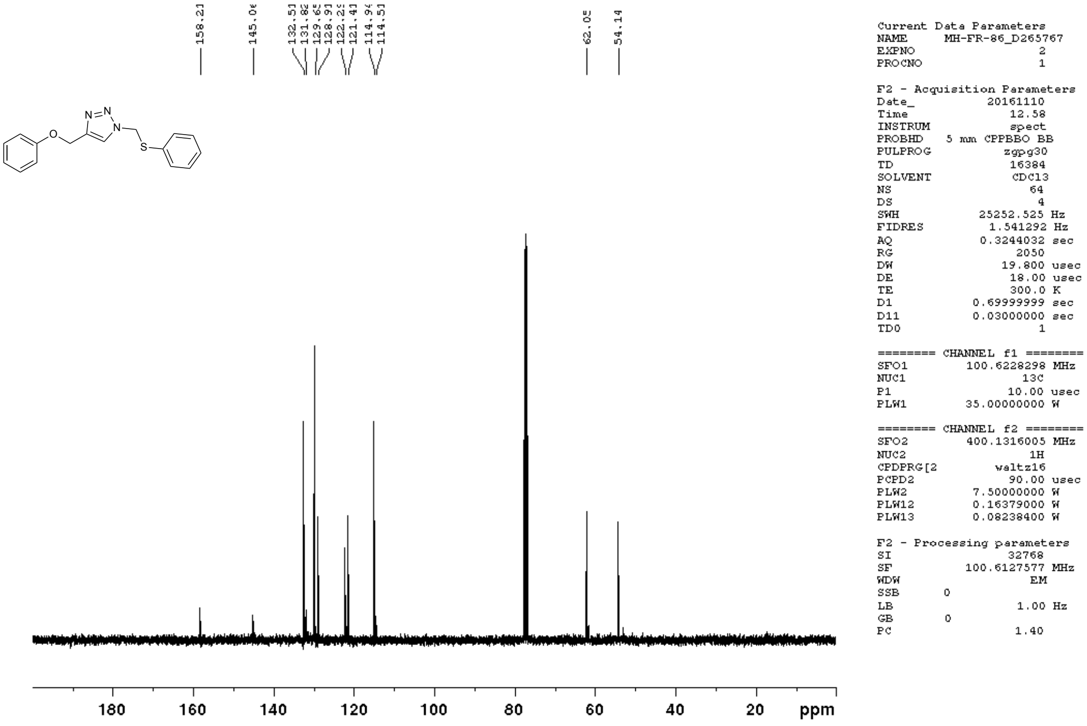

Supplementary Figure 51. ^{13}C NMR spectrum of **6b**.

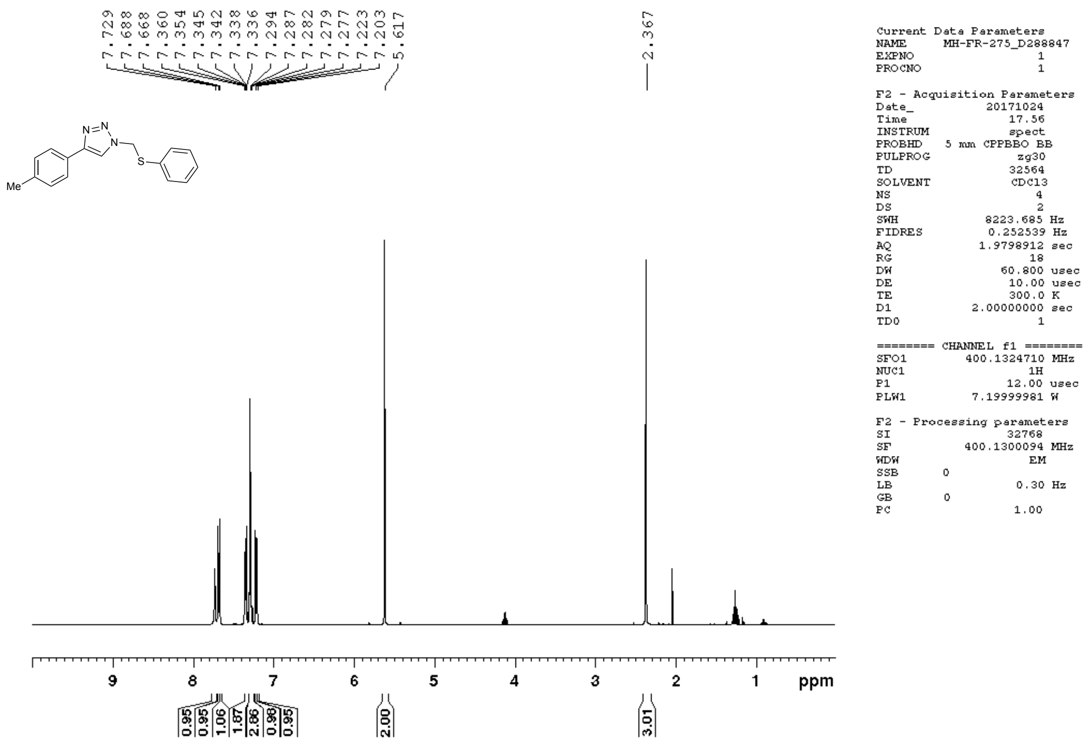

Supplementary Figure 52. ^{1}H NMR spectrum of **6c**.

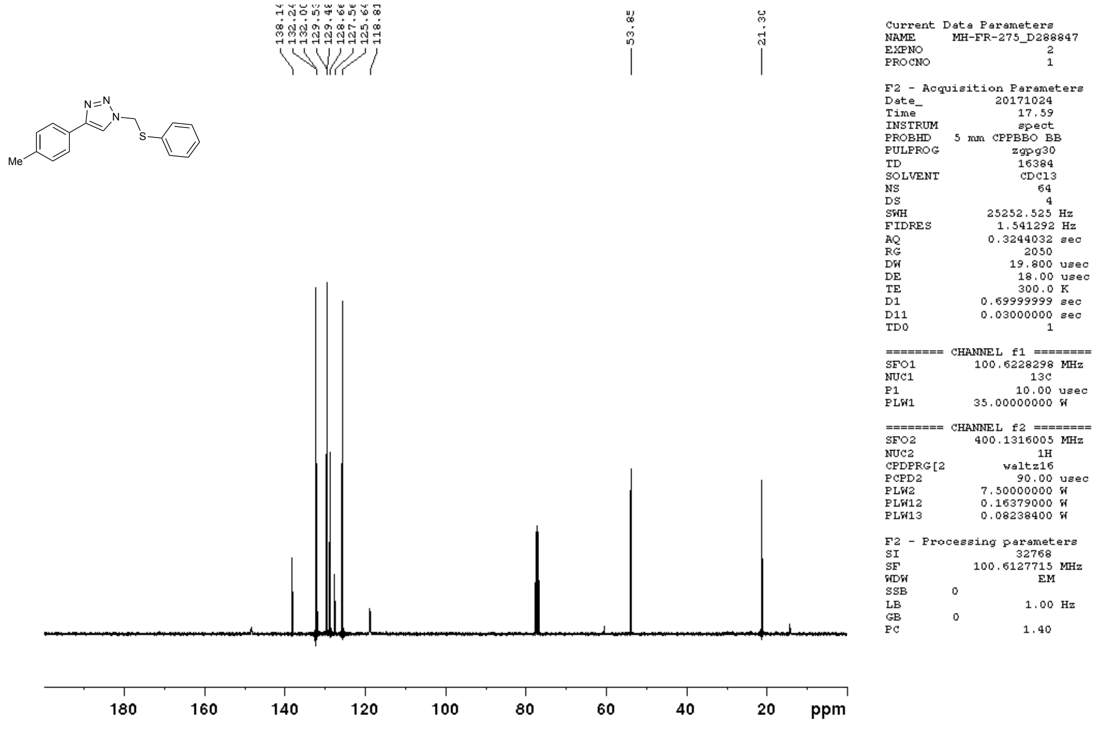

Supplementary Figure 53. ^{13}C NMR spectrum of **6c**.

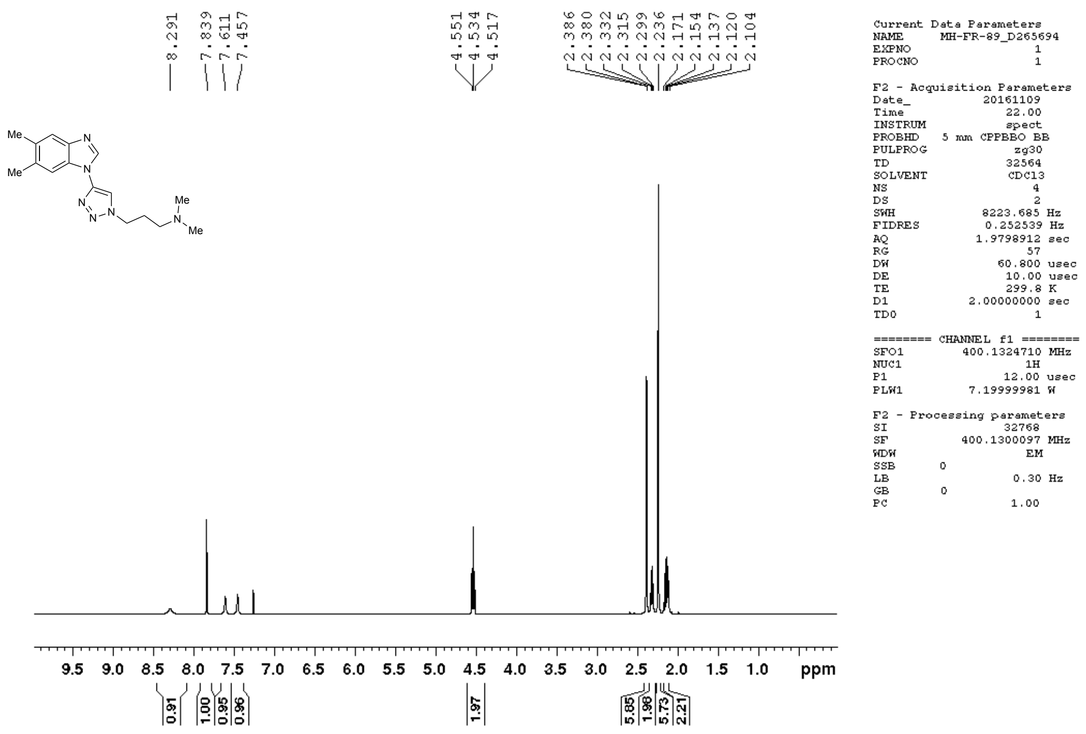

Supplementary Figure 54. ^{1}H NMR spectrum of **7a**.

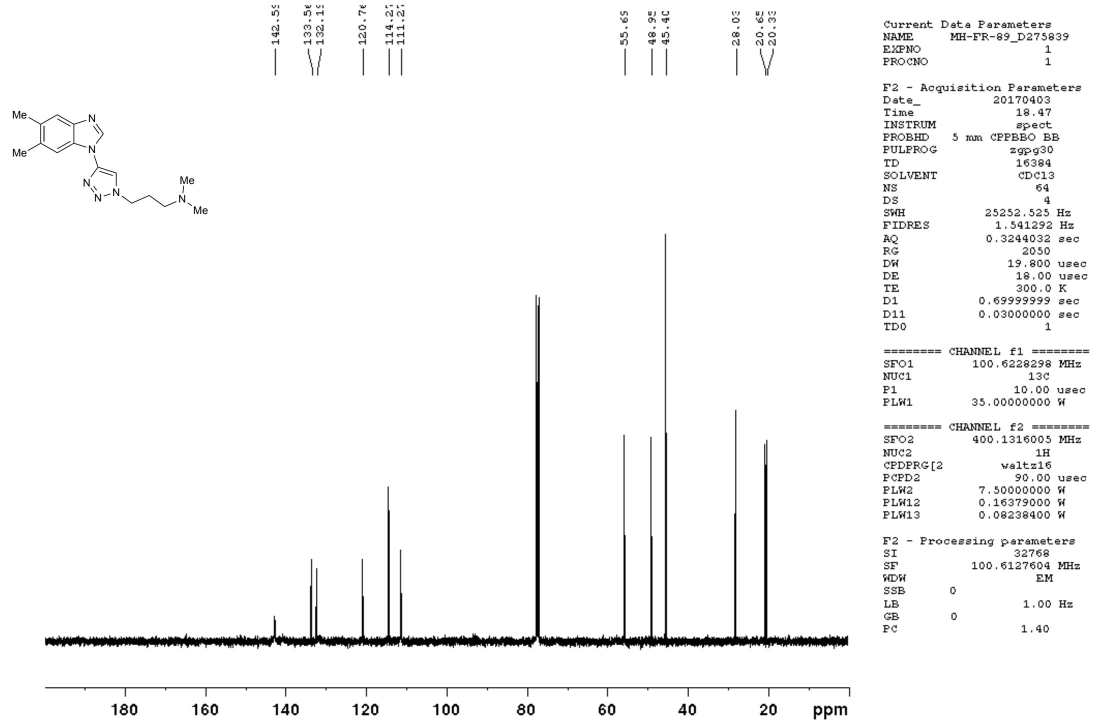

Supplementary Figure 55. ^{13}C NMR spectrum of **7a**.

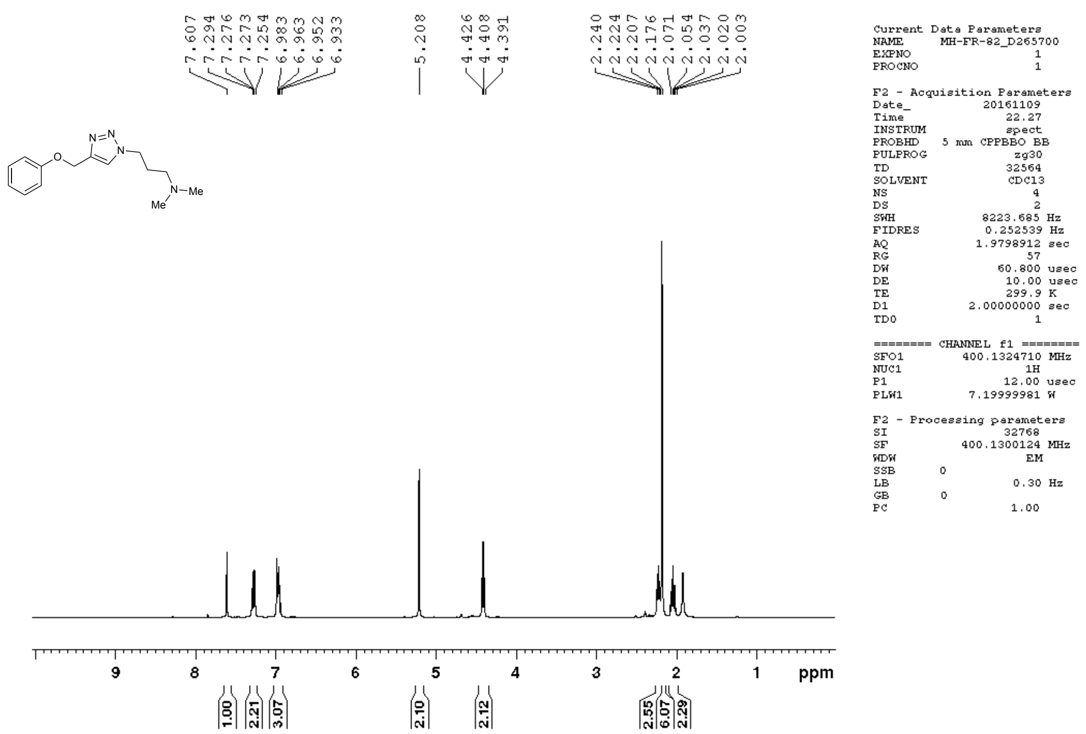

Supplementary Figure 56. ^{1}H NMR spectrum of **7b**.

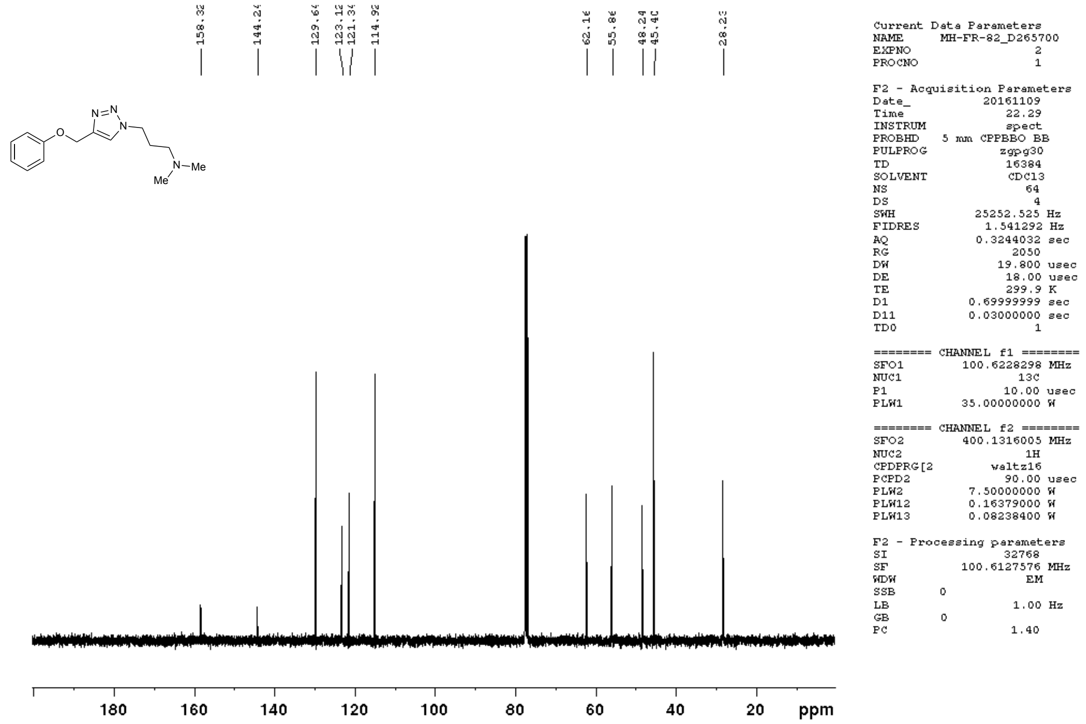

Supplementary Figure 57. ^{13}C NMR spectrum of **7b**.

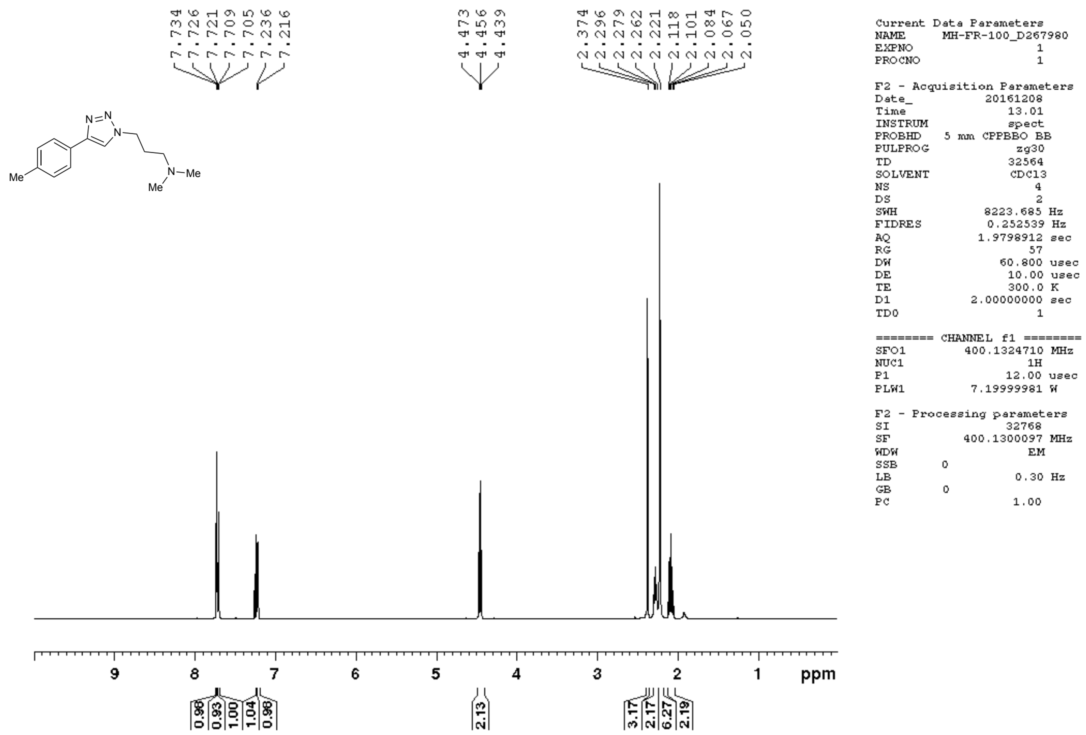

Supplementary Figure 58. ^{1}H NMR spectrum of **7c**.

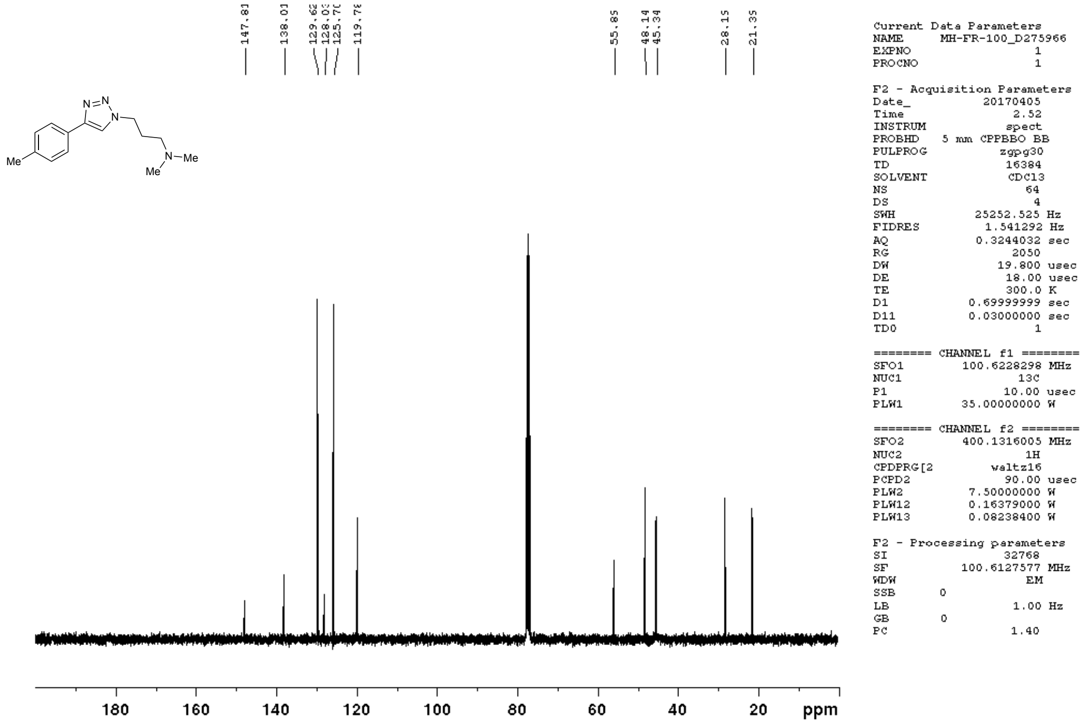

Supplementary Figure 59. ^{13}C NMR spectrum of **7c**.

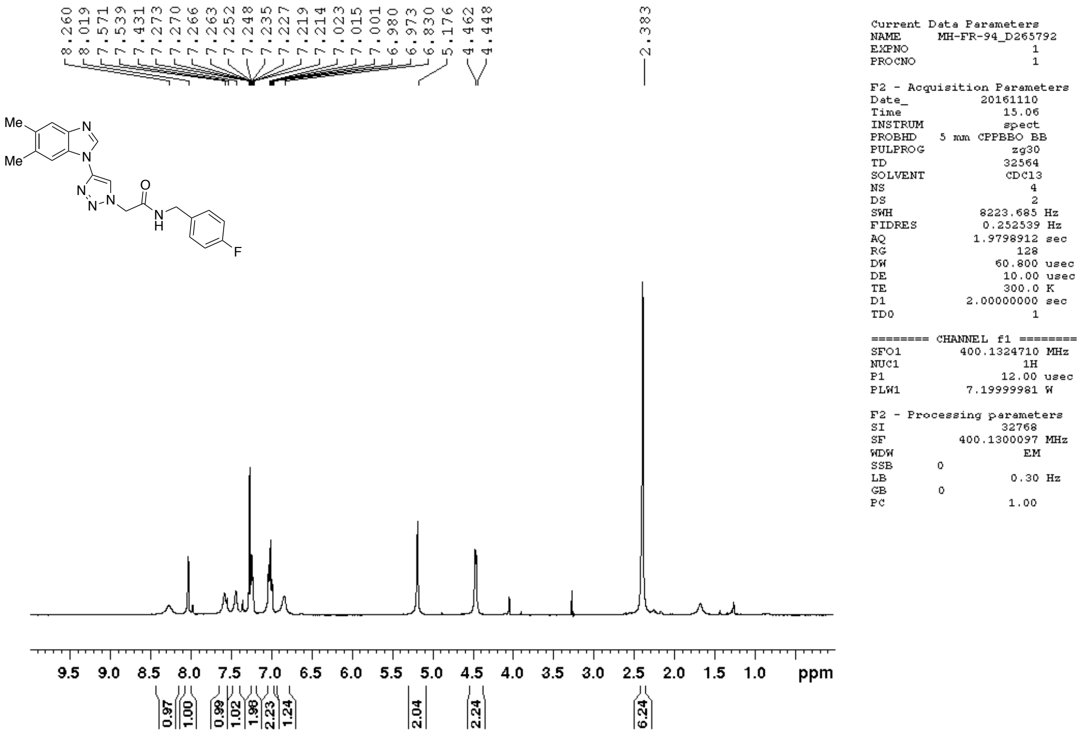

Supplementary Figure 60. ^{1}H NMR spectrum of **8a**.

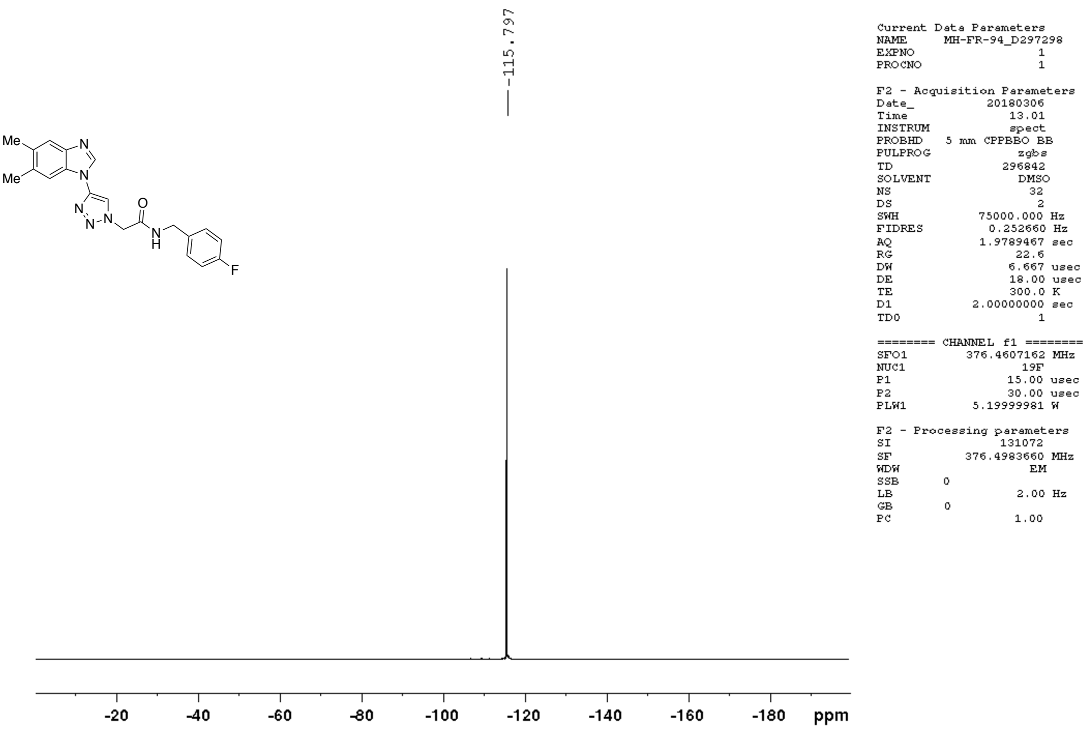

Supplementary Figure 61. ^{19}F NMR spectrum of **8a**.

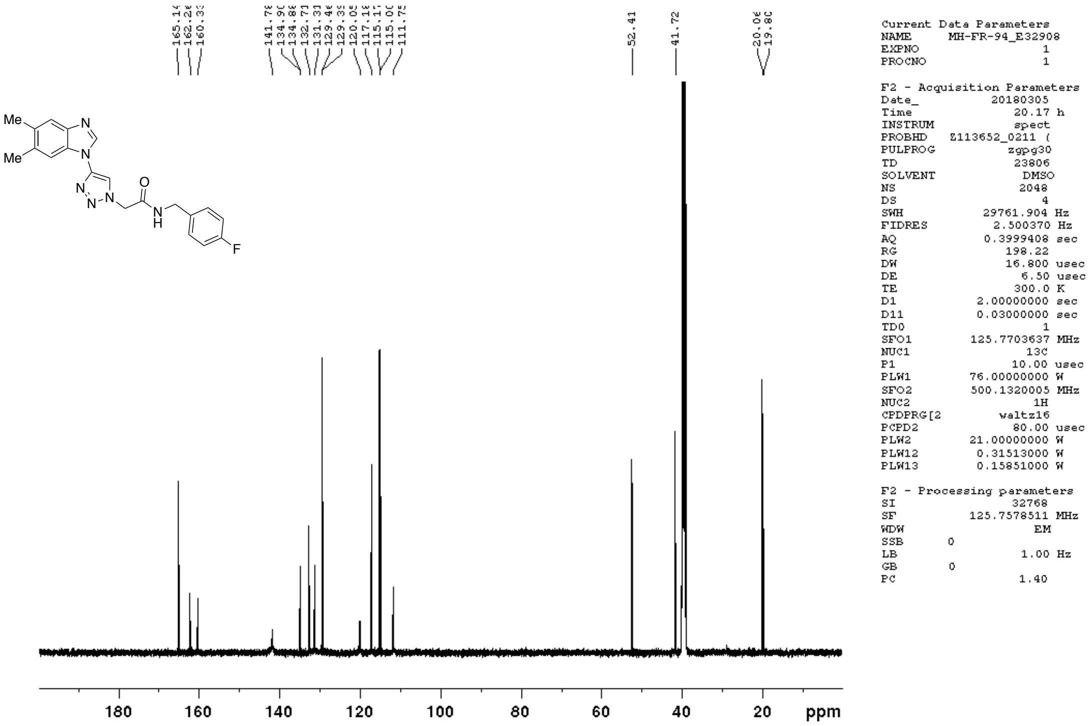

Supplementary Figure 62. ^{13}C NMR spectrum of **8a**.

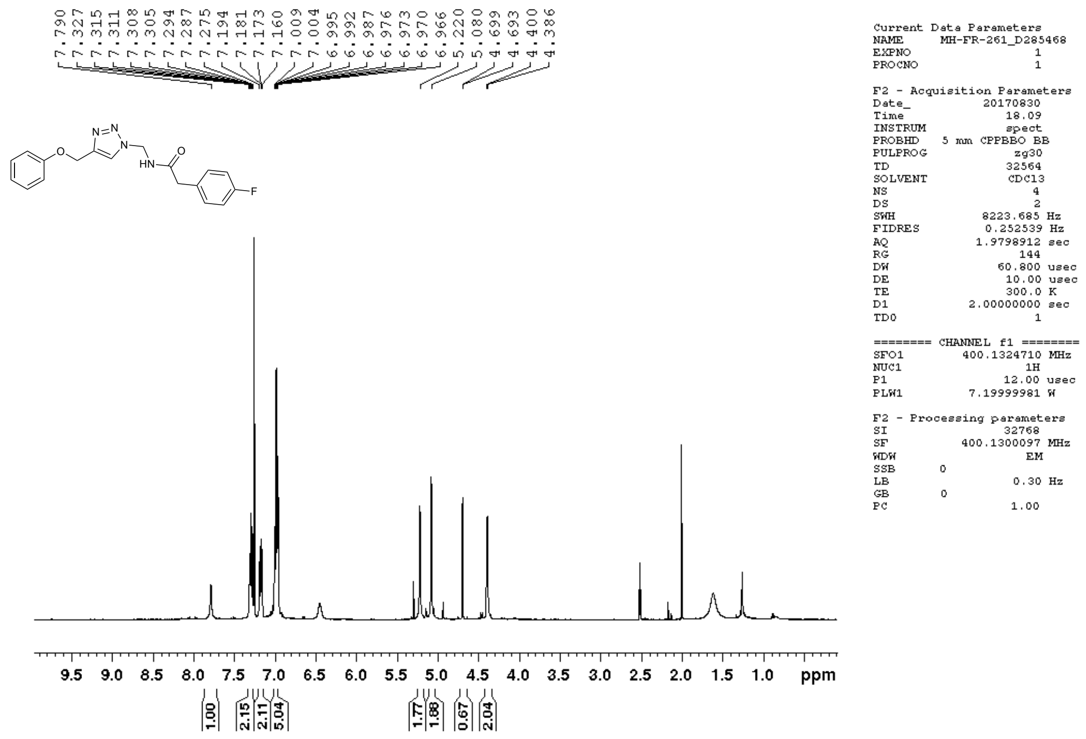

Supplementary Figure 63. ^{1}H NMR spectrum of **8b**.

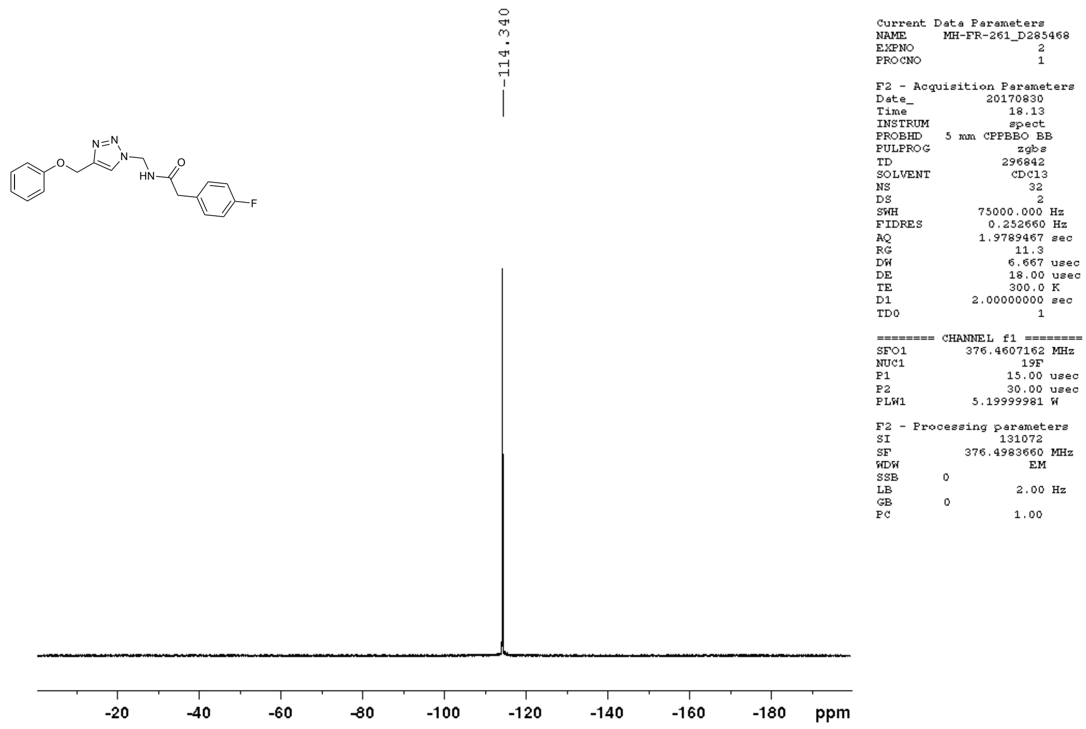

Supplementary Figure 64. ^{19}F NMR spectrum of **8b**.

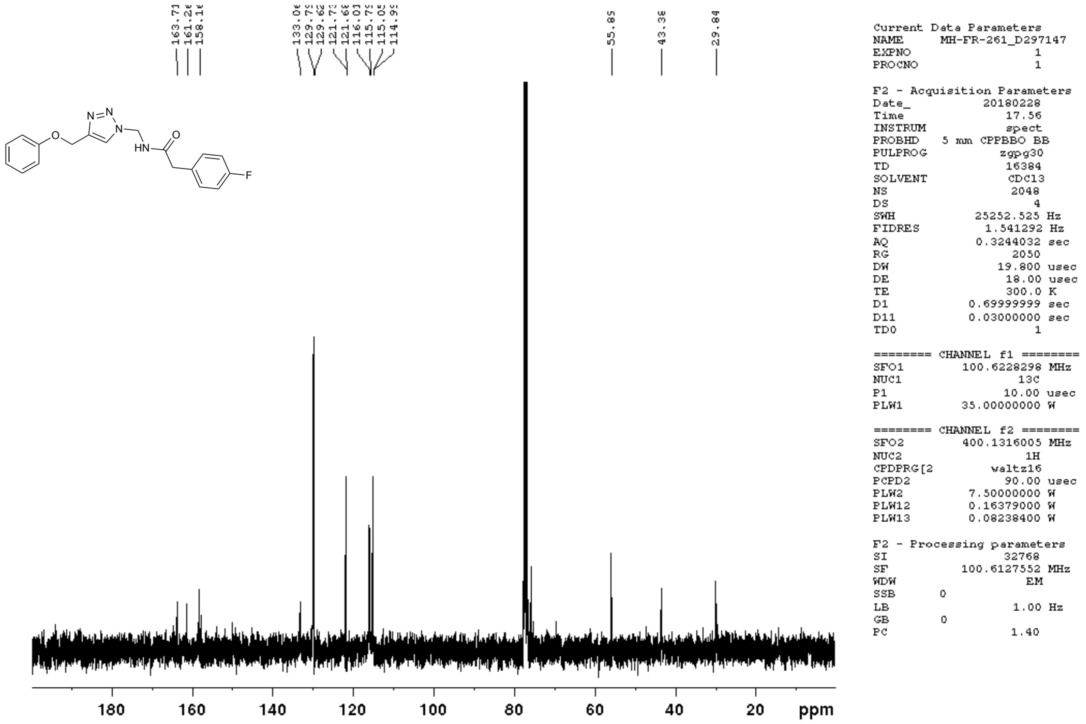

Supplementary Figure 65. ^{13}C NMR spectrum of **8b**.

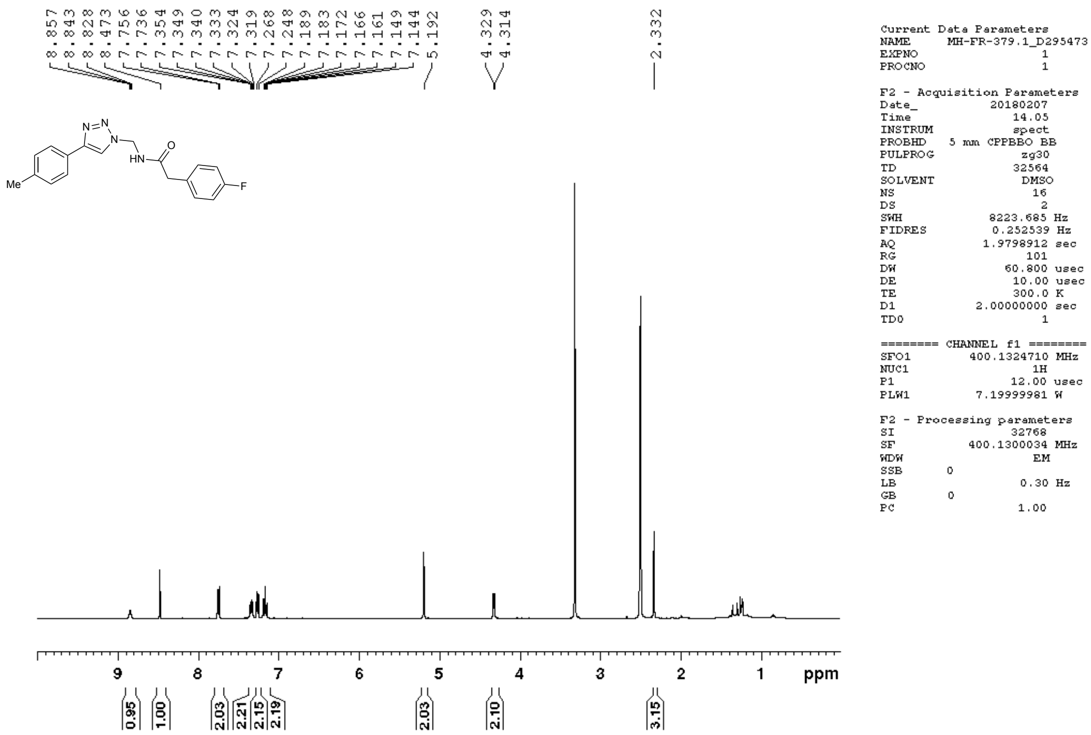

Supplementary Figure 66. ^{1}H NMR spectrum of **8c**.

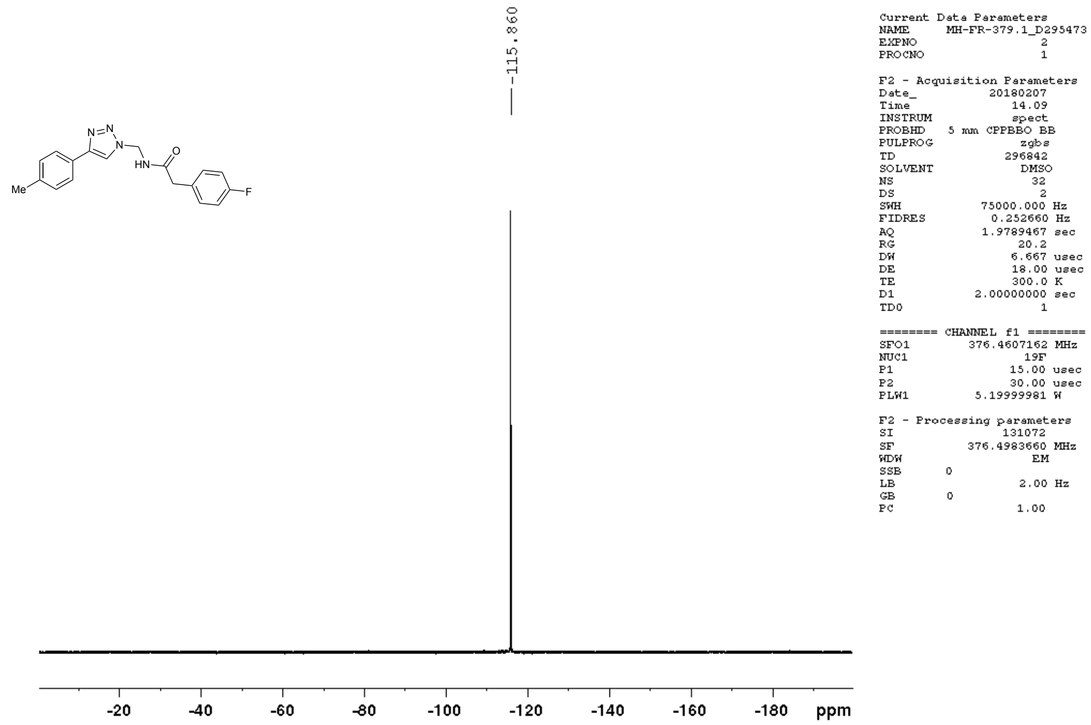

Supplementary Figure 67. ^{19}F NMR spectrum of **8c**.

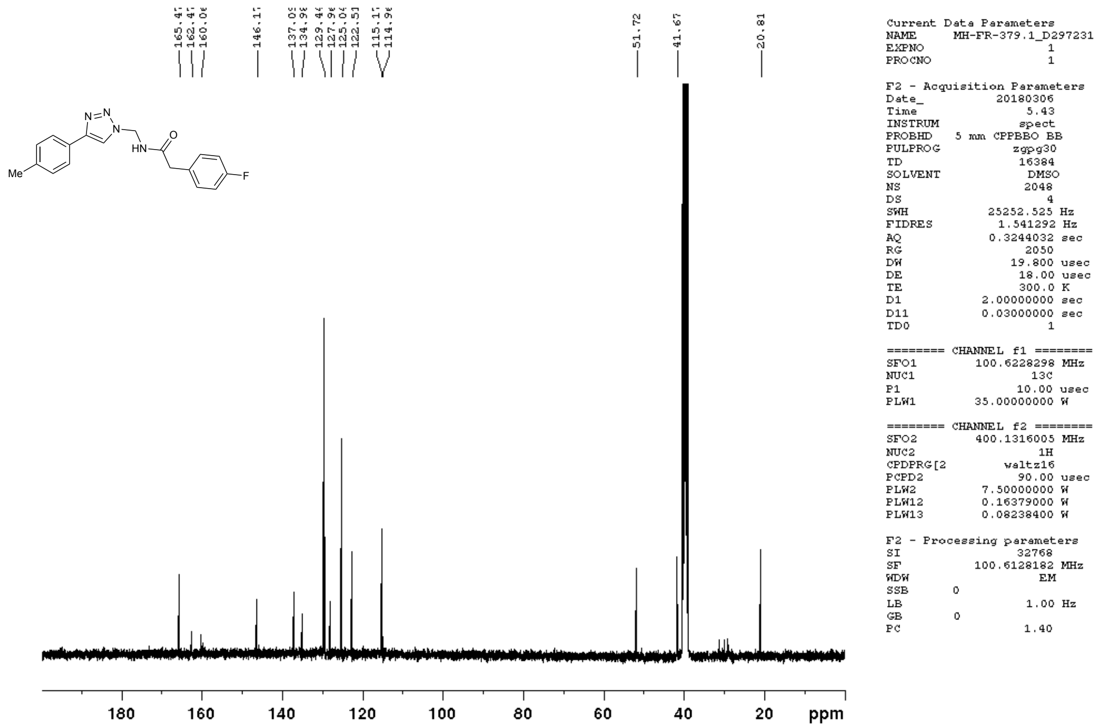

Supplementary Figure 68. ^{13}C NMR spectrum of **8c**.

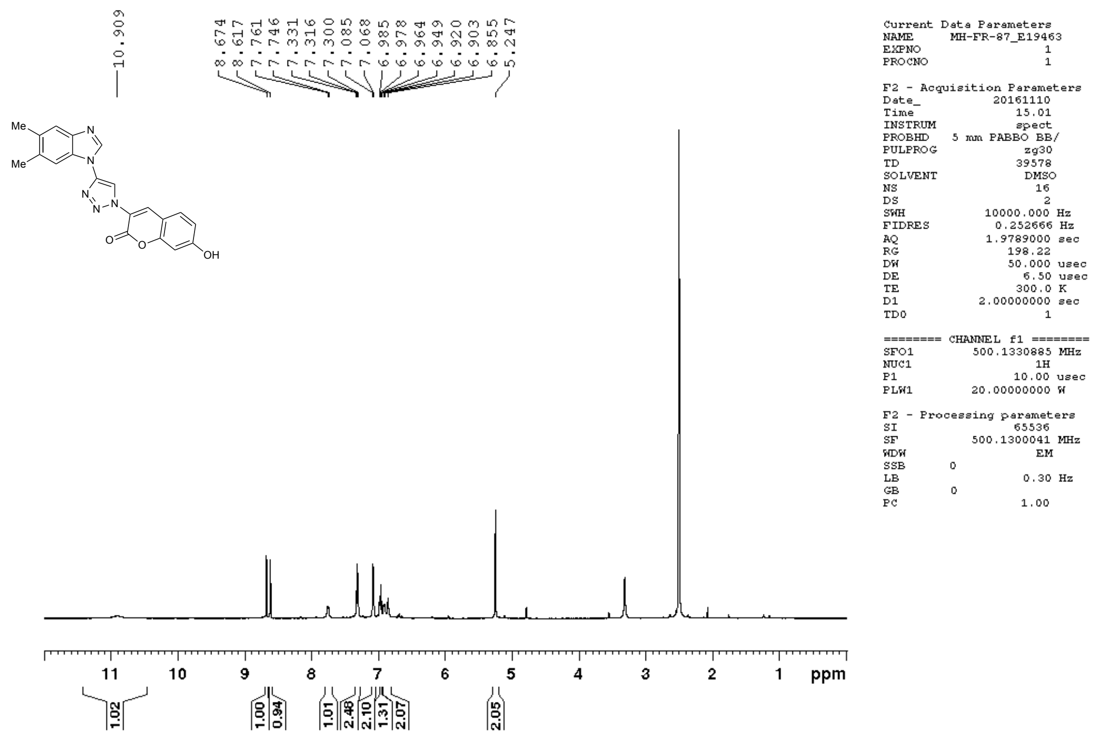

Supplementary Figure 69. ^{1}H NMR spectrum of **9a**.

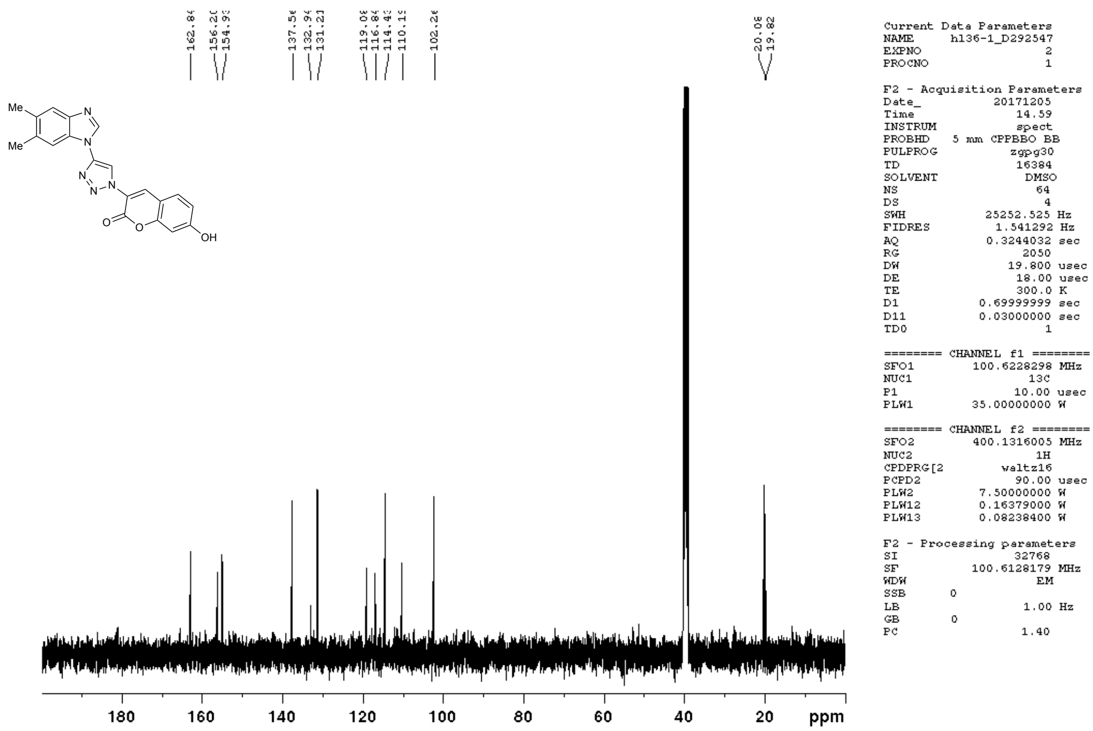

Supplementary Figure 70. ^{13}C NMR spectrum of **9a**.

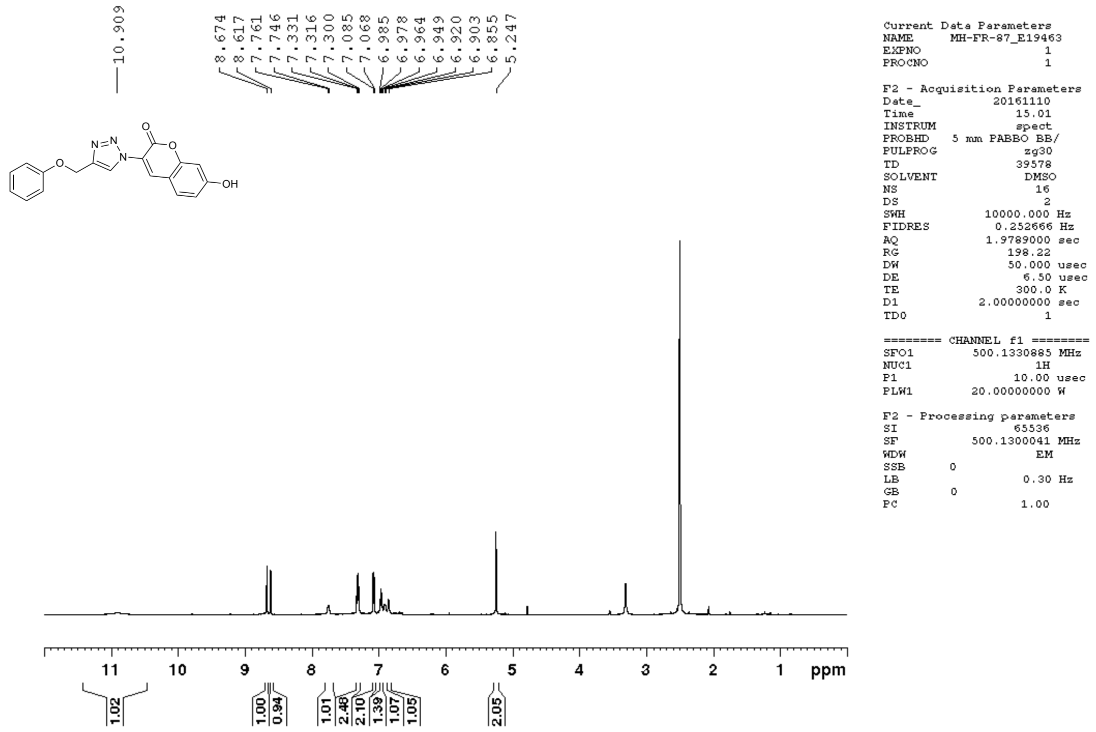

Supplementary Figure 71. ^{1}H NMR spectrum of **9b**.

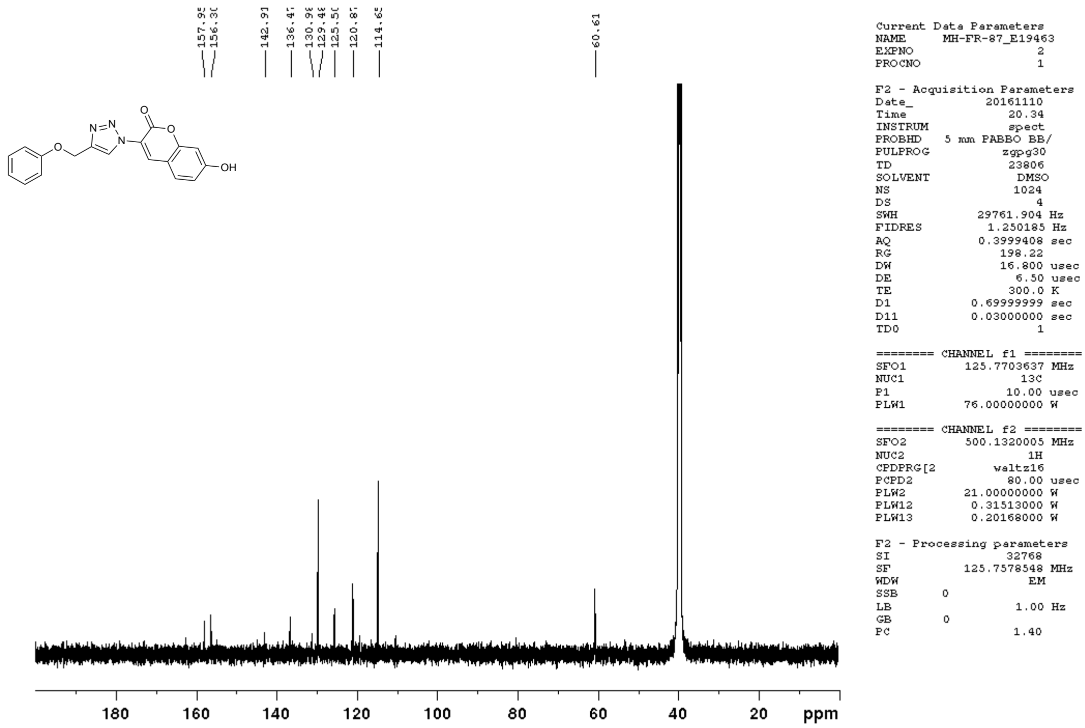

Supplementary Figure 72. ^{13}C NMR spectrum of **9b**.

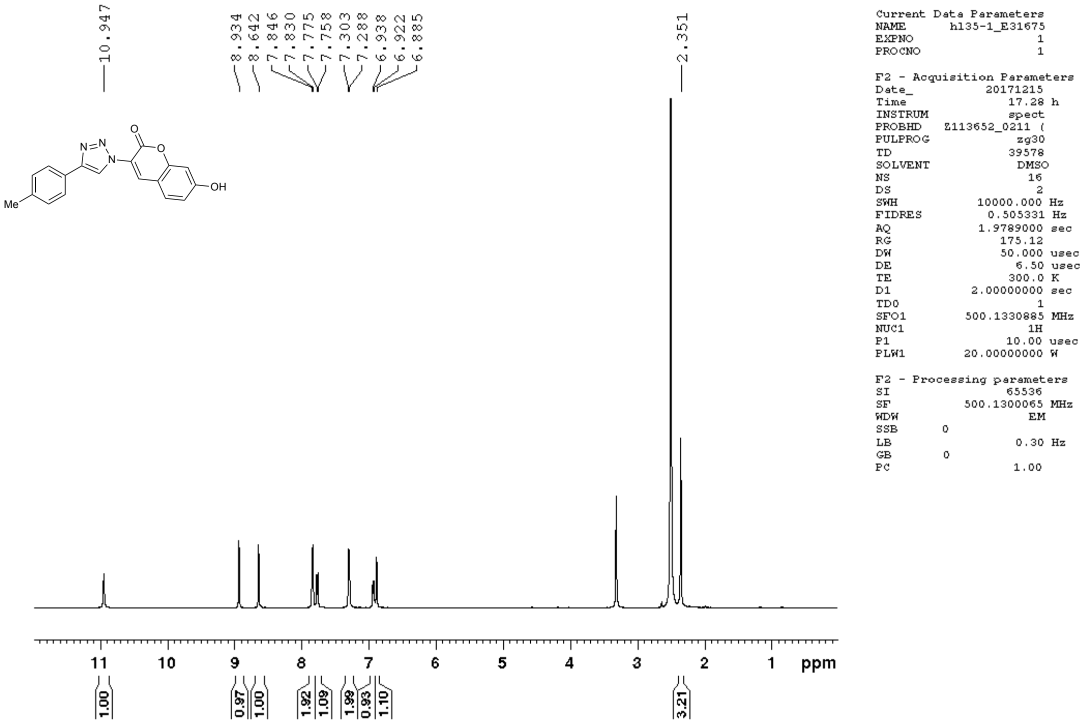

Supplementary Figure 73. ^{1}H NMR spectrum of **9c**.

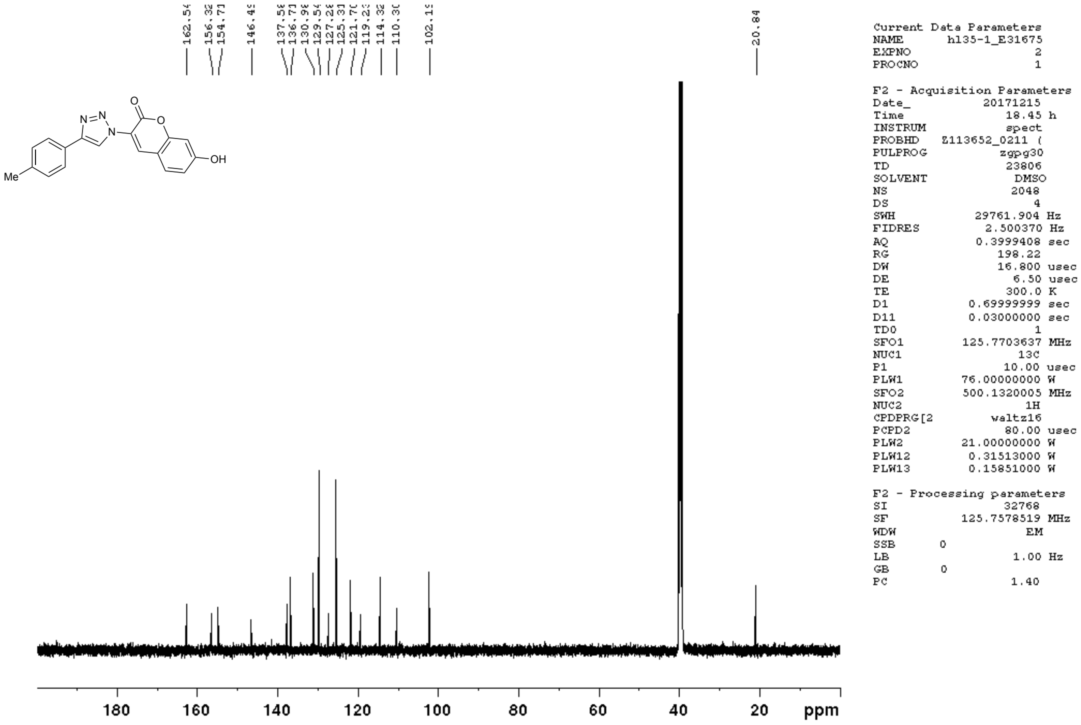

Supplementary Figure 74. ^{13}C NMR spectrum of **9c**.

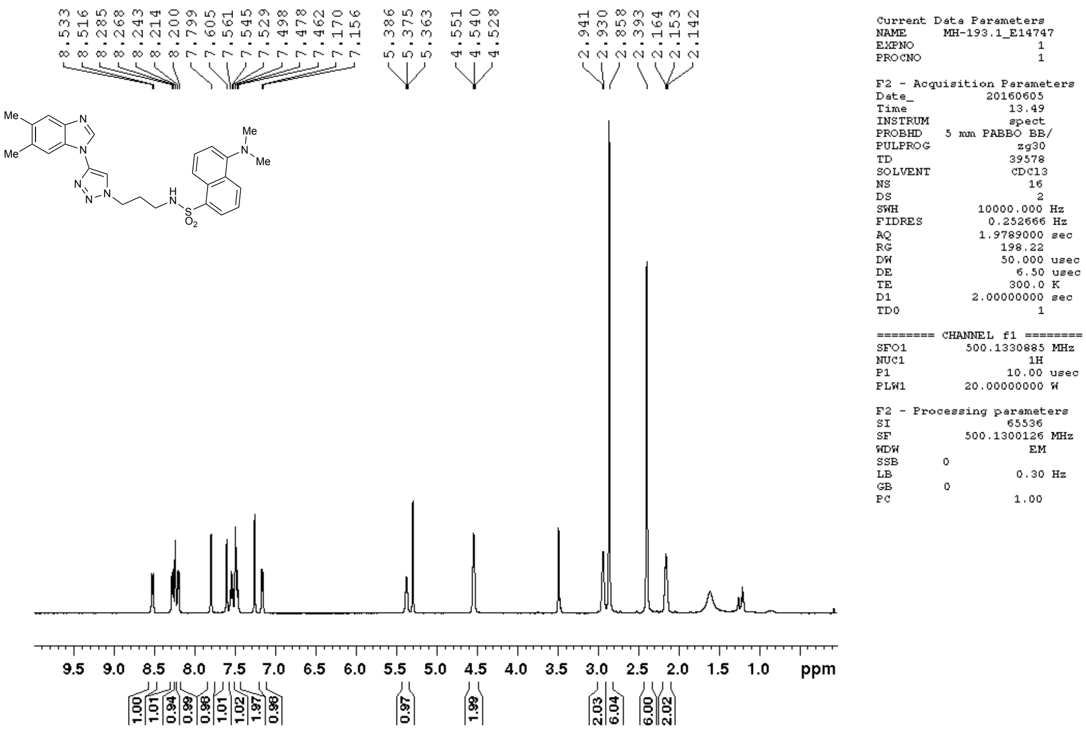

Supplementary Figure 75. ^{1}H NMR spectrum of **10a**.

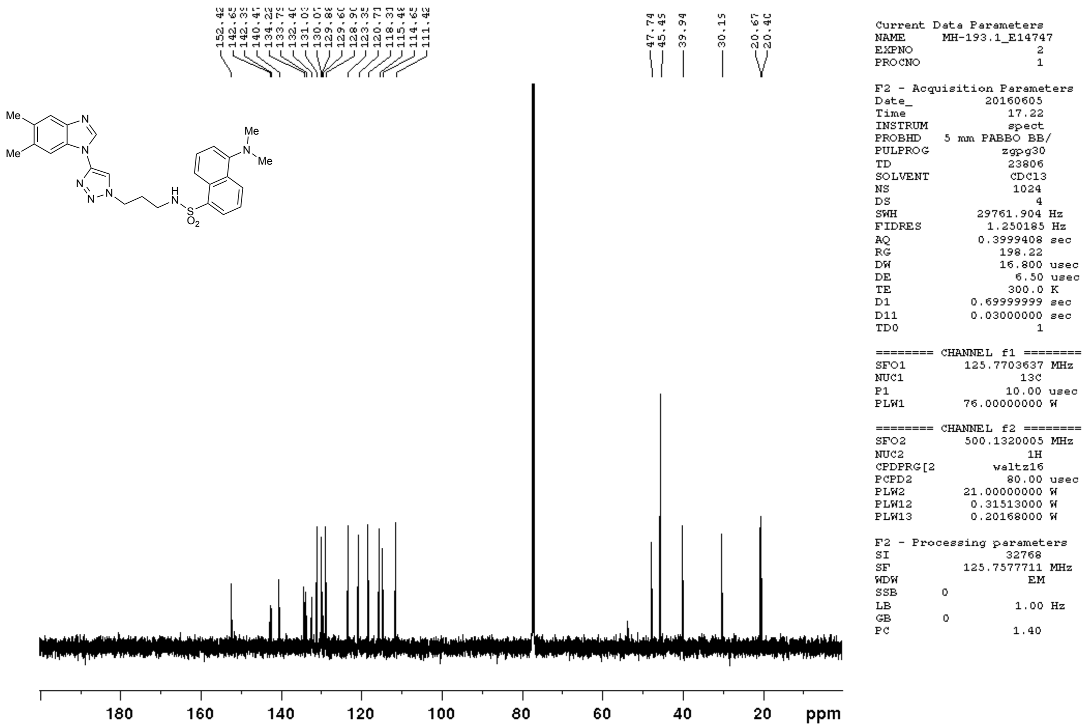

Supplementary Figure 76. ^{13}C NMR spectrum of **10a**.

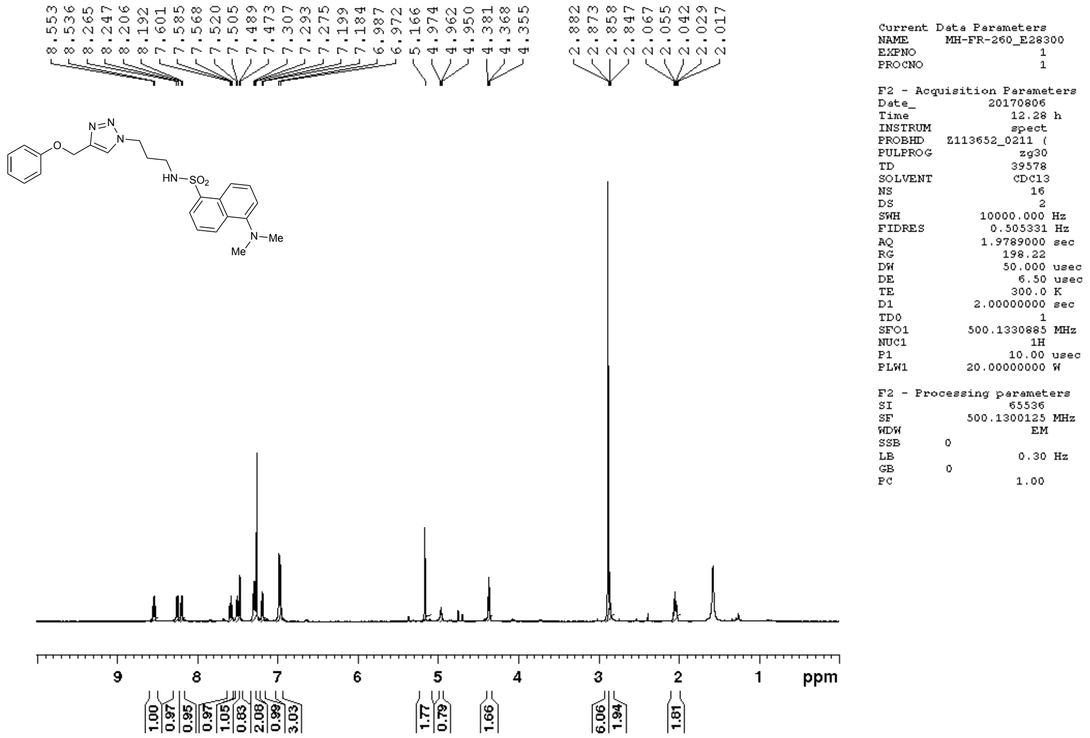

Supplementary Figure 77. ^{1}H NMR spectrum of **10b**.

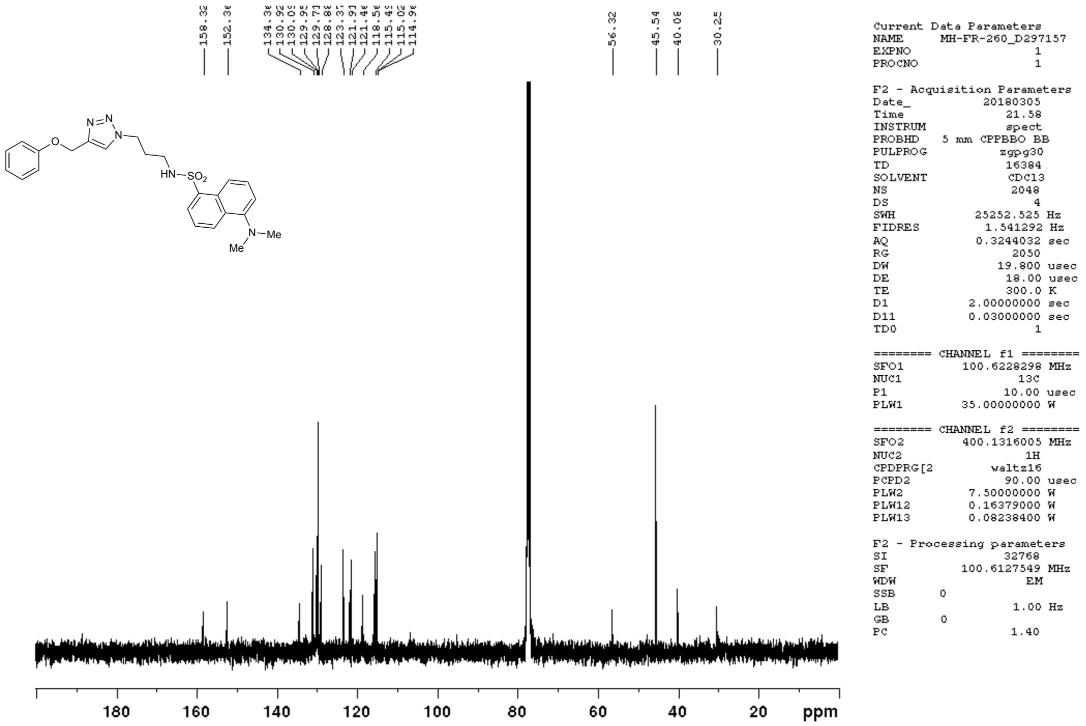

Supplementary Figure 78. ^{13}C NMR spectrum of **10b**.

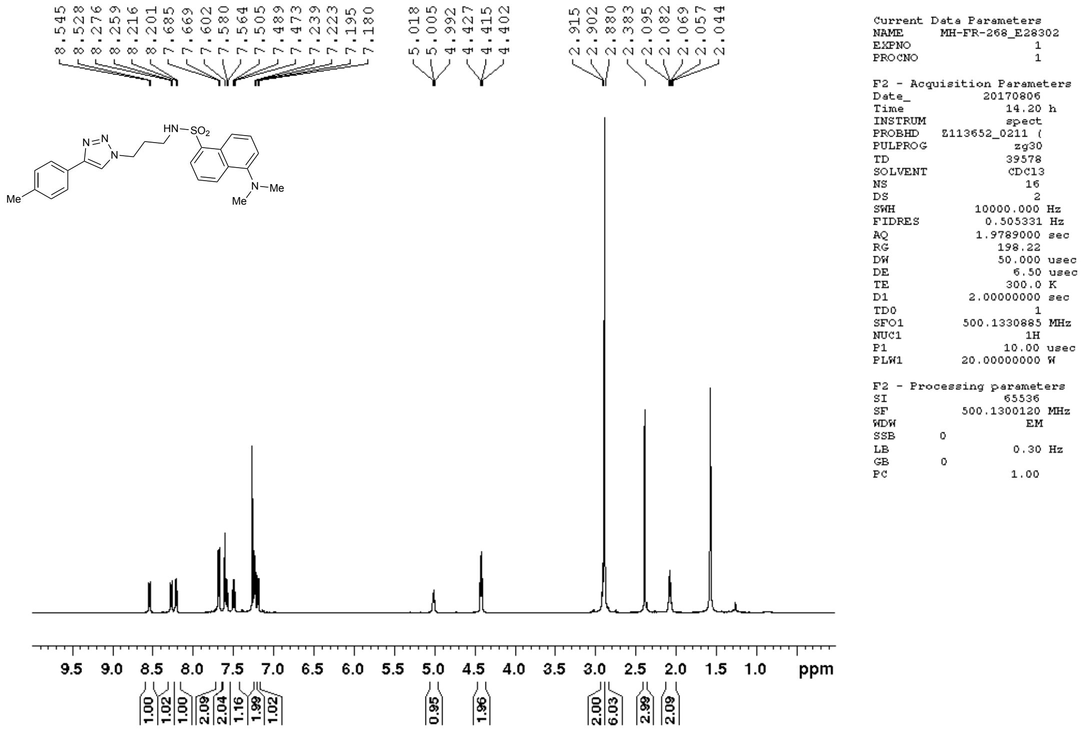

Supplementary Figure 79. ^{1}H NMR spectrum of **10c**.

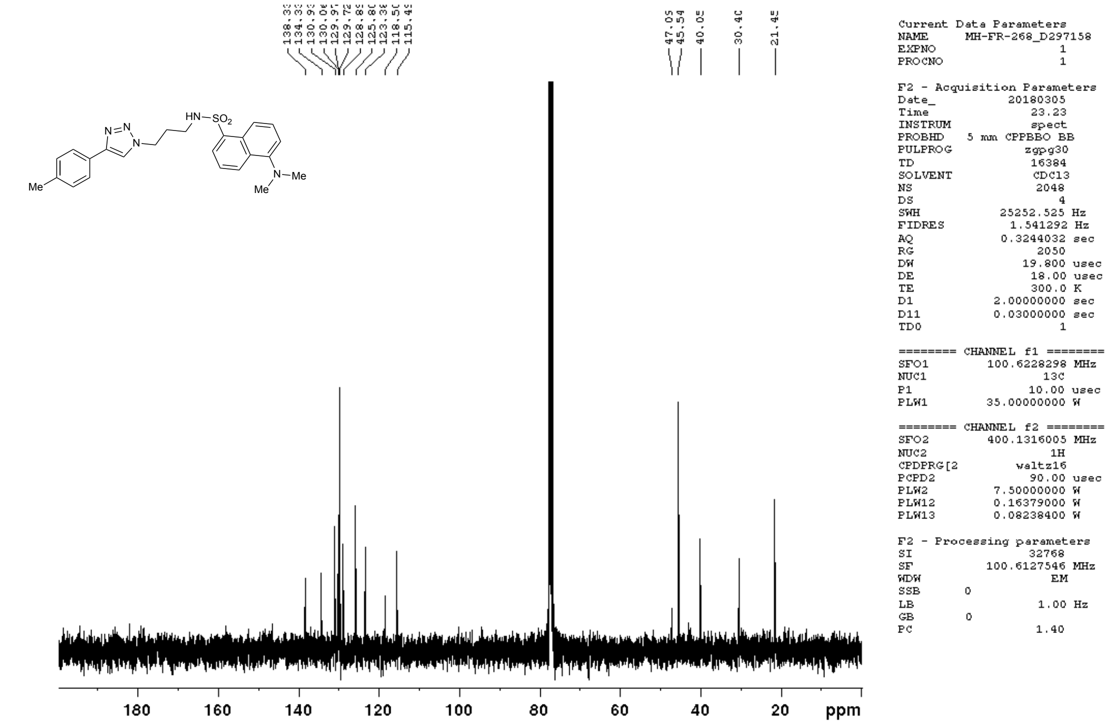

Supplementary Figure 80. ^{13}C NMR spectrum of **10c**.

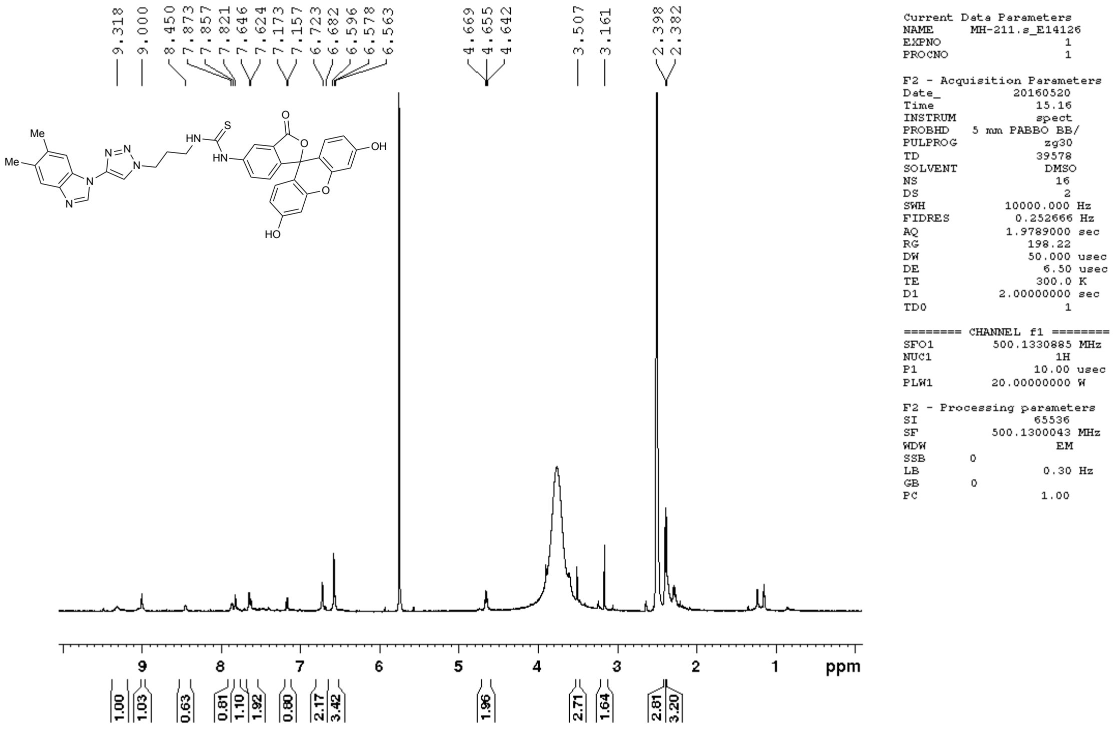

Supplementary Figure 81. ^{1}H NMR spectrum of **11a**.

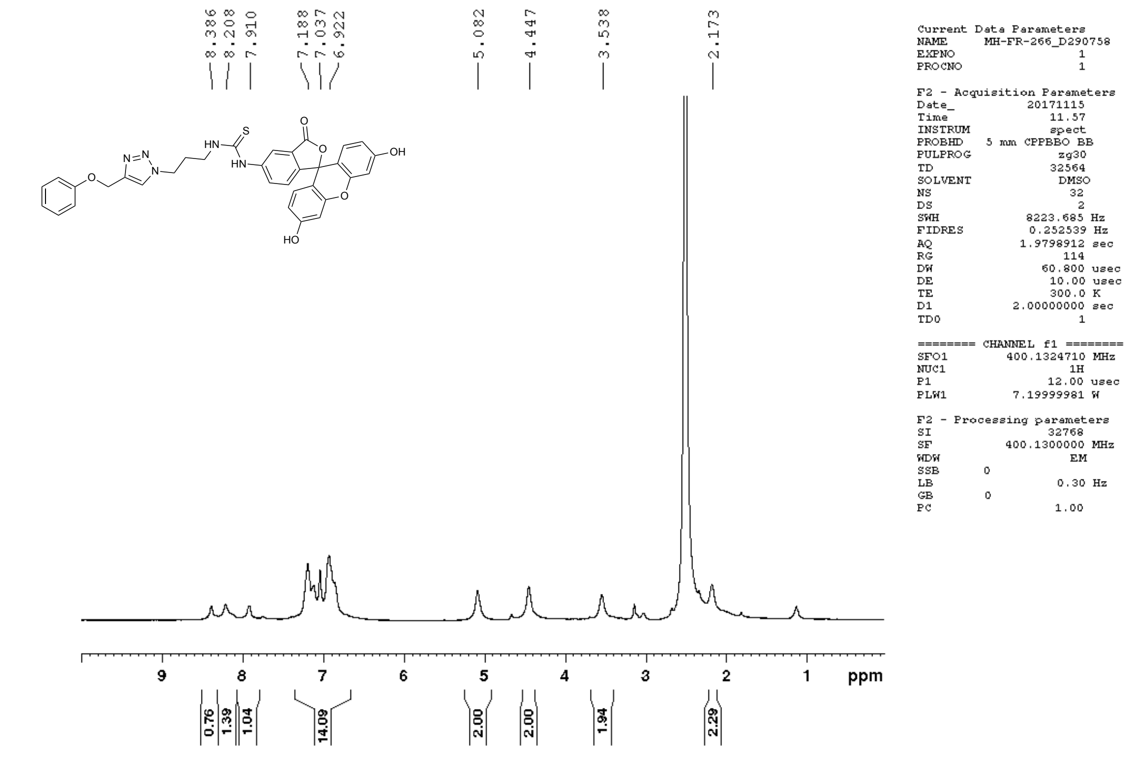

Supplementary Figure 82. ^{1}H NMR spectrum of **11b**.

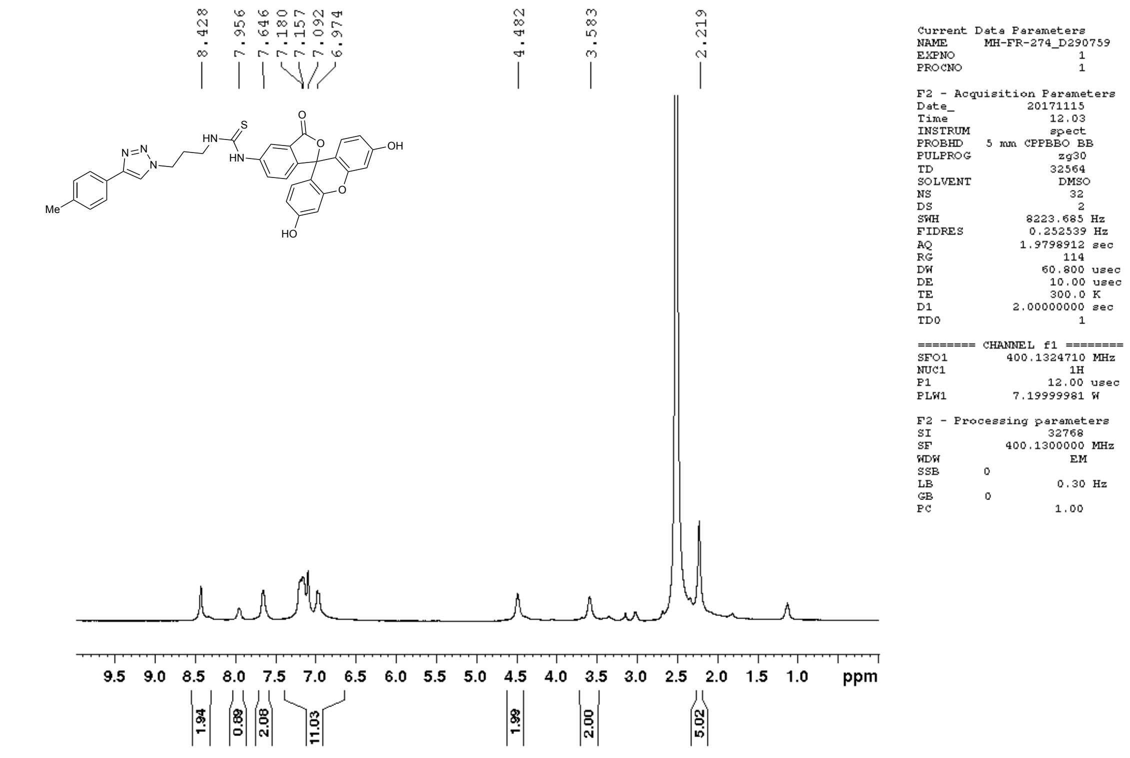

Supplementary Figure 83. ^{1}H NMR spectrum of **11c**.

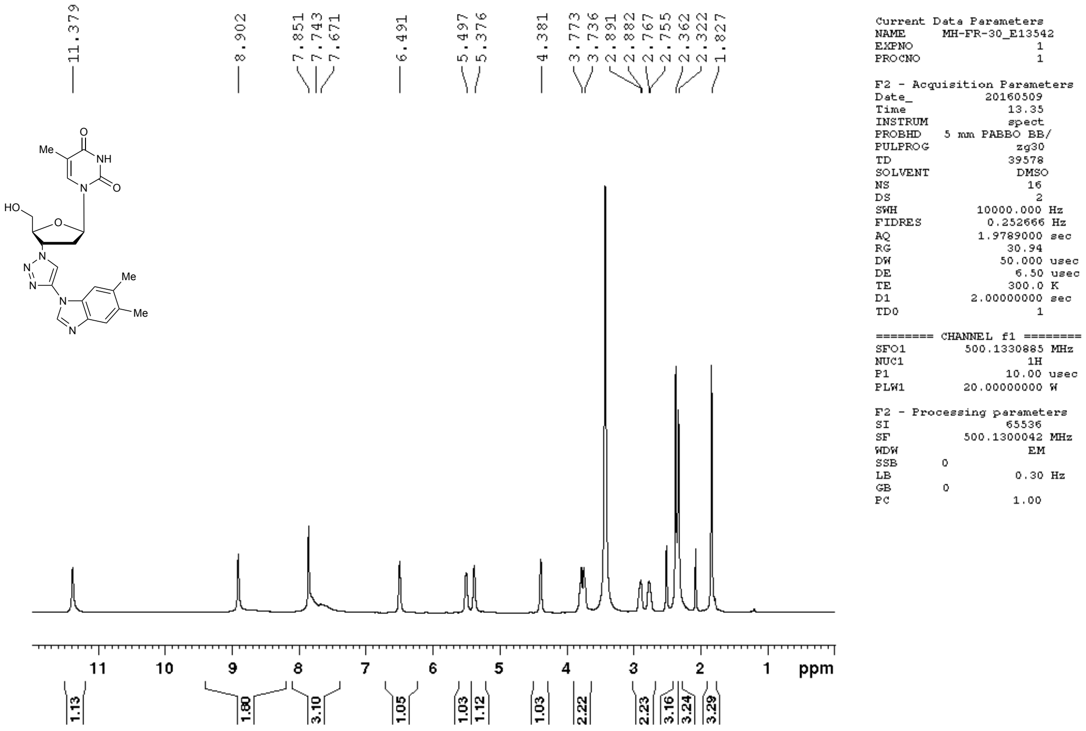

Supplementary Figure 84. ^{1}H NMR spectrum of **12a**.

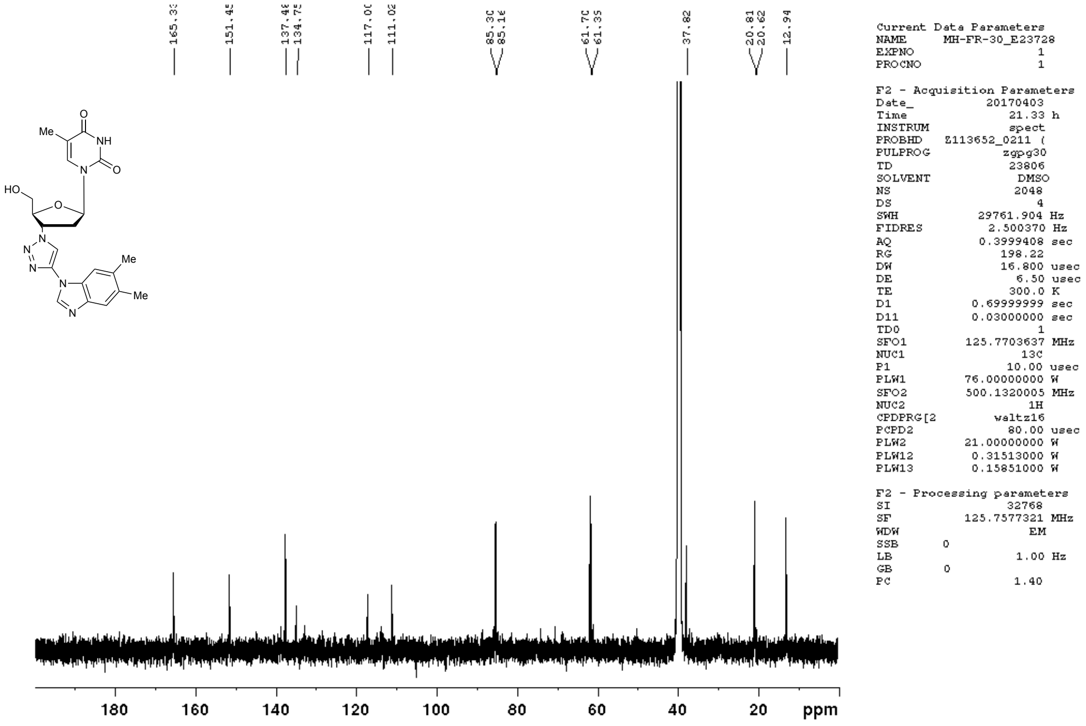

Supplementary Figure 85. ^{13}C NMR spectrum of **12a**.

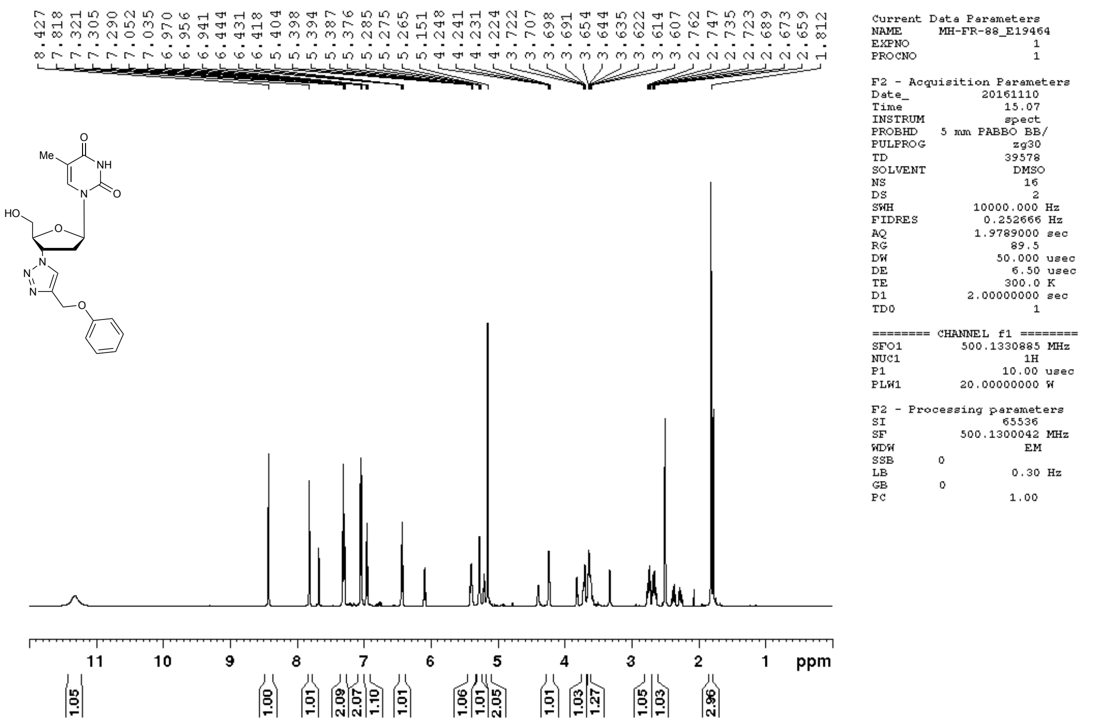

Supplementary Figure 86. ^{1}H NMR spectrum of **12b**.

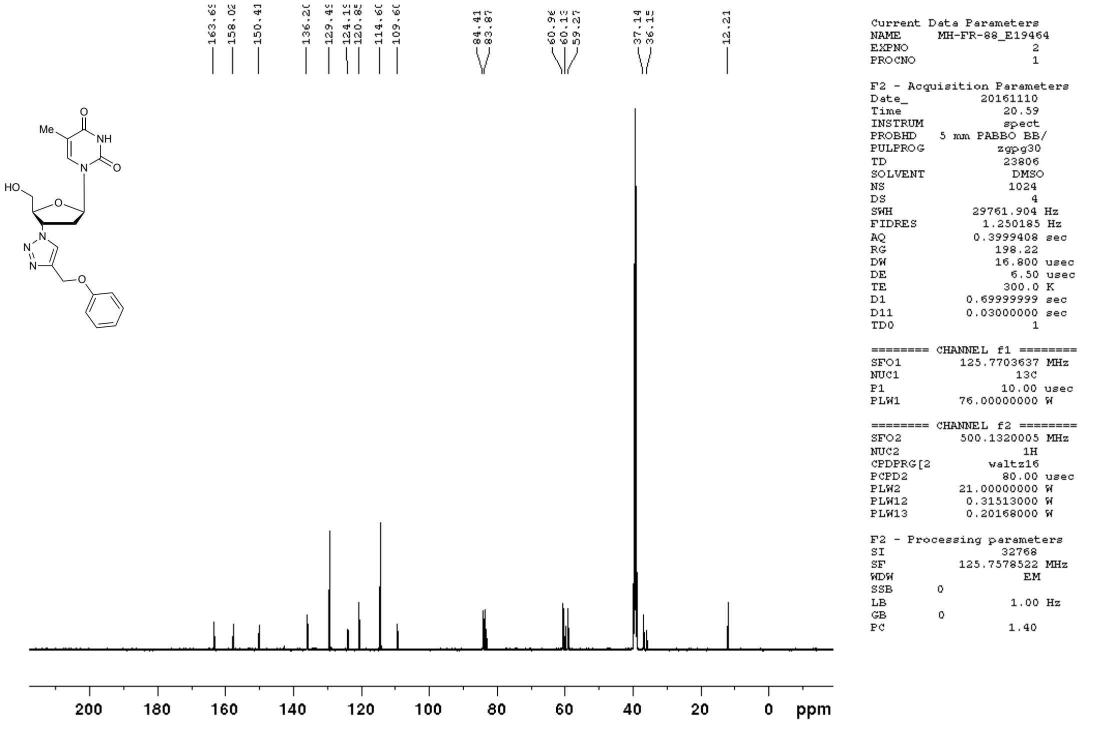

Supplementary Figure 87. ^{13}C NMR spectrum of **12b**.

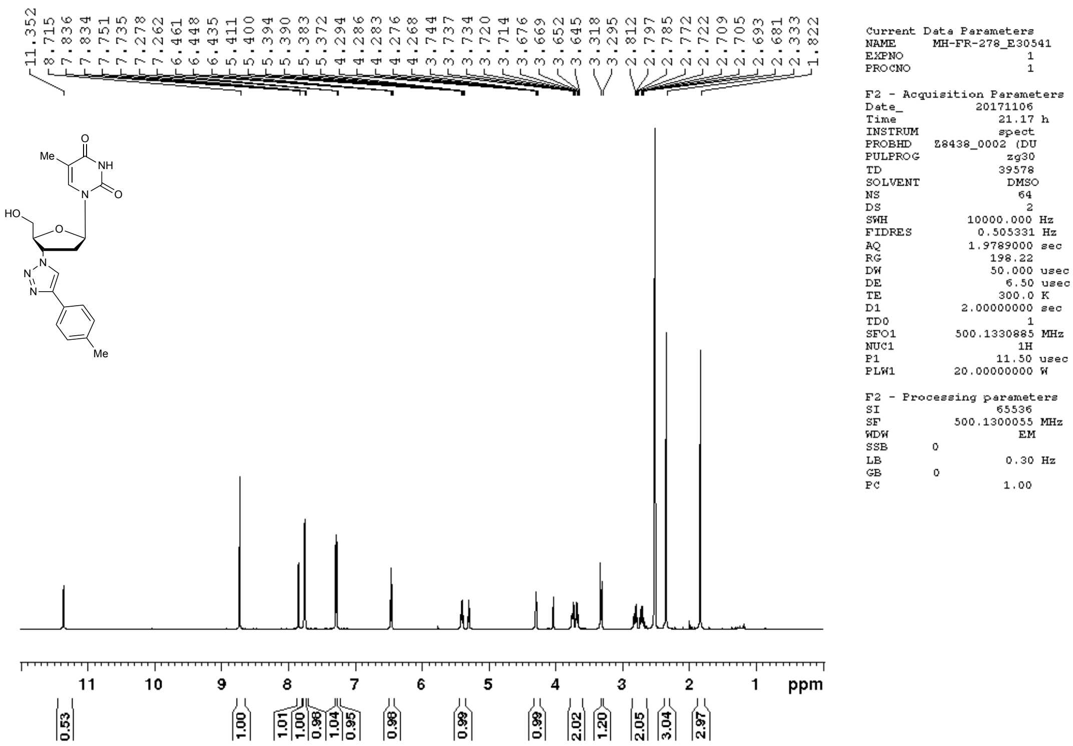

Supplementary Figure 88. ^{1}H NMR spectrum of **12c**.

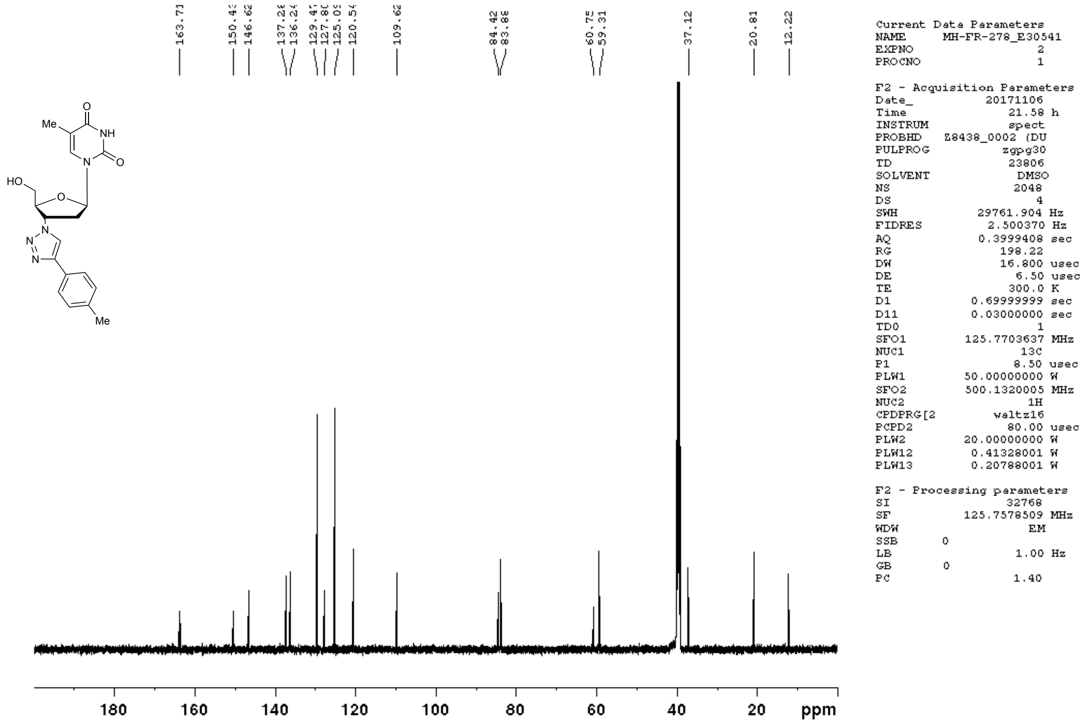

Supplementary Figure 89. ^{13}C NMR spectrum of **12c**.

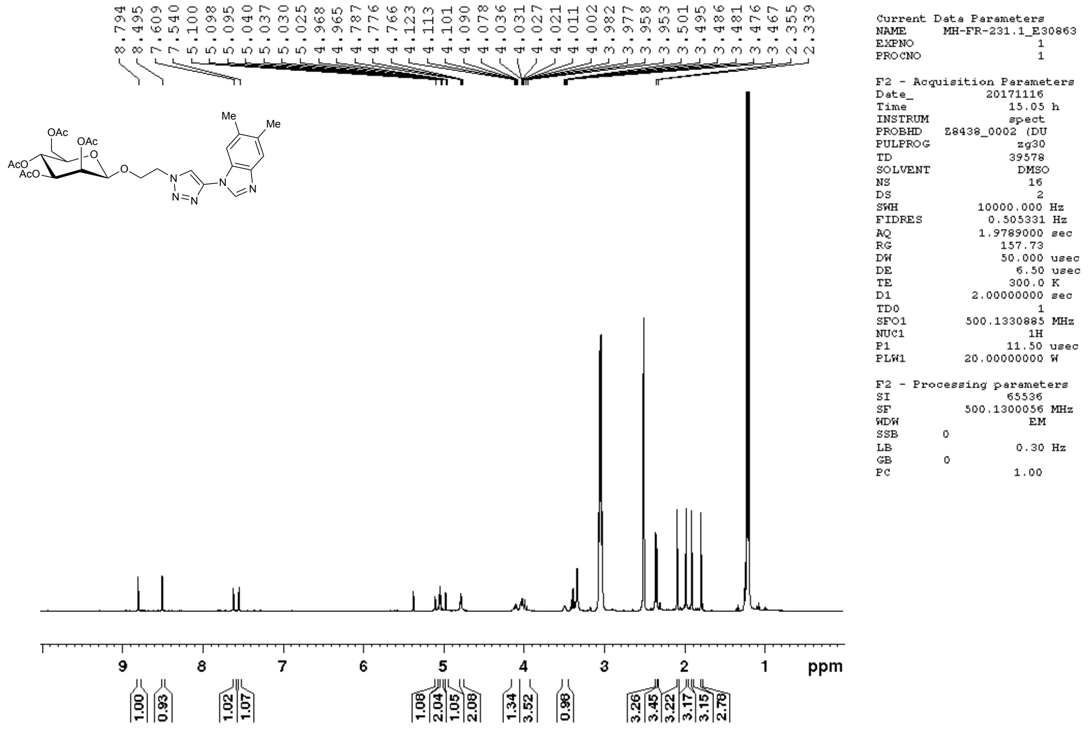

Supplementary Figure 90. ^{1}H NMR spectrum of **13a**.

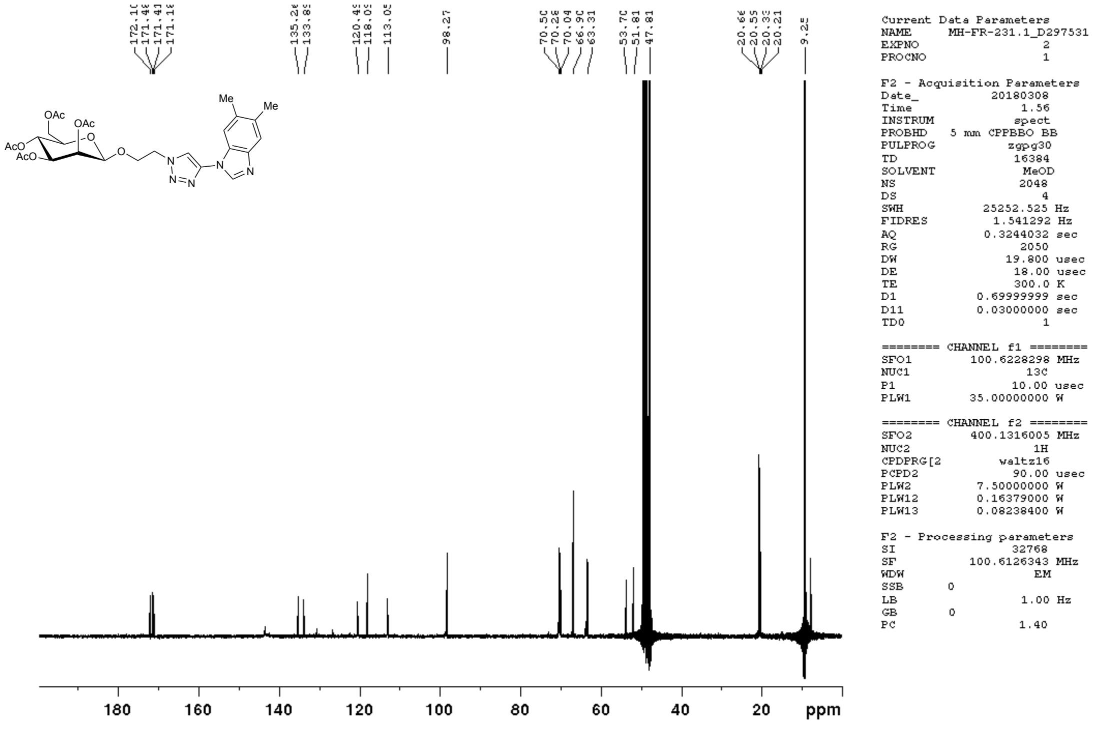

Supplementary Figure 91. ^{13}C NMR spectrum of **13a**.

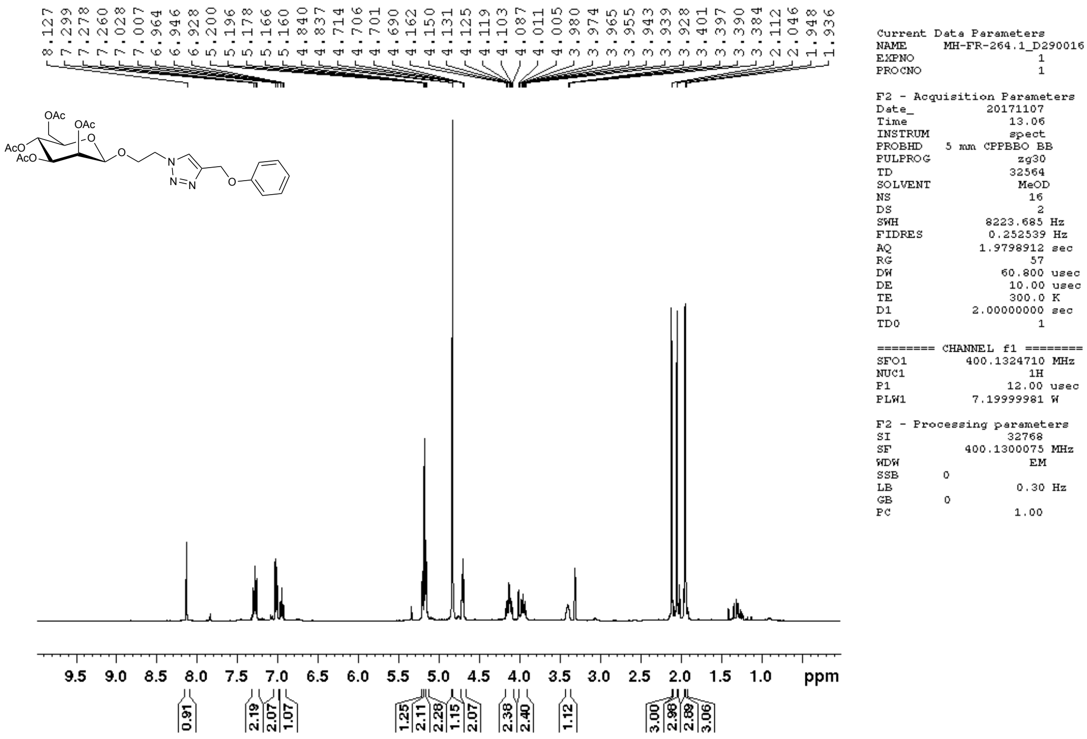

Supplementary Figure 92. ^{1}H NMR spectrum of **13b**.

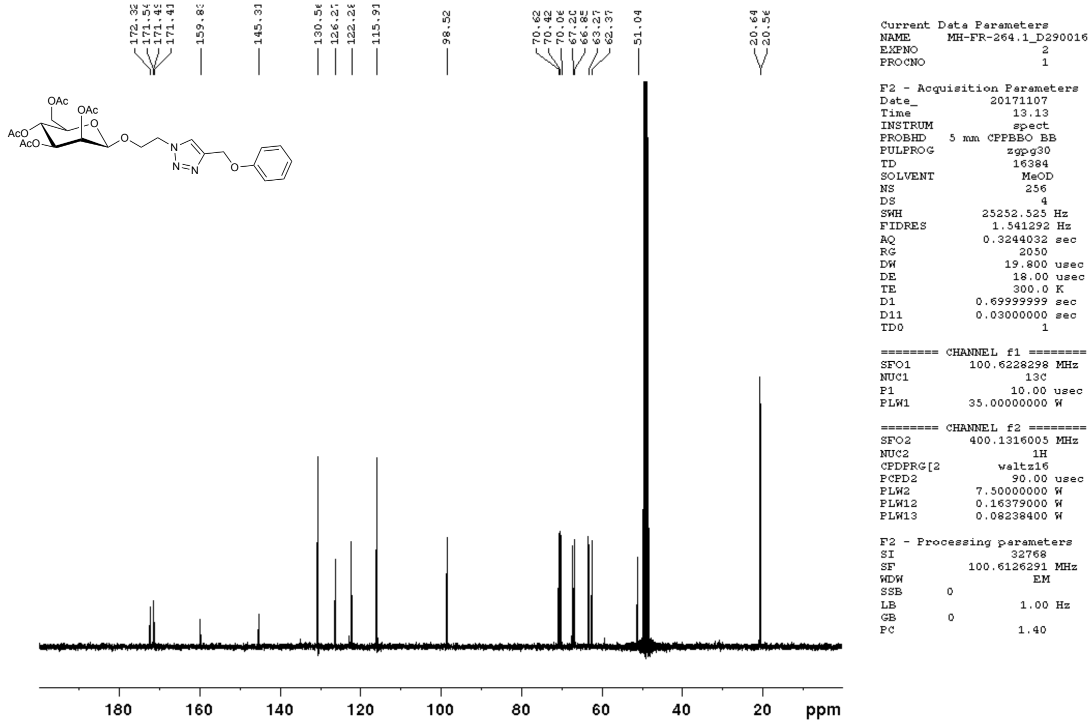

Supplementary Figure 93. ^{13}C NMR spectrum of **13b**.

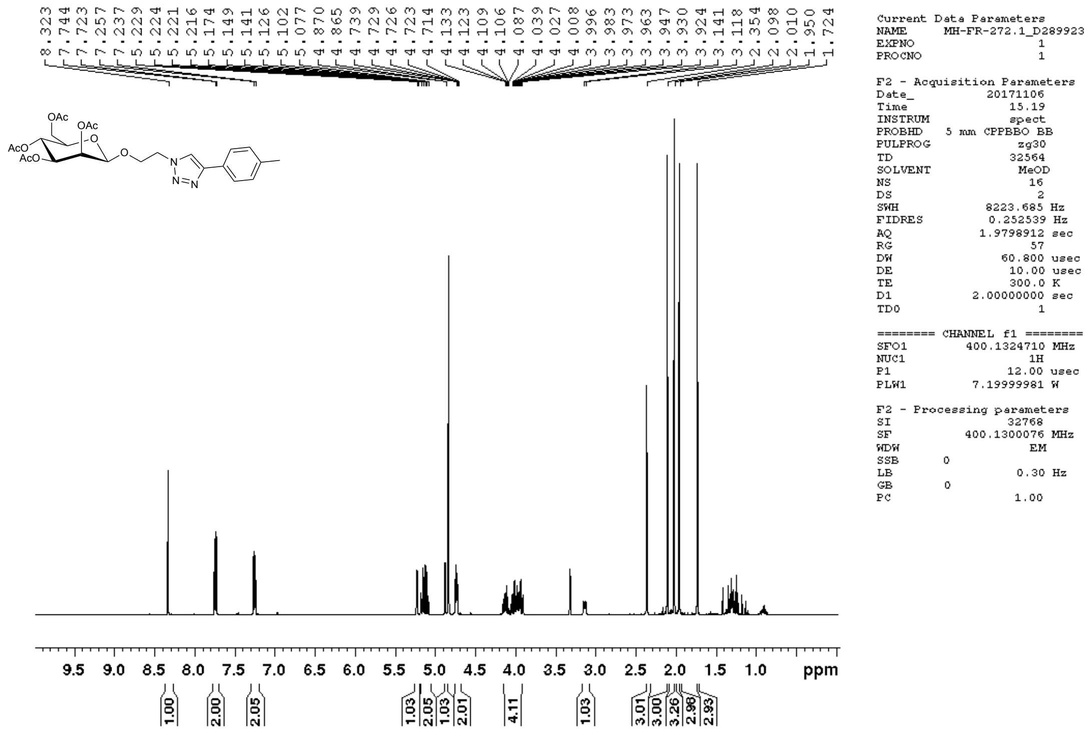

Supplementary Figure 94. ^{1}H NMR spectrum of **13c**.

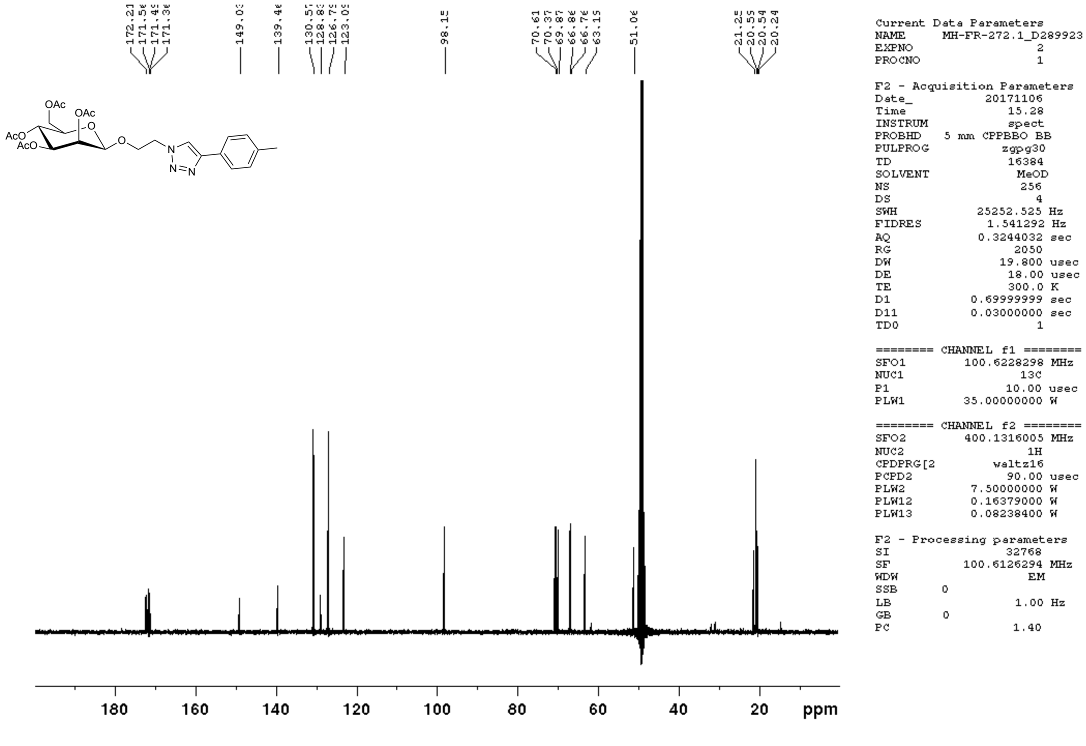

Supplementary Figure 95. ^{13}C NMR spectrum of **13c**.

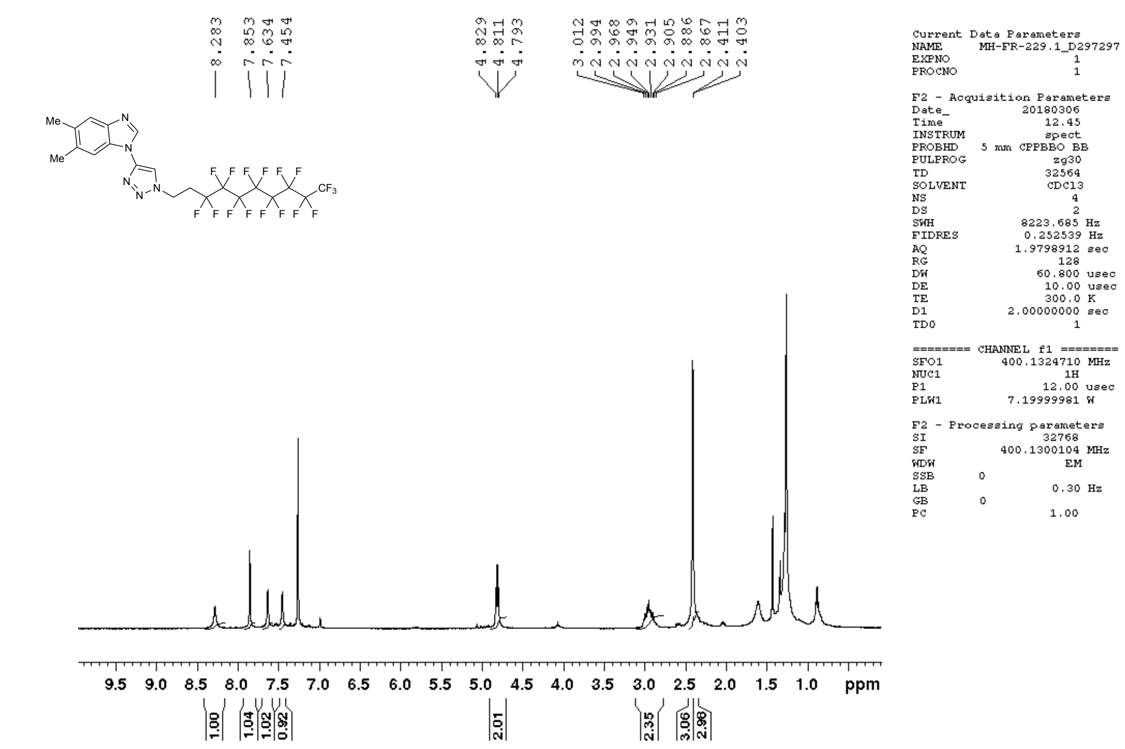

Supplementary Figure 96. ^{1}H NMR spectrum of **14a**.

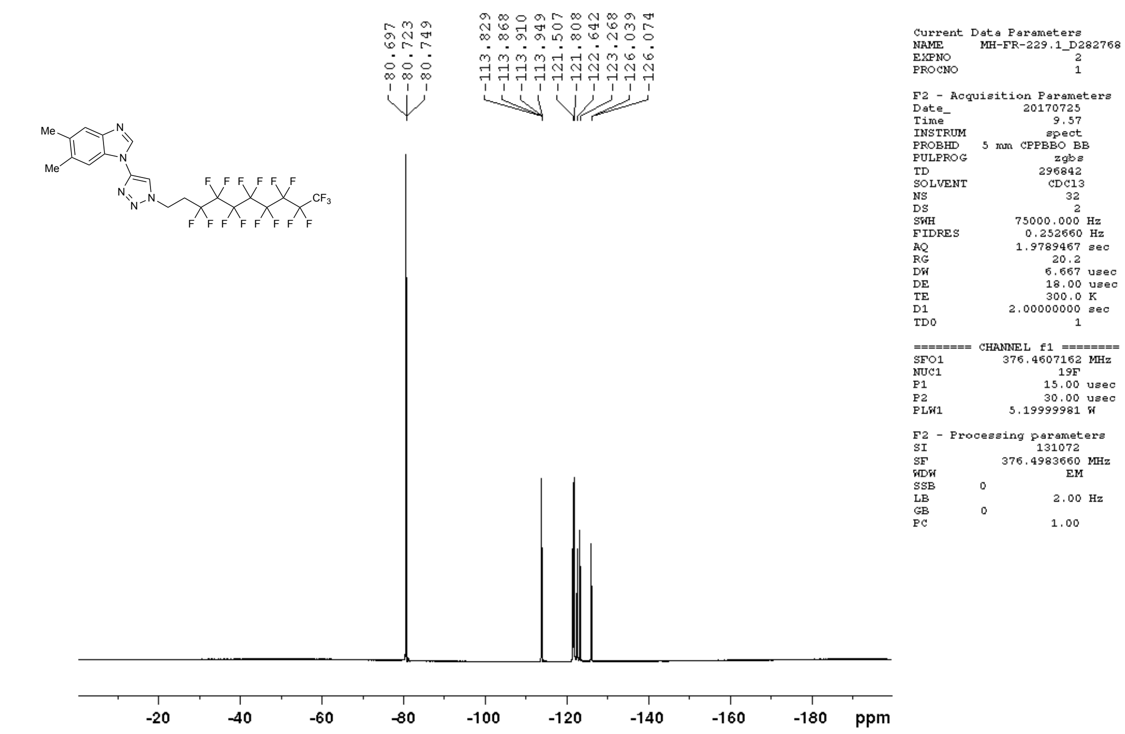

Supplementary Figure 97. ^{19}F NMR spectrum of **14a**.

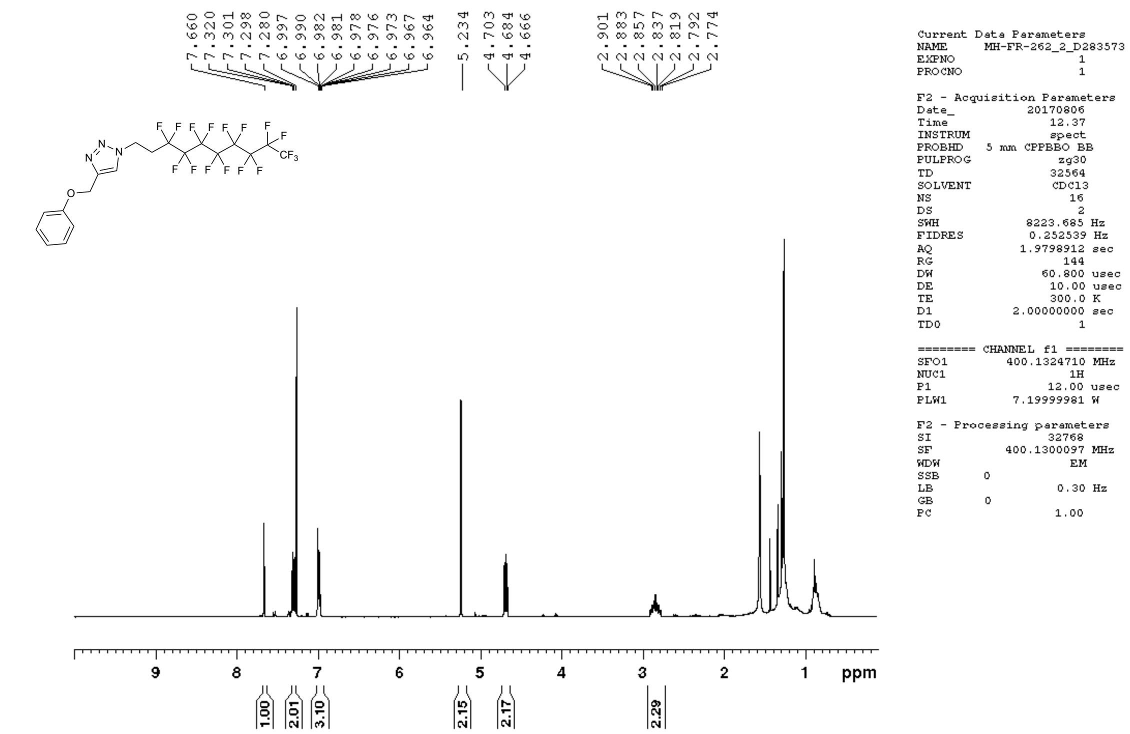

Supplementary Figure 98. ^{1}H NMR spectrum of **14b**.

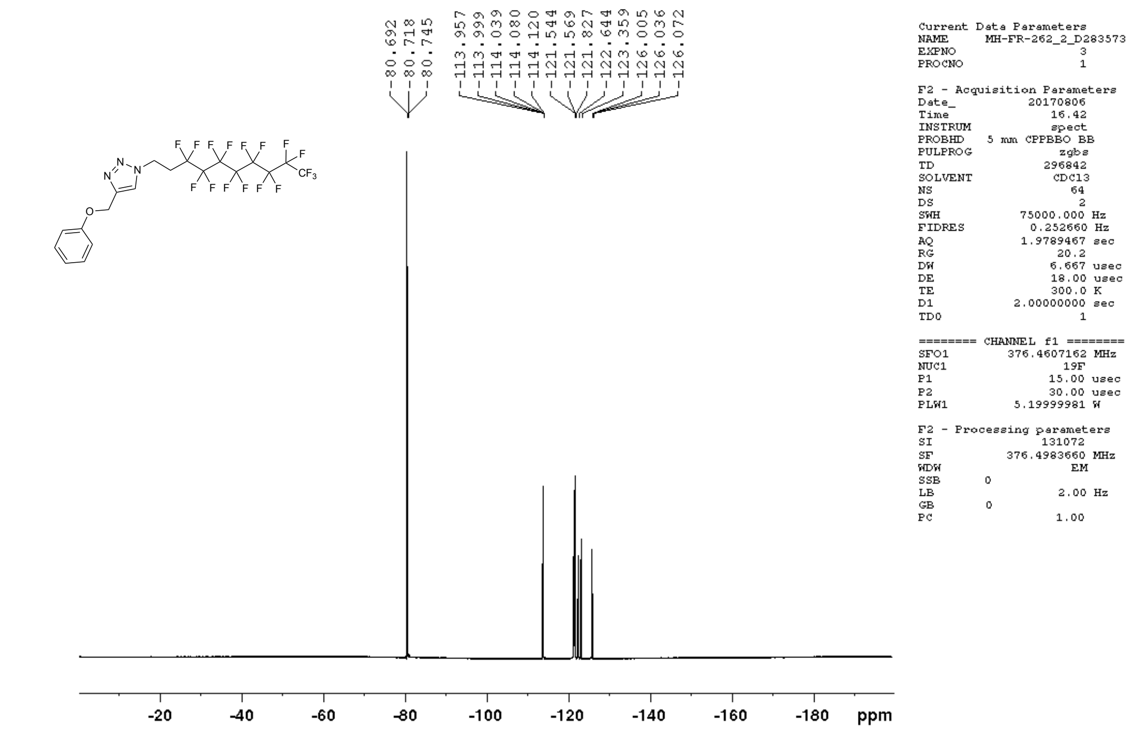

Supplementary Figure 99. ^{19}F NMR spectrum of **14b**.

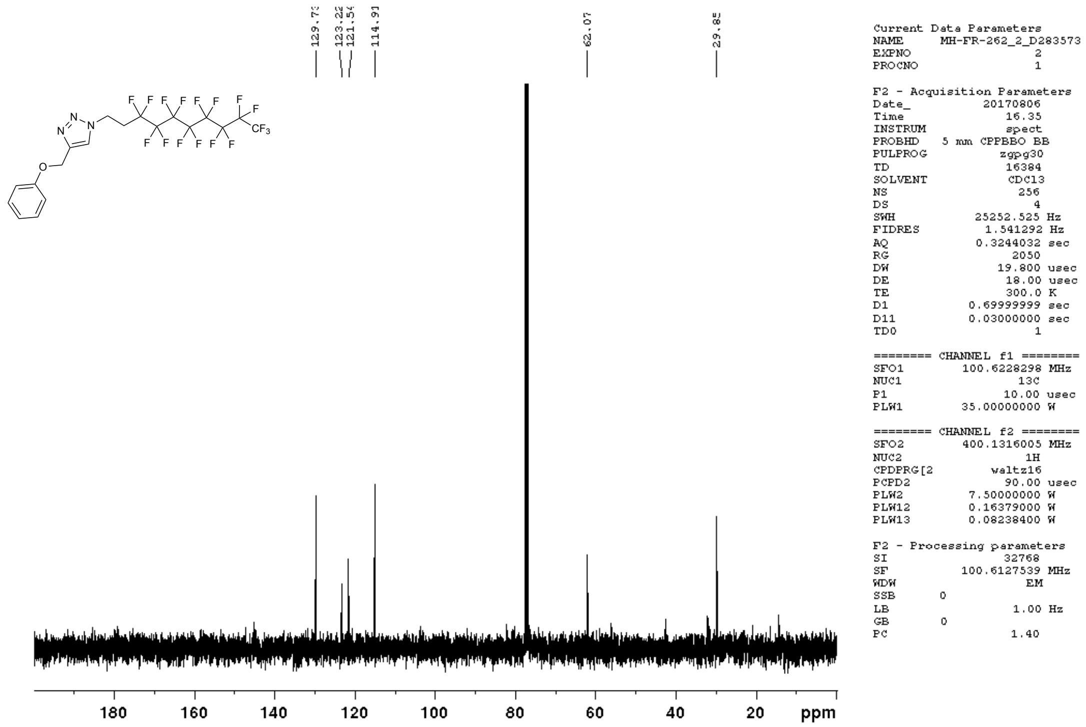

Supplementary Figure 100. ^{13}C NMR spectrum of **14b**.

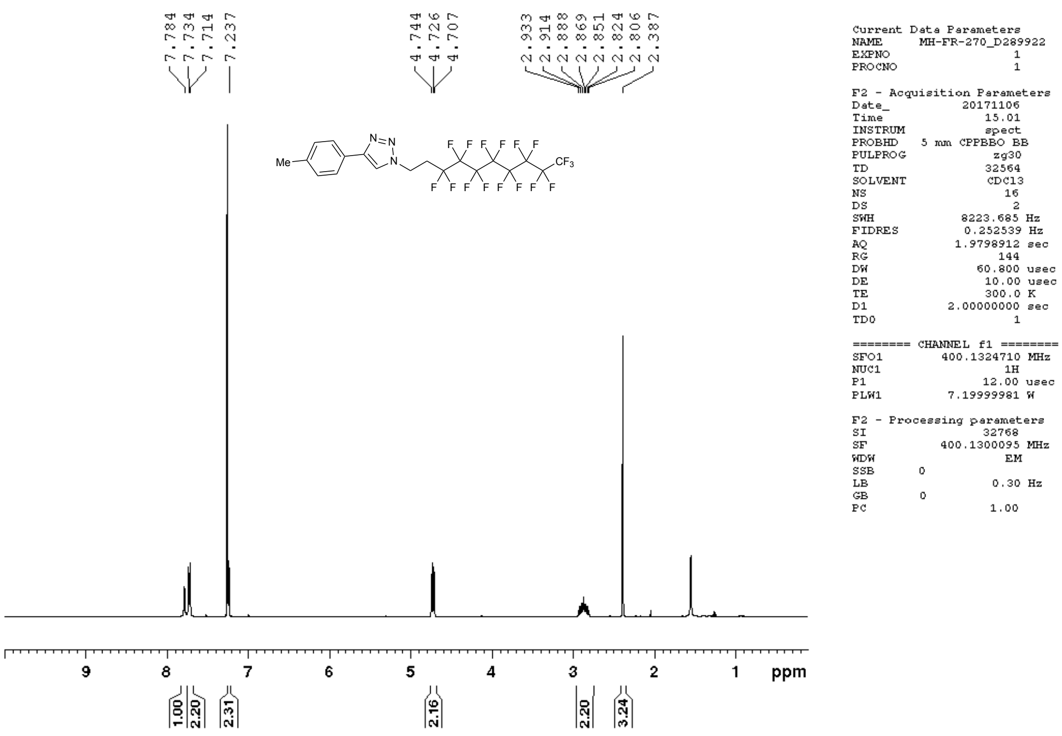

Supplementary Figure 101. ^{1}H NMR spectrum of **14c**.

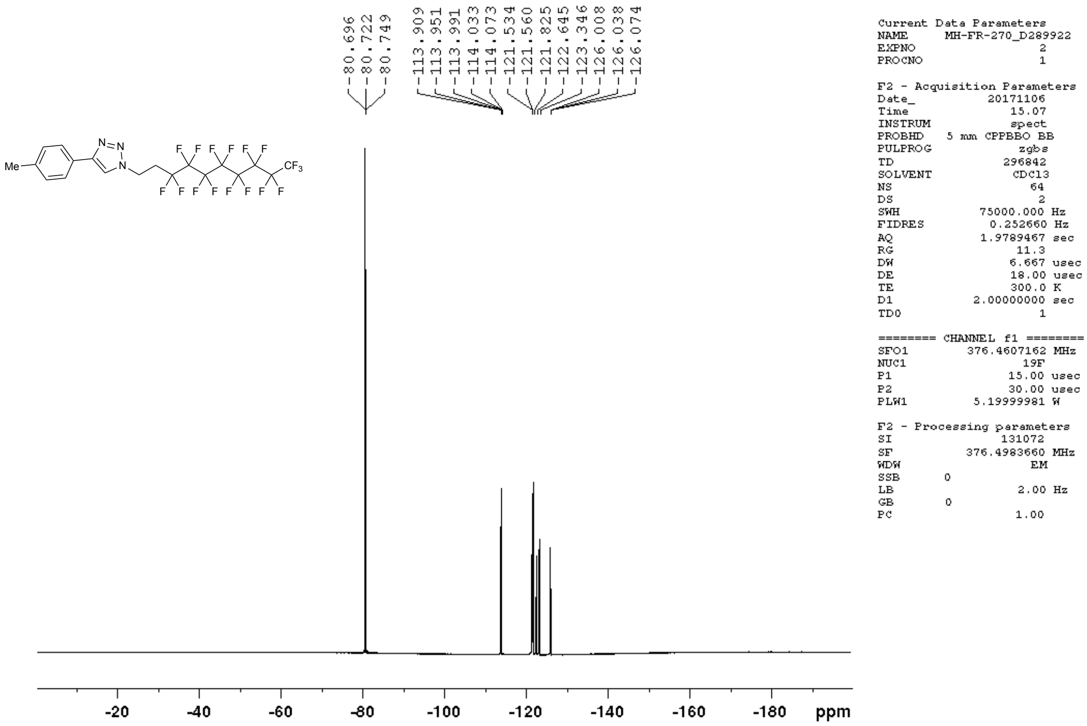

Supplementary Figure 102. ^{19}F NMR spectrum of **14c**.

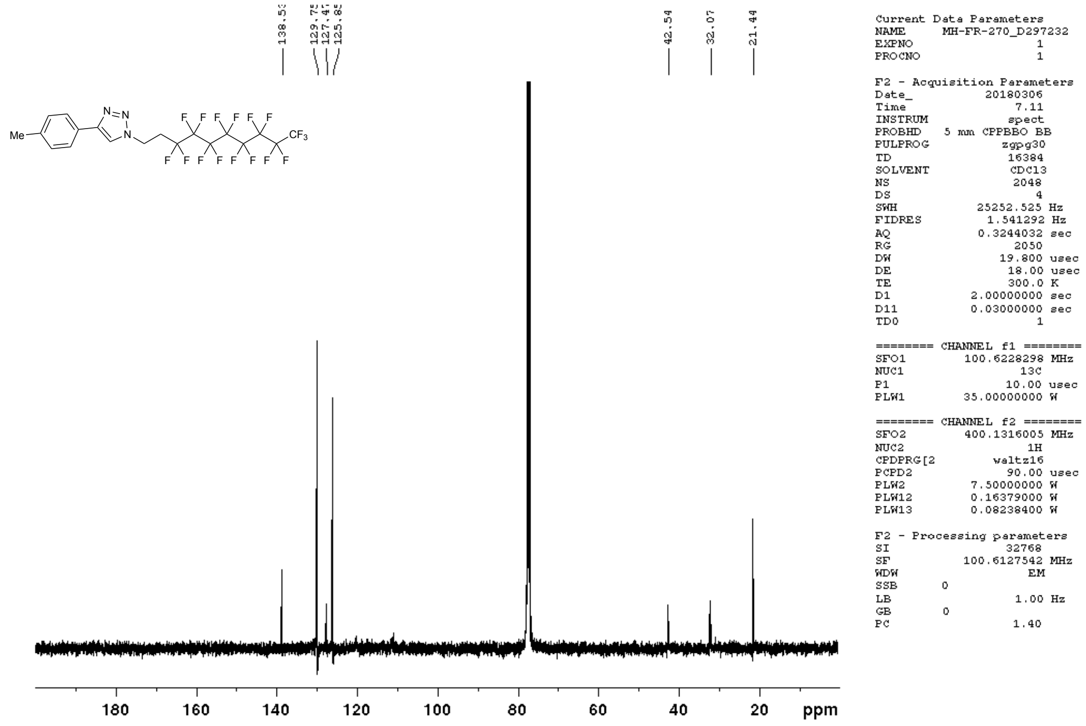

Supplementary Figure 103. ^{13}C NMR spectrum of **14c**.

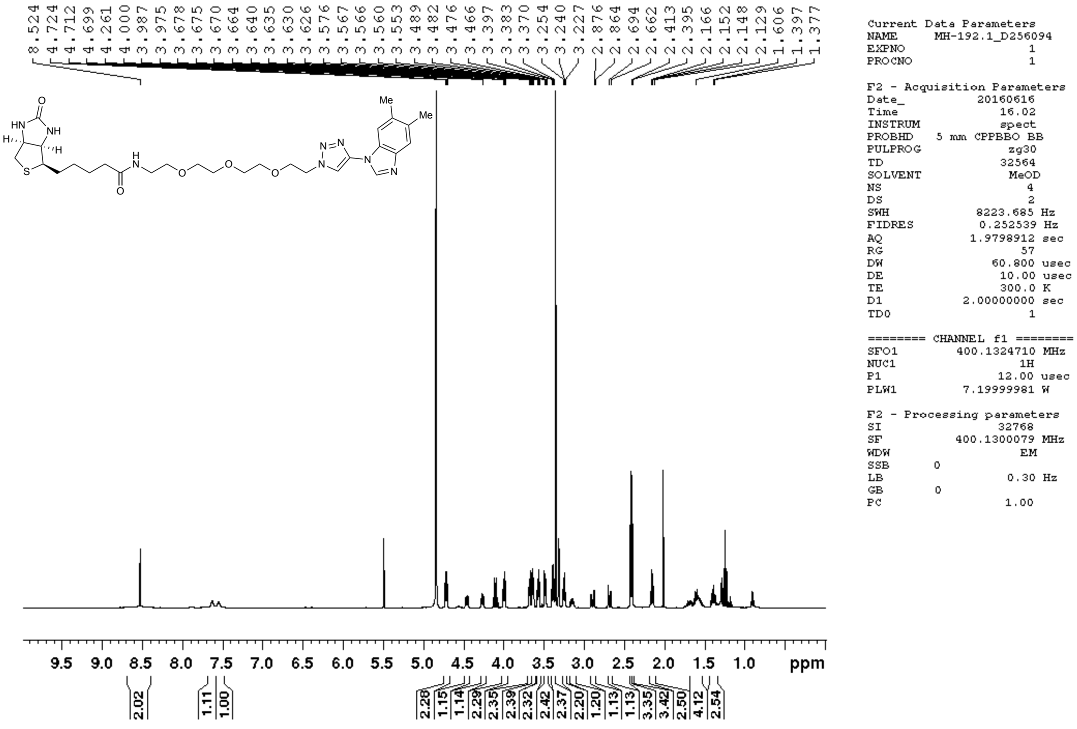

Supplementary Figure 104. ^{1}H NMR spectrum of **15a**.

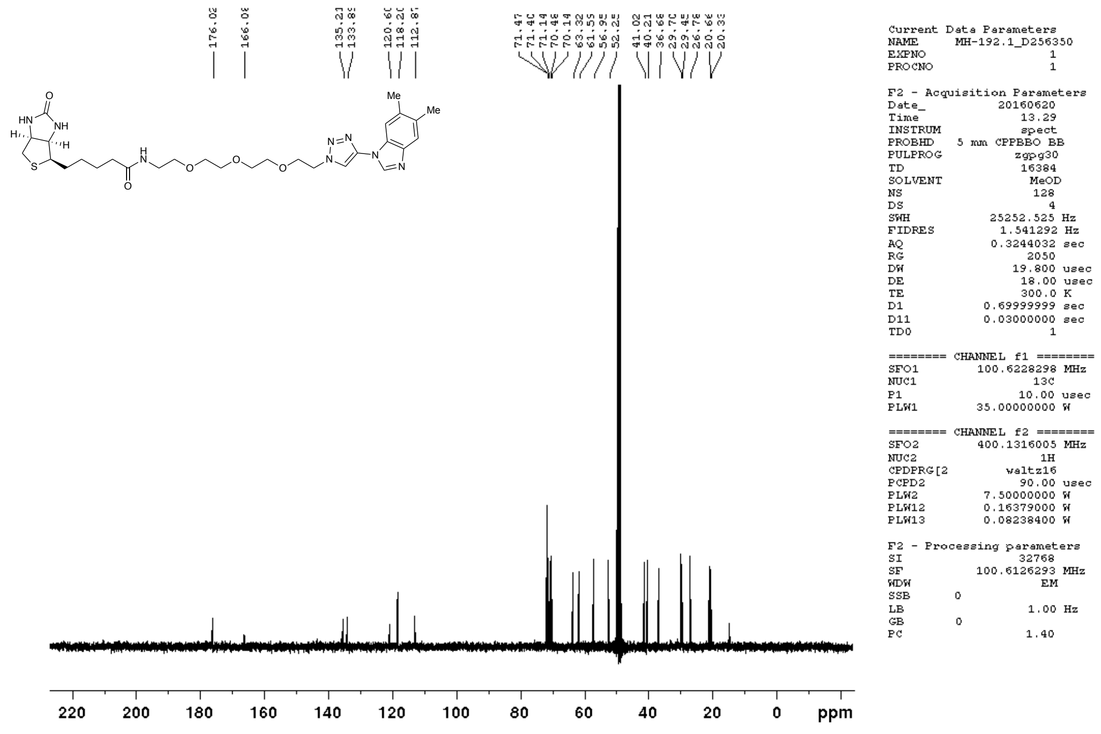

Supplementary Figure 105. ^{13}C NMR spectrum of **15a**.

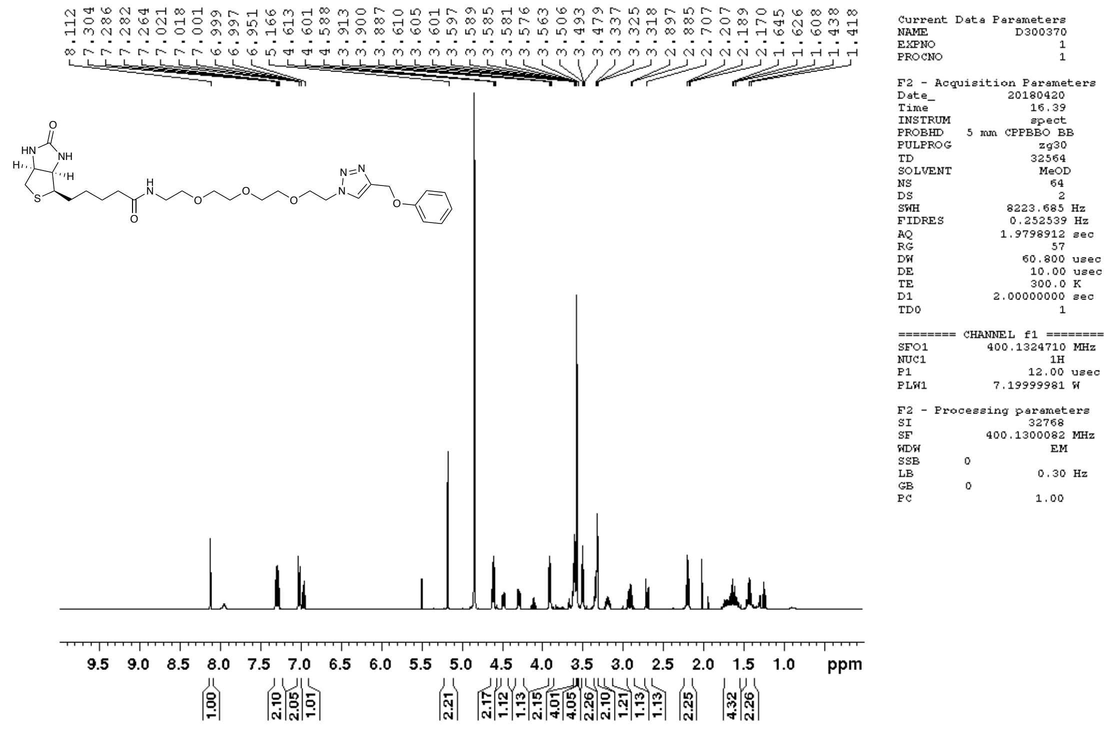

Supplementary Figure 106. ^{1}H NMR spectrum of **15b**.

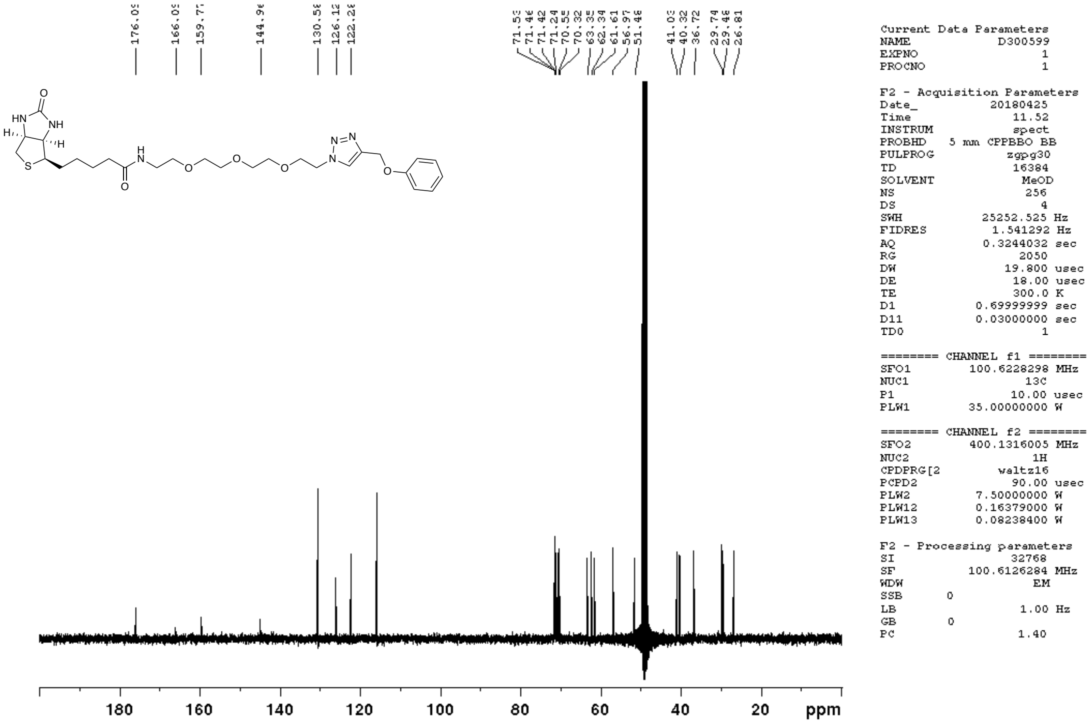

Supplementary Figure 107. ^{13}C NMR spectrum of **15b**.

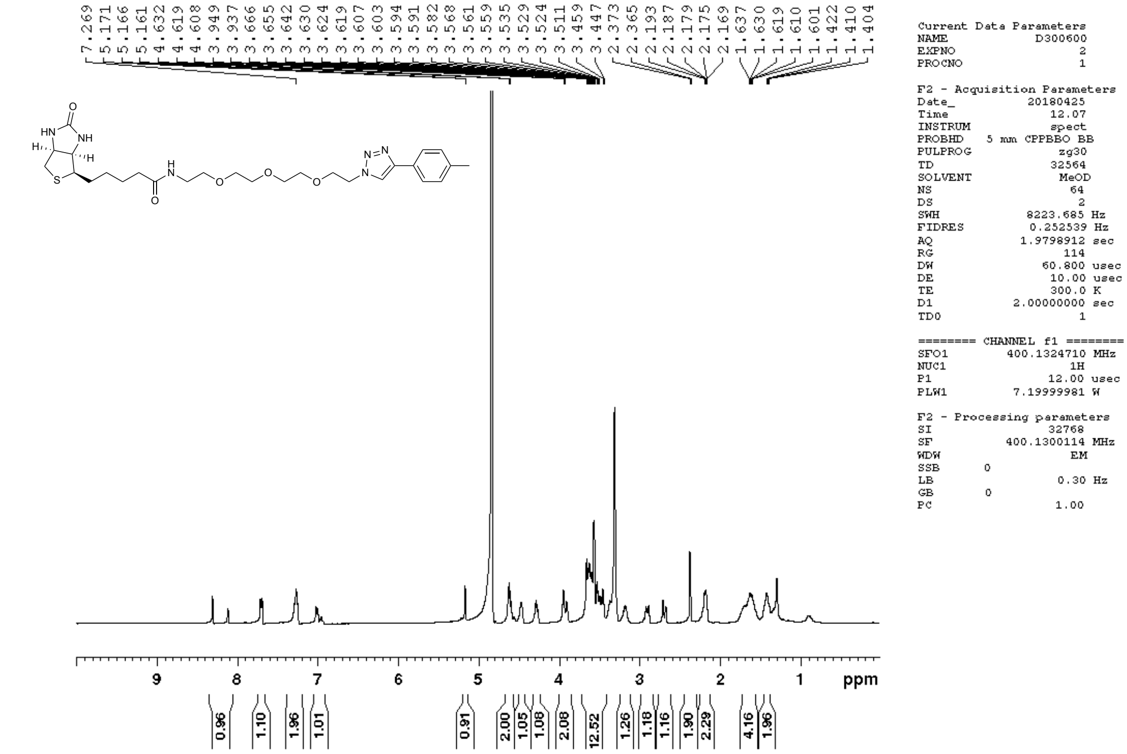

Supplementary Figure 108. ^{1}H NMR spectrum of **15c**.

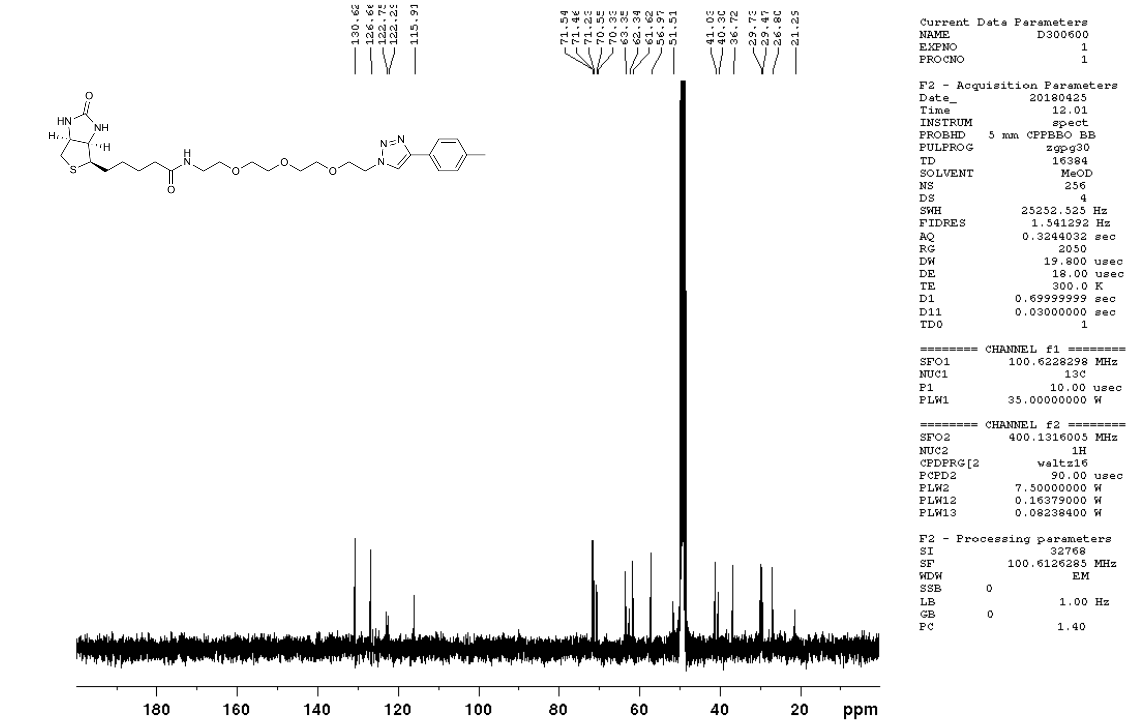

Supplementary Figure 109. ^{13}C NMR spectrum of **15c**.

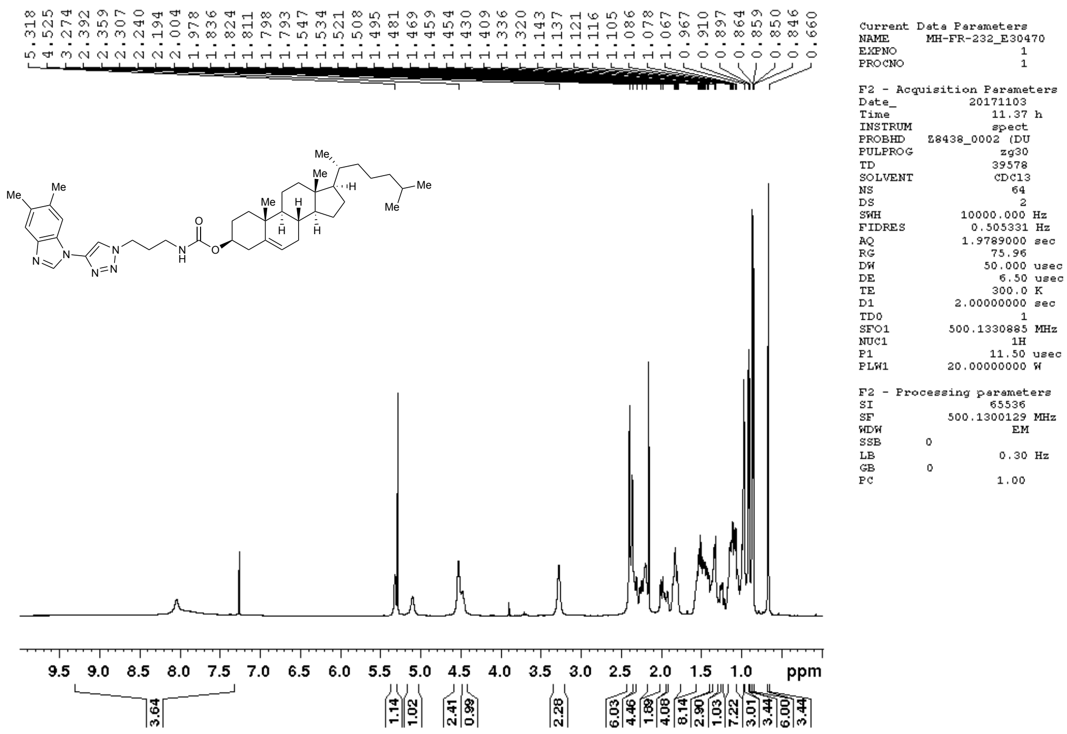

Supplementary Figure 110. ^{1}H NMR spectrum of **17a**.

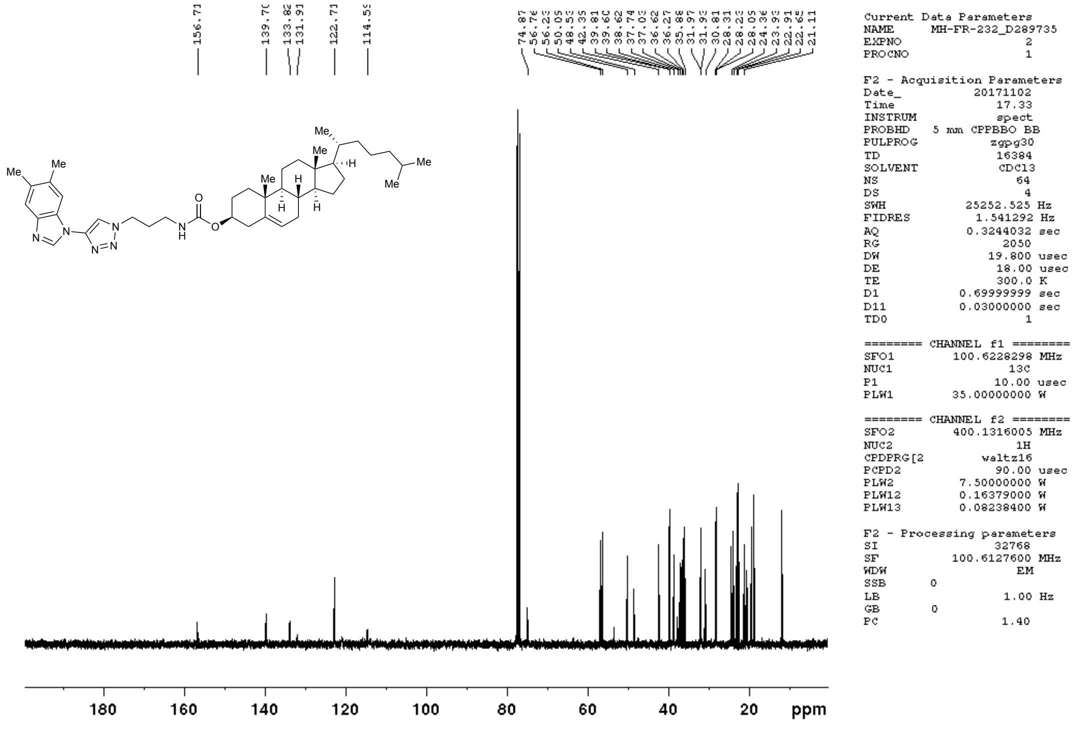

Supplementary Figure 111. ^{13}C NMR spectrum of **17a**.

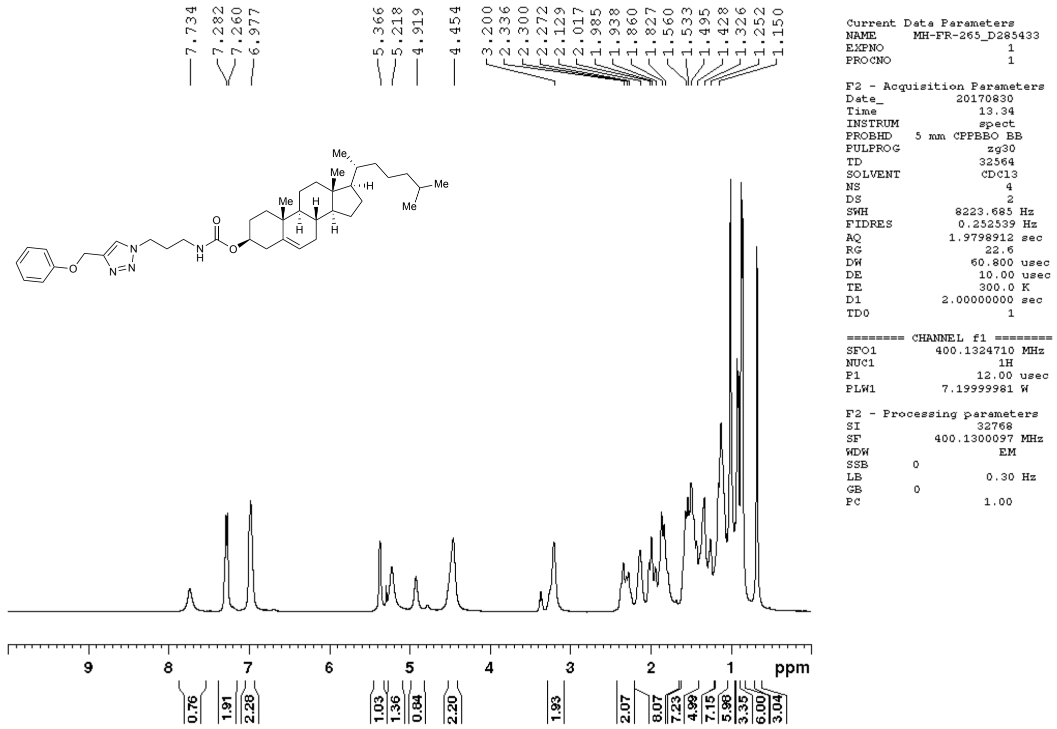

Supplementary Figure 112. ^{1}H NMR spectrum of **17b**.

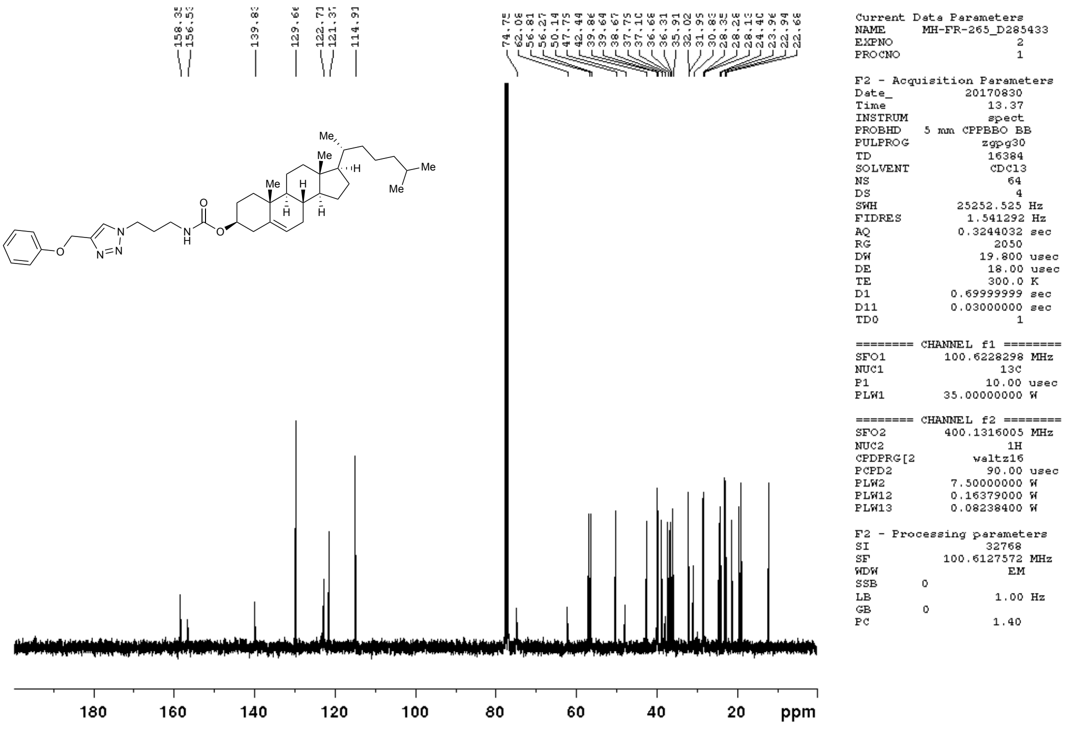

Supplementary Figure 113. ^{13}C NMR spectrum of **17b**.

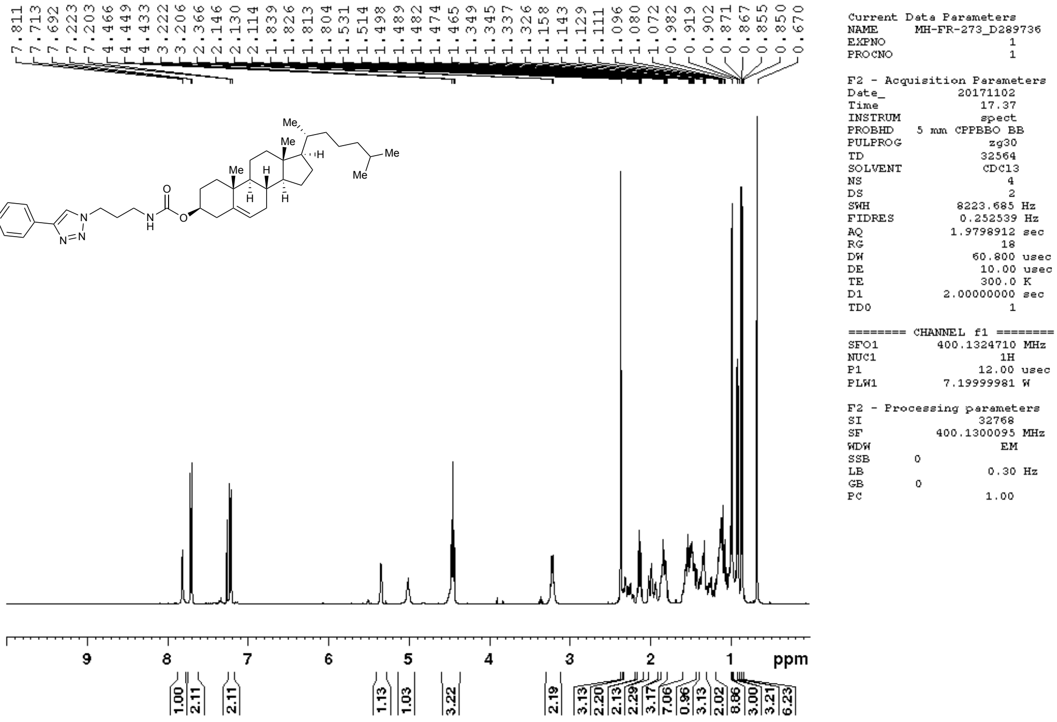

Supplementary Figure 114. ^{1}H NMR spectrum of **17c**.

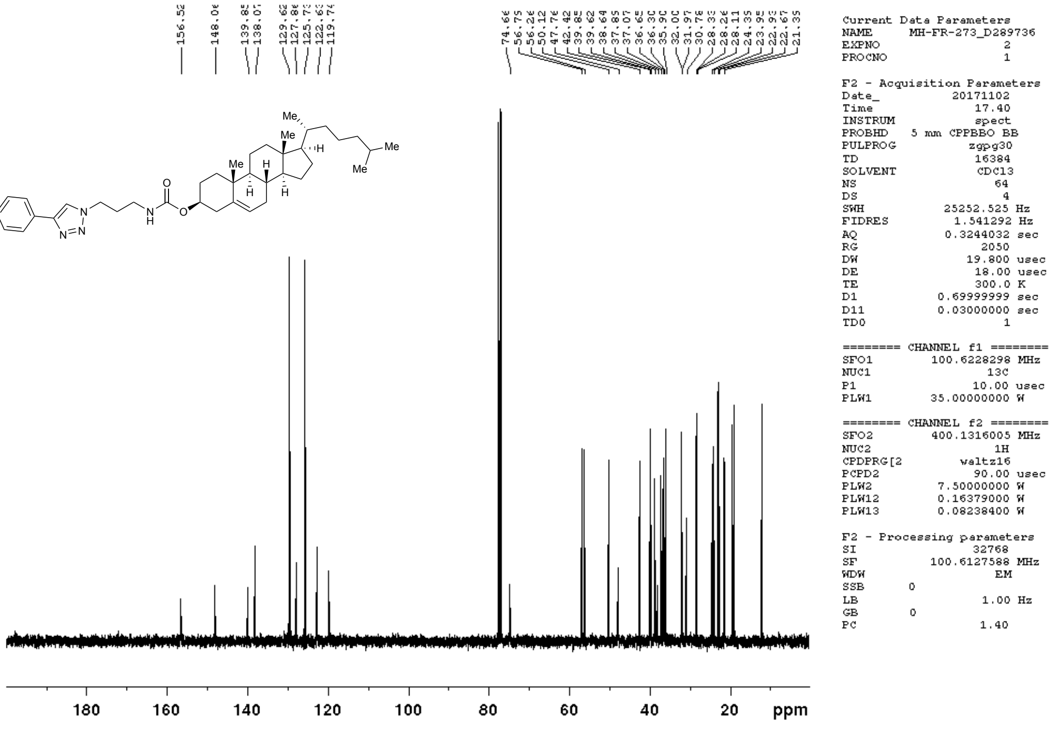

Supplementary Figure 115. ^{13}C NMR spectrum of **17c**.

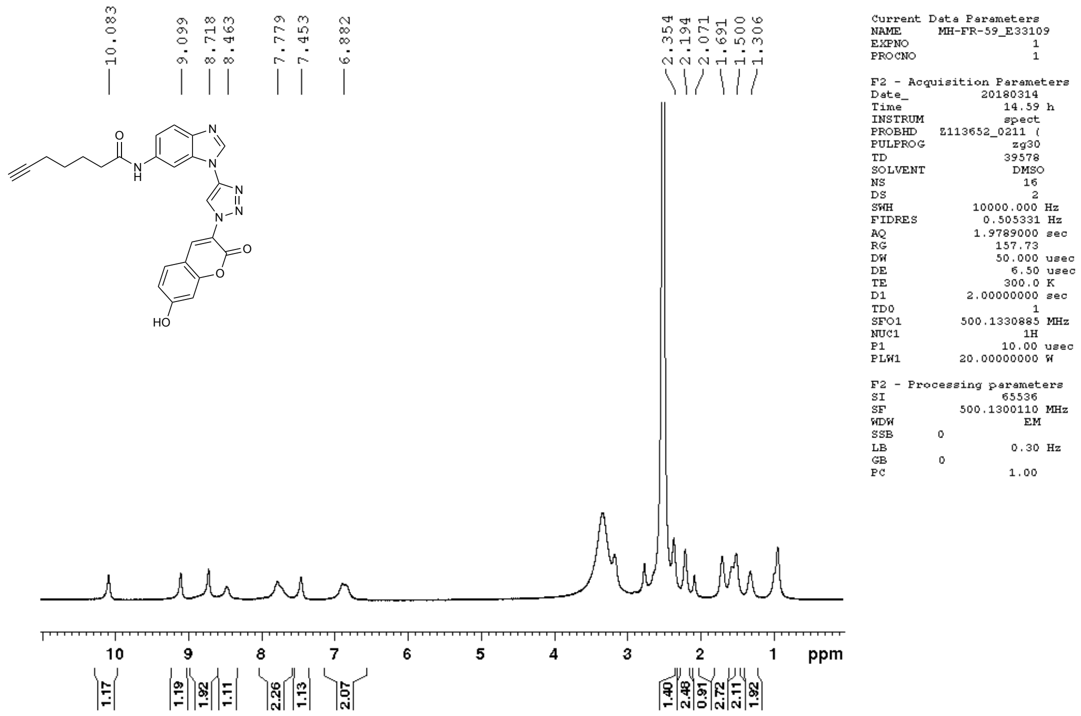

Supplementary Figure 116. ^{1}H NMR spectrum of **S2**.

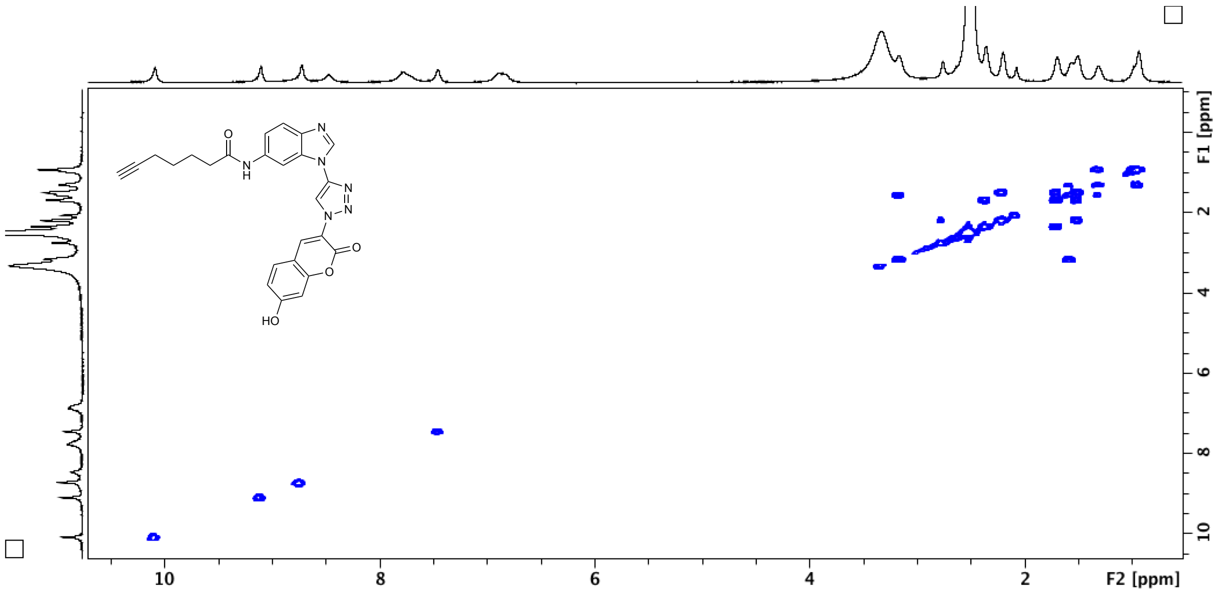

Supplementary Figure 117. COSY spectrum of **S2**.

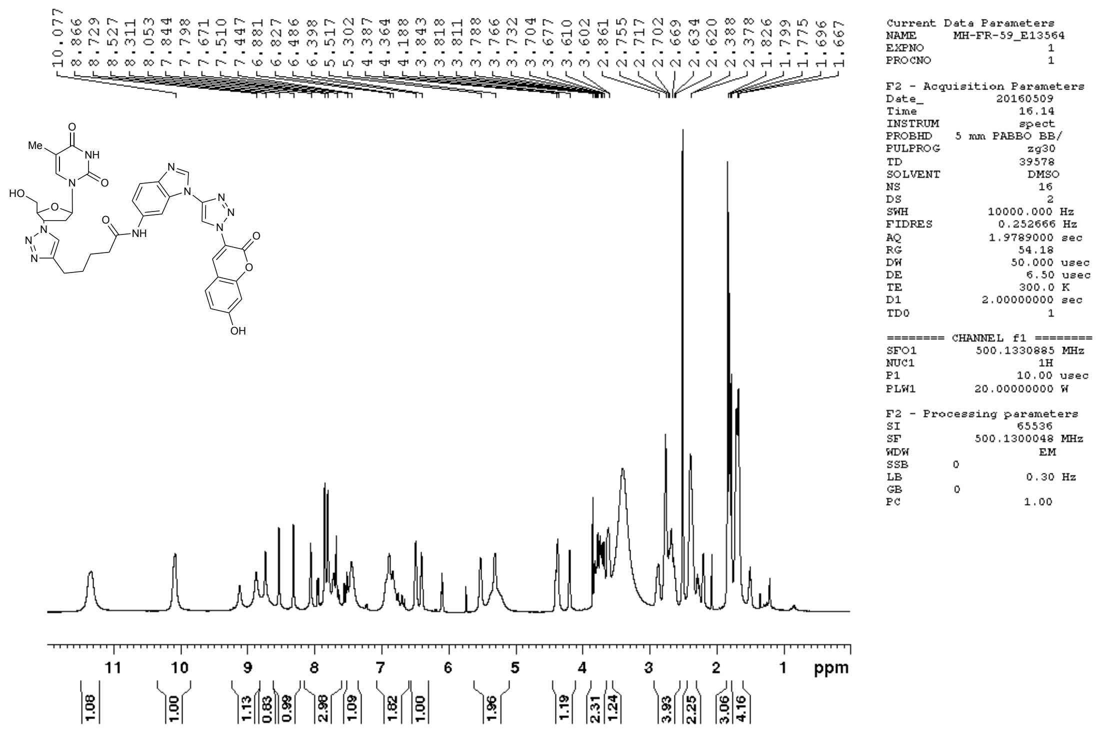

Supplementary Figure 118. ^{1}H NMR spectrum of **21**.

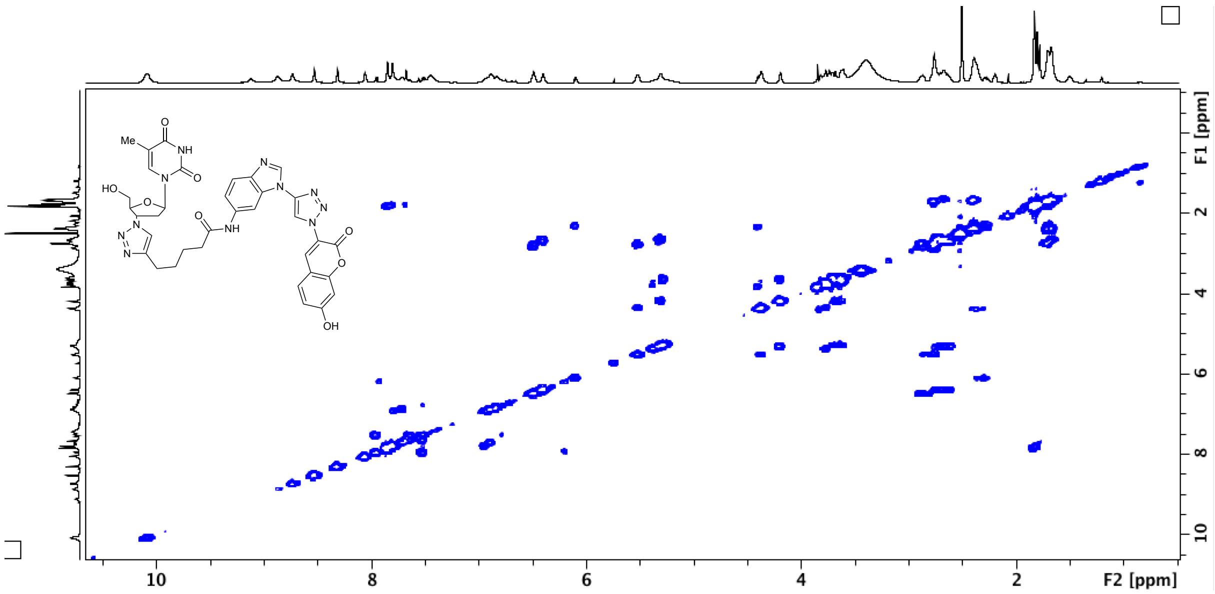

Supplementary Figure 119. COSY spectrum of **21**.

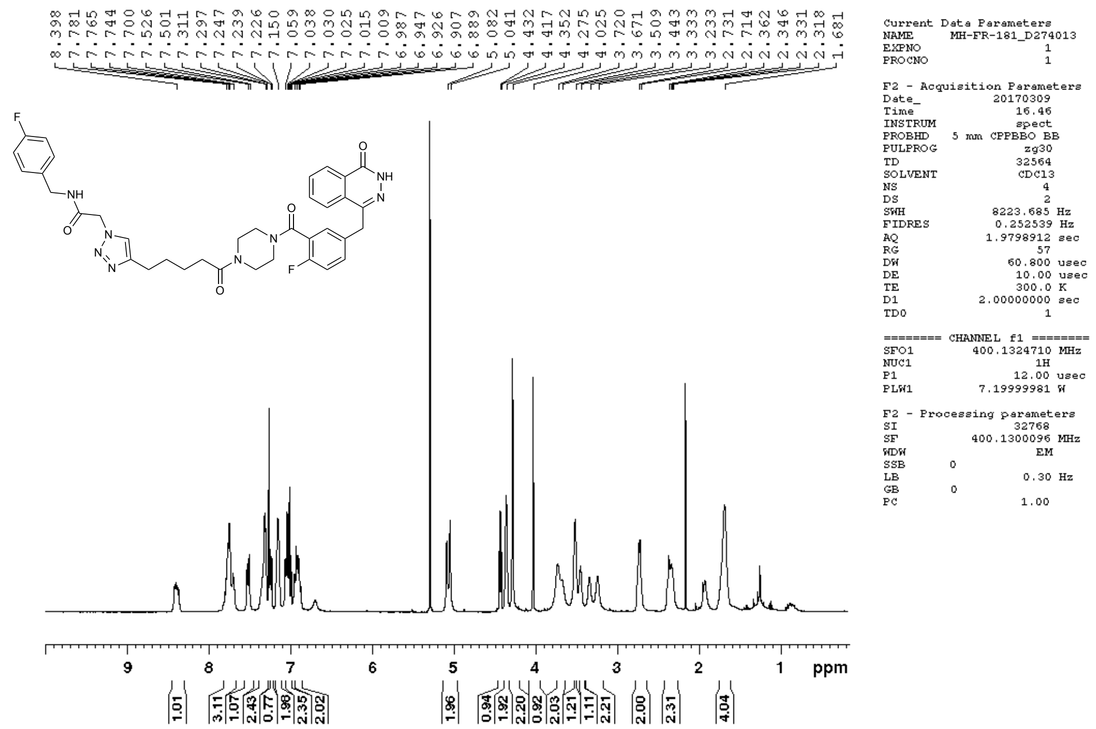

Supplementary Figure 120. ^{1}H NMR spectrum of **25**.

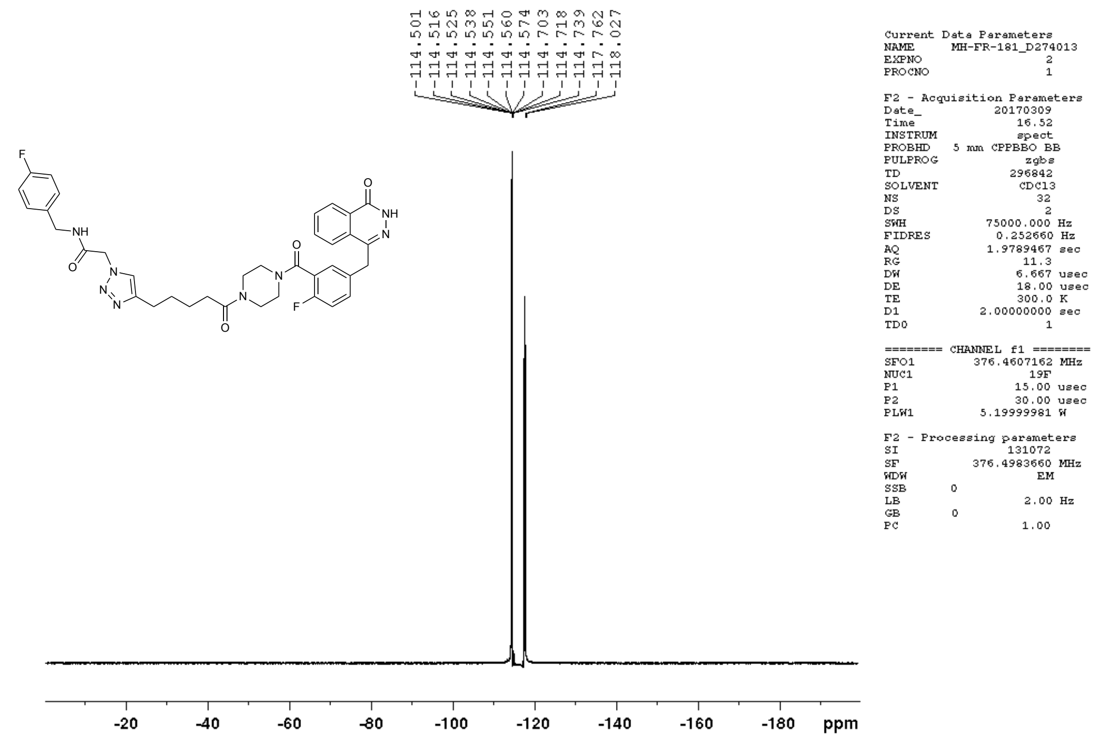

Supplementary Figure 121. ^{19}F NMR spectrum of **25**.

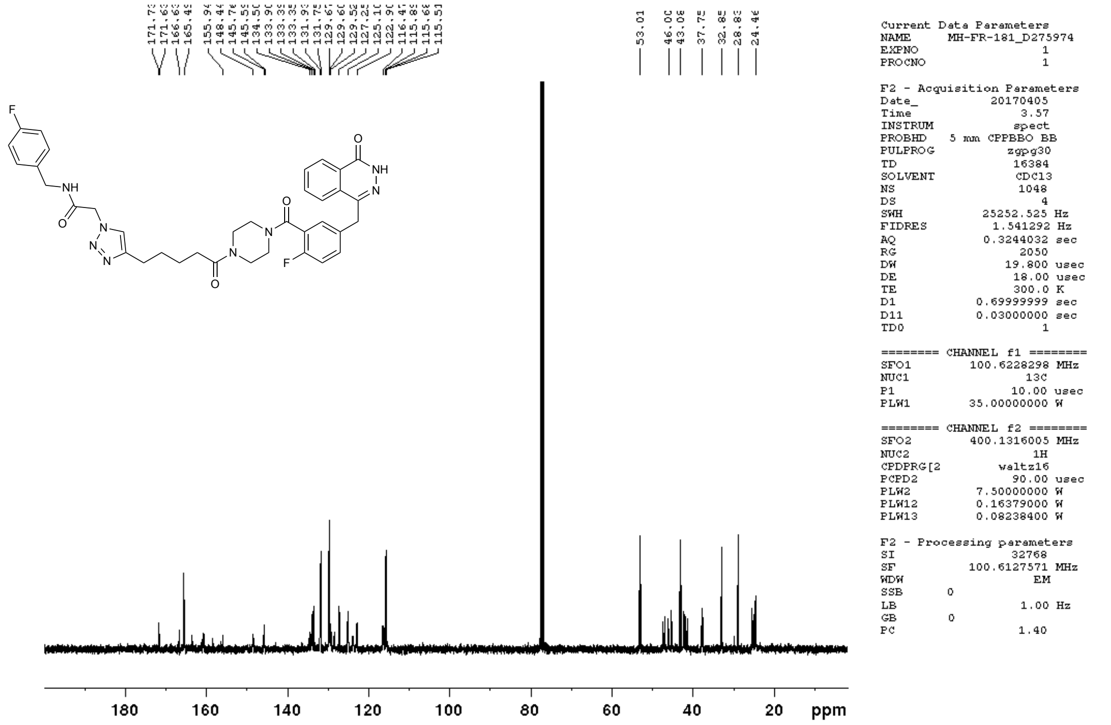

Supplementary Figure 122. ^{13}C NMR spectrum of **25**.

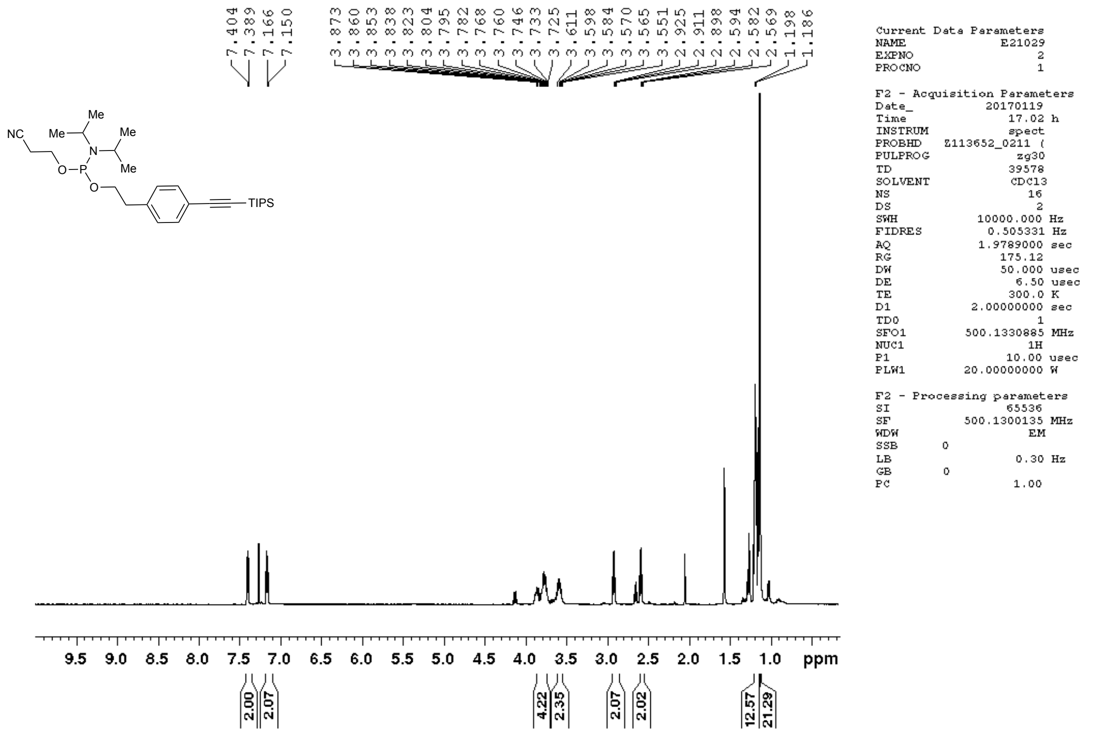

Supplementary Figure 123. ^{1}H NMR spectrum of **S3**.

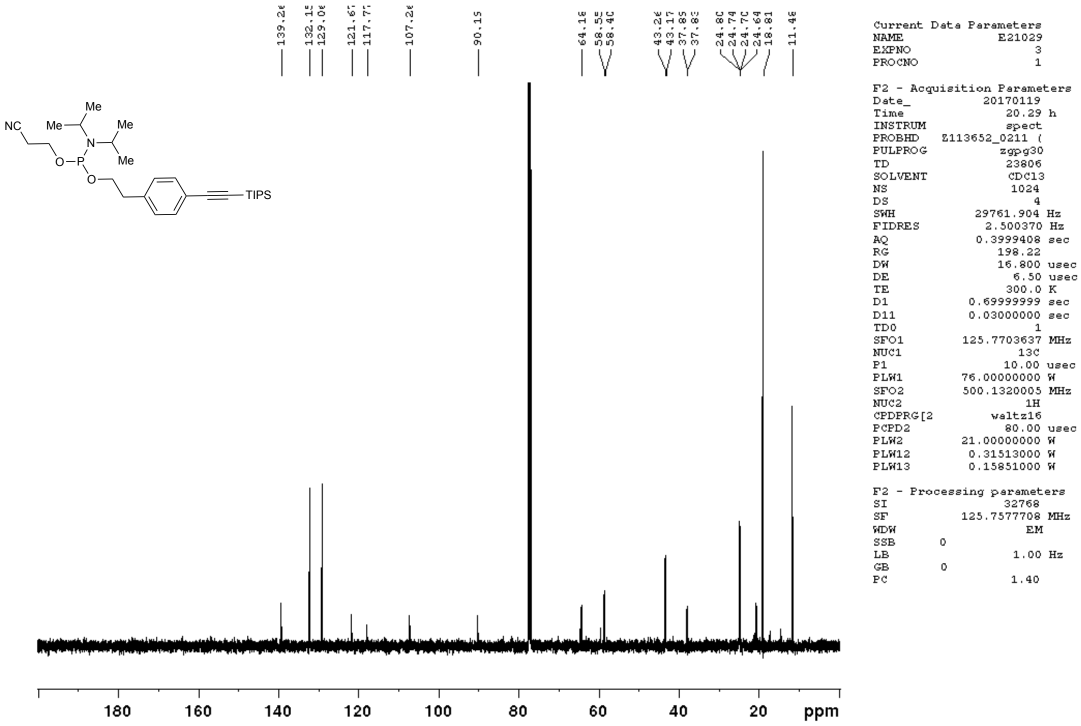

Supplementary Figure S124. ^{13}C NMR spectrum of **S3**.

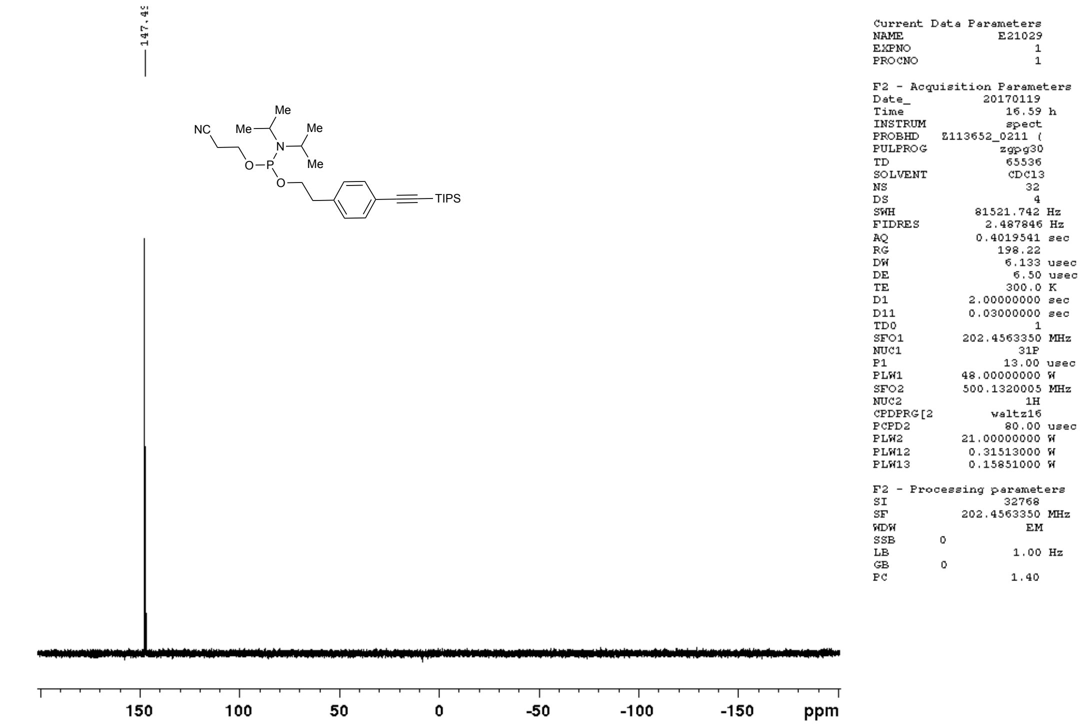

Supplementary Figure 125. ^{31}P NMR spectrum of **S3**.

Supplementary References

(1) Armarego, W. L., Perrin, D. *Purification of Laboratory Chemicals*, 5th Ed., Butterworth Heinemann (1996).

(2) Ovenall, D. W., Chang, J. J. Carbon-13 NMR of Fluorinated Compounds Using Wide-Band Fluorine Decoupling. *J. Magn. Reson.* **25**, 361–372 (1977).

(3) Hatit, M. Z. C., Seath, C. P., Watson, A. J. B., Burley, G. A. A Strategy for Conditional Orthogonal Sequential CuAAC Reactions Using a Protected Aromatic Ynamine. *J. Org. Chem.* **82**, 5461–5468 (2017).

(4) Mamidyala, S. K., Cooper, M. A. Probing the Reactivity of o-Phthalaldehydic Acid/Methyl Ester: Synthesis of *N*-Isoindolinones and 3-Arylaminophthalides. *Chem. Commun.* **49**, 8407–8409 (2013).

(5) Engler, A. C., Bonner, D. K., Buss, H. G., Cheung, Y., Hammond, P. T., Cheung, E. Y., Hammond, P. T., Cheung, Y., Hammond, P. T., Cheung, E. Y., Hammond, P. T., Cheung, Y. The Synthetic Tuning of Clickable PH Responsive Cationic Polypeptides and Block Copolypeptides. *Soft Matter* **7**, 5627–5637 (2011).

(6) Srinivasan, R., Uttamchandani, M., Yao, S. Q. Rapid Assembly and in Situ Screening of Bidentate Inhibitors of Protein Tyrosine Phosphatases. *Org. Lett.* **8**, 713–716 (2006).

(7) Sivakumar, K., Xie, F., Cash, B. M., Long, S., Barnhill, H. N., Wang, Q. A Fluorogenic 1,3-Dipolar Cycloaddition Reaction of 3-Azidocoumarins and Acetylenes. *Org. Lett.* **6**, 4603–4606 (2004).

(8) Wolfram, S., Wurfel, H., Habenicht, S. H., Lembke, C., Richter, P., Birckner, E., Beckert, R., Pohnert, G. A Small Azide-Modified Thiazole-Based Reporter Molecule for Fluorescence and Mass Spectrometric Detection. *Beilstein J. Org. Chem.* **10**, 2470–2479 (2014).

(9) Lin, P. C., Ueng, S. H., Tseng, M. C., Ko, J. L., Huang, K. T., Yu, S. C., Adak, A. K., Chen, Y. J. Lin, C. C. Site-Specific Protein Modification through CuI-Catalyzed 1,2,3-Triazole Formation and Its Implementation in Protein Microarray Fabrication. *Angew. Chem. Int. Ed.* **45**, 4286–4290 (2006).

(10) Ponader, D., Wojcik, F., Beceren-Braun, F., Dernedde, J., Hartmann, L. Sequence-Defined Glycopolymer Segments Presenting Mannose: Synthesis and Lectin Binding Affinity. *Biomacromolecules* **13**, 1845–1852 (2012).

(11) De Bruycker, K., Delahaye, M., Cools, P., Winne, J., Prez, F. E. D. Covalent Fluorination Strategies for the Surface Modification of Polydienes. *Macromol. Rapid Commun.* **38**, 1–6 (2017).

(12) Willibald, J., Harder, J., Sparrer, K., Conzelmann, K. K., Carell, T. Click-Modified Anandamide SiRNA Enables Delivery and Gene Silencing in Neuronal and Immune Cells. *J. Am. Chem. Soc.* **134**, 12330–12333 (2012).

(13) Wang, Q., Li, K., Chen, Y., Li, S., Nguyen, H. G., Niu, Z., You, S., Mello, C. M., Lu, X. Chemical Modification of M13 Bacteriophage and Its Application in Cancer Cell Imaging. *Bioconjug. Chem.* **21**, 1369–1377 (2010).

(14) Hatit, M. Z. C., Sadler, J. C., McLean, L. A., Whitehurst, B. C., Seath, C. P., Humphreys, L. D., Young, R. J., Watson, A. J. B., Burley, G. A. Chemoselective Sequential Click Ligations Directed by Enhanced Reactivity of an Aromatic Ynamine. *Org. Lett.* **18**, 1694–1697 (2016).

(15) Tale, R. H., Gopula, V. B., Toradmal, G. K. 'Click' Ligand for 'click' Chemistry: (1-(4-Methoxybenzyl)-1-*H*-1,2,3-Triazol-4-Yl)Methanol (MBHTM) Accelerated Copper-Catalyzed [3+2] Azide-Alkyne Cycloaddition (CuAAC) at Low Catalyst Loading. *Tetrahedron Lett.* **56**, 5864–5869 (2015).

(16) Zheng, Z. Shi, L. An Efficient Regioselective Copper-Catalyzed Approach to the Synthesis of 1,2,3-Triazoles from *N*-Tosylhydrazones and Azides. *Tetrahedron Lett.* **57**, 5132–5134 (2016).

(17) Zhang, X., Liu, P., Zhu, L. Structural Determinants of Alkyne Reactivity in Copper-Catalyzed Azide-Alkyne Cycloadditions. *Molecules* **21**, 1697–1714 (2016).

(18) Coelho, A., Diz, P., Caamaño, O., Sotelo, E. Polymer-Supported 1,5,7-Triazabicyclo [4.4.0] Dec-5-Ene as Polyvalent Ligands in the Copper-Catalyzed Huisgen 1,3-Dipolar Cycloaddition. *Adv. Synth. Catal.* **352**, 1179–1192 (2010).

(19) Santos, C. M., Kumar, A., Zhang, W., Cai, C. Functionalization of Fluorous Thin Films via "click" Chemistry. *Chem. Commun.* 2854–2856 (2009).

(20) Martin, M. M., Lindqvist, L. The PH Dependence of Fluorescein Fluorescence. *J. Lumin.* **10**, 381–390 (1975).

(21) Sirivolu, V. R., Vernekar, S. K. V., Ilina, T., Myshakina, N. S., Parniak, M. A. Wang, Z. Clicking 3′-Azidothymidine into Novel Potent Inhibitors of Human Immunodeficiency Virus. *J. Med. Chem.* **56**, 8765–8780 (2013).

(22) Betts, C., Saleh, A. F., Arzumanov, A. A., Hammond, S. M., Godfrey, C., Coursindel, T., Gait, M. J., Wood, M. J. Pip6-PMO, a New Generation of Peptide-Oligonucleotide Conjugates with Improved Cardiac Exon Skipping Activity for DMD Treatment. *Mol. Ther. - Nucleic Acids* **1**, 1–13 (2012).
