# Supplementary material for: A flow platform for degradation-free CuAAC bioconjugation
Source: Nat Commun. 2018 Oct 1;9:4021. doi: 10.1038/s41467-018-06551-0 (PMC6167327; doi:10.1038/s41467-018-06551-0)
Supplement: Supplementary file 2 — Peer Review File [file 41467_2018_6551_MOESM2_ESM.pdf]

Editorial Note: This manuscript has been previously reviewed at another journal that is not operating a transparent peer review scheme. This document only contains reviewer comments and rebuttal letters for versions considered at *Nature Communications.*

REVIEWERS' COMMENTS:

Reviewer #1 (Remarks to the Author):

The authors have adequately answered the questions of all the referees. This referee appreciates the efforts of the authors and believes that the paper can be accepted as is. This is a very nice publication of a flow method which can readily make an impact in the synthetic field.
